# Far-reaching hunter-gatherer networks during the Last Glacial Maximum in Western Europe

**DOI:** 10.1126/sciadv.adz7697

**Published:** 2026-01-21

**Authors:** Marta Sánchez de la Torre, Xavier Mangado, Samuel Castillo-Jiménez, Felipe Cuartero, Richard J. Hewitt, Luis Luque, Bernard Gratuze, Miguel Almeida, María de Andrés-Herrero, Guilhem Constans, Louis Marguet, Thierry Aubry, José J. Alcolea-González, Manuel Alcaraz-Castaño

**Affiliations:** ^1^ Seminar on Prehistoric Studies and Research (SERP), University of Barcelona, Barcelona, Spain.; ^2^ Institute of Archaeology of the University of Barcelona, Barcelona, Spain.; ^3^ Area of Prehistory, Department of History and Philosophy, University of Alcalá, Alcalá de Henares, Spain.; ^4^ Institute of Economy, Geography and Demography, CCHS CSIC, Madrid, Spain.; ^5^ Research Institute on Archaeomaterials (IRAMAT), CNRS and University of Orléans (UMR 7065),Orleans, France.; ^6^ Côa Park, Foundation for the Safeguarding and Development of the Côa Valley, Vila Nova de Foz Côa, Portugal.; ^7^ Dryas/Octopetala, Coimbra, Portugal.; ^8^ Department of Prehistory, Ancient History and Archaeology, Complutense University of Madrid, Madrid,Spain.; ^9^ TRACES Laboratory, UMR 5608, University of Toulouse, Tolouse France.; ^10^ Éveha, Limoges, France.; ^11^ PACEA Laboratory, UMR 5199, CNRS, University of Bordeaux, Bordeaux, France.; ^12^ UMR 7194 HNHP–National Museum of Natural History (MNHN), Paris, France.; ^13^ UNIARQ–Centre for Archaeology, University of Lisbon, Lisbon, Portugal.

## Abstract

Social networking is an essential feature of hunter-gatherer societies. It fosters the circulation of goods and information and enables kinship ties across different scales, including long-distance contacts. While such behaviors are known since at least the Upper Palaeolithic, evidence for geographically extensive social networks remains scarce. This evidence is limited to indirect inferences based on shared cultural traits, “art” styles, and symbolic items, while lithic raw material movements are mostly local and regional, with few cases exceeding 300 kilometers. We provide geochemical evidence for the largest confirmed distance between the source and discard location of a knapped lithic object in Palaeolithic Europe. Solutrean artifacts discarded at Peña Capón, Central Iberia, were sourced in Southwest France, 600 to 700 kilometers away. This demonstrates social networks of unprecedented geographic scale maintained during ∼1400 years during the Last Glacial Maximum. It also suggests that stone tools were exchanged as symbolic items to solidify social contacts and sustain far-reaching networks as risk-buffering mechanisms among widely dispersed hunter-gatherers.

## Teaser

Geochemistry of stone tools reveals unprecedently large social ties among European hunter-gatherers during the Last Ice Age.

## INTRODUCTION

The establishment of social connections at different spatial scales has long been recognized as a crucial mechanism for the successful adaptation of hunter-gatherer societies, past and present. The combination of kinship and mating ties, reciprocal relationships, cooperative activities, and exchanges of goods and information is essential for the survival of these societies across their worldwide ecological, cultural, and historical diversity (*1*–*9*). Yet understanding these networking behaviors is an enduring challenge when it comes to Palaeolithic foragers of the distant past. Despite much relevant work in recent decades (*8*, *10*–*25*), we are still far from achieving a comprehensive understanding of Palaeolithic social networks, especially concerning long-distance contacts, their frequency, extent and meaning.

Palaeogenetic evidence attests to the broad genetic similarity among widely geographically separated European populations during the Upper Palaeolithic, including high levels of connectedness at the continental scale during periods such as the Gravettian (*26*–*28*). This evidence includes the documentation of large mating networks associated with high individual mobility at the site of Sunghir, in the Siberian plain (*29*), resembling social and reproductive patterns of current hunter-gatherers aimed at limiting within-band relatedness and inbreeding (*2*, *4*). Social connectivity across wide areas is also suggested by the broad cultural uniformity of some Upper Palaeolithic technocomplexes at different scales (*29*, *30*), with the Solutrean at the scale of Southwest Europe (*15*, *31*), where genetic relatedness has been recorded among human populations before and after the Last Glacial Maximum (LGM) (*32*). Also, the geographical clustering in smaller cultural units during some periods has been related to ethnolinguistic variation within the wide framework of shared techno-cultural traits (*13*) as is the case with the Upper Solutrean (*15*).

Archaeological evidence for specific Palaeolithic networks beyond the regional level is mostly based on the circulation and distribution of clearly “non-utilitarian” items, such as decorative shells or figurines and rock art motifs (*13*, *14*, *20*, *22*, *31*–*36*). In contrast, studies on the circulation of lithic raw materials exploited for producing stone tools, based on their source and discard locations, mostly show local and regional procurement strategies. Movements exceeding 200 to 300 km are rarely suggested and mostly for the Upper Palaeolithic. Such long distances are often considered beyond the annual home range of hunter-gatherer bands and are hence interpreted in the context of geographically broad social networks (*16*–*18*, *25*, *37*). Yet, most of these cases are located in Eastern Europe, East Asia, and North America (*16*, *37*–*42*), while examples in Western Europe are scarce, albeit growing in recent years (*16*, *24*, *43*–*49*).

This scarcity of material evidence attesting to long-distance transfers of lithic raw materials is, to some extent, understandable given the essentially utilitarian character of stone tools. Also, in the framework of Human Behavioral Ecology, it is widely assumed that lithic procurement strategies are usually embedded in other subsistence and mobility strategies and ultimately related to the optimal exploitation of subsistence resources within the foraging area (*5*, *50*–*54*) [but see (*54*–*57*) for critical views]. Furthermore, the most common strategy when investigating the sources of lithic raw materials from a given site is to survey and analyze the outcrops in its immediate surroundings and adjacent territories (i.e., on a regional basis), often with little or no engagement with researchers working in more distant regions (*16*, *17*). All these factors could be behind the fact that there are still very few cases documenting lithic raw material circulation over distances of more than 300 km. However, the existence of broad social networks involving the circulation of lithic raw material across large areas as part of non-utilitarian mobility strategies (*14*) is yet to be widely explored (*23*).

Here, we present the results obtained from analyses of chert sourcing and mobility patterns of hunter-gatherers occupying the Peña Capón rock shelter (Muriel-Tamajón, Spain) during the LGM [sensu (*58*)]. This site is located in the central part of the Iberian Peninsula (Southwest Europe), in the southeastern foothills of the Central System Mountain range, in the northern area of the Iberian southern plateau, within the Upper Tagus River basin. Its stratigraphic sequence bears Upper Solutrean (level 0), Middle Solutrean (levels 1, 2a, 2b, and 3), Proto-Solutrean (level 4), and Terminal Gravettian (levels 5 and 6) technocomplexes between ∼26,200 and 22,200 calibrated years ago (cal B.P.) [text S1; see also (*59*)]. We conducted textural, micropalaeontological, and geochemical analyses of archaeological and geological chert samples, combined with Least Cost Analysis and techno-typological and use-wear studies of lithic assemblages. The statistical treatment of the geochemical data, combined with the macroscopic results, demonstrates that a substantial part of the cherts exploited throughout the sequence was sourced from various outcrops exceeding the regional level. More prominently, we show the existence of extensive long-distance contacts connecting central Iberia with southwest/Central France during the Middle Solutrean.

This unprecedented finding constitutes the largest confirmed distance between the source and discard locations of a lithic object, not only during the Solutrean (*24*, *60*, *61*) but during the whole European Upper Palaeolithic (*16*, *41*, *62*). Furthermore, it attests to the existence of broad social networks, maintained at least ∼1400 years, connecting regions separated by different mountain chains and different biogeographic areas during the cold episode of Heinrich Stadial 2 (HS2). This provides empirical confirmation of the long-standing hypothetical existence of large networks connecting the whole Solutrean geographic range (*15*, *18*, *31*, *63*–*65*). It also attests to the potential use of some stone tools as symbolic items exchanged to solidify social contacts among very distant groups and maintain large networks as risk-buffering mechanisms among Upper Palaeolithic hunter-gatherers (*14*).

## RESULTS

### Textural and micropalaeontological analysis

#### Archaeological assemblages

A total of 1041 lithic artifacts from Peña Capón were analyzed. Their textural and micropalaeontological characterization allowed the identification of five varieties from three sedimentary environments (fig. S25). The main macroscopic characteristics of each chert type and their distribution throughout the archaeological sequence are synthesized below and described at length in the Supplementary Materials (text S2.1):

1) Eight hundred seventy samples were classified as lithotype 1. Evaporitic chert type showing whitish colorations with a quite smooth original relict of a mudstone texture.

2) Thirty-three samples were classified as lithotype 2. Evaporitic chert showing reddish to brownish colorations in an originally mudstone to wackestone texture.

3) Fourteen samples were classified as lithotype 3. Evaporitic variety composed of light brownish to whitish cherts with an originally azoic mudstone texture.

4) Seventy samples were classified as lithotype 4. Lacustrine type composed of brownish colored cherts with a heterogeneous macroscopic variety of original textures ranging from wackestone to packstone type.

5) Thirty-two samples were classified as lithotype 5. Siliceous jasper variety showing orange to reddish colors with extremely smooth surfaces and an original mudstone to wackestone texture with inclusions of metal oxides. Some blackish spots, probably corresponding to metal oxides or amorphous organic matter, were present in some samples.

**Fig. 1.**
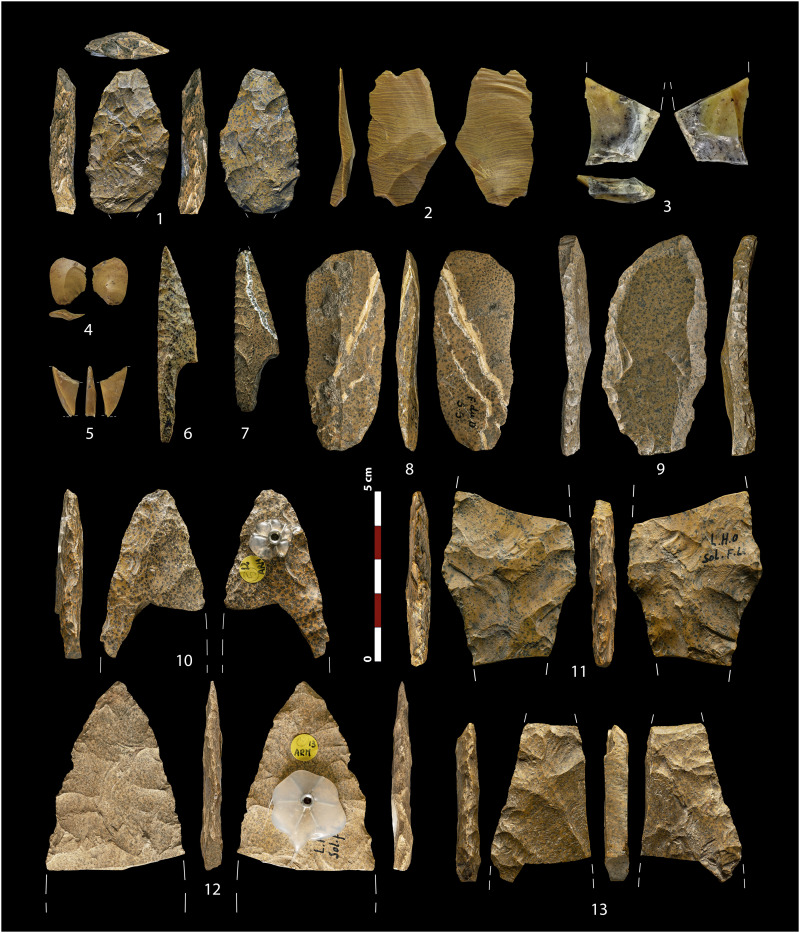
Jasperoid chert artifacts. 1 to 5: Solutrean tools from Peña Capón classified as lithotype 5 and confirmed by LA-ICP-MS as sourced from the Hettangian-Sinemurian outcrops west of the Central Massif (France) (1: PC23-11, 2: PC23-16, 3: PC23-17, 4: PC-43, 5: PC-36). 6 to 13: Artifacts from Solutrean sites in southwestern France produced on “Infralias” jasperoid cherts and confirmed by LA-ICP-MS (except 6 and 7, not analyzed by us) as sourced from the same Hettangian-Sinemurian formation. Le Placard: 6 and 7. Forneau du Diable: 8 (Fou-2-252). Combe Saunière: 9 (Com-2-37). Laugerie-Haute Ouest: 10 (Lau-1-284), 11(Lau-1-285) and 13 (Lau-1-286). Laugerie-Haute Est: 12 (Lau-2-286). Photo credits: M.A.-C., except 6 (Don Hitchcock) and 7 (T.A.).

The evaporitic cherts of lithotype 1 are always the most represented variety, with averages above 70% in most levels. The other types are found in different proportions throughout the sequence, with the jasper varieties only detected in the Middle Solutrean occupations (text S2.1). A notable finding was the unique features of one artifact from the Middle Solutrean level 3 defined as lithotype 5. This item, bearing a mottled dark brown color oolithic texture (see full description below) and classified as a preform of a Solutrean laurel leaf point (Fig. 1, 1), showed a set of macroscopic features which are not found in any other known chert or jasper formation in Iberia. In turn, they strongly resemble the jasperoid silicifications from the Hettangian-Sinemurian outcropping in the western border of the Central Massif, in France (*66*–*68*), located 600 to 700 km away from Peña Capón. These silicifications have been described in the archaeological assemblages of several sites from the Middle and especially Upper Palaeolithic of Southwest and central France, including Solutrean assemblages from sites such as Le Placard, Laugerie-Haute, or Le Maîtreaux (*60*, *69*, *70*) (Fig. 1, 6 to 13).

#### Geological samples

**Fig. 2.**
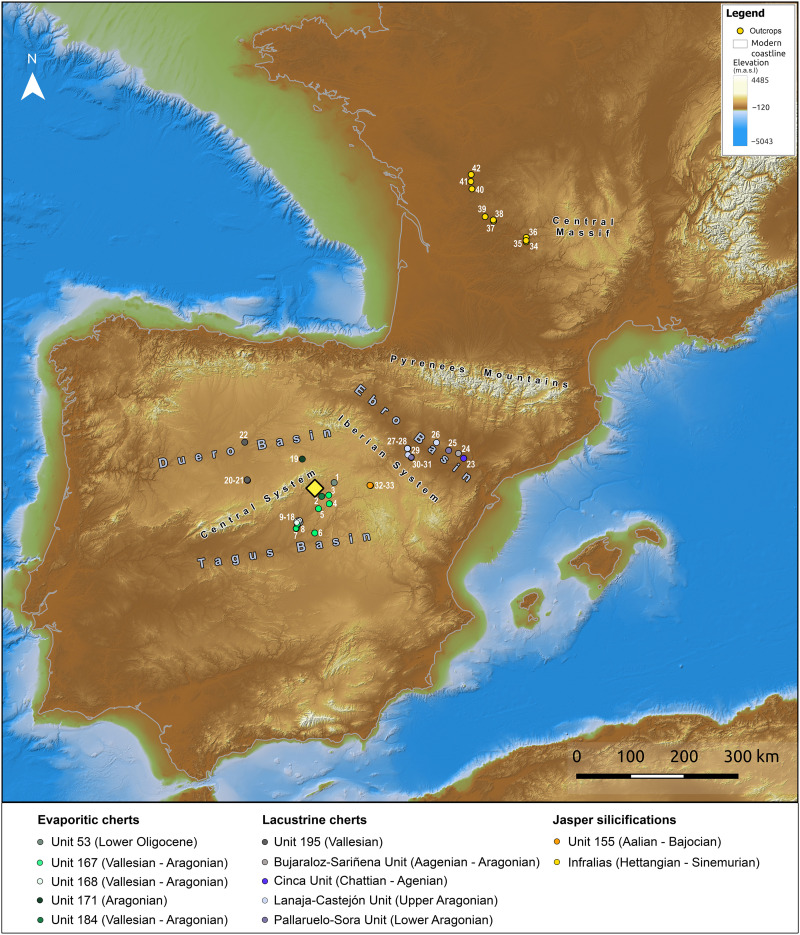
Location of studied geological units and outcrops. Geographic distribution of the units and outcrops selected for comparison with the archeological assemblages (for the numbering of outcrops, see dataset S1). m.a.s.l., meters above sea level.

**Fig. 3.**
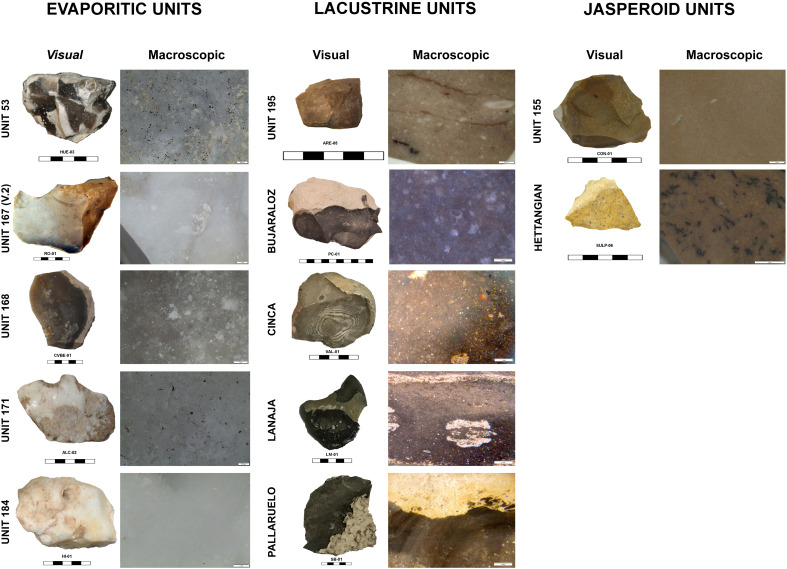
Studied geological units. Visual and macroscopic characteristics of the geological units selected for comparison with the archeological assemblages.

**Fig. 4.**
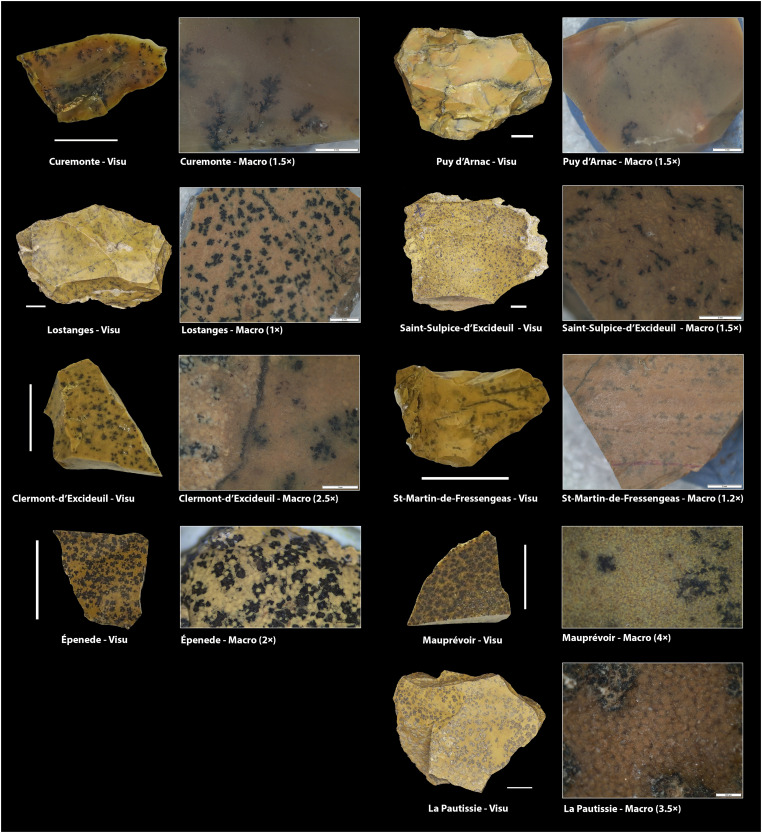
Geological samples from Hettangian-Sinemurian outcrops. Photos and captions at the stereoscopic microscope of the texture of geological samples from the Hettangian outcrops of the French Central Massif (see Fig. 2 and dataset S1 for the location of the outcrops).

Several geological formations, mostly outcropping in the Middle and Upper Tagus valley but also in the Duero and Ebro basins and Southwest France (Figs. 2 to 4 and dataset S1) show similar features to the archaeological assemblages. For the evaporitic cherts defined as lithotypes 1 to 3, we identified five geological units, defined by the Geological Survey of Spain (IGME). They correspond to IGME codes Geode 53, 167, 168, 171, and 184, and they consist of limestones, dolostones, and marls with chert from continental Cenozoic basins of the Duero and Tagus basins (see text S2.2 for a complete description of these units) (*71*).

For the lacustrine cherts of lithotype 4, we identified five geological units with similar macroscopic features. They correspond to Unit 195 (IGME Geode 50, Z2300) in the Middle Duero Basin and the units of Bujaraloz-Sariñena, Torrente de Cinca–Alcolea de Cinca, Sierra de Lanaja–Montes de Castejón, and Sierra de Pallaruelo–Monte de la Sora in the Middle Ebro basin (*71*, *72*). These units are described as cherts in marls and carbonate deposits from the Cenozoic continental facies of the Duero and Ebro basins (text S2.2 for a complete description).

**Fig. 5.**
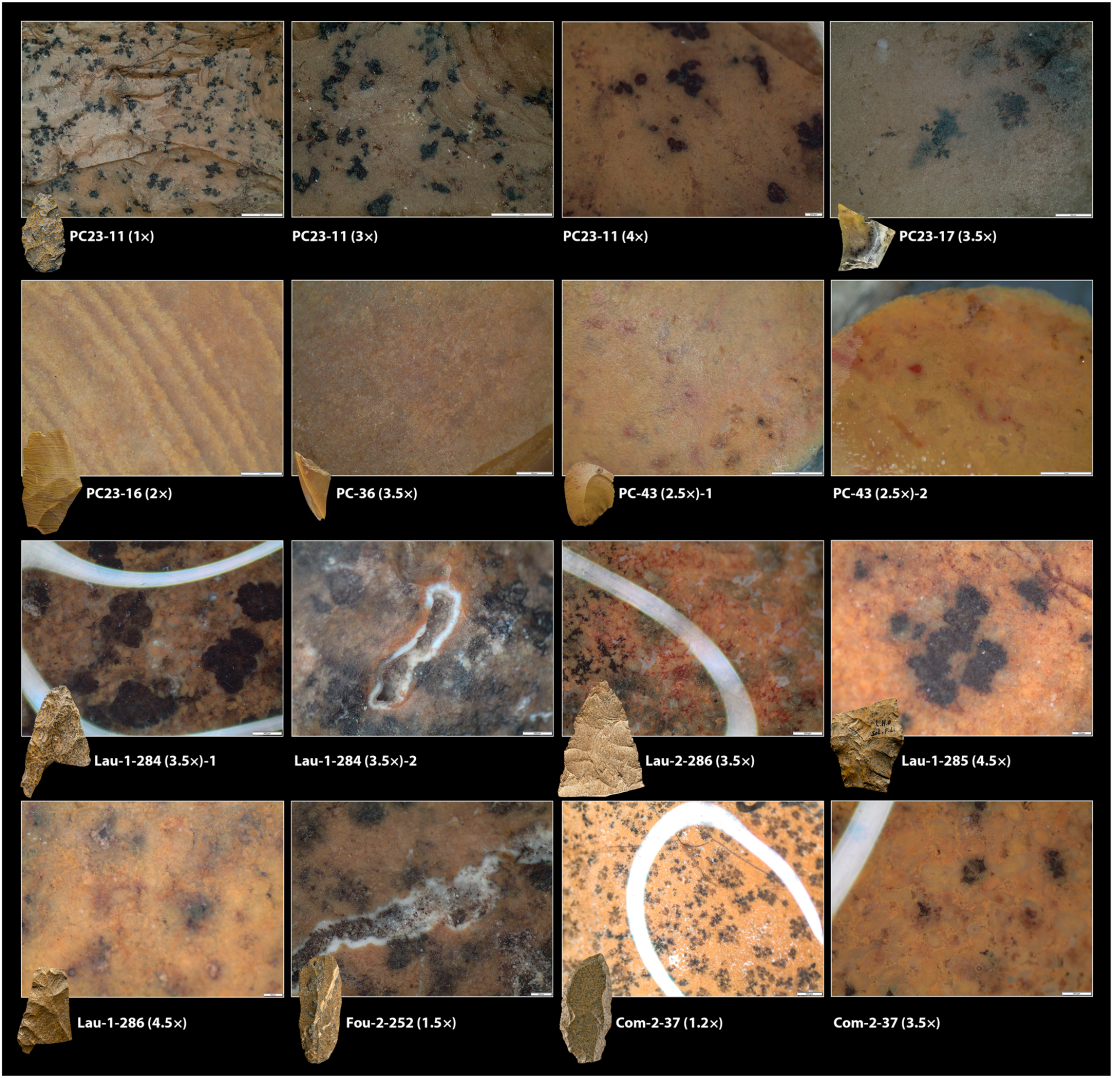
Archaeological jasperoid chert samples. Captions at the stereoscopic microscope of the texture of archaeological artifacts from Peña Capón (PC23-11, PC23-16, PC-36, and PC-43), Laugerie Haute (Lau-1-284, Lau-2-286, Lau-1-285, and Lau-1-286), Fourneau du Diable (Fou-2-252), and Combe Saunière (Com-2-37) made on Hettangian jasper silicifications. White lines in captions from Lau-1-284, Lau-2-286, and Com-2-37 correspond to water drops, as water was sometimes used for proper observation.

For the jasper silicifications of lithotype 5, most archaeological samples were similar to one single geological unit outcropping in the Upper Tagus basin, Unit 155 (IGME Geode 50, Z1700). Here, jasper nodules are embedded within the limestones from El Pedregal formation (Aalenian–Bajocian, Jurassic) (*71*). However, the bifacial preform from level 3 discussed above showed a clearly different fabric (Fig. 1, 1). This was characterized by relatively abundant dendritic blackish spots and some allogenic quartz crystals inclusions, narrow macroquartz veins with a previous white fibrous silica rim cement, and a generally well-preserved peloidal texture (Fig. 5). These features are not attested in any known Cenozoic evaporitic or jasperoid silicifications in Iberia and are unexpectedly similar to those of the jasperoids silicifications of carbonate sedimentary rocks from the Hettangian and Sinemurian ages (Lower Jurassic) outcropping west of the Central Massif area, in France (*66*–*68*). Thus, we selected samples from Corrèze (Curemonte, Lostanges and Puy-d’Arnac outcrops), Dordogne (Clermont-d’Excideuil, Saint-Martin-de-Fressengeas, and Saint-Sulpice-d’Excideuil outcrops), Charente (La Pautissie, Épenède) and Vienne (Mauprévoir). The main characteristics of the two units are presented in text S2.2.

At the macroscopic level, all these geological samples showed features allowing their outcropping formations to be characterized as likely catchment areas for the cherts exploited and discarded at Peña Capón during MIS 2. However, given the cultural and behavioral significance of most of these potential connections, especially those suggesting very long-distance contacts outside the Iberian Peninsula, we conducted geochemical analyses to cross-check interpretations and provide more accurate evidence on such hypotheses. Nevertheless, the macroscopic similarities between the discussed foliate preform from level 3 and the Hettangian–Sinemurian jasperoid outcrops of Southwest/Central France are notable, especially when considering archaeological data from other Solutrean sites in the Dordogne and Charente regions, such as Laugerie-Haute, Fourneau-du-Diable, Combe Saunière, and Le Placard (Figs. 1, 4, and 5). A sample of artifacts from the first three sites, potentially sourced from these jasperoid formations, were thus included in our study, both at the macroscopic and geochemical levels (dataset S3).

### Geochemical analysis

#### Evaporitic and lacustrine cherts

**Fig. 6.**
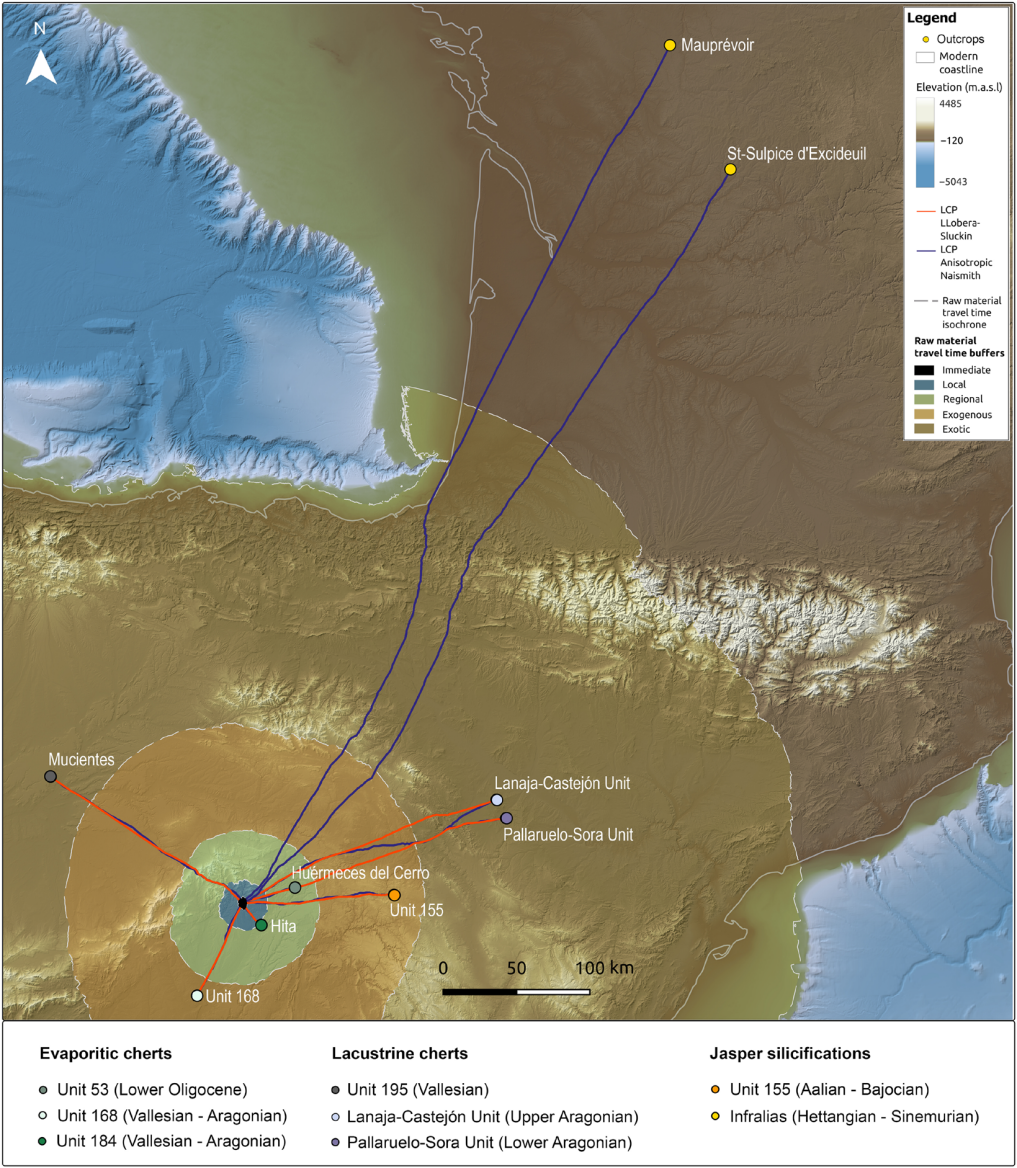
Least cost paths to lithic raw material sources and travel time buffers. Least cost paths connecting Peña Capón with all the geological units and outcrops shown by the geochemical analysis to be sources of the cherts discarded at the site throughout the Solutrean and Proto-Solutrean human occupations. Raw material time-cost classification is also represented (see text S4 for discussion).

Forty-seven evaporitic cherts recovered throughout the Peña Capón sequence were compared with 300 geological samples from five geological units by means of laser-ablation inductively coupled plasma mass spectrometry (LA-ICP-MS). The results indicate that they mostly come from the regional outcrops of Huérmeces del Cerro (Unit 53) and Hita (Unit 184), as well as from different outcrops within Unit 168, in the Madrid Miocene basin. For the lacustrine cherts, 38 artifacts macroscopically attributed to lithotype 4 were compared with 146 geological samples from five geological units located in the Duero and Ebro basins. The results indicate that their sources were the Mucientes outcrop (Unit 195) in the Duero Basin—probably also including other as-yet unidentified outcrops from this unit—and the Lanaja-Castejón (La Muela 1, La Muela 2, and Campo de las Horgas outcrops) and Pallaruelo-Sora Units (San Borombón 1 and San Borombón 2 outcrops), in the Middle Ebro Basin (Figs. 2 and 6 and datasets S2 and S3). Detailed results of the geochemical analysis of evaporitic and lacustrine cherts are presented in text S3.

#### Jasperoid cherts

Eighteen archaeological samples macroscopically attributed to lithotype 5 were analyzed by LA-ICP-MS. These artifacts were compared to 135 geological samples from two regions: the jaspers from Unit 155, outcropping at the northeast of the Guadalajara province, and the Hettangian-Sinemurian jasperoids from the western border of French Central Massif (Fig. 2). As discussed above, four archaeological samples from Laugerie-Haute, one from Fourneau-du-Diable, and one from Combe Saunière were also included in the study under the same analytical conditions (dataset S3).

**Fig. 7.**
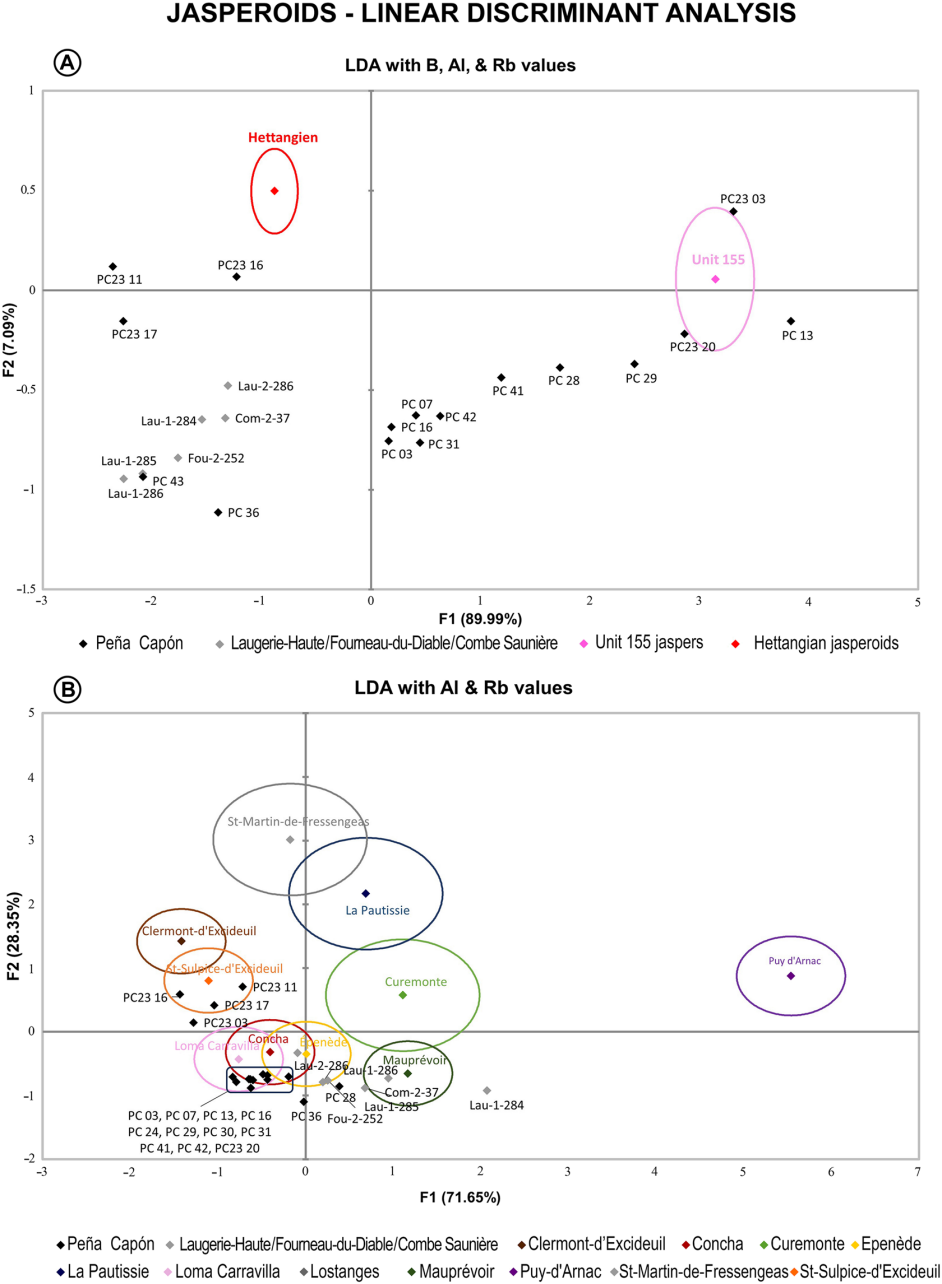
LDA showing the studied jasperoid cherts. (**A**) Separated by geological units and concerning B, Al, and Rb values. (**B**) Separated by outcrops and concerning Al and Rb values. For the geological units, only the centroids are represented, with 95% ellipse for confidence interval.

The statistical analysis of the LA-ICP-MS results revealed that B, Si, Al, and Rb values presented the largest SDs between geological units and hence were suitable tracers for the distinction between sources. Therefore, we carried out a first linear discriminant analysis (LDA) on B, Al, and Rb values grouping the geological units and individualizing each archaeological sample. The resulting plot (Fig. 7A), with F1 (89.99%) and F2 (7.09%) of the variability represented, clearly separated the two geological units. Most of the archaeological samples from Peña Capón were within or near the dispersion area of the Unit 155 cluster on the plot. However, five of them (PC23-11, PC23-16, PC23-17, PC-36, and PC-43) were closely placed near the dispersion area of the Hettangian cluster, as were the samples from Laugerie-Haute, Fourneau-du-Diable, and Combe Saunière. PC-43 was placed strictly overlapping one of the samples from Laugerie-Haute (Lau-1-286) (Fig. 1). We then performed a LDA with Al and Rb values (Fig. 7B), separating the geological samples by outcrops. Whereas most samples from Peña Capón were placed in the overlapping area of Loma Carravilla and Concha clusters (both outcrops from Unit 155), four of them were located in the dispersion area of Saint-Sulpice-d’Excideuil cluster, from the Hettangian sources. Two more samples from Peña Capón (PC-36 and PC-28) were located in a similar dispersion area as most of the artifacts from Fourneau-du-Diable, Combe Saunière, and Laugerie-Haute, being all closely located to the Mauprévoir and Épènede Hettangian clusters. This second LDA allowed discarding the geological outcrops of Puy-d’Arnac, Saint-Martin-de-Fressengeas, La Pautissie, and Curemonte as potential sources, as they were located far away from the dispersion areas of the archaeological samples.

**Fig. 8.**
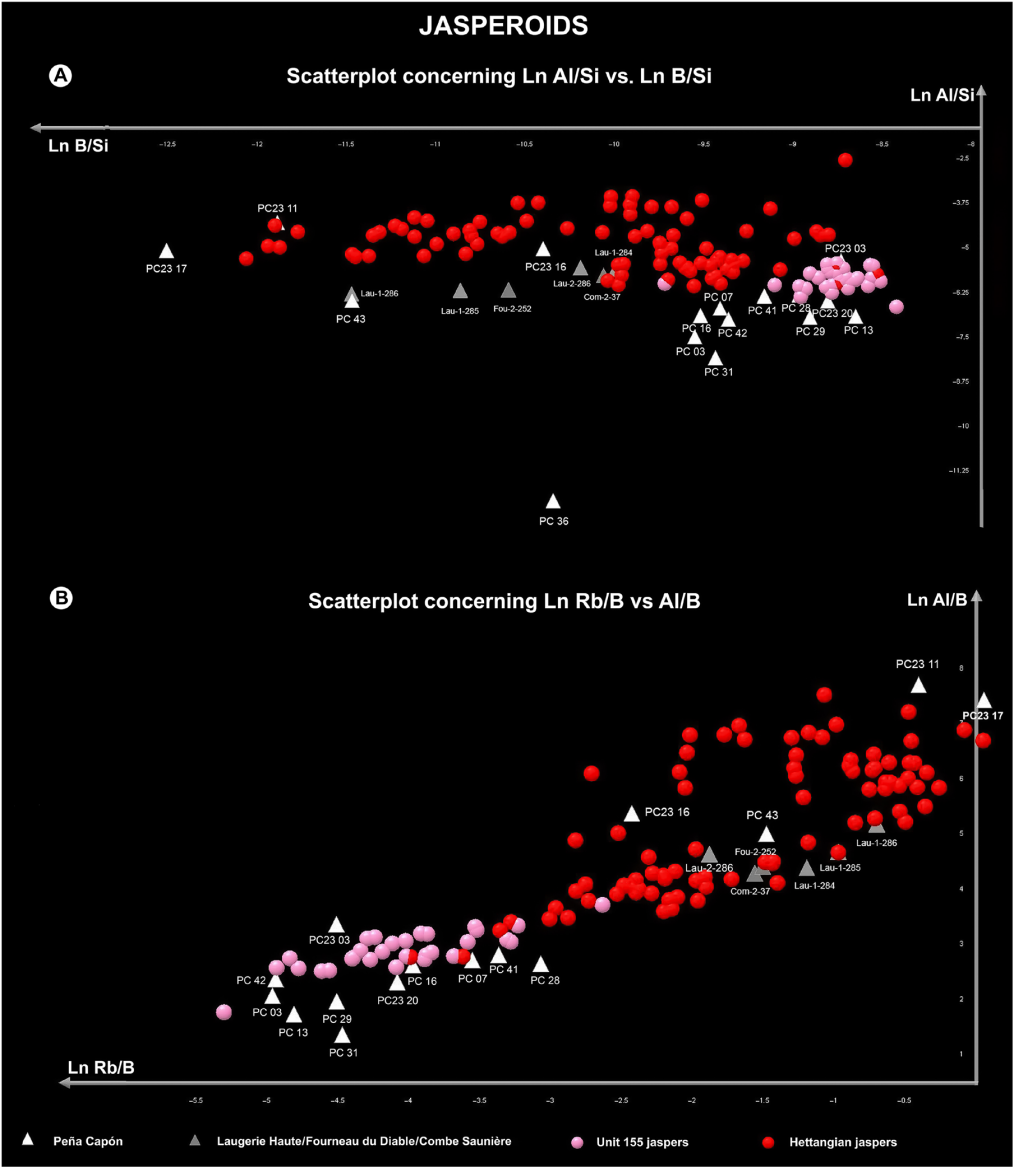
Scatterplots showing archaeological jasperoid cherts and geological units. (**A**) Results for La Al/Si versus Ln Rb/Si, including all the geological samples separated by geological units and the sampled archaeological artifacts from Peña Capón and the Dordogne region of SW France. (**B**) Same for Ln Rb/B versus Al/B.

Next, we made two scatterplots representing all the geological samples by units and the archaeological artifacts. The first, based on Ln Al/Si versus Ln B/Si (Fig. 8A), showed a very clear separation between the two geological units. The Peña Capón samples were mostly located in the dispersion area of jaspers from Unit 155, whereas samples PC23-11, PC23-16, PC23-17, and PC-43 were placed in the dispersion area of the Hettangian jaspers, thus confirming the LDA attributions. PC-36 was located far from the main dispersion area and hence was considered an outlier. The artifacts from Laugerie-Haute, Fourneau-du-Diable, and Combe Saunière were all located in the dispersion area of the Hettangian sources. Again, PC-43 almost completely overlapped Lau-1-286. The second scatterplot, based on Ln Rb/B versus Al/B values (Fig. 8B), showed very similar results, with PC23-11, PC23-16, PC23-17, and PC-43 situated again in the dispersion area of the Hettangian geological samples, and PC-36 located outside the main dispersion area (not present in the plot). Samples from Laugerie-Haute, Fourneau-du-Diable, and Combe Saunière were also placed in the dispersion area of the Hettangian samples and near PC-43.

**Fig. 9.**
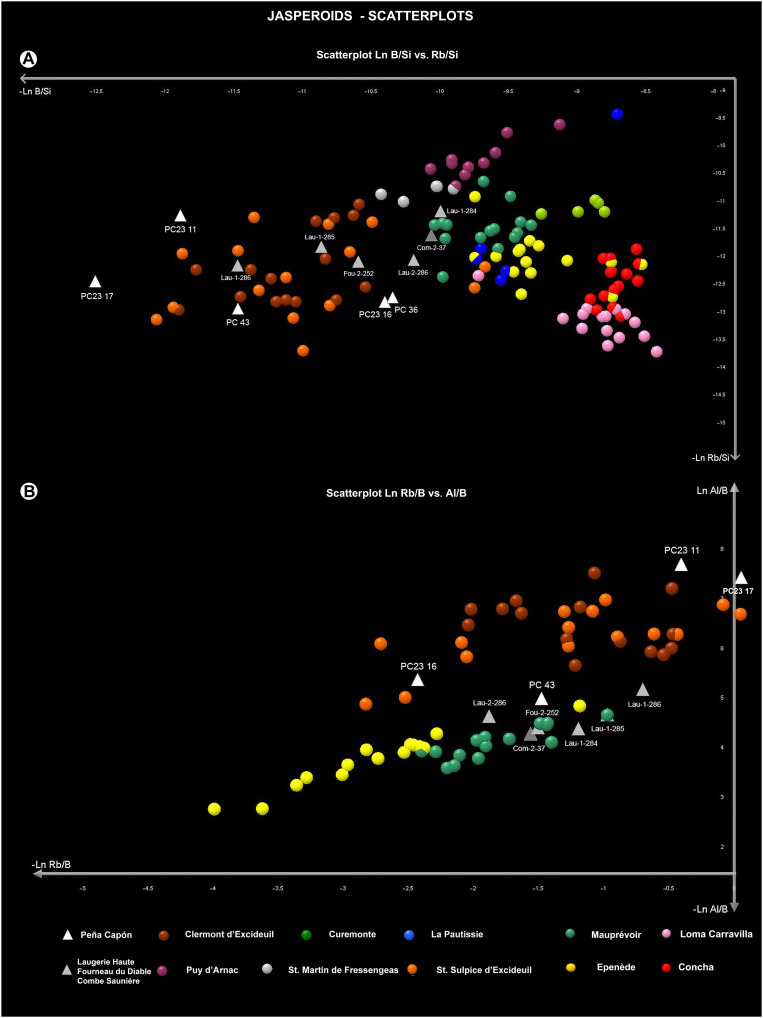
Scatterplots showing archaeological jasperoid cherts and outcrops from SW France. (**A**) Results for Ln B/Si versus Ln Rb/Si, including all the geological units separated by outcrops, the archaeological samples from the Dordogne region, and the Peña Capón samples previously located close to the Hettangian jasperoids. (**B**) Same for Ln Rb/B versus Al/B and including only the Hettangian outcrops previously located close to the archaeological samples.

Last, we made two scatterplots separating the geological samples per outcrop and including only the archaeological samples that had shown a previous connection to the Hettangian jaspers. The first plot, concerning Ln B/Si versus Rb/Si, placed most of the archaeological samples in the dispersion area of Saint-Sulpice-d’Excideuil and Clermont-d’Excideuil clusters, which were overlapping. However, Lau-1-284 and the sample from Combe Saunière (Com-2-37) were most closely related to the Mauprévoir cluster (Fig. 9A). In the second plot, for Ln Rb/B versus Al/B and including only the Hettangian outcrops that had been previously located close to the archaeological samples, PC23-11, PC23-16, and PC23-17 were placed in the dispersion area of Saint-Sulpice-d’Excideuil and Clermont-d’Excideuil, which were again overlapping. However, sample PC-43 and all samples from Combe Saunière, Fourneau-du-Diable, and Laugerie-Haute were most closely located to the Mauprévoir cluster (Fig. 9B).

Overall, the LA-ICP-MS analysis confirms that at least four archaeological artifacts from the archaeological sequence of Peña Capón (PC23-11, PC23-16, PC23-17, and PC-43) have their geological source in the Hettangian jaspers from southwest France. Three of them are chemically connected to the Saint-Sulpice-d’Excideuil and Clermont-d’Excideuil outcrops (Dordogne), while it is unclear if sample PC-43 originated in these same outcrops or in the Mauprévoir outcrop (Poitou-Charente) to the north. The archaeological samples from the Solutrean occupations of Laugerie-Haute, Fourneau-du-Diable, and Combe Saunière could be related to the Saint-Sulpice and Clermont-d’Excideuil outcrops according to the first plot (Fig. 9A) or to the Mauprévoir outcrop according to the second plot (Fig. 9B). However, there is certainly a close relationship between sample PC-43 from Peña Capón and sample Lau-1-286 from Laugerie-Haute, as in most plots they are strictly placed in the same area. The results demonstrate that both the Peña Capón and the French archaeological samples were sourced from the same geological formation, albeit from different outcrops in some cases, during the Solutrean.

An important outcome of our analysis is that some of the Peña Capón samples that were revealed by LA-ICP-MS to have been sourced in the Hettangian-Sinemurian formation west of the Central Massif show notable differences at the surface level with regard to both the geological samples and known archaeological artifacts from such formation. This is the case of samples PC23-16 (Fig. 1, 2), PC-43 (Fig. 1, 4), and PC-36 (Fig. 1, 5) (Fig. 5), which are macroscopically different not only from the French samples but also from PC23-11 (Fig. 1, 1). This shows that the macroscopic approach alone is not sufficient to detect the high variability present in some geological units or even within a single outcrop (Fig. 4). The mentioned samples would have been assigned to Unit 155 or most probably left unassigned to a specific geological unit, in the absence of a geochemical study. They would also have been misclassified without the previous preliminary assignment of PC23-11 to the Hettangian jasperoids based on its macroscopic features, which in this case were very clear.

#### Least cost analysis

Analysis of the time and physical effort needed for accessing the geological units and outcrops identified as sources for the lithic artifacts discarded at Peña Capón has shown that both relatively close and distant rocks were present at the site throughout the sequence of human occupation (Fig. 6 and dataset S4). Although no local cherts have been documented, a substantial proportion of the exploited rocks (in most cases above 70% according to the macroscopic study; see text S2.1) is evaporitic cherts acquired at the regional level, including the outcrops of Hita and Huérmeces del Cerro, both accessible in roundtrips taking between 1 and 2 days (text S4). However, a smaller but substantial number of cherts are exogenous rocks sourced in the evaporitic formations of the Manzanares/Jarama rivers area in the Madrid province (Unit 168) ∼72 km away, in need of roundtrips of more than 3 days to be directly procured. Also exogenous are the jaspers from the Loma Carravilla and Concha outcrops (Unit 155), present in all layers ranging from 0.9 to 6.6%, and located in the Upper Tagus basin, more than 5 days away. A further category of “exotic” materials, appearing also in all Solutrean and Proto-Solutrean levels ranging from 2.6 to 18.5%, is constituted by the lacustrine cherts from Mucientes (Duero Basin) and the Lanaja-Castejón and Pallaruelo-Sora units (Middle Ebro basin), located 8.7 and 10.5 days away, respectively. Last, the jasperoid cherts from the French Hettangian outcrops, present in very few quantities only in the Middle Solutrean layers except level 1 (one object or 0.64% in level 2a, one object or 0.31% in level 2b, and one object or 0.65% in level 3, plus two objects recorded as surface findings; see dataset S3) are considered “ultra-exotic.” They traveled a distance of 620.2 km in the case of those from Saint-Sulpice-d’Excideuil, and 671.7 km for those sourced at Mauprévoir. From Peña Capón, this would entail a return journey of 284.4 hours, equivalent to 35.6 days (dataset S4 and text S4).

## DISCUSSION

**Fig. 10.**
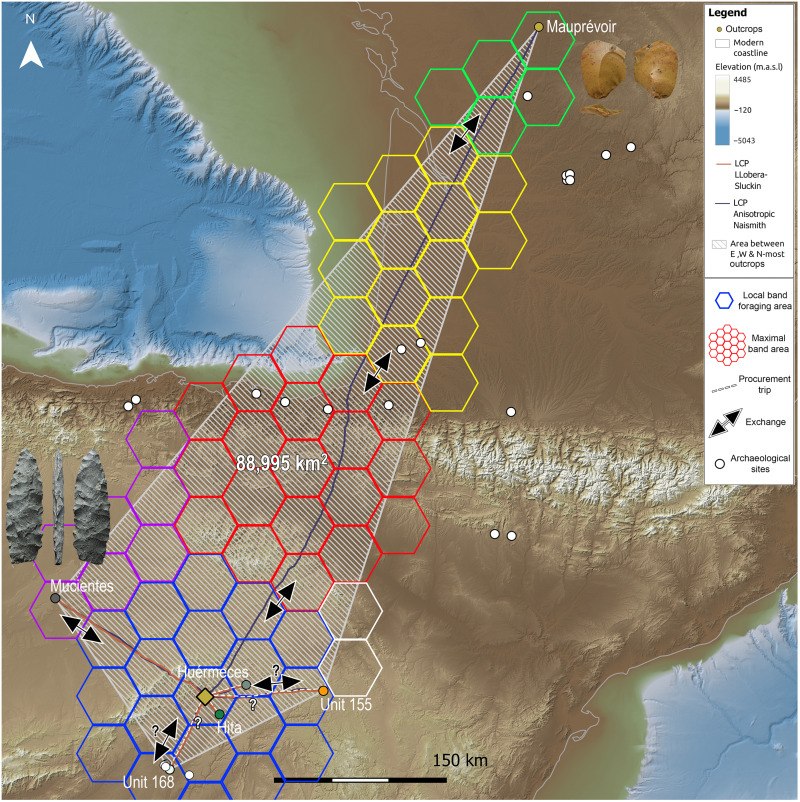
Social networks between central Iberia and SW France during the Middle Solutrean. Heuristic model showing the social network defined by the sources of lithic raw materials discarded at Peña Capón level 2b as indicated by LA-ICP-MS and least cost analyses. We show the potential procurement trips and exchanges between “minimal” and “maximal” hunter-gatherer bands (*14*), as well as the known archaeological sites within or close to the network limits (see text S5 for discussion).

The presence of exogenous and exotic rocks in all Proto-Solutrean and Solutrean levels from Peña Capón demonstrates the existence of a complex network of raw material circulation maintained for at least ∼3200 years. While regional and even exogenous rocks could be the product of direct catchment, either as part of embedded procurement or as a result of special-purpose trips, the presence of exotic, and especially ultra-exotic materials at the site, attests to the existence of very long-distance contacts and hence of geographically broad social networks, especially during the Middle Solutrean. This is demonstrated, in the first place, by the least-cost distances between the site and the sources of the exotic and ultra-exotic rocks—and the time needed to cover them (dataset S4). In levels 2a, 2b, and 3, where the ultra-exotic Hettangian jasperoids from Southwest France are present, such distances are well beyond the longest documented straight-line distances covered by ethnographic hunter-gatherers during foraging trips (text S4). Furthermore, the sizes of the areas defined by the source locations of the rocks discarded at the site in levels 2a, 2b, and 3 are not consistent with any typical hunter-gatherer mobility pattern. There is not a single hunter-gatherer group in the ethnographic record whose annual land-use range even approaches such sizes. Not even highly mobile subarctic hunter-gatherers, like the Baffinland Inuit, who might cover an annual territory of around 25,000 km^2^ [or even less, according to Ellis (*37*): note 1], are close to the 88,995 km^2^ indicated by data of Peña Capón level 2b (Fig. 10 and text S5). Also, considering that the Hettangian jasperoids do not account for the best knapping quality among the rocks discarded at the site, it is unlikely that their presence, limited to one retouched flake fragment in level 2a, one small flake in level 2b, and one laurel leaf preform in level 3 (plus one thinning flake and a flake fragment recorded as surface findings), was the result of direct procurement due to “utilitarian” mobility (see texts S4 and S5).

Therefore, it is very unlikely that at least exotic and ultra-exotic rocks were directly procured by the same people discarding them at Peña Capón. The existence of large social networks, comprising, in the case of the Middle Solutrean, the largest distances documented to date in the Western European Paleolithic record, is both the most parsimonious and groundbreaking interpretation of the results. When considering the mean population sizes of hunter-gatherer groups and their exploitation areas, both archaeological and ethnographic (*5*, *12*, *14*, *51*), the network connecting central Iberia to Southwest France cannot be explained only by the participation of a limited number of bands. Given the geographic distances involved, this network should have included a sufficiently large number of “maximal bands,” grouping an even larger number of interconnected “minimum” or local bands (*3*, *4*, *8*, *9*, *14*), participating in a down-the-line trade process allowing for different exchange processes across the landscape (text S5) (*73*). Although it is not possible to know the exact number of exchange events and the distances travelled by the different human groups involved, we illustrate this networking process with a heuristic model based on Whallon’s (*14*) estimates for the spatial organization of Upper Palaeolithic hunter-gatherer bands and their territories (text S5). This model, applied to the data of all Proto-Solutrean and Solutrean levels of Peña Capón, represents the social networks and potential exchanges operating in a given time, as shown by the location of the sources for the rocks discarded at the site in each sedimentary level (figs. S38 to S43).

The largest network, indicated by the data of level 2b, suggests the participation of at least six different adjacent maximal bands involving different exchange events for transporting a small amount of jasperoid chert from Mauprévoir to Peña Capón, among other exchange processes involving other raw materials (Fig. 10). This pattern, which is similar in levels 2a and 3, demonstrates the maintenance of large social networks connecting different human groups through regions separated by several mountain chains and different biogeographic areas between at least ∼25.3 and 23.9 ka cal B.P., partly coincident with the cold episode of Heinrich Stadial 2 (fig. S11). This reinforces the idea that such networks operated as “safety nets” to mitigate risk during periods of environmental stress and resource uncertainty among scattered groups (*14*, *74*), hence enabling the transmission of social knowledge by means of non-utilitarian mobility and eventually driving and shaping cultural evolution (*21*) and cumulative culture (*2*, *6*, *75*, *76*). It also strengthens the contentious hypothesis that Pleistocene hunter-gatherers were involved in cooperative large-scale networks more often than previously believed (*77*). Moreover, it provides further support to the hypothesis that the central region of Iberia was not a mere crossing area for hunter-gatherers during the coldest stages of the Last Glacial (*59*, *78*). Conversely, our data show that the Peña Capón region and most probably other regions of inland Iberia were not disconnected from the densely populated regions of northern Iberia and Southwest France, long-considered as glacial refugia for humans, plants, and animals during the climate deterioration of the LGM (*31*). Connections between both sides of the Pyrenees during this time frame have been documented at both ends of this mountain range (*45*, *46*, *79*), including long-distance movements of lithic raw materials (*24*), thus demonstrating recurrent social contacts within southern France and northern Spain. However, data from Peña Capón demonstrate that the social networks established in those territories extended at least to Central Iberia, thus attesting to a far wider area of social connectivity than previously thought. Furthermore, our results suggest that the Peña Capón region potentially functioned as an aggregation area, where dispersed bands seasonally came together as part of their social and subsistence needs (*5*, *18*, *80*, *81*). This is suggested not only by the presence at the rock shelter of exotic and ultra-exotic lithic raw materials from at least five outcrops from different regions through a prolonged period of time (Fig. 6 and figs. S29 to S34) but also by the existence of other penecontemporary sites in the nearby territory, including La Malia rock shelter (*82*) and El Reno cave, where pre-Magdalenian rock art depictions are found (fig. S24) (*83*). Furthermore, some images from El Reno, located just 9 km away from Peña Capón (fig. S1), include stylistic features strongly resembling other cave art motifs in the Dordogne and Charente regions, thus reinforcing the existence of a network allowing the circulation of shared ideas between Southwest France and Central Spain throughout this period (text S6).

However, the current archaeological record for the period in which this network was operating does not show the density of sites expected to allow such a broad social system to be maintained in the long-term (Fig. 10 and text S5) (*84*). Our results strengthen the hypothesis that this is due to the absence of systematic research in these areas, and they enable us to predict that new sites will be found once further research is carried out (*17*, *18*, *59*, *78*, *84*–*86*). Our down-the-line exchange model also predicts that jasperoid cherts from Southwest France, as well as other rocks from regions within the broad network (including Central Iberia), will be found in these and other sites in the defined area, following a pattern in which the shorter the distance to the source, the larger the amount of a given raw material (*73*, *87*).

Our results support previous hypotheses on the existence of cultural connectivity across large areas of western Europe since the onset of the climatic crisis triggered by HS2 and the LGM, as reflected by the cultural homogeneity of the Solutrean and its rapid dissemination during its early and middle phases (*15*, *17*, *18*, *31*, *63*–*65*), and its posterior cultural regionalization during the Upper Solutrean (text S5) (*15*, *88*). Yet, we demonstrate that the social networks operating during the Middle Solutrean were unexpectedly broad, involved the circulation of lithic raw materials across vast distances, and included central Iberian regions previously considered unsuitable for hosting substantial cultural developments and social networks during the LGM due to ecological constraints (*31*, *53*, *84*).

These results open previously unexplored research avenues, first by bringing our attention to the potential existence of other unexpectedly large social networks connecting bands from distant Eurasian regions during the Upper Palaeolithic, hitherto unexplored. Second, by establishing the fact that non-utilitarian mobility and the circulation of ideas and goods through very large geographic areas were not limited to explicit symbolic items, but it also included stone tools. This led to the hypothesis that the artifacts produced on “French” jasperoids that ended up in Central Iberia during the Middle Solutrean had some sort of symbolic meaning for the societies transporting and exchanging them, as it is ethnographically documented for some hunting weapons used as social gifts (*5*). Although the potential symbolic meaning of these stone tools is difficult to ascertain (*56*), in addition to their distant origin, the distinctive macroscopic features of the foliate preform from Peña Capón level 3, unparalleled in the Iberian geological and archaeological contexts (Fig. 1, 1), suggest that this could be the case. Furthermore, the technological study of this object, including its diacritical reading, combined with the use-wear analysis of its surfaces, provides additional support to this hypothesis (text S7). These studies showed that the piece, besides not being a finished artifact in the techno-typological sense, was never used as a projectile or cutting tool. They also showed the presence of wear traces on its ventral and dorsal surfaces suggesting that it was transported after the piece was knapped, probably as such from the place where it was produced (figs. S49 and S50). These additional results thus strengthen the potential non-utilitarian and symbolic meaning of this artifact and consequently also that of the other ultra-exotic Hettangian jasperoids discarded at Peña Capón. In sum, the data suggest that the large social network connecting Southwest France to Central Iberia, sustained for at least ∼1400 years, was not only intended at mitigating subsistence risk but also maintained social and cultural cohesion among different local and regional groups by promoting the circulation of ideas and symbols.

## MATERIALS AND METHODS

### Textural, micropalaeontological, and geochemical approaches

With the aim of defining the textural and micropalaeontological features of the lithic assemblages from Peña Capón, the first step of our study was the macroscopic characterization of the cherts exploited throughout the sequence of human occupation recorded at the site. This involved the macroscopic description of a sample of 1041 lithic remains, which corresponds to most of the retouched artifacts and a selection of nonretouched products throughout the sequence. These objects mostly come from the excavation conducted in a central area of the site during the 2015, 2019, 2021, and 2022 seasons. This area, dubbed “Central Pit,” comprised the excavation of 13 m^2^, to which a 2-m^2^ “geotrench” was added in 2021. A small number of artifacts (*n* = 19) come from a previous excavation conducted in 1972, in an area between our Central Pit and “Upper Pit” (text S1.2 and fig. S6). The stratigraphic provenience for all of these artifacts is secure, since a precise correlation between the archaeological levels defined in the old and new excavations has been established, based on stratigraphic, radiocarbon, and archaeological data (texts S1.3 and S1.4). Nine hundred sixty-one samples come from the Solutrean levels, which are the focus of our research: 3 from level 0, 327 from level 1, 156 from level 2a, 320 from level 2b, and 155 from level 3. Twenty-seven samples come from the Proto-Solutrean level 4, and 45 come from the Terminal Gravettian levels: 27 from level 5 and 18 from level 6. The remaining eight samples were surface findings (table S6). The relatively uneven distribution of the sampled materials throughout the sequence is due to the smaller excavation area conducted to date in level 0 and levels 5 and 6 and the lower presence of cherts in levels 5 and 6 (text S1).

The textural and micropalaeontological characterization of the lithics were carried out using a binocular microscope Olympus SZ61 (from ×6.7 to ×45 magnification). Images were taken using a coupled Olympus SC30 camera. As the aim of the study was to compare the archaeological items with geological chert samples, nondestructive techniques were prioritized.

To cross-check results obtained in the micropalaeontological and textural study and provide quantitative and reproducible data, a further step of our analytical approach was geochemical analysis. This was intended to quantify major, minor, and trace components to accurately compare the raw materials of the artifacts with those from geological outcrops. Thus, 103 chert artifacts from Peña Capón were analyzed by LA-ICP-MS. We focused on the Solutrean and Proto-Solutrean occupations, as cherts from the Terminal Gravettian levels (5 and 6) were few and less diverse (text S3 and dataset S3). Archaeological tools without cortex and surface alterations were preferred.

For the LA-ICP-MS analyses, samples were grouped according to the sedimentary environment of the different macroscopic varieties. Thus, lithotypes 1 to 3, as defined in the macroscopic study, were grouped as evaporitic cherts and studied as a whole. This decision was taken considering the large macroscopic variety observed during the macroscopic and geochemical analysis of the geological samples, as the variability of the evaporitic cherts at the scale of a given outcrop was very large. Furthermore, the statistical treatment of the geochemical data confirmed that the macroscopic varieties did not correspond to different groups in terms of chemical elemental composition. Thus, for the geochemical analysis, macroscopically defined lithotypes 1 to 3 were considered evaporitic cherts, lithotype 4 was referred to as lacustrine cherts, and lithotype 5 as jaspers. In dataset S3, we provide the median values and the SD in parts per million for each geological source, as well as the values for all the archaeological samples.

To quantify major, minor, and trace elements, LA-ICP-MS was conducted at the IRAMAT laboratory (Orleans, France). Elements were quantified using a Thermo Fisher Scientific Element XR mass spectrometer associated with a Resonetics RESOlution M50e ablation device. This spectrometer has the advantage of being equipped with a dual mode (counting and analogue modes) secondary electron multiplier with a linear dynamic range of over nine orders of magnitude, associated with a single Faraday collector which allows an increase in the linear dynamic range by an additional three orders of magnitude. This feature is particularly important for laser ablation analysis of lithic samples, as it makes it possible to analyze major, minor, and trace elements in a single run regardless of their concentrations and their isotopic abundance. The ablation device is an excimer laser (ArF, 193 nm), which was operated at 7 to 8 mJ and 20 to 30 Hz depending on the sample and only if saturation was observed where conditions reduced to 10 Hz. A dual gas system with helium (0.65 liters/min) released at the base of the chamber, and argon at the head of the chamber (1.1 liters/min) carried the ablated material to the plasma torch. Ablation time was set to 40 s: 10-s pre-ablation to let the ablated material reach the spectrometer and 30-s collection time. Laser spot size was set to 100 μm, and only reduced to 80 or 50 μm if saturation was detected, and line mode acquisition was chosen to enhance sensitivity. Background measurements were run every 10 to 20 samples. Fresh fractures were analyzed on geological samples to reduce potential contamination. Priority was given to characterizing large samples; thus, only one ablation line was carried out per specimen. However, if element spikes due to the presence of inclusions or heterogeneities were observed during analysis, results were discarded, and a new ablation location was selected.

Calibration was performed using standard reference glass NIST610 which was run periodically (every 10 to 20 samples) to correct for drift. NIST610 was used to calculate the response coefficient (*k*) of each element (*89**,* *90*), and the measured values of each element were normalized against 28Si, the internal standard, to produce a final percentage. Glass Standard NIST612 was analyzed independently of calibration to provide comparative data. Thirty elements were quantified (Li, Be, B, Mg, Al, Si, Ca, Ti, V, Cr, Fe, Ga, Ge, As, Rb, Sr, Y, Zr, Nb, Cs, Ba, La, Ce, Pr, Nd, Sm, W, Bi, Th, and U), although measures corresponding to Be, Ca, and Cr were usually below the detection limits, so they were not considered for interpretation. The results were statistically analyzed using the XLSTAT software (*91*). LDA was prioritized for discriminating between sources, as previous studies have shown its value in the characterization of lithic tools (*92*, *93*) and scatterplots were used to produce graphical results. To generate the scatterplots, we first conducted descriptive statistical analyses to identify those elements that showed the highest SD among geological formations, thus favoring discrimination between sources.

To compare results obtained after the analysis of the archaeological assemblages, we surveyed and sampled chert geological formations showing similar characteristics in central Iberia and other regions. We first focused on the regional area of Peña Capón in the Middle and Upper Tagus River basin, including the fluvial valleys of the Sorbe, Jarama, and Badiel rivers in Guadalajara province (Fig. 2 and fig. S1) and the Manzanares and Jarama valleys, as well as the interfluvial platform between both, in Madrid province. A large number of chert formations are known in these regions (*71*, *94*–*99*). The surveyed areas were chosen based both on the existing literature and the available digital geological map of Spain, scale 1:50.000 (GEODE Project) from the Spanish Geological Survey (IGME). Then, with the aim of exploring potentially larger procurement areas or long-distance contacts, we included several geological formations in the Duero and Ebro basins, based both on the macroscopic features of some archaeological items and the preliminary results obtained in a previous archaeopetrological and petrographic study (*71*). Other areas previously considered as potential sources for the cherts discarded at Peña Capón along the Tagus valley, such as the Portuguese Estremadura and the Lusitanian Basin (*100*), were discarded after a macroscopic inspection of representative cherts from these regions. Furthermore, as one archaeological item from Peña Capón showed notable similarities at the macroscopic scale with the jasperoids silicifications of carbonate sedimentary rocks from the Hettangian and Sinemurian ages (Lower Jurassic) outcropping in the western border of the French Central Massif (*67*–*69*), we also surveyed and thoroughly sampled this formation. The whole geological sampling involved up to 42 outcrops (mostly primary outcrops) from 12 geological formations or units in Spain and France (Fig. 2), from which 606 samples were selected for geochemical analyses (table S7). To improve analysis time and to avoid surface alterations, geological samples were prepared in squares of 5 mm by 5 mm, removing cortex surfaces.

Last, research on the potential connections between Peña Capón and the Hettangian-Sinemurian formations of Southwest France was also based on the study and macroscopic characterization of a sample of lithic artifacts from the Solutrean layers of Laugerie Haute (Les Eyzies-de-Tayac, Dordogne), Fourneau-du-Diable (Bourdeilles, Dordogne), and Combe Saunière (Sarliac-sur-l’Isle, Dordogne). A relevant number of lithic items from these and other sites from Southwest France, including also Le Placard (Vilhonneur, Charente), showed notable similarities with both the Hettangian-Sinemurian geological samples and at least one piece from Peña Capón, and hence they were included in our study. After the macroscopic characterization of 32 items from these sites, six of them were selected for the geochemical study: four from Laugerie Haute, one from Fourneau-du-Diable, and one from Combe Saunière (dataset S3). These artifacts are permanently stored at the National Museum of Prehistory in Les-Eyzies-de-Tayac (France), where they were macroscopically analyzed. Permissions for their temporary transfer to the IRAMAT laboratory at Orléans to develop geochemical studies were obtained from the French Ministry of Culture.

### Least cost analysis

Methods for calculating Least Cost Paths (LCPs) and anisotropic (accounting for uphill or downhill direction) travel times from Peña Capón to the outcrops and geological units demonstrated by the LA-ICP-MS to be sources for the lithics discarded at the site are based on the following workflow (see text S4 for discussion): First, we obtained digital terrain model (DTM) information at 1–arc sec resolution from NASA Shuttle Radar Topography Mission, equivalent in our study area to a resolution of ~30 m. Because of the large number of map tiles that needed to be merged to cover France and Iberia, the data were resampled and projected to ETRS89 UTM30N (EPSG code 25830) at a resolution of 100 m, which is suitable for our research objectives. We estimated the vertical root mean square error (RMSE) between the original and the resampled dataset using 1000 randomly selected control points, giving a value of 7.74 m. Thus, taking into account the RMSE estimates for the original dataset of 9.73 m (*101*), this would give an average overall RMSE of 8.735 m, assuming equal weighting for both RMSE computations. The coastline and coastal plain at the LGM, corresponding to ~120 m below modern mean sea level [see, e.g., (*102*)], were added from EMODnet Bathymetry data (https://emodnet.ec.europa.eu/en/bathymetry) at 3.75 arc sec (~90-m resolution in central Iberia). Individual bathymetry tiles were joined using r.patch in GRASS GIS and then subsequently reprojected (r.proj) and added (simple sum in r.mapcalc) to produce a final DTM including bathymetric survey data.

In view of the possibility of inaccuracies in the DTM data, and because different least cost analysis approaches are known to give different results (*103*), we used several different methods so that the output could be compared. (i) Following Lewis (*104*), we calculated LCPs from Peña Capón to the outcrops and units in Spain using the algorithm proposed by Llobera and Sluckin (*105*) which is based on an energy expenditure cost function as the walker tries to maintain an optimal slope. The LCP to the much more distant outcrops of Saint-Sulpice-d’Excideuil and Mauprévoir could not be computed using this method, due to the memory intensive nature of the method and its application in R software. (ii) To compute the more distant paths, we used a different cost algorithm implemented inside the computationally more efficient GRASS GIS software, following the approach described by Ullah and Bergin (*106*). First we used the r.walk tool (*107*), which computes anisotropic travel cost in seconds from a point (Peña Capón, in our case) using Naismith’s rule to estimate time cost (*108*) and Dijkstra’s algorithm to calculate least cost directions (*109*), producing a time-cost surface. For comparative purposes, we also computed a time cost surface using Tobler’s hiking function, as recommended by some authors (*103*, *110*). In both cases, we applied the “knight’s move” option in GRASS GIS (*111*), which includes cells outside a given cell’s neighborhood where they are opposite or adjacent to that cell’s immediate Moore neighbors. We extracted travel times for all sites from the time cost surface using the extract function in the R package terra (dataset S4). LCPs were computed using the r.drain function with the time-cost surface and the accompanying distance raster also output from r.walk. Holes in the LCPs resulting from the knight’s move option were filled by vectorizing the LCP rasters to points and joining the points using the QGIS operations points to pixels and points to path. LCPs calculated using the knight’s move option were different from those produced without it. These are not shown here since the knight’s move option is known to be more accurate (*111*).

LCPs for the Spanish outcrops were computed using both the LLobera-Sluckin’s algorithm and the anisotropic Naismith’s rule approach from the GRASS module r.walk, which, in most cases, did not lead to substantial differences. However, LCPs to the outcrops of the Middle Ebro basin (Lanaja-Castejón Unit and Palalluero-Sora Unit) did show substantially different trajectories, depending on whether the Llobera-Sluckin algorithm or the anisotropic Naismith’s rule was used (Fig. 6). Both methods applied are known to be robust and have been shown to produce accurate results in various circumstances (*104*, *106*). Note that in the cases where the geochemical signal did not allow distinguishing between outcrops within a given unit (dataset S2), LCPs were computed to the central point of each geological formation, created from the centroid of a polygon obtained by connecting all sample locations for the relevant unit.

## Supplementary Materials

### The PDF file includes:

Supplementary Text S1 to S7 Figs. S1 to S50 Tables S1 to S9 Provenance Legends for datasets S1 to S6 References

### Other Supplementary Material for this manuscript includes the following:

Datasets S1 to S6
