# Supplementary material for: Far-reaching hunter-gatherer networks during the Last Glacial Maximum in Western Europe
Source: Sci Adv. 2026 Jan 21;12(4):eadz7697. doi: 10.1126/sciadv.adz7697 (PMC12822654; doi:10.1126/sciadv.adz7697)
Supplement: Supplementary file 1 — Supplementary Text S1 to S7 Figs. S1 to S50 Tables S1 to S9 Provenance Legends for datasets S1 to S6 References [file sciadv.adz7697_sm.pdf]

Science Advances
AAAS

Supplementary Materials for

Far-reaching hunter-gatherer networks during the Last Glacial Maximum in Western Europe

Marta Sánchez de la Torre *et al.*

Corresponding author: Marta Sánchez de la Torre, martasanchezdelatorre@ub.edu;
Manuel Alcaraz-Castaño, manuel.alcaraz@uah.es

Sci. Adv. **12**, eadz7697 (2026)
DOI: 10.1126/sciadv.adz7697

The PDF file includes:

Supplementary Text S1 to S7
Figs. S1 to S50
Tables S1 to S9
Provenance
Legends for datasets S1 to S6
References

Other Supplementary Material for this manuscript includes the following:

Datasets S1 to S6

Supplementary Text S1. The Peña Capón rock shelter

1.1. Geological and geographic setting

The Peña Capón rock shelter (Guadalajara province, Spain) is located near the left bank of the Sorbe River, which flows into the Henares, tributary of the Tagus, the main Iberian watercourse, crossing the Spanish Southern Meseta from E to W (Figs. S1, S2). The Sorbe has its source in the highest part of Sierra de Pela, a mountain range located in the eastern limit of the Central System Range at 1,500 m above mean sea level (amsl). The river runs southwards, first crossing gentle Paleozoic reliefs (schist, quartzite and slate) and marine Mesozoic carbonates before meeting the alluvial terrains of the Tertiary sediment infill of the Tagus basin and finally joining the Henares River at a height of 710 m amsl. The archaeological site is located at an altitude of 826 m amsl and 11 to 13.5 m above the current riverbed, under a dolostone rock cliff, in a narrow section of the Sorbe valley before it widens and the Quaternary fluvial and alluvial deposits become more frequent (Figs. S2, S3). The Quaternary deposits are described in the geological maps of the region (*112, 113*) and mainly consist of fluvial terraces, alluvial cones and slope deposits (Fig. S3.A). Based on these maps, our geomorphological analysis showed the existence of fourteen Quaternary fluvial terraces, from +6 to +180-190 m above the current riverbed (*59*). Those below +20 m are generally considered Upper Pleistocene and Holocene in other nearby areas of the Tagus basin and Duero basins (*114, 115*).

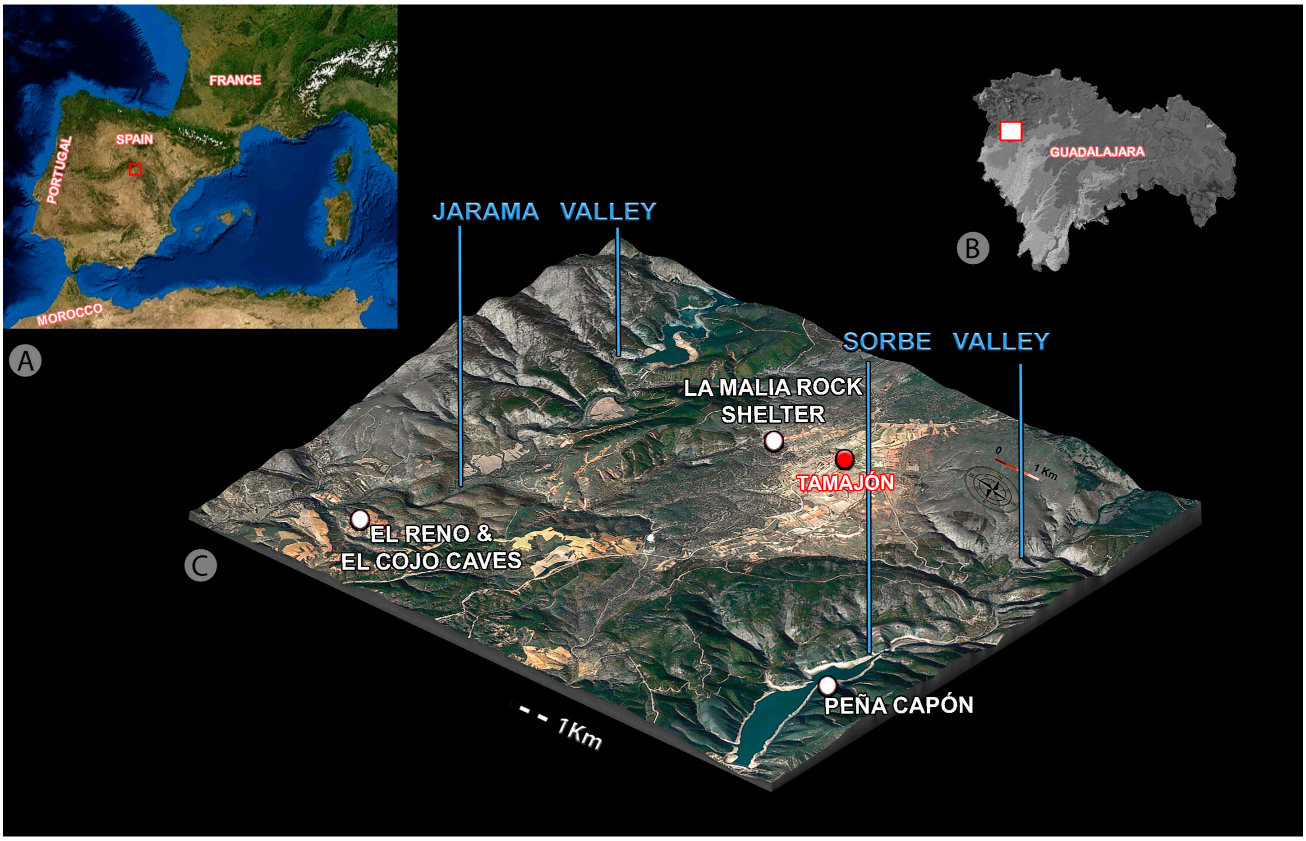

Figure S1. Location of the study area in the Iberian Peninsula and Southwest Europe (A), the Guadalajara province (B) and the upper parts of the Sorbe and Jarama River valleys, showing other Upper Palaeolithic sites (C).

The Peña Capón site is located under an east–west oriented, 42 m high rock cliff, formed by Upper Cretaceous marine dolostone layers dipping to the south. The dolostone outcrops as part of a long hogback relief oriented to the NW–SE that surrounds the Paleozoic shales, schists and quartzites, as well as the Lower Triassic Buntsandstein facies, located to the north and west. The

archaeological site is located 80 m away from the current riverbed, close to a narrowing of the valley excavated in the dolostone relief. Due to its location, the site is flooded by the Beleña reservoir waters for most of the year since a dam was constructed in 1982 (Figs. S1, S4).

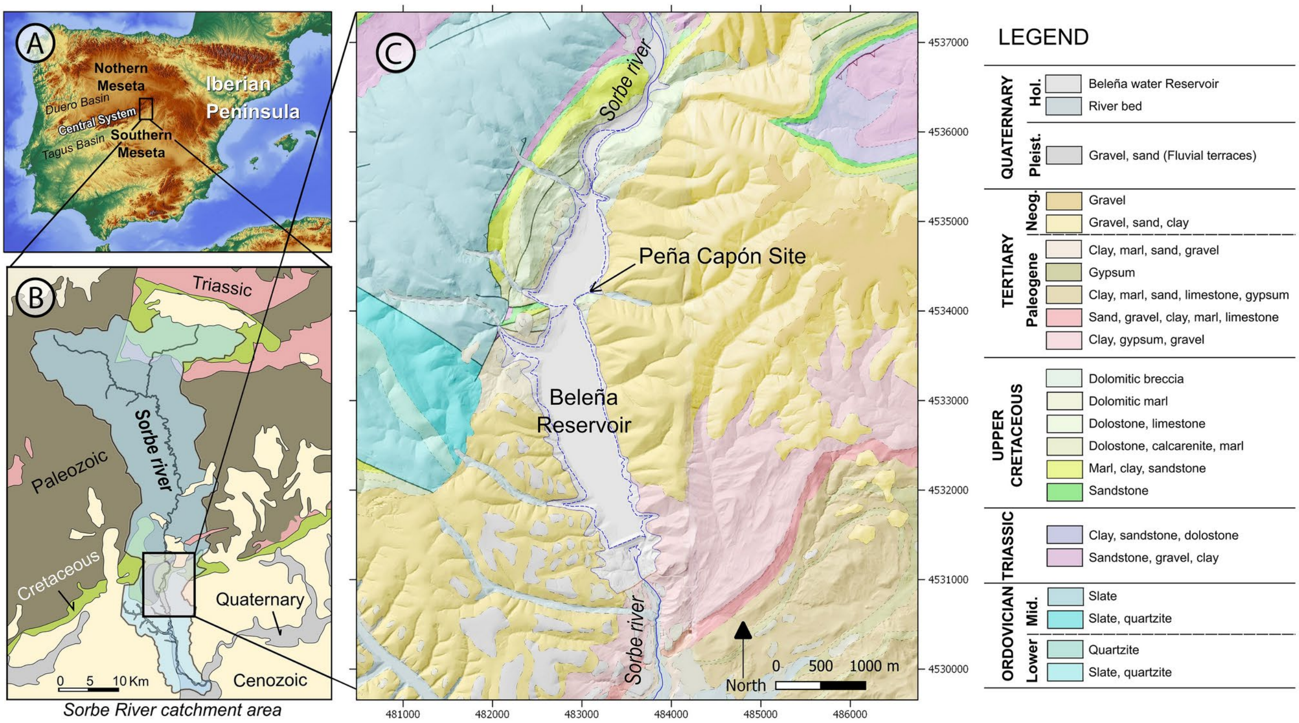

Figure S2. Geological maps showing the location of the Peña Capón rock shelter in the Iberian Peninsula and the Tagus basin (Guadalajara, Spain) (A), the Sorbe River basin (B) and at the shore of the Beleña water reservoir (C). Maps generated using QGIS Open Source Geographic Information System v. 3.4 (Madeira) combined with Digital Terrain Models and slope maps from the Spanish National Centre for Geographic Information (CNIG) and geological maps from the Spanish Geological Survey (IGME).

1.2. Sedimentary deposit, excavation and stratigraphy

The Peña Capon archaeological site covers an area of 30 m long and 5 to 8 m wide (about 150 sq m in area) along the foot of the dolostone rock wall (Figs. S3-S6). The sedimentary deposit consists of poorly sorted, relatively homogeneous sands mainly composed of quartz and feldspar, with an abundant silty-clayey matrix (from 25 %to 55%). Interspersed within this deposit there are angular dolomite blocks fallen from the rock shelter walls. The entire deposit contains archaeological material (mostly lithic industry, charcoal and altered bones) to varying degrees. The lithic assemblages subject to textural, micropalaeontological and geochemical analyses mostly come from the excavation conducted in a central area of the site, during the 2015, 2019, 2021 and 2022 seasons. This area, dubbed 'Central Pit', include the excavation of 13 square meters, to which a 2-sq meter 'Geotrench' was added in the 2021 season (Figs. S6-S9). The maximum thickness of the *in situ* archaeological deposit in the Central Pit is 2.10 m, on top of which a ~0.65 m-deposit of mostly reworked sediments, disturbed in historical times due to the use of the shelter as a livestock refuge, was recorded. This upper part of the sedimentary sequence (Level R, subdivided in R01 and R02) has a darker grey coloration, whereas the lower section exhibits a typically yellowish to orange hue (Fig. S7-S9). Excavation of sediments followed standard methods in Palaeolithic Archaeology and their details, as well as methods for spatial recording and sampling for geoarchaeological, palaeoenvironmental and chronometric analyses can be found in Alcaraz-Castaño *et al.* (*59*).

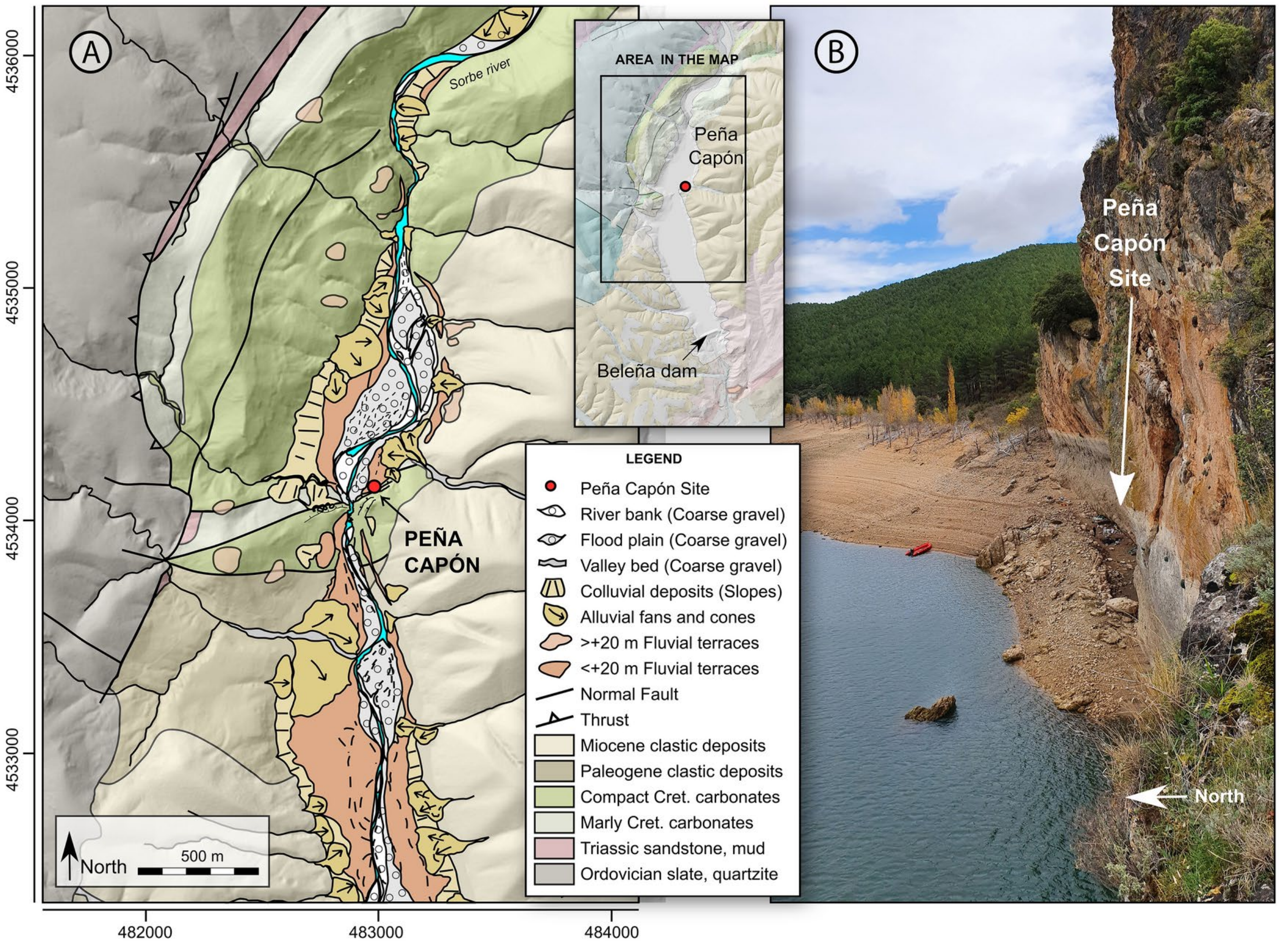

Figure S3. (**A**) Geomorphological map of the study area showing the position of Peña Capon at the foot of a dolomite cretaceous relief, and the distribution of the Quaternary deposits located in the area. (**B**) General view of the site from above. Map generated as explained in Fig. S2. Photo credit (B): Luis Luque.

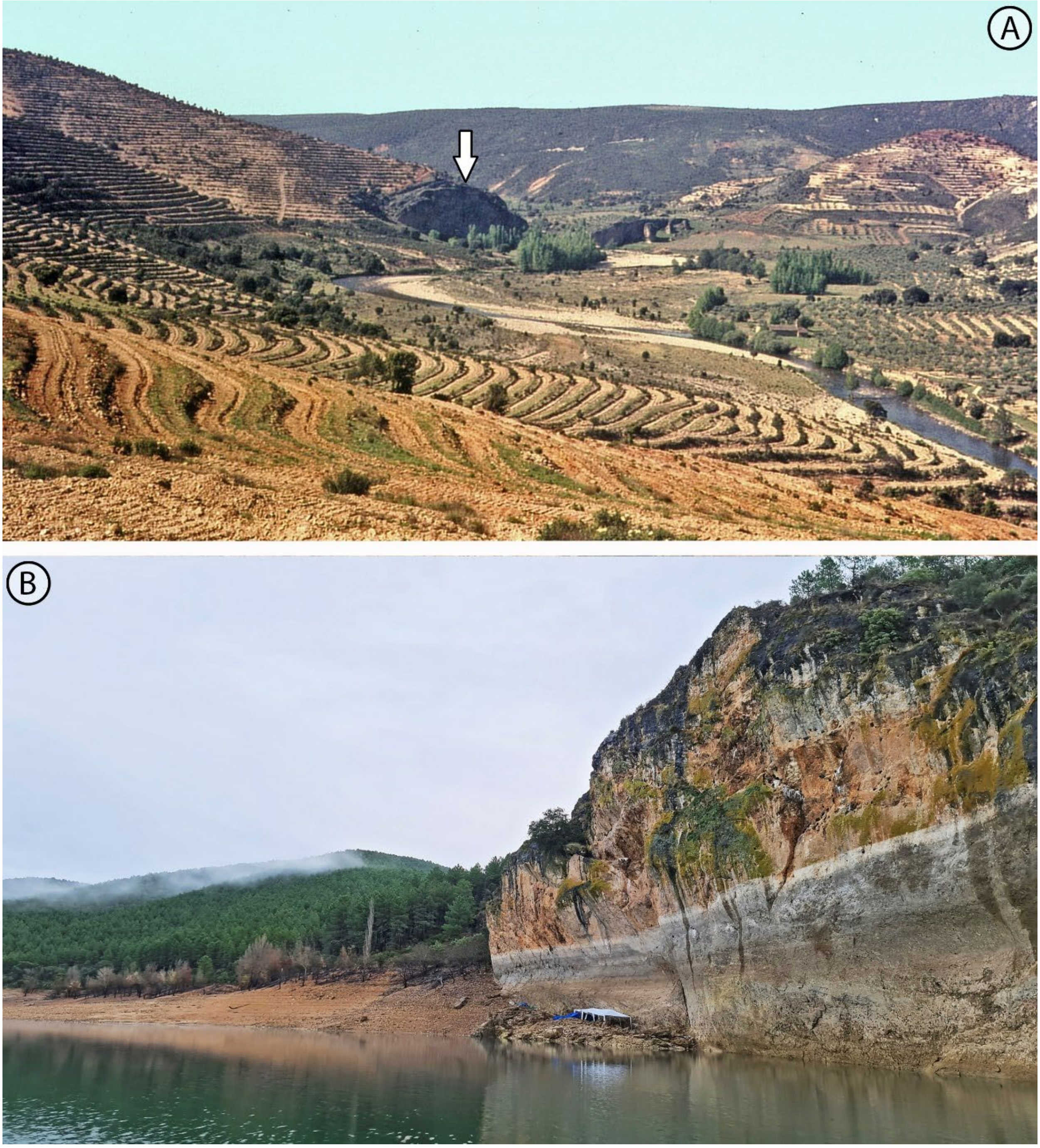

Figure S4. General views of the Peña Capón rock shelter (**A**) View from the northeast in 1980, before the construction of the dam. (**B**) View from the northwest in November 2022. Photo credits: Antonio Alcaina (A) and Guillermo Jiménez-Gisbert (B).

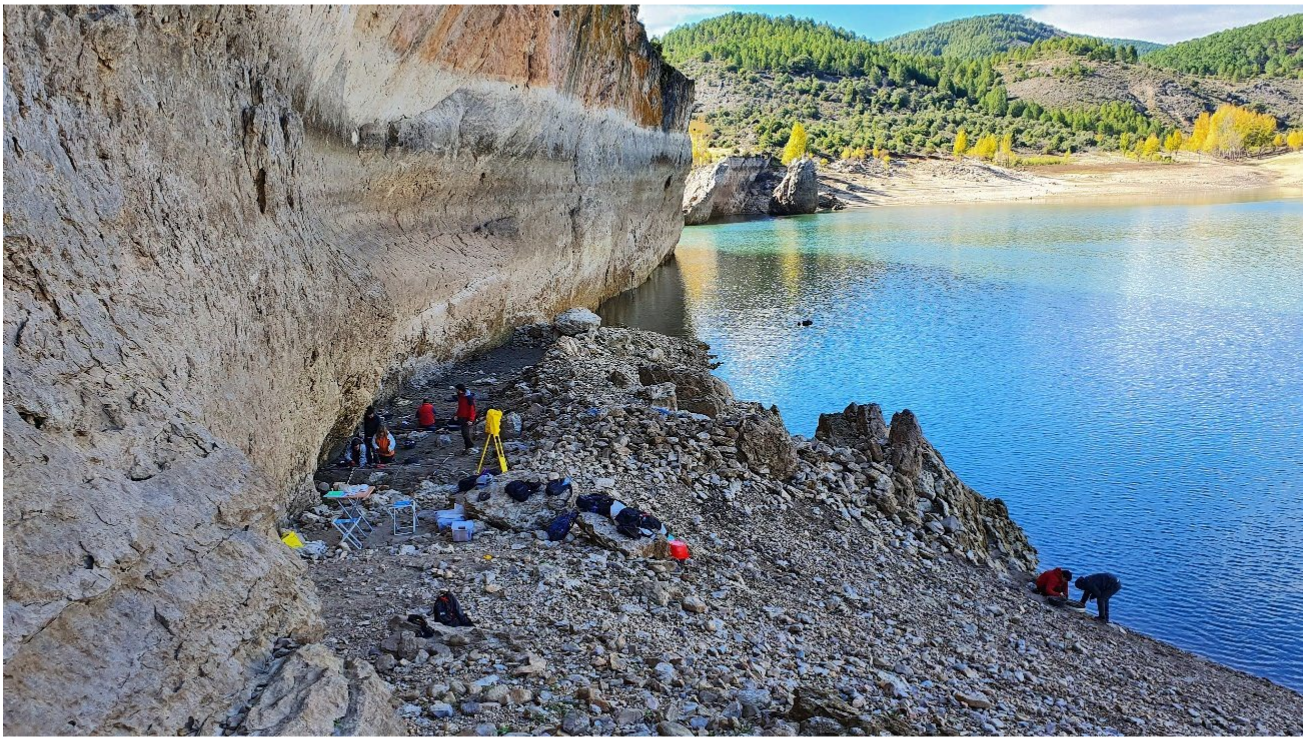

Figure S5. View of the Peña Capón sedimentary deposit from the east, during the 2019 archaeological season. Photo credit: Luis Luque.

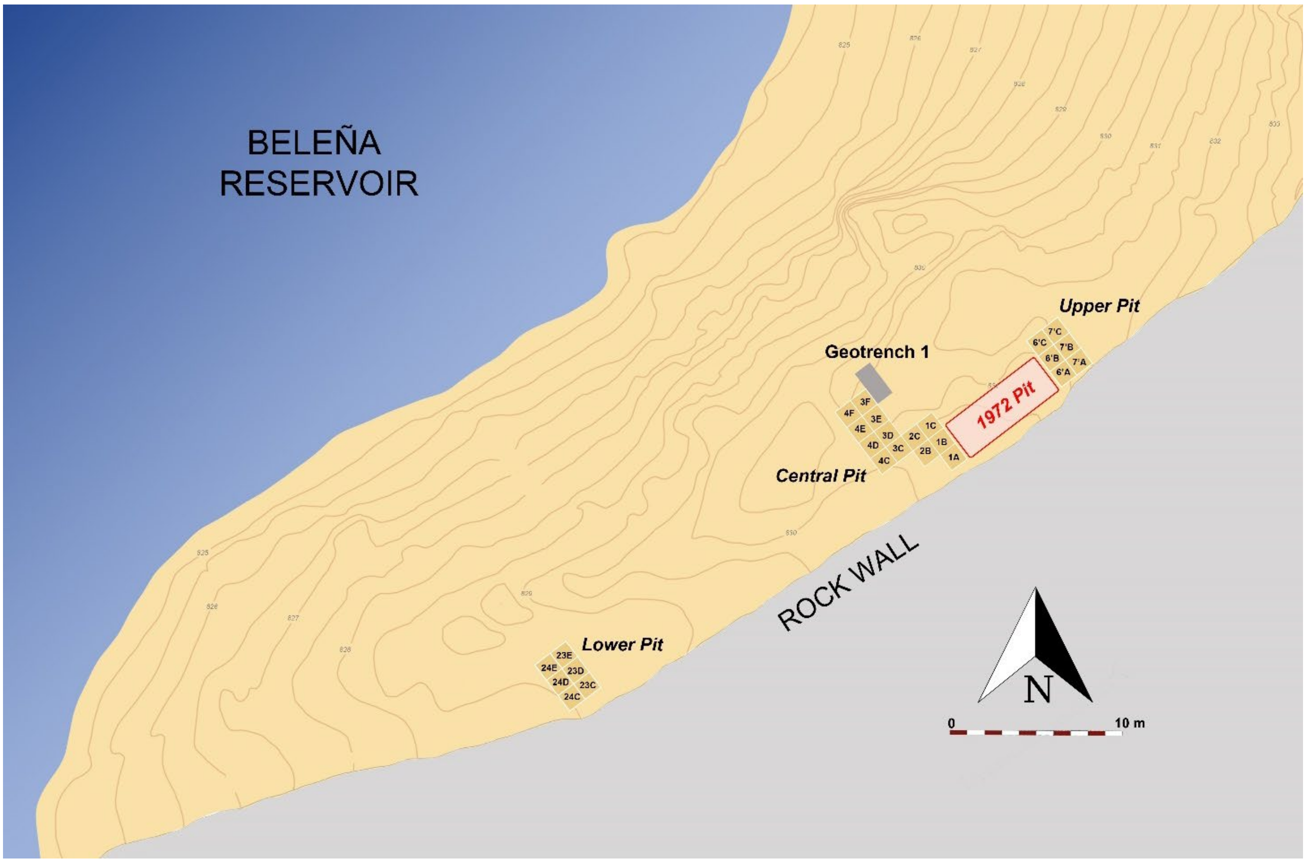

Figure S6. Topographic plan of the Peña Capón sedimentary deposit and the archaeological test pits conducted to date.

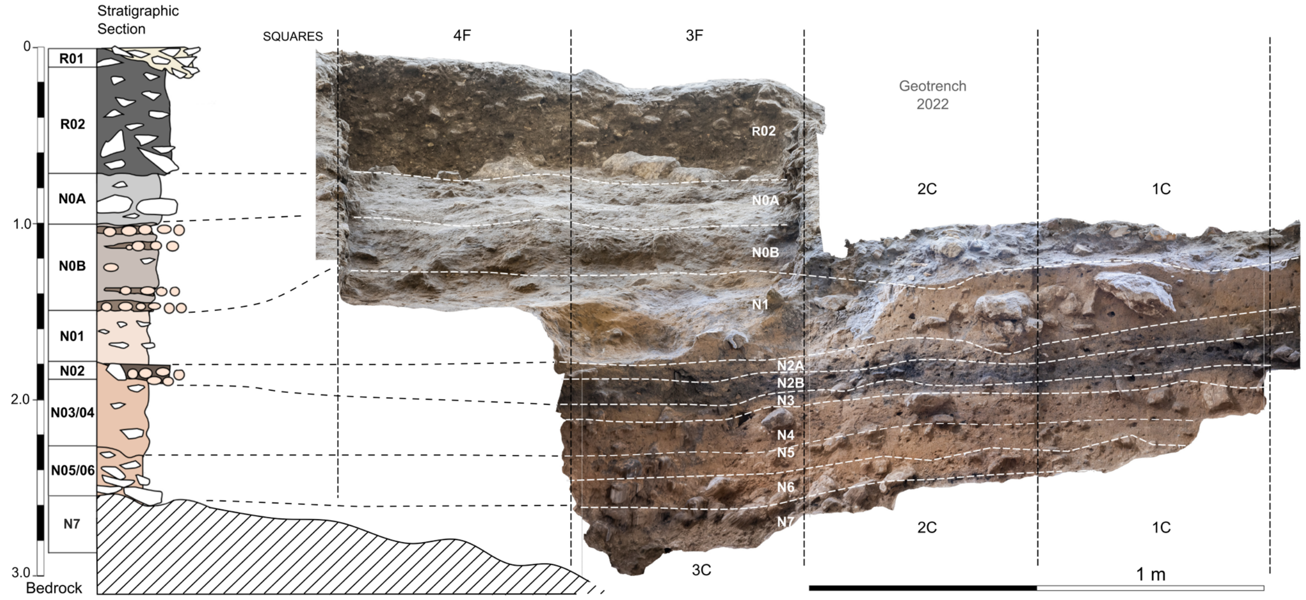

Figure S7. Stratigraphic sequence defined in Geotrench 1and correlation with the levels recorded in the Central Pit at Peña Capón.

The stratigraphic sequence, as defined in the *Central Pit* and Geotrench 1 (Fig. S6) is composed of eight different sedimentological units containing archaeological remains (Levels 0 to 7) (Figs. S7-S9). They are primarily distinguished by colour (influenced by carbon and secondary carbonate content), grain size, block abundance, and erosional surfaces between them. These levels correlate with the geotrenches and test pits conducted at other points of the site. The deposit is sufficiently homogeneous to be considered of a single origin, linked to fluvial deposits from the Sorbe River. The absence of conglomeratic levels or coarse-grained gravels composed of quartzite or slate—characteristic of current and fossil channel and alluvial fan deposits in the area—suggests that the site was formed in a typical floodplain environment. The narrow point of the valley where Peña Capón is located may have led to the formation of a broad flood zone during floodings, facilitating the deposition of clayey sands on the lowest slopes of the valley. Although minor erosional surfaces are present, sedimentation appears to have been relatively continuous over the more than 4,000 years of site formation recorded to date. Bioturbation is present at two scales: large burrows made by mammals and small tubular structures produced by endobenthic invertebrates. The former significantly alters the sedimentary and archaeological record (although in very localised, easily perceived areas), whereas the latter have little impact on it.

Level 0, the uppermost stratigraphic unit recorded to date, has been only registered at the highest area of the deposit, corresponding to squares 4F, 3F, 4E and 3E, as well as in the adjacent Geotrench 1 (Figs. S7 & S9). In Level 0 the abundance of fallen blocks increases, along with a higher concentration of organic matter, suggesting a slower sedimentation rate during the final phases of deposition before the site became fully isolated from river influence. The base of the deposit rest on the dolomitic bedrock or large fallen blocks lying on it, corresponding to Level 7. The bedrock below Levels 6 and 7 has been reached in squares 1B, 2B, 1C, 2C and 3C, as well as in the Geotrench (Fig. S9). Above this, the sediments thin out with a slight slope (4°) from the valley axis towards the upper part of the site. A greater abundance of blocks is observed at the outer section of the site, beneath the overhang of the rock shelter, where verticalized layers of the bedrock also outcrop (Fig. S3). This configuration, along with the high ceiling of the shelter, has acted as a protective barrier, preserving the site from erosion both during its formation and as the Sorbe River progressively entrenched into the valley over the past millennia (*59*).

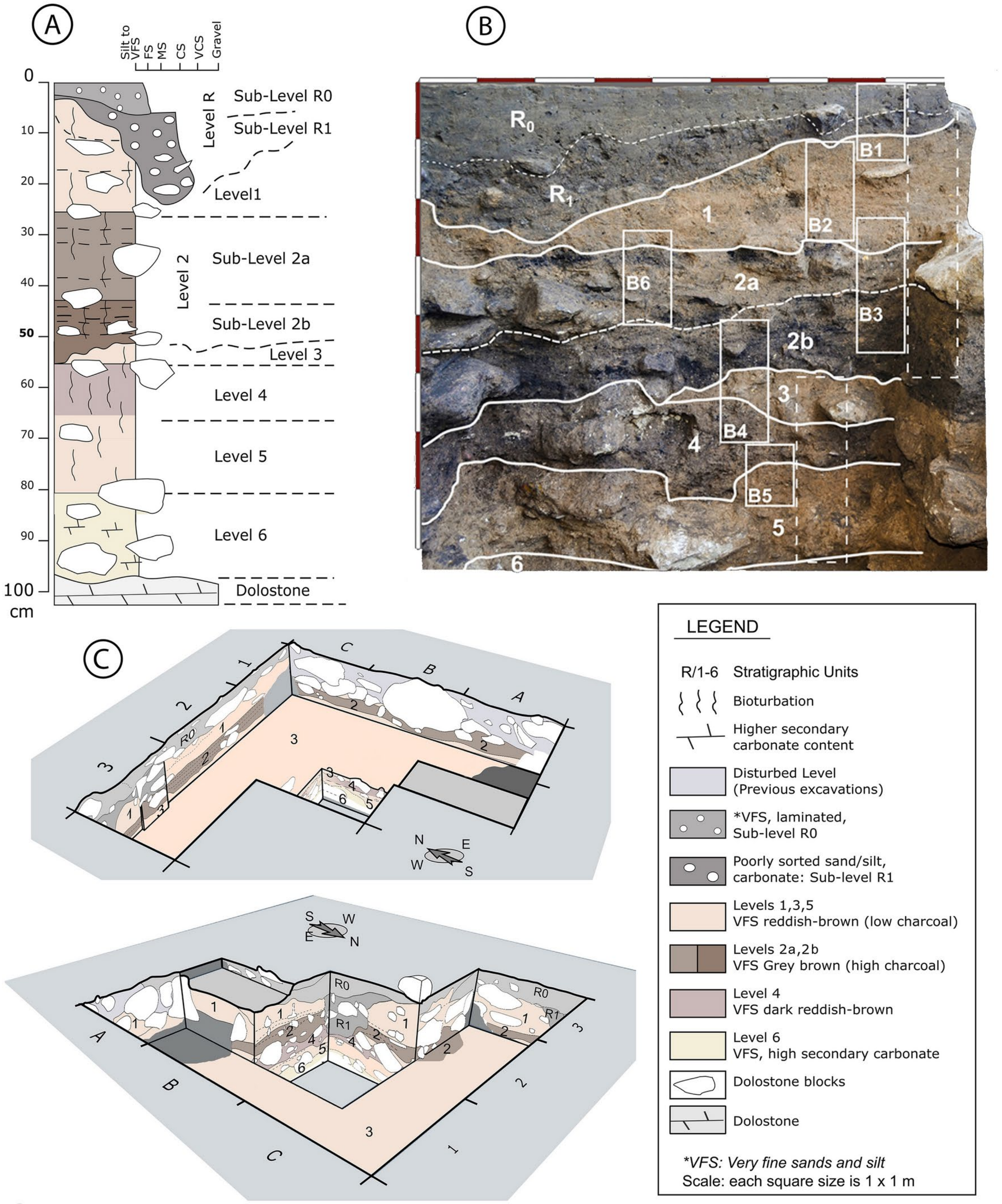

Figure S8. (A) Stratigraphic sequence defined in square 2B of the Central Pit at Peña Capón (after Alcaraz-Castaño *et al*. (*59*). Note that the main differences between levels are due to variations in organic matter and secondary carbonate content. (B) Stratigraphic sequence recorded in the western profile of square 2B showing sample location for micromorphology (B1–B5) and sedimentology (dashed-lines rectangles) [see (*59*)]. (C) Views of the excavation profiles at Peña Capón showing the different distribution and geometry of the stratigraphic units defined in the site during the 2019 season.

The sedimentary characteristics of the Peña Capón stratigraphic sequence area as follows (Figs. S7-S8):

Level 0 (0.8 m): The most recent *in situ* level of the site, mixed with recent soil at the top (Level R). It contains an abundance of angular dolomitic clasts (4–10 cm wide) within a dark gray sandy matrix. The sands include small lenses of quartzite gravel, together with burned bone fragments, charcoal, and lithic artifacts. There is diffuse internal stratification, with sublevels richer in blocks and others that are more distinctly sandy, just like the base of this unit.

Level 1 (0.18-0.55 m): A highly homogeneous level composed of poorly sorted, yellowish-coloured clayey sands with some charcoal fragments. It has an abundant matrix (>60% clay and silt) and a massive appearance, although up to three sublevels with slightly different textures can be distinguished in some profiles. The lower contact is gradual.

Level 2a (0.08–0.12 m): A fairly continuous level characterized by yellowish sandy clays interbedded with thin, darker lenses due to millimetre-sized charcoal fragments. It also contains dolomitic rock blocks. Lithic artifacts and bone fragments are abundant in this level. The lower contact is gradual.

Level 2b (0.06–0.1 m): A highly distinctive clayey sand black-coloured level with a high abundance of charcoal fragments. Its contact with the underlying level is sharp and highly irregular, with disturbances possibly linked to anthropogenic activities. Overall, it presents an erosive base over Levels 3 and 4.

Level 3 (0–0.05 m): Very homogeneous yellowish to orange clayey sands, laterally wedging out due to erosion. Contains angular dolomitic blocks, bone fragments, lithic artifacts, and secondary calcite partially filling the porosity. Its lower contact is gradual with the underlying level.

Level 4 (0.04–0.17 m): A homogeneous stratigraphic level with a reddish-yellow sandy clays transitioning to orange-grey or brown coloration due to rubefaction processes and with abundant organic matter. The overall appearance is massive, with few angular dolomitic blocks in it. Contains small charcoal fragments and abundant bioturbation. The lower contact is gradual or locally sharp.

Level 5 (0.12–0.27 m): Yellowish to orange-coloured sandy clays with dispersed carbonaceous organic matter, including large dolomitic rock blocks. It has a slightly lighter tone than other levels due to a higher amount of diffuse carbonate in the sediment and is somewhat more compacted or cemented. The contact with the underlying level is transitional and sometimes difficult to define, as it is associated with an increase in secondary carbonate content.

Level 6 (0.05–0.2 m): A level of light yellowish clays, highly carbonated and cemented due to the influence of the dolomitic substrate (intense lixiviation processes), with large fallen blocks in contact beneath it. It wedges out towards the north. The base of the level contains large bone fragments among dolomitic rock blocks and locally a higher concentration of charcoal, giving it a darker colour.

Level 7 (0.24–0.27 m): The oldest level excavated to date at Peña Capón, locally overlying the bedrock. It consists of sandy sediments, somewhat more silty-clayey than the overlying levels, fairly homogeneous, and light gray in color (with higher charcoal and carbonate content), containing abundant large dolomitic blocks (0.2–0.3m in size). This level continues to yield archaeological remains and bone fragments.

1.3. History of research at Peña Capón and the 1972 excavation

Peña Capón was discovered in 1970 and first excavated in 1972 during two field seasons. Fieldwork was conducted by a team led by J. Villasenín-Gómez and directed by J. Martínez Santa-Olalla (*116-119*). However, results of these excavations were never published, and it was only in the late 1990s that the archaeological assemblages, diaries, and photos from the field seasons were collected and analysed in a preliminary paper on the site by Alcolea-González *et al.* (*119*). Since this first publication, in which a preliminary archaeological sequence was presented, the relevance of Peña Capón was evident: in no other location on the whole Iberian plateau had Solutrean and pre-

Solutrean assemblages been described within a multi-layered sequence (*120*). However, those assemblages, including lithic and faunal remains, were recovered in a poorly recorded excavation, lacking any stratigraphic, chronometric or paleoenvironmental data besides a few photographs and oral records (*119*).

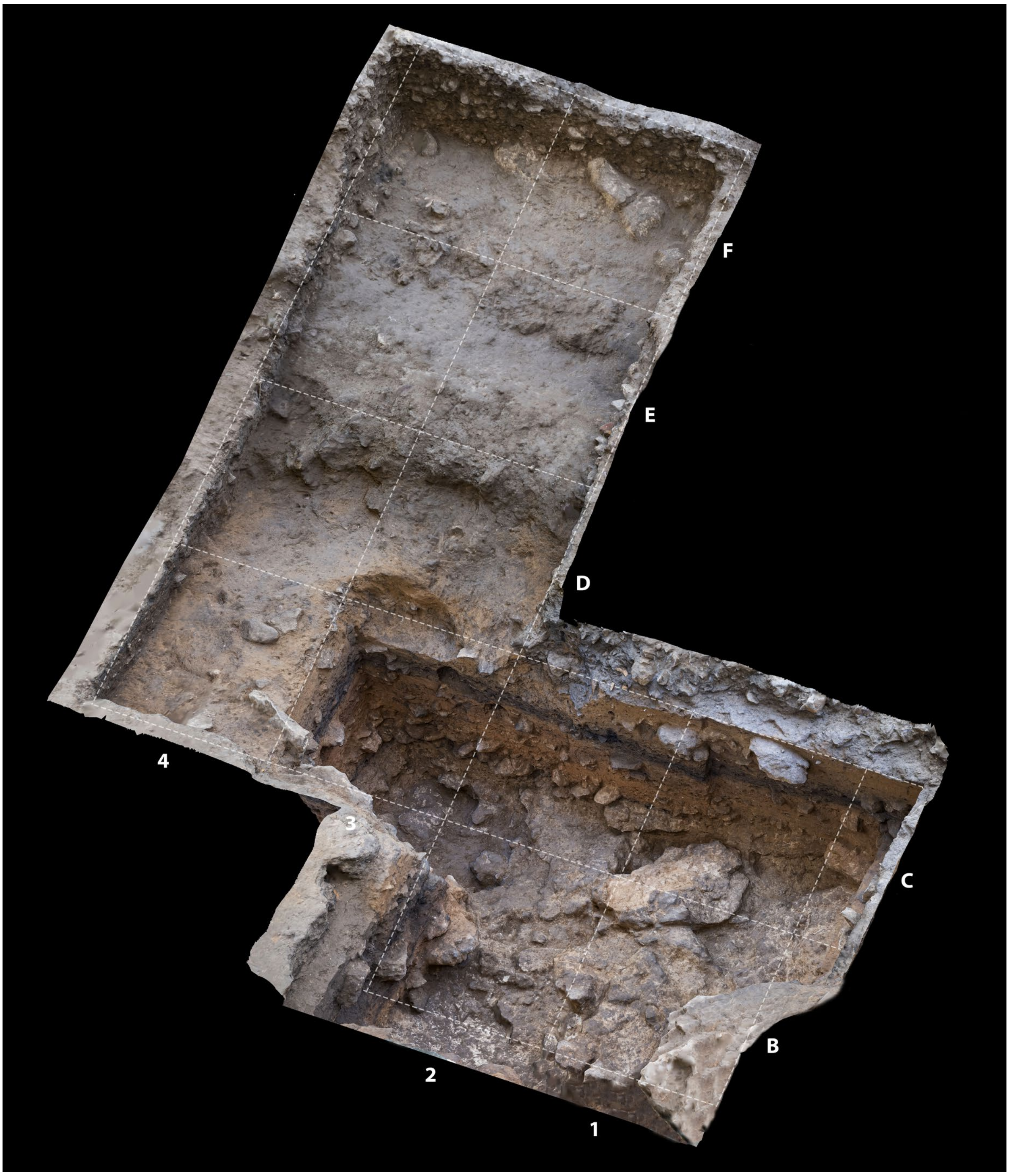

Figure S9. Photogrammetric model of the ‘Central Pit’ excavated at the end of the 2020 field season. Squares 1B, 2B, 1C, 2C and 3C shows the base of Level 7 in contact with the dolostone boulders of the bedrock. Squares 3E, 4E, 3F and 4F shows Level 0 during the excavation process (layer 14). Squares 4C, 3D and 4D shows the cap of Level 1, after the cleaning of the superficial Level R. Photo credits: Javier Alcolea-González.

The first attempt at solving the methodological inconsistencies and the lack of sound data at Peña Capón, was carried out by Alcaraz-Castaño *et al.* (*116*). Up to that point, excavation of the site

had not been possible due to the difficulties of accessing the archaeological deposits, which are often under water due to the construction of a dam in 1982 [Figs. S1 – S6 and see (59)]. These scholars therefore limited their work to an in-depth study of the lithic and faunal assemblages and the radiocarbon dating of a single tooth per level, as preliminarily defined according to the data of the 1972 fieldwork (Dates from Beta in Table S1). This confirmed that an occupation sequence around the time of the LGM was indeed present at Peña Capón, and that this included at least Upper Solutrean (Level I), Middle Solutrean (Level II) and Proto-Solutrean assemblages (Level III), as well as a potential Gravettian component (Level IV). While zooarchaeological and taphonomic studies have been conducted for levels II and III, including stable isotope analyses on the teeth of herbivores (*116, 121*), analyses of lithic assemblages have been thoroughly published only for the Proto-Solutrean level (III) (*116, 119*). However, the assignment of each level to a given technocomplex is sound, as is based on the presence of willow leaf, laurel leaf and shouldered points (including both 'Mediterranean' and 'Cantabrian' types) in Level 0, laurel leaf points in Level II, and Vale Comprido points in Level III (Fig. S10). For Level IV, given the limited amount of lithics and the absence of typologically informative elements, only a tentative 'Gravettian' assignment has been possible to date (*59, 116, 120*) (but see below). Bone tools, including antler points, are found in all levels throughout the sequence.

Level	Sample ID & material	Lab-ID	C^{14} BP	δ13C (‰)	Age cal BP (95,4%)
I. Upper & Middle Solutrean (Reworked)	PCP-I.B1 Bone	OxA-44243	19820 ± 140	-19.5	24220 - 23395
	PCP-I.B7 Bone	OxA-44244	20830 ± 140	-19.5	25575 - 24695
	PCP-I.B12 Bone	P-54257	Failed: Low yield	-	-
II. Middle Solutrean	PCP Bone 16-5	COL4223.1.1	20204 ± 108	-15.2	24270 - 23812
	Tooth B-5	Beta - 246880	19930 ± 110	-20.2	24213 - 23780
III. Proto-Solutrean	PCP Bone 16-6	COL4224.1.1	20863 ± 116	-15.3	25596 - 24853
	Tooth B-4	Beta - 246879	19980 ± 110	-20.4	24239 - 23800
IV. Terminal Gravettian	PCP Bone 16-7	COL4225.1.1	21647 ± 123	-17,8	26258 - 25726
	Tooth B-3	Beta - 246878	21220 ± 120	-20.5	25819 - 25251

Table S1. Sampled materials and radiocarbon dates from the Peña Capón sequence as defined in the 1972 excavation. All bone samples were cut-marked. C14 dates were calibrated with OxCal 4.4 (*211*) using IntCal20 (*212*). Dates from Beta were first published in (*116*), and dates from CologneAMS were published in (*59*).

A rough correspondence between the stratigraphic sequence defined after the 1972 excavations and the one recorded during the modern fieldworks has always been clear, as both excavations are contiguous (Fig S4) and the broad archaeological sequence in each of them (Upper Solutrean – Middle Solutrean – Proto-Solutrean – Gravettian) is the same. However, in previous works we decided to keep separate the archaeological assemblages from each excavation as a cautionary measure (*59, 117*). Yet, new stratigraphic, radiocarbon and archaeological data now allows for a precise correlation between the archaeological levels defined in the old and new excavations (Table S2). The available radiocarbon determinations for Level II, Level III and Level IV of the 1972 sequence virtually mirror those obtained for the Middle Solutrean (Levels 1-3), Proto-Solutrean (Level 4) and Terminal Gravettian (Levels 5 & 6) of the new excavations (*Central Pit*) respectively (Tables S1 and S3). The case of Level I is different, as it has always been clear that it corresponds to several reworked stratigraphic units –something that went unnoticed in 1972. This was already indicated by the heterogenous nature of its lithic assemblages (*59, 116, 117*), and is now reinforced by two new radiocarbon results on anthropogenically-modified bone samples which are closer to the time range of the Middle Solutrean or Proto-Solutrean occupations (Levels 1 to 4) (Table S1) than to the chronology established for Level 0 of the new excavations (Table S3). Therefore, most of the archaeological items from Level I have not been considered for this or any other study, as their

stratigraphic provenience is unknown. However, in the case of the shouldered points, their significant value as index fossils of the Upper Solutrean enable us to assign them to the same archaeological context as the assemblages from Level 0 (Table S2). As no shouldered points have ever been reported in Middle Solutrean layers besides a couple of problematic examples (*31, 64, 88, 122-126*) [but see (*127*) for a critical view] and in the modern excavations at Peña Capón they have been only found in Level 0 of the *Central Pit* (with not a single hint of them in levels below), the assignment of these artefacts to Level 0 is strongly justified.

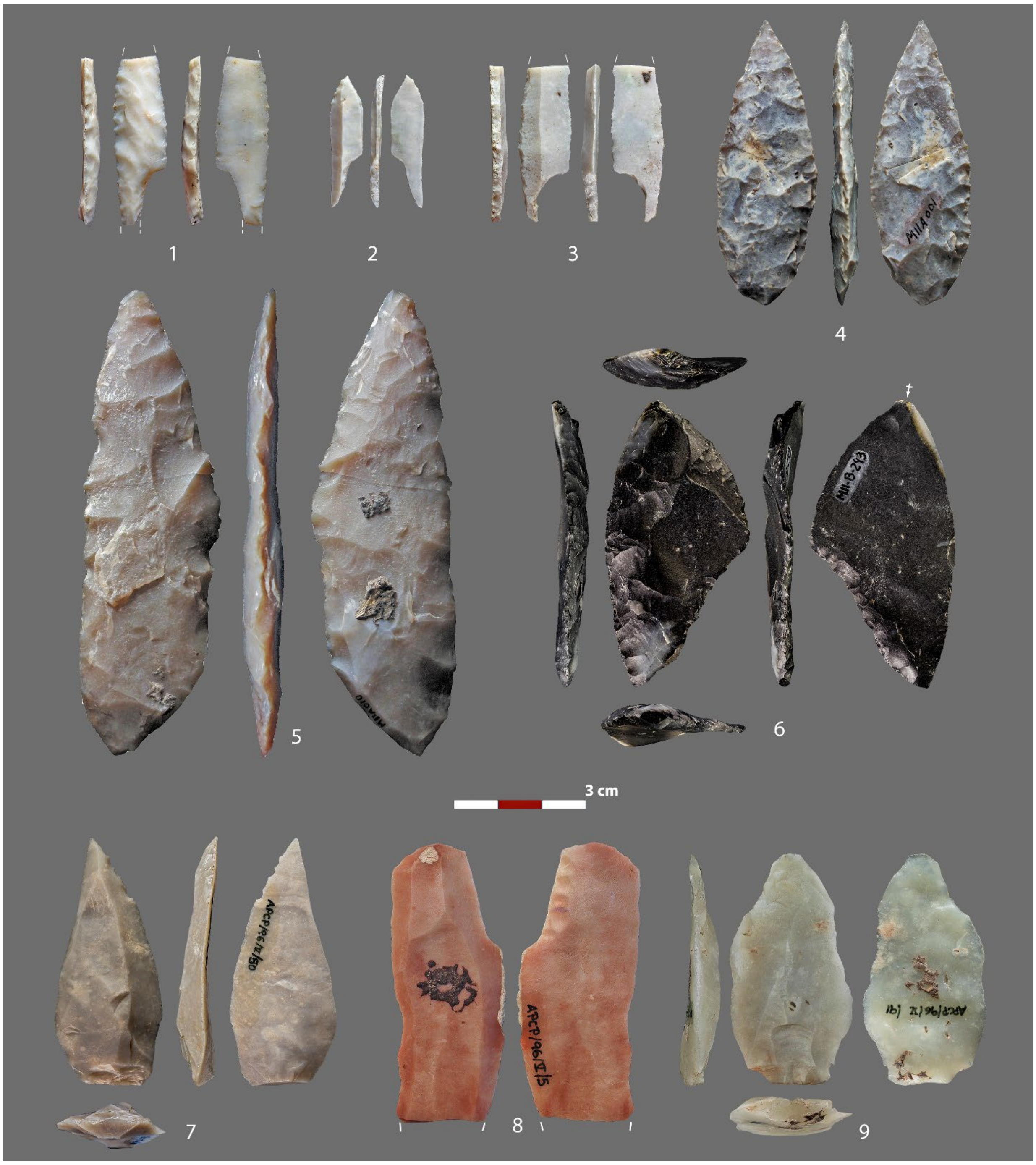

Figure S10. Lithic assemblages from levels I, II and III of Peña Capón as defined in the 1972 excavation. 1: Cantabrian-type shouldered point. 2-3: Mediterranean-type shouldered points (Level I). 4: Laurel leaf with convex base. 5: Large laurel leaf. 6: Montaut point (level II). 7 & 9: Vale Comprido points. 8: Endscraper on a blade with inverse flat retouch (Level III). Photo credits: Manuel Alcaraz-Castaño.

1972 Excavation		Central Pit	Techno-cultural assignment	Age cal BP (95,4%)
Level I (Only shouldered points)	--->	Level 0	Upper Solutrean	23854 - 22178
Level II	--->	Levels 1, 2a, 2b & 3	Middle Solutrean	25264 - 22882
Level III	--->	Level 4	Proto-Solutrean	25402 - 25064
Level IV	--->	Levels 5 & 6	Terminal Gravettian	26172 - 25190

Table S2. Correlation between levels from the 1972 excavation and those defined in the stratigraphic sequence recorded in the Central Pit of modern fieldworks. Ages refer to calendar ages estimations as obtained through Bayesian modelling of radiocarbon dates from the modern excavation (see Text S1.4.).

1.4. Chronometric sequence: radiocarbon dating and Bayesian modelling

The chronological setting of the Peña Capón sequence available to date is based on the radiocarbon dating of selected samples of faunal remains with anthropogenic modifications (mostly cut marks) (n = 23) and charcoal fragments (n = 12) recovered from secure stratigraphic contexts and covering most of the sequence excavated thus far (Levels 0 to 6). These samples were first identified to taxon (when possible) and then sent to two different laboratories for cross-checking results: 15 samples were sent to the CologneAMS centre at the University of Cologne (Germany) and 20 samples to the Oxford Radiocarbon Accelerator Unit (ORAU) at the University of Oxford (United Kingdom). The results have enabled us to build a chronological sequence based on 24 AMS reliable determinations, most of which were already published in Alcaraz-Castaño *et al* (*59*). However, two samples from Level 0, the uppermost level, not covered until the 2021 season, have been added and first published in the present study. A detailed description of the methodology used for Bayesian modelling can be found in Alcaraz-Castaño *et al*. (*59*). Here we present a list of the dated samples and the radiocarbon results (Table S3), the detailed results of the Bayesian model (Table S4) and the model outcome showing the Probability Distribution Functions (Figure S11). The OxCal CQL code is also provided (Code S1).

The model results place the start boundary of the sequence radiocarbon dated thus far (base of level 6) between 26.8 and 25.8 ka cal BP at 95.4% probability, and the end boundary (top of the known sequence at Level 0) at 22.8–21.0 ka cal BP. However, when considering the results of applying the 'date' command in OxCal, the Probability Distribution Function (PDF) for the time span of human occupation at the site is constrained between 26.2 and 22.3 ka cal BP. The calendar age estimates for each level are shown in Table S5 and their associated PDFs are provided in Fig. S12 (complete data and boundaries between levels are shown in Table S4).

Level	Sample ID & material	Lab-ID	C^{14} BP	δ13C (‰)	Age cal BP (95,4%)	% C	Collagen yield (%)	Taxa
0	PCP-A.1-TR1 Bone	OxA-42423	18608 ± 87	-19.5	22859 - 22352	45.1	3.5	-
(Upper Solutrean)	PCP-A.1-TR2 Bone	OxA-42424	18602 ± 83	-19.2	22845 - 22348	42.6	2.2	-
1	PCP Char 16-1	COL4210.1.1	20008 ± 112	-24.8	24371 - 23770	-	-	Angiosperm indet.
(Middle Solutrean)	PCP Bone 1/5	OxA-39498	19928 ± 97	-20.1	24195 - 23786	43.3	9.5	-
	PCP Bone 1/7	OxA-39505	19950 ± 110	-19.7	24220 - 23790	42.9	1	-
	PCP Char 16-2	COL4211.1.1	Modern	-25.4	-	-	-	Salix sp.
2a	PCP Bone 16-1	COL4214.1.1	19987 ± 110	-17.9	24240 - 23804	41,6	5.0	-
(Middle Solutrean)	PCP Char 16-4	COL4213.1.1	20107 ± 111	-24.6	24468 - 23843			Angiosperm indet.
	PCP Bone 16-2	COL4215.1.1	20261 ± 111	-21.8	24675 - 23970	41,7	14.0	-
	PCP Bone 2/3	OxA-39499	20370 ± 110	-19.19	24871 - 24175	43.5	5.47	
	PCP Char 16-3	COL4212.1.1	20278 ± 107	-30.4	24700 - 24012	-		Angiosperm indet.
2b	PCP Bone 2/5	OxA-X-3048-17	20308 ± 150	-19.19	24903 - 23974	43.7	0.5	-
(Middle Solutrean)	PCP Char 2/5	OxA-39506	20399 ± 63	-24.82	24747 - 24225	69	19.3	-
	PCP Bone 2/7	OxA-39500	20450 ± 110	-20.19	24956 - 24239	43.3	1.1	-
3	PCP Bone 16-3	COL4217.1.1	20006 ± 107	-18.1	24252 - 23811	36,9	1.0	-
(Middle Solutrean)	PCP Char 16-5	COL4216.1.1	20950 ± 128	-22.7	25655 - 24980	-	-	Angiosperm indet.
	PCP Bone 3/5	OxA-39501	20910 ± 110	-19.59	25608 - 24963	44.8	5.9	-
4	PCP Bone 4/Rect	OxA-39502	20930 ± 110	-19.47	25618 - 24991	44.4	4.3	
(Proto-Solutrean)	PCP Bone 4/Rect3B	OxA-39750	20760 ± 110	-19.40	25283 - 24664	43.9	1.8	
	PCP Char 16-6	COL4218.1.1	21007 ± 118	-19.6	25671 - 25079			Indet.
	PCP Bone 16.10-1	COL-	Failed: Low yield	-	-	-	-	-
5	PCP Char 16-7	COL4219.1.1	20905 ± 118	-27.8	25623 - 24937	-	-	Angiosperm indet.
(Terminal Gravettian)	PCP Bone 5/Rect2A	OxA-X-3058-10	20950 ± 180	-18.93	25730 - 24794	43.7	0.4	-
	PCP Bone 5/Rect3B	OxA-39749	21670 ± 130	-19.29	26280 - 25745	43.5	3.9	-
	PCP Bone 16-4	COL4220	Failed: no yield	-	-	-	-	-
	PCP Bone 16.10-2	COL-	Failed: no yield	-	-	-	-	-
	PCP Bone 5/4	P-48038	Failed: very low yield	-	-	-	-	-
	PCP Bone 5/4/1	P-48039	Failed: very low yield	-	-	-	-	-
6	PCP Char 16-8	COL4221.1.1	21591 ± 124	-27.5	26069 - 25669	-	-	Angiosperm indet.
(Terminal Gravettian)	PCP Char 16-9	COL4222.1.1	21593 ± 121	-24.8	26065 - 25675	-	-	Conifer
	PCP Bone 6/1	P-48041	Failed: very low yield	-	-			-
	PCP Bone 6/2	P-49063	Failed: no yield	-	-	-	-	-
	PCP Bone 6/BN	P-49064	Failed: no yield	-	-	-	-	-
	PCP2 Char 6/1	P-49065	Failed: very low yield	-	-	-	-	-
	PCP2 Char 6/3	P-49066	Failed: very low yield	-	-	-	-	-

Table S3. Sampled materials and radiocarbon dates from the Peña Capón sequence. C^{14} dates were calibrated with OxCal 4.4 (*211*) using IntCal20 (*212*). OxA-, OxA-X- and P- are ORAU refs, the last corresponding to failed samples. COL- are CologneAMS refs. Some charcoal fragments could not be identified. All bones were cut-marked shaft fragments of unidentifiable macromammals, most probably herbivores. Failed attempts are shown in red.

Code S1. CQL Codes for the Bayesian analysis – Peña Capón sequence

```
Options()
 {
  Resolution=20;
 };
 Plot()
 {
  Outlier_Model("General",T(5),U(0,4),"t");
  Sequence("Peña Capón")
  {
   Boundary("Start");
   Phase("Level 6")
   {
    R_Date("COL4222.1.1", 21593, 121)
    {
     Outlier(0.05);
    };
    R_Date("COL4221.1.1", 21591, 124)
    {
     Outlier(0.05);
    };
   };
   Boundary("Level 6/Level 5");
   Phase("Level 5")
   {
    R_Date("OxA-X-3058-10", 20950, 180)
    {
     Outlier(0.05);
    };
    R_Date("COL4219.1.1", 20905, 118)
    {
     Outlier(0.05);
    };
   };
   Boundary("Level 5/Level 4");
   Phase("Level 4")
   {
    R_Date("COL4218.1.1", 21007, 118)
    {
     Outlier(0.05);
    };
    R_Date("OxA-39750", 20760, 110)
    {
     Outlier(0.05);
    };
```

```
 R_Date("OxA-39502", 20930, 110)
 {
  Outlier(0.05);
 };
};
Boundary("Level 4/Level 3");
Phase("Level 3")
{
 R_Date("OxA-39501", 20910, 110)
 {
  Outlier(0.05);
 };
 R_Date("COL4216.1.1", 20950, 128)
 {
  Outlier(0.05);
 };
};
Boundary("Level 3/Level 2b");
Phase("Level 2b")
{
 R_Date("OxA-39500", 20450, 110)
 {
  Outlier(0.05);
 };
 R_Date("OxA-39506", 20399, 63)
 {
  Outlier(0.05);
 };
 R_Date("OxA-X-3048-17", 20308, 150)
 {
  Outlier(0.05);
 };
};
Boundary("Level 2b/Level 2a");
Phase("Level 2a")
{
 R_Date("COL4212.1.1", 20278, 107)
 {
  Outlier(0.05);
 };
 R_Date("OxA-39499", 20370, 110)
 {
  Outlier(0.05);
 };
 R_Date("COL4215.1.1", 20261, 111)
 {
```

```
   Outlier(0.05);
  };
  R_Date("COL4213.1.1", 20107, 111)
  {
   Outlier(0.05);
  };
  R_Date("COL4214.1.1", 19987, 110)
  {
   Outlier(0.05);
  };
 };
 Boundary("Level 2a/Level 1");
 Phase("Level 1")
 {
  R_Date ("OxA-39505", 19950, 110)
  {
   Outlier(0.05);
  };
  R_Date ("OxA-39498", 19928, 27)
  {
   Outlier(0.05);
  };
  R_Date("COL4210.1.1", 20008, 112)
  {
   Outlier(0.05);
  };
 };
 Boundary("Level 1/Level 0");
 Phase("Level 0")
 {
  R_Date ("OxA-42424", 18602, 83)
  {
   Outlier(0.05);
  };
  R_Date ("OxA-42423", 18608, 87)
  {
   Outlier(0.05);
  };
 };
 Boundary("End");
};
Sequence()
{
 Boundary("=Start");
 Date("Level 6");
 Boundary("=Level 6/Level 5");
```

```
};
Sequence()
{
 Boundary("=Level 6/Level 5");
 Date("Level 5");
 Boundary("=Level 5/Level 4");
};
Sequence()
{
 Boundary("=Level 5/Level 4");
 Date("Level 4");
 Boundary("=Level 4/Level 3");
};
Sequence()
{
 Boundary("=Level 4/Level 3");
 Date("Level 3");
 Boundary("=Level 3/Level 2b");
};
Sequence()
{
 Boundary("=Level 3/Level 2b");
 Date("Level 2b");
 Boundary("=Level 2b/Level 2a");
};
Sequence()
{
 Boundary("=Level 2b/Level 2a");
 Date("Level 2a");
 Boundary("=Level 2a/Level 1");
};
Sequence()
{
 Boundary("=Level 2a/Level 1");
 Date("Level 1");
 Boundary("=Level 1/Level 0");
};
Sequence()
{
 Boundary("=Level 1/Level 0");
 Date("Level 0");
 Boundary("=End");
};
Sequence("Total")
{
 Boundary("=Start");
```

```
 Date("Peña Capón");
 Boundary("=End");
 };
};
```

Peña Capón	Unmodelled (cal BP)				Modelled (cal BP)				Indices		
	68.3% probability range		95.4% probability range		68.3% probability range		95.4% probability range		A_{model}=106.3 $A_{overall}$=104.8		
	from	to	from	to	from	to	from	to	A	P	C
Boundary End					22514	22004	22758	20956			100
OxA-42423	22616	22380	22860	22350	22626	22396	22866	22356	100	96.2	100
OxA-42424	22596	22380	22836	22348	22620	22390	22854	22356	98	95.8	100
↑ Phase Level 0											
Boundary Level 1/Level 0					24016	23576	24080	22460			100
COL4210.1.1	24140	23876	24260	23806	24036	23858	24148	23812	111.4	96.1	100
OxA-39498	23968	23830	24092	23806	23970	23838	24090	23810	100.3	95.9	100
OxA-39505	24090	23836	24220	23790	24022	23846	24134	23798	113.4	96.2	100
↑ Phase Level 1											
Boundary Level 2a/Level 1					24208	23988	24246	23900			100
COL4214.1.1	24116	23864	24240	23804	24252	24082	24314	23952	71.5	94.7	100
COL4213.1.1	24238	23938	24466	23842	24280	24106	24376	23964	106.2	95.8	100
COL4215.1.1	24530	24190	24676	23970	24336	24152	24480	24066	113.6	96	100
OxA-39499	24628	24270	24878	24174	24364	24190	24494	24124	87.7	95.2	100
COL4212.1.1	24542	24210	24700	24012	24338	24164	24490	24086	108	95.8	100
↑ Phase Level 2a											
Boundary Level 2b/Level 2a					24462	24246	24586	24188			100
OxA-X-3048-17	24624	24188	24904	23970	24648	24362	24872	24270	107.7	96	100
OxA-39506	24614	24340	24746	24226	24650	24414	24752	24290	105.5	95.6	100
OxA-39500	24770	24330	24958	24238	24718	24400	24902	24306	110.8	95.8	100
↑Phase Level 2b											
Boundary Level 3/Level 2b					25150	24600	25190	24470			100
COL4216.1.1	25550	25100	25654	24980	25184	25026	25260	24904	85.2	95.6	100
OxA-39501	25344	25028	25608	24962	25186	25026	25254	24914	102.9	95.7	100
↑Phase Level 3											
Boundary Level 4/Level 3					25230	25096	25308	25010			100
OxA-39502	25514	25068	25618	24990	25270	25134	25390	25072	137.9	96.2	100
OxA-39750	25204	24902	25286	24660	25254	25132	25348	25058	77.2	95.3	100
COL4218.1.1	25560	25194	25672	25078	25278	25140	25402	25078	103.1	95.8	100
↑Phase Level 4											
Boundary Level 5/Level 4					25328	25156	25504	25110			100
COL4219.1.1	25350	25012	25626	24932	25550	25210	25610	25170	79.3	95.6	100
OxA-X-3058-10	25568	25086	25730	24792	25558	25252	25666	25170	111.4	96	100
↑ Phase Level 5											

Peña Capón	Unmodelled (cal BP)				Modelled (cal BP)				Indices		
	68.3% probability range		95.4% probability range		68.3% probability range		95.4% probability range		A_{model}=106.3 $A_{overall}$=104.8		
	from	to	from	to	from	to	from	to	A	P	C
Boundary Level 6/Level 5					25874	25510	25920	25262			100
COL4221.1.1	25964	25790	26070	25668	25948	25790	26026	25706	107.2	96.2	100
COL4222.1.1	25964	25794	26070	25672	25948	25792	26014	25714	107.4	96	100
↑ Phase Level 6											
Boundary Start					26142	25806	26836	25790			100
Sequence Peña Capón											
U(0,4)	3.98986e-17	4	3.98986e-17	4	5.37764e-17	2.3	5.37764e-17	3.956	100		100
T(5)	-1.14	1.14	-2.65	2.65							100
Outlier_Model General					-52	48	-256	328			100

Table S4. Detailed results of the Bayesian Model for the Peña Capón sequence (Levels 0 to 6). Modeled calibrated ages and Boundaries with the 68.3% and 95.4% probability ranges. Calculated with OxCal 4.4 (*211*), using a General t-type Outlier Model (*213*) and IntCal20 (*212*.

Level (phase)	Duration (cal BP)			
	68.2% probability		95.4% probability	
Peña Capón	25754	22796	25986	22328
0 (Upper Solutrean)	23228	22354	23854	22178
1 (Middle Solutrean)	24150	23690	24210	22882
2a (Middle Solutrean)	24330	24122	24470	23990
2b (Middle Solutrean)	24778	24350	25048	24270
3 (Middle Solutrean)	25210	24964	25264	24656
4 (Proto-Solutrean)	25280	25140	25402	25064
5 (Terminal Gravettian)	25620	25256	25800	25190
6 (Terminal Gravettian)	25970	25726	26172	25430

Table S5. Calendar age estimates for each archaeological level at Peña Capón, based on the Bayesian Model (Fig. S11) as calculated by the ‘date’ command in Oxcal. Associated PDFs are shown in Figure S12.

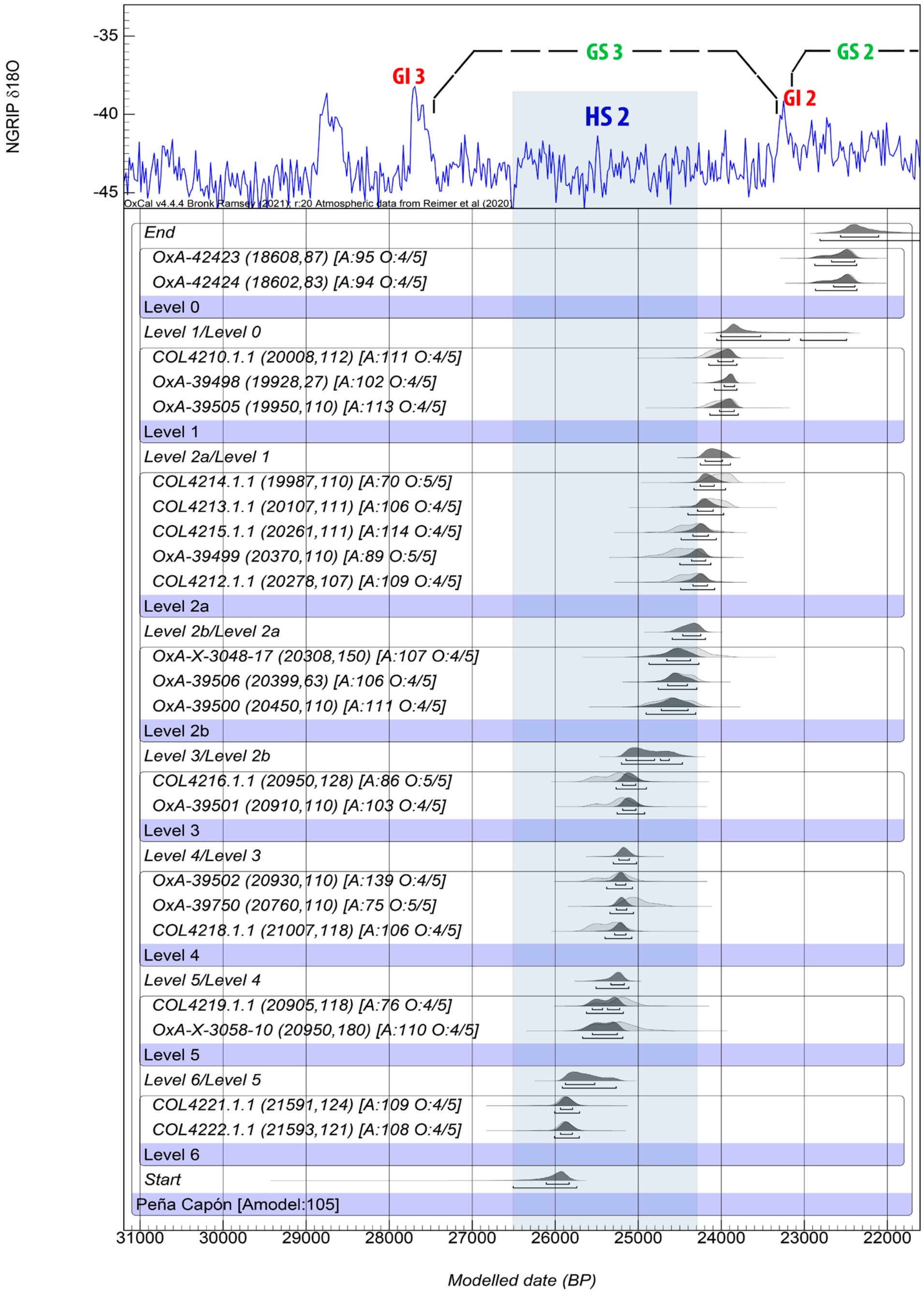

Figure S11. Bayesian Final Model (2) for the Peña Capón sequence showing Probability Distribution Functions (PDFs) for all radiocarbon determinations and boundaries between archaeological levels. Results are plotted against the δ18O record of the NGRIP ice core, indicating Greenland Interstadials 3 and 2 (GI 2 & GI 3), Greenland Stadial 3 (GS 3) (*214*), and the chronology of Heinrich Stadial 2 (blue bar) (*215*). 14C dates are shown in parentheses, and Agreement indexes and Outliers' prior and posterior probabilities are shown in square brackets. Calibration of dates and Bayesian modelling were calculated using OxCal 4.4 online software.

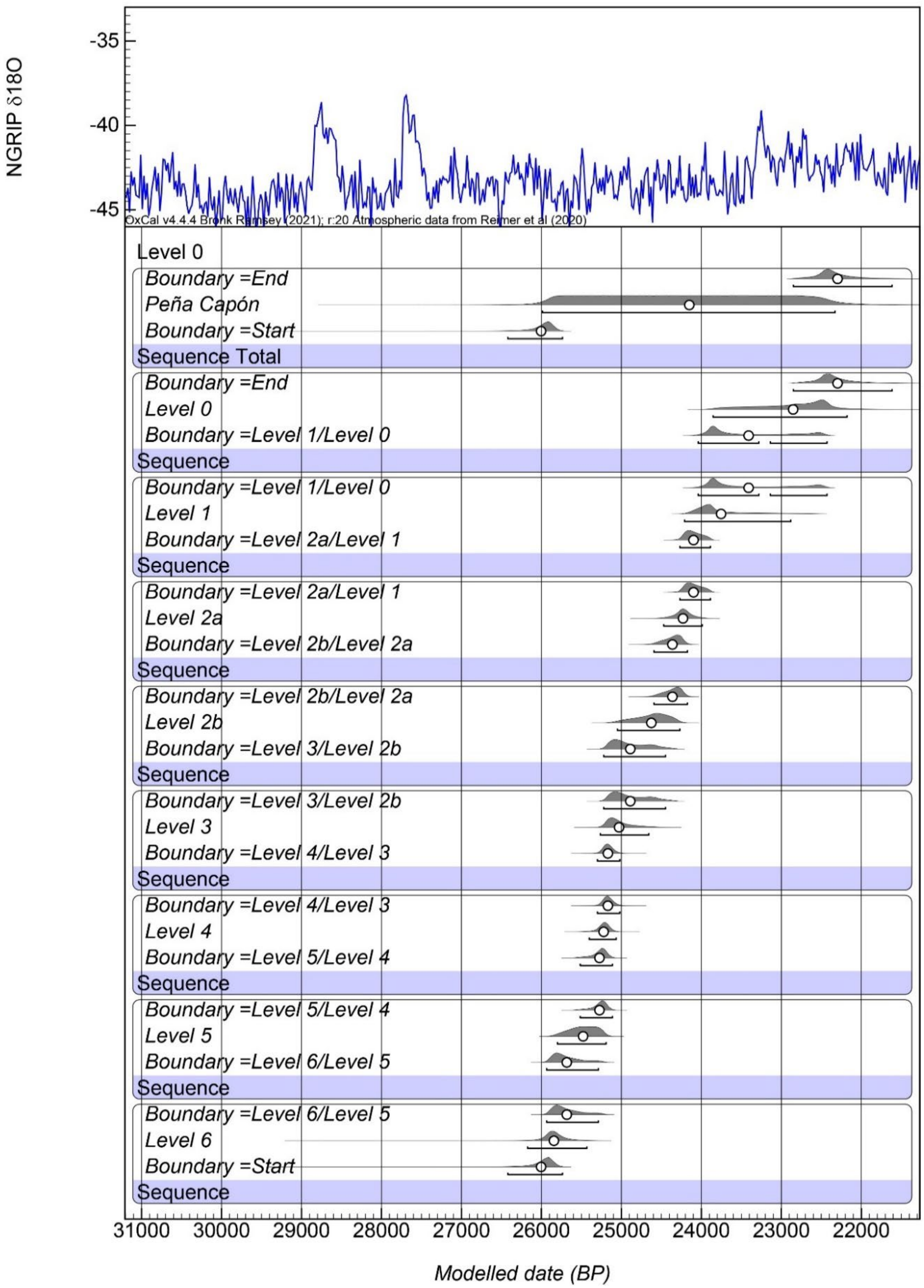

Figure S12. Probability Distribution Functions (PDFs) for the estimated duration of all archaeological levels at Peña Capón, based on the Bayesian Model as calculated by the 'date' command in Oxcal. Results are plotted against the δ18O record of the NGRIP ice core.

1.5. Archaeological sequence

The archaeological sequence recorded at Peña Capón covers most of the Solutrean time span, including the Proto-Solutrean, as well as the late phases of the Gravettian. As previously reported (*59, 117-121*), Peña Capón hosts a sequence of recurrent occupations of hunter-gatherers which, to date, has no parallel in the whole Iberian *Meseta* (*78, 82*). Together with anthropogenically-modified faunal remains, a limited number of bone tools, ochre fragments and fire structures throughout the sequence, lithics account for the most abundant archaeological material (Figs S13 to S20). Foliate lithic armatures obtained through bifacial and unifacial invasive flat retouch are found throughout levels 1 to 3, between 25.3 and 22.9 ka cal BP (Table S5). Considering the classic typology-based chronological framework of the Solutrean, assemblages from these levels are classified as Middle Solutrean (*59*) based on the presence of bifacial laurel leaf points (Fig. S21: 5 to 15) and the absence of typically Upper Solutrean tools. The latter are found only in the uppermost layer, Level 0, in the form of shouldered points, including both 'Mediterranean' types with abrupt retouch (Fig. S21: 2 & 3). and 'Cantabrian' types with flat invasive retouch (Fig. S21: 1 & 4). Based on these armatures, Layer 0, dated to 23.8 – 22.2 ka cal BP, is classified as Upper Solutrean.

As for the previously termed 'pre-Solutrean' levels (*59*), new data from the 2021 and 2022 seasons enable us to confirm the assignment of Level 4 to the Proto-Solutrean, and of Levels 5 and 6 to the Terminal Gravettian, thus validating our previous hypotheses. The presence of at least three Vale Comprido points in Level 4 (Fig. S22: 5), together with the dating of this level between 25.4 and 25.1, thus matching the Proto-Solutrean time span in Portugal between ~26 and 25 ka cal BP (*64, 127, 128*), support this attribution. It is thus confirmed that Level 4 corresponds to Level III of the 1972 excavations, where a Proto-Solutrean component with Vale Comprido points (Fig. S22: 1-3; Figure S8: 7-9) was first recognized (*116*). Likewise, the chronology of Levels 5 and 6 precisely match that of Level IV of the 1972 excavation, and they all are now classified as Terminal Gravettian. This technocomplex was first defined in Portugal as a transitional sub-phase between the Late Gravettian and the Proto-Solutrean/Lower Solutrean, and their main features are the intensive use of quartz, the exploitation of carinated cores, and a preference for the use of marginally retouched (not backed) bladelets (*129, 130*). All these features are found in Levels 5 and 6 of Peña Capón (Fig. S22: 6-12), which are radiocarbon dated to 26.2 – 25.2 ka cal BP, and hence are situated within the timeframe of the Portuguese Terminal Gravettian (*130*).

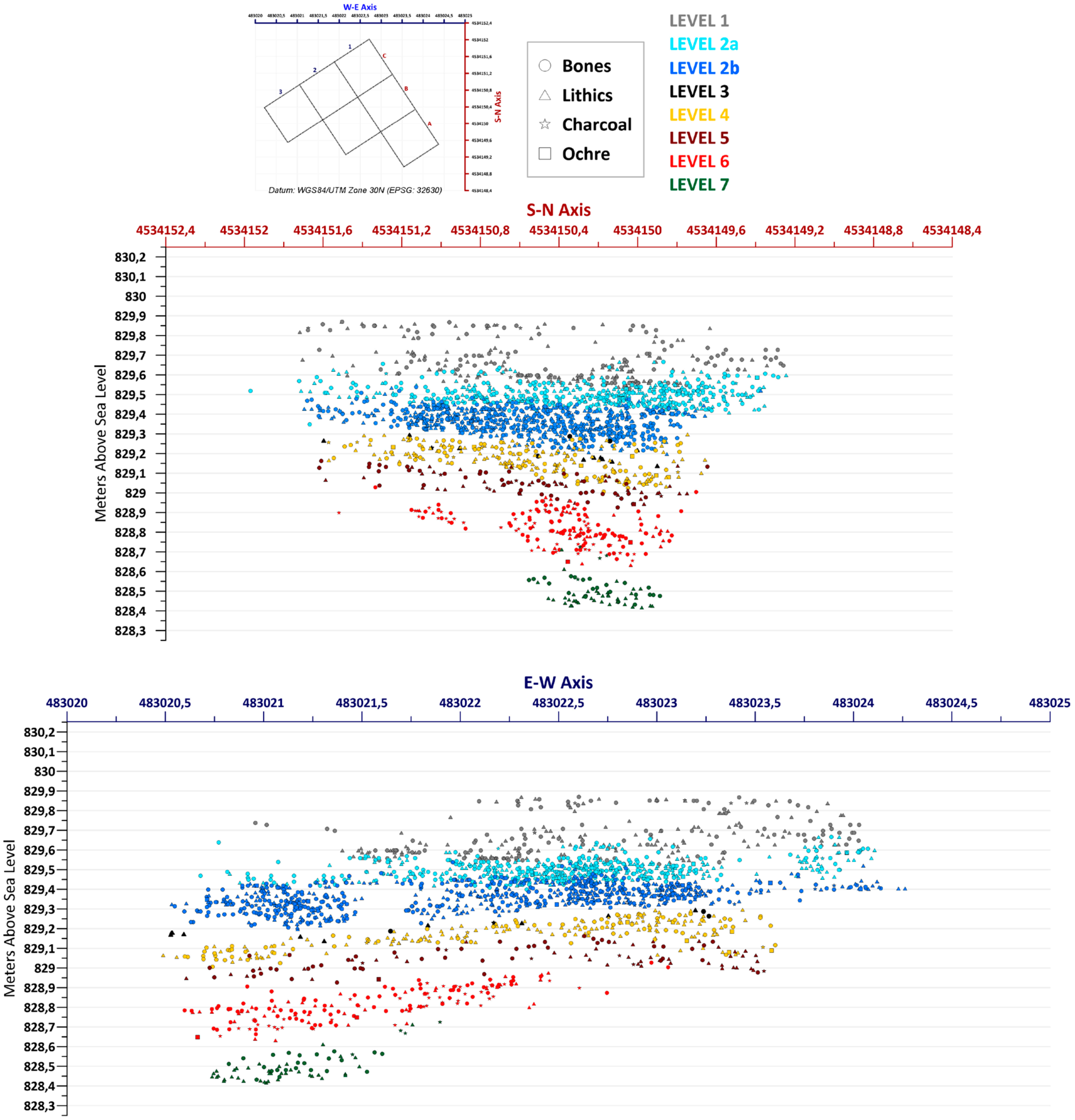

Figure S13. Vertical distribution of archaeological items > 2 cm recorded throughout the Peña Capón sequence in the Central Pit. Both N-S axis and E-W axis are represented.

With respect to the nature and function of the human occupations throughout the sequence, all Middle Solutrean levels except Level 3 (Figs S13 to S20) show high densities of artefacts, and in all of them lithic projectile points are the most represented artefacts (Dataset S6). This strongly points to hunting and game processing as the main activities explaining the presence of humans at the rock shelter, as also indicated by the high presence of anthropogenic modifications on the bone remains of ungulates (mostly red deer, horse, ibex) and leporids. Cut and percussion marks, as well as thermal alterations, are found in all layers, suggesting that all cooking processes related to faunal individuals, including marrow extraction, were conducted at

the site (*59*) (Fig. S23). Furthermore, the significant presence in most of these assemblages of 'domestic' tools, like endscrapers, scaled pieces and burins (Dataset S6), together with the existence of ochre remains and fire structures at least in Levels 2a and 2b (Figs. S15 and S16), suggest that the settlement of the rock shelter by humans during the Middle Solutrean also entailed a residential motivation, either as part of a forager-like or, most probably, a collector-like strategy (*131*) along the forager-collector continuum (*14, 132*). Likewise, the low percentages of cores, core-maintenance pieces and initialisation products, suggest that the initial knapping of lithic raw materials was not carried out at the site during the Middle Solutrean.

A different occupation strategy was developed during the Upper Solutrean as reflected in Level 0, where the presence of cores, core-maintenance products and raw blanks in the form of blade and bladelet products, is significantly higher than in Levels 1-3. However, projectile points, here represented by shouldered points, are still the dominant artefacts (Dataset S6). A similar pattern is observed during the Proto-Solutrean of Level 4, where cores and blade blanks are also present, endscrapers are significantly abundant, and lithic projectile points are also relevant, in this case in the form of Vale Comprido points (Dataset S6).

Finally, the Terminal Gravettian occupations of Levels 5 and 6 reflect a clear break with respect to lithic exploitation, as well as concerning hunting strategies, land use and mobility patterns. Lithic assemblages are dominated by the exploitation of quartz by means of mostly expedient or low-cost technologies (Dataset S6). Together with a strong preference for horses as prey, a lower density of artefacts per square meter and the existence of small fireplaces, this suggests that these assemblages reflect cultural adaptations to specific needs that arose during short stays at the rock shelter related to game processing. Lithic projectile points are also found in these layers, as shown by the presence of diagnostic impact fractures (DIF's) in some chert blanks, but they are significantly less abundant than in the Solutrean and Proto-Solutrean levels.

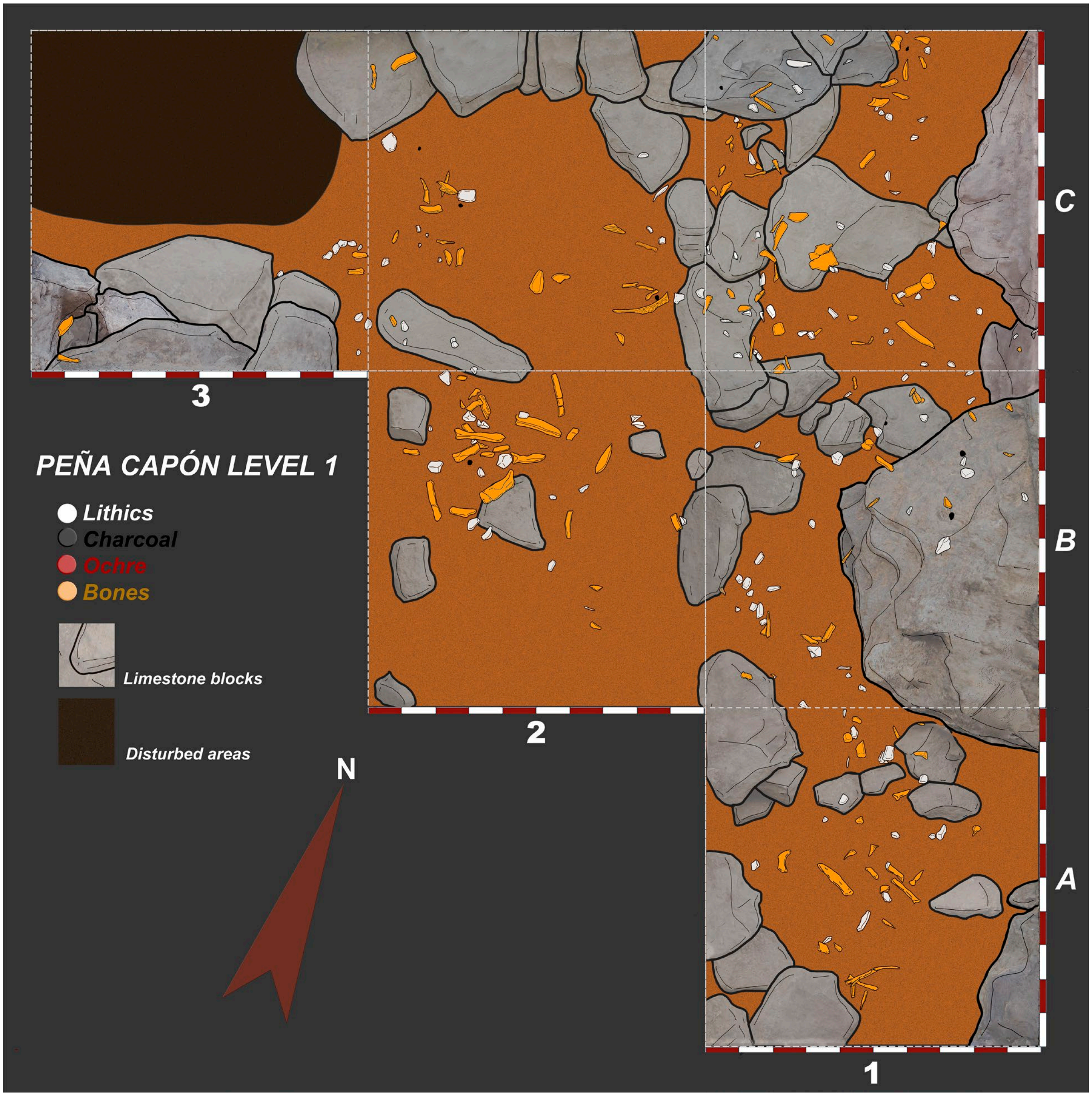

Figure S14. Plan of level 1 showing recorded archaeological items > 2 cm.

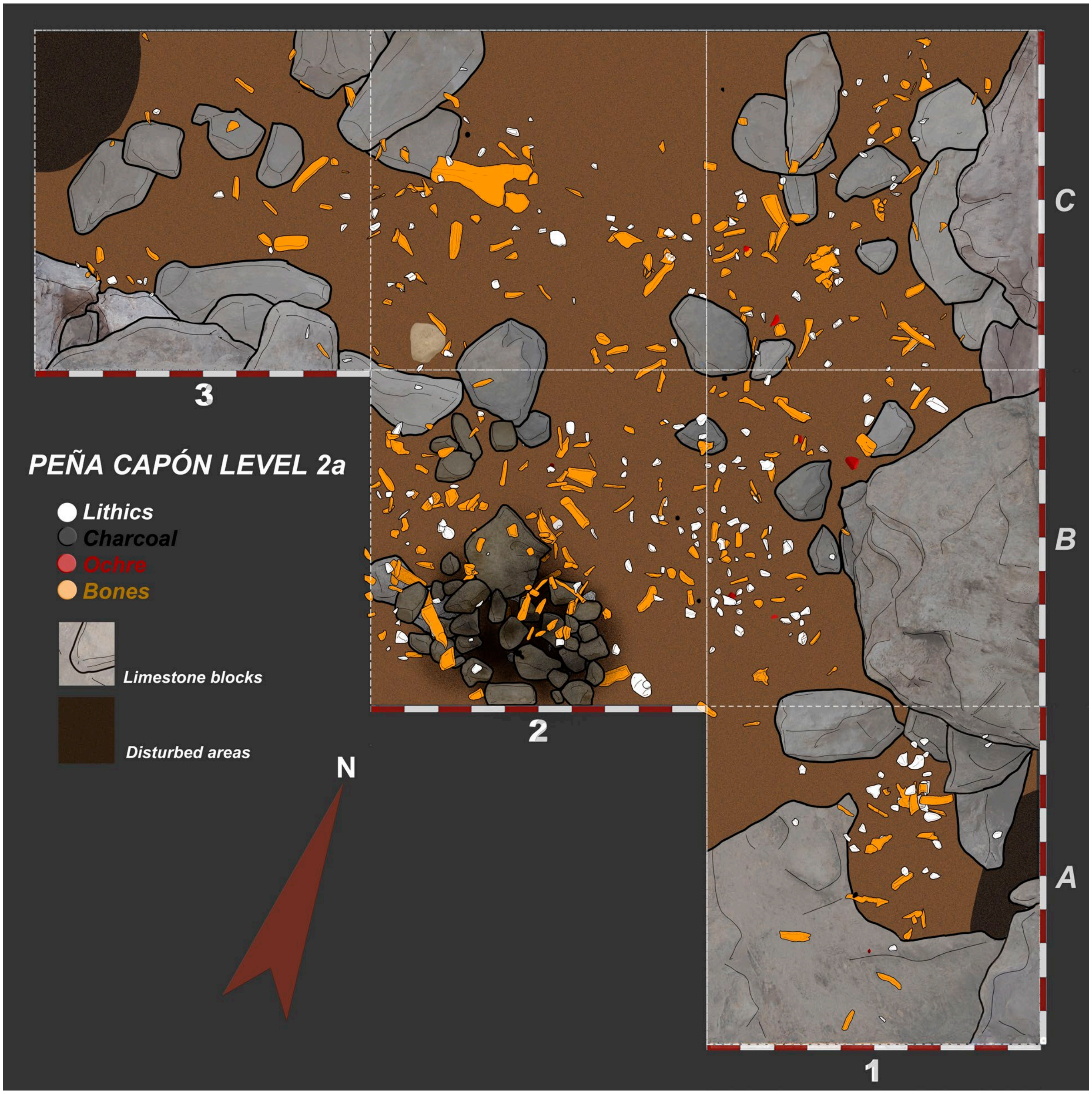

Figure S15. Plan of level 2a showing recorded archaeological items > 2 cm.

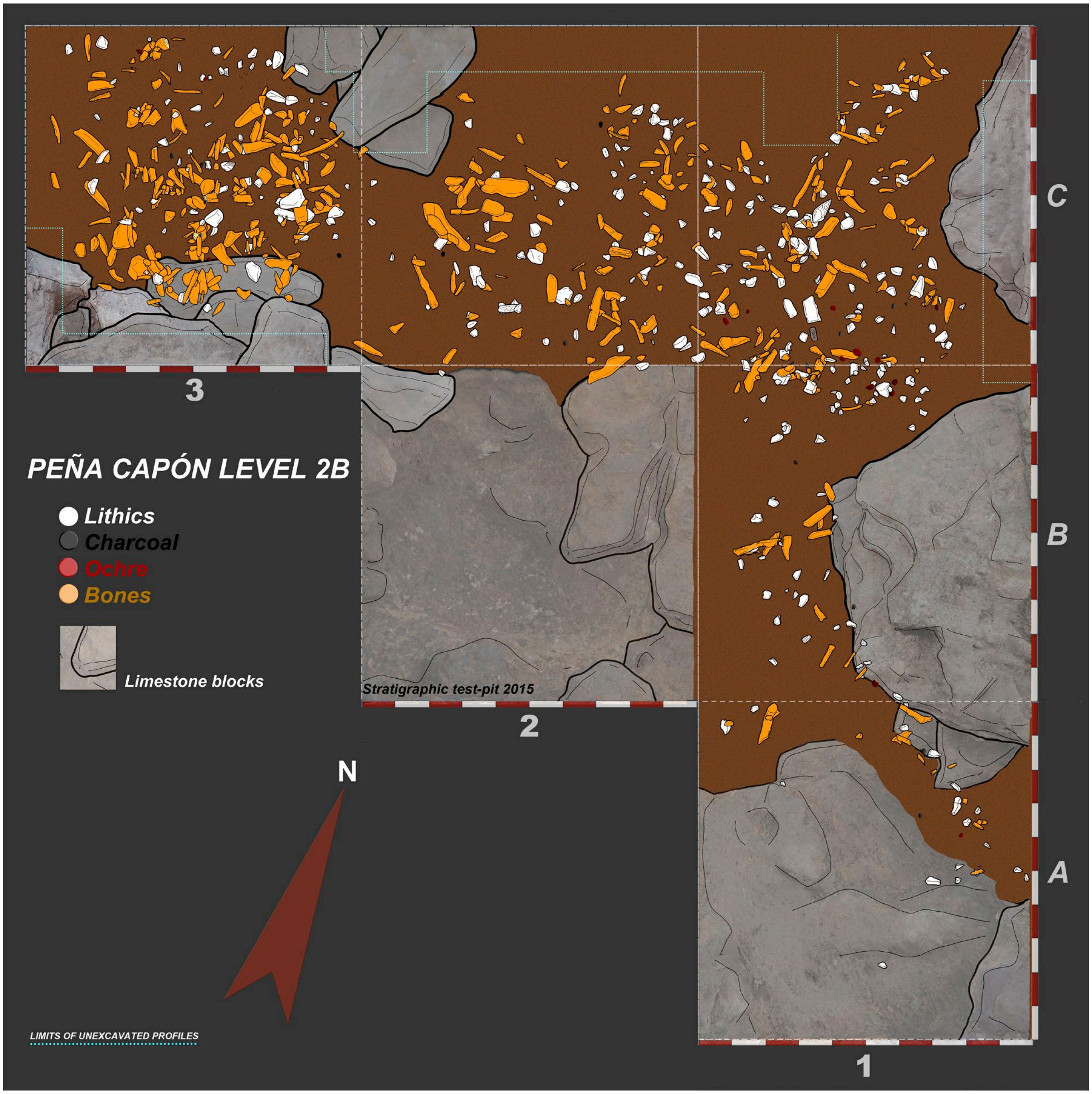

Figure S16. Plan of level 2b showing recorded archaeological items > 2 cm.

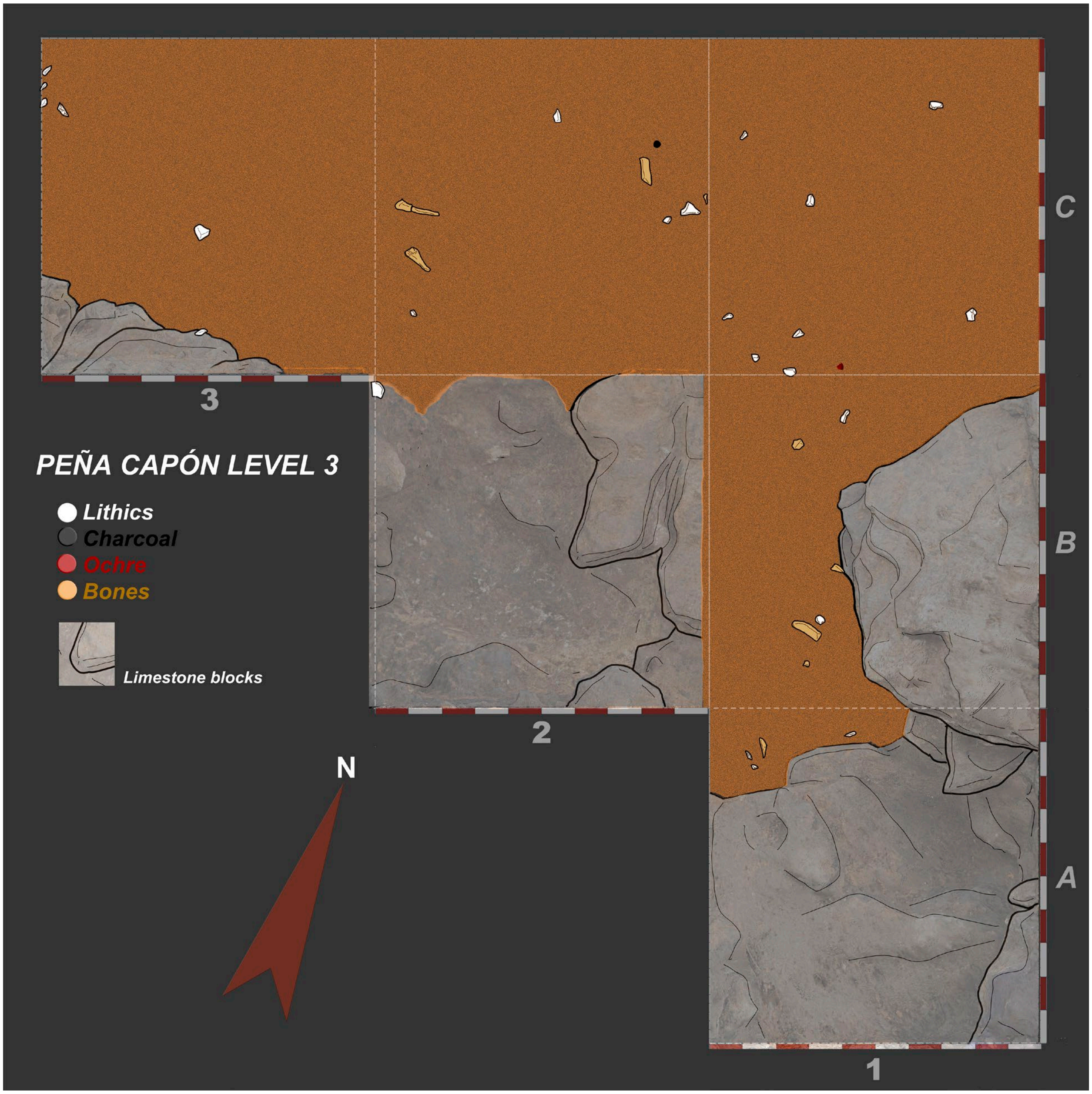

Figure S17. Plan of level 3 showing recorded archaeological items > 2 cm.

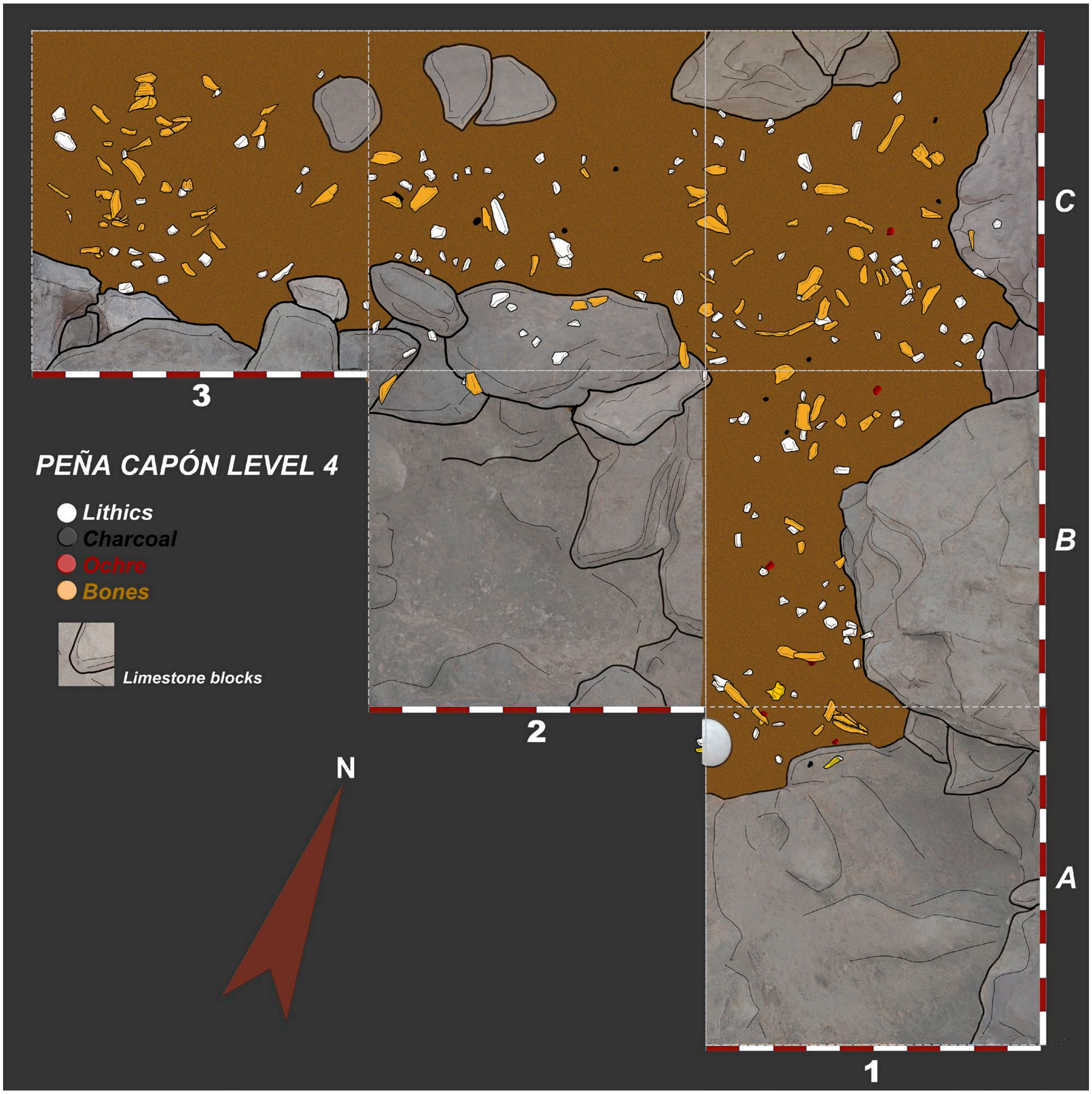

Figure S18. Plan of level 4 showing recorded archaeological items > 2 cm.

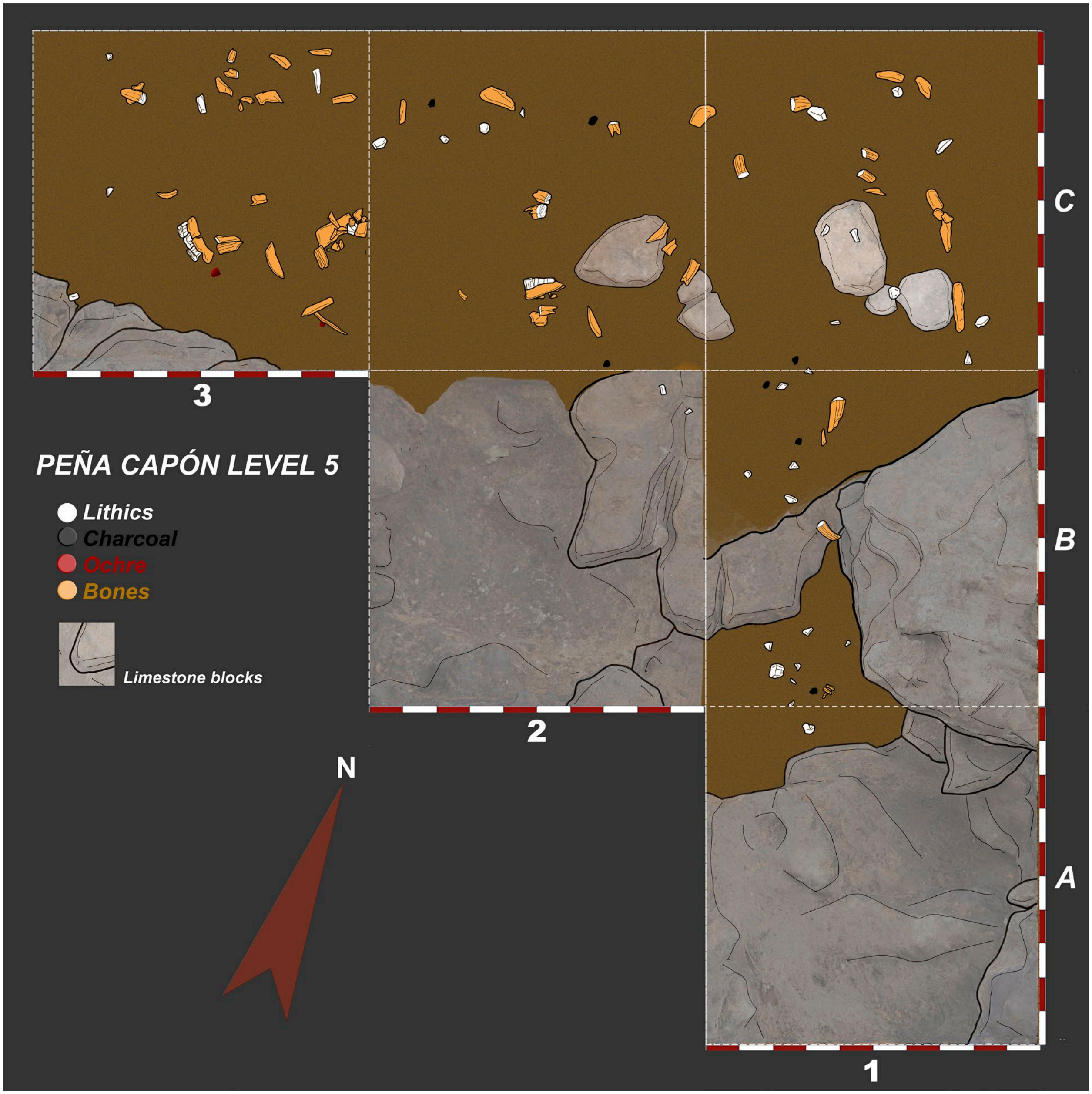

Figure S19. Plan of level 5 showing recorded archaeological items > 2 cm.

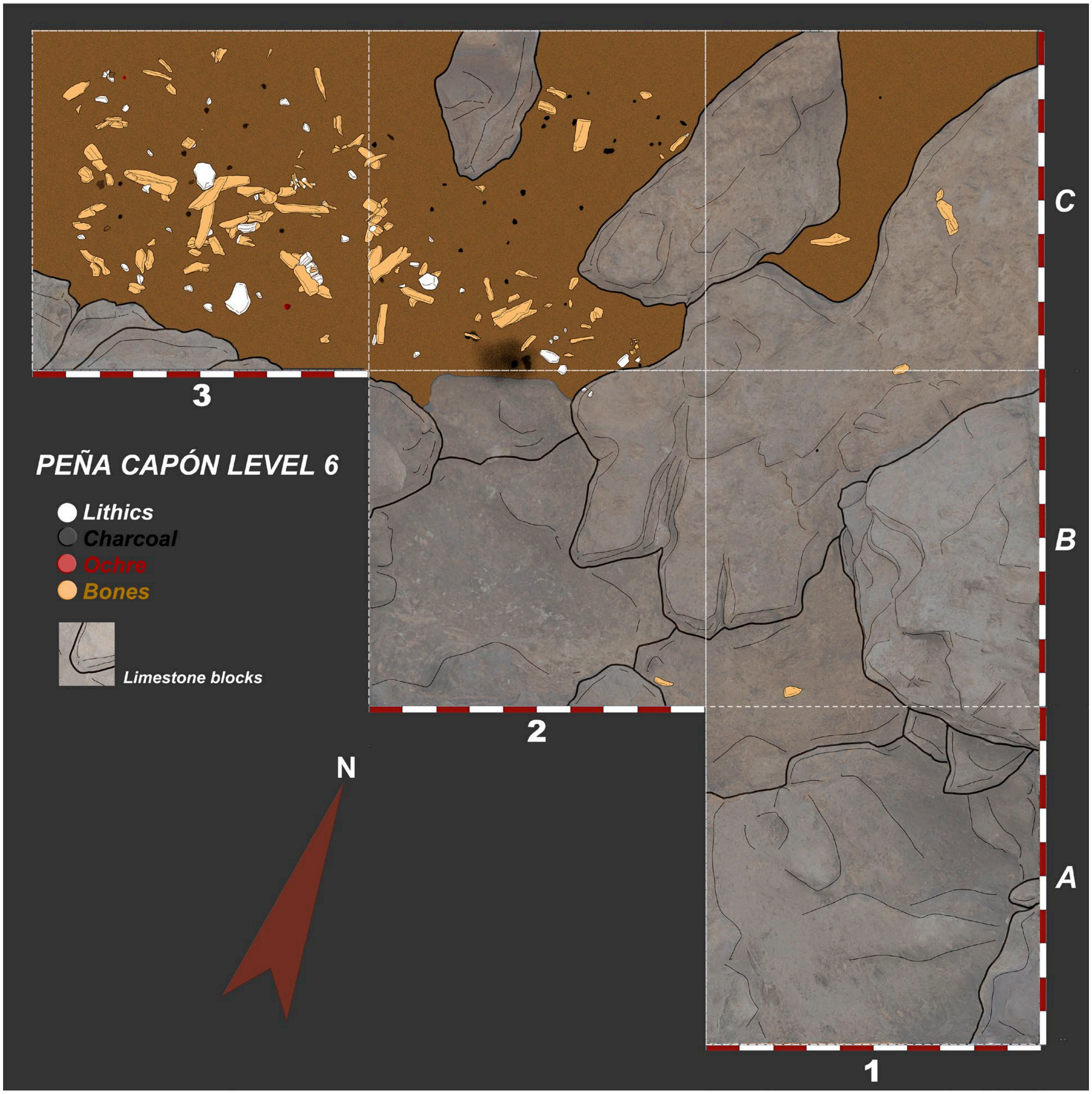

Figure S20. Plan of level 6 showing recorded archaeological items > 2 cm.

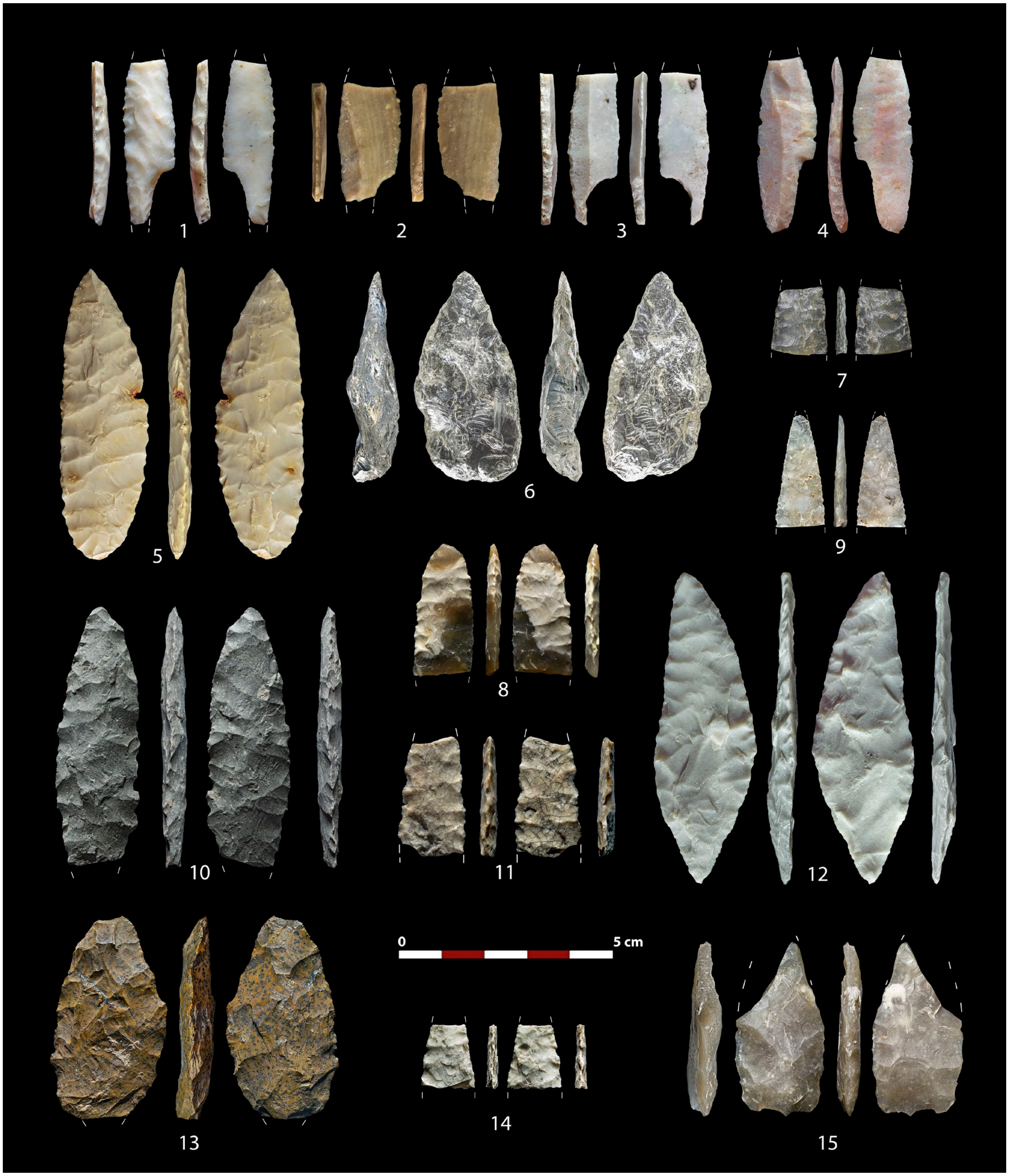

Figure S21. Selected foliate points from the Solutrean levels of Peña Capón. 1-4: Shouldered points from the Upper Solutrean (Level 0). 5-15: Laurel leaf points from the Middle Solutrean (Level 1: 5-8. Level 2a: 9. Level 2b: 10-12. Level 3: 13-15), including preforms in different stages of reduction (6, 13 & 15). Photo credits: Manuel Alcaraz-Castaño.

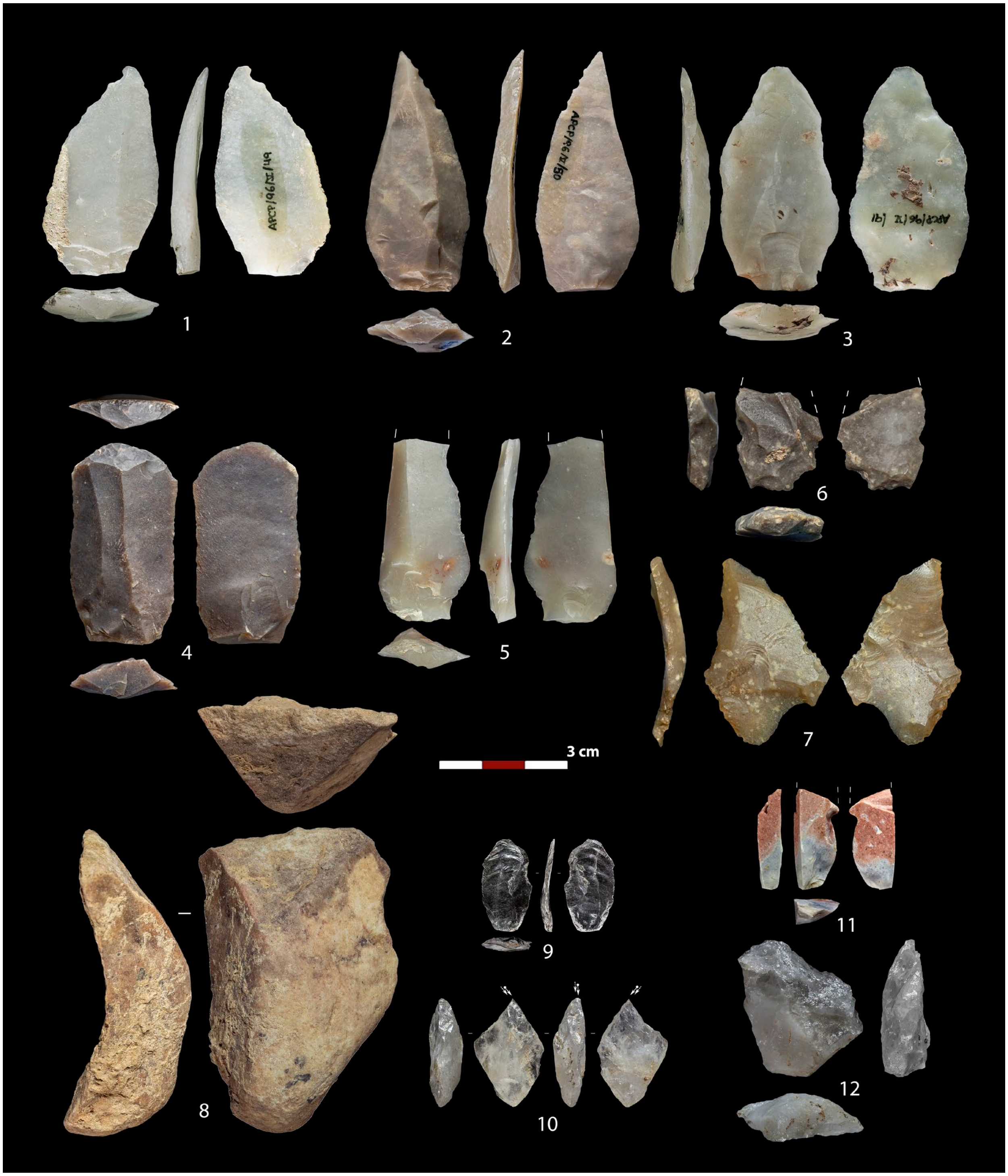

Figure S22. Selected artefacts from the Proto-Solutrean and Terminal Gravettian levels of Peña Capón. Level 4 (Proto-Solutrean): Vale Comprido points (1, 2, 3 & 5), and endscraper (4). Level 5 (Terminal Gravettian): notch (6) and retouched flake (7). Level 6 (Terminal Gravettian). Endscraper on large flake (8), rock crystal bladelet (9), dihedral burin (10), bladelet (11) and notch (12). Photo credits: Manuel Alcaraz-Castaño.

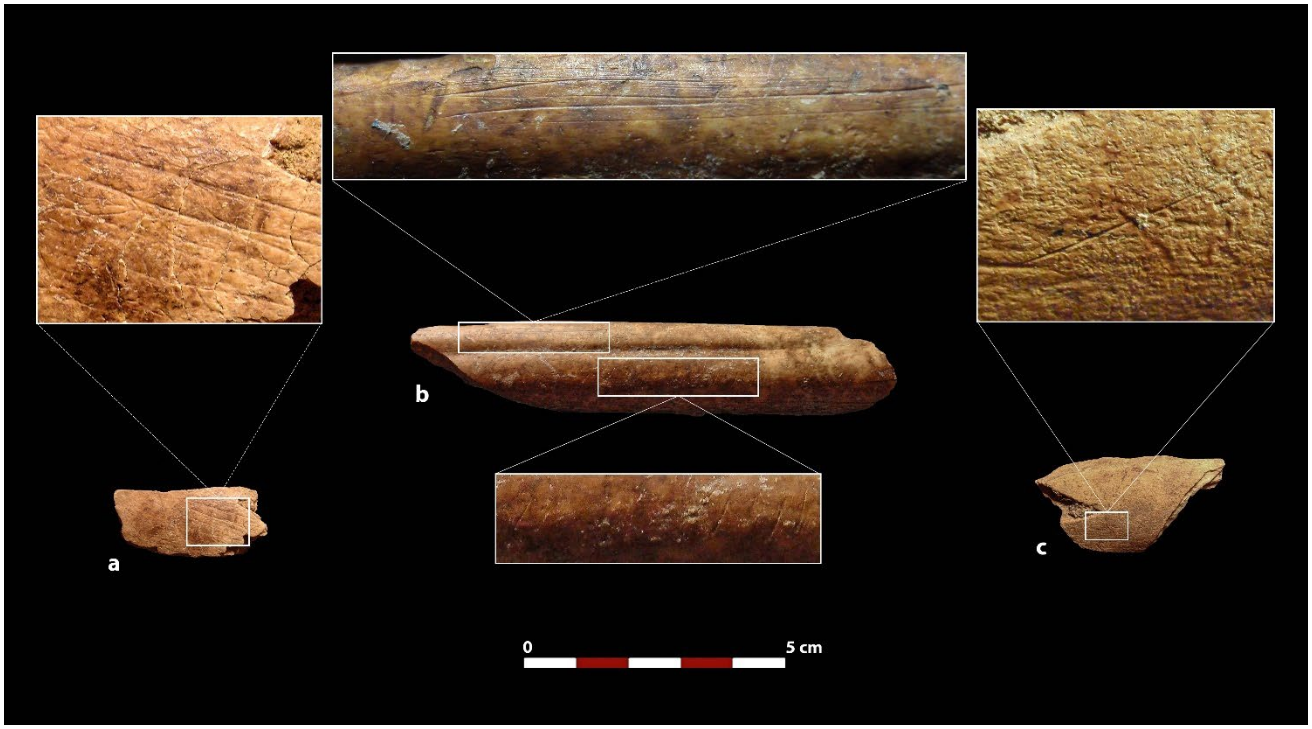

Figure S23. Examples of cut-marked shafts from level 1 (a), level 2a (b) and level 3 (c). All of them were sampled for radiocarbon dating (OxA-39498, OxA-39499, OxA-39501 respectively; see Table S3). Photo credits: Manuel Alcaraz-Castaño.

1.6. Cultural and palaeoecological significance

The archaeological levels recorded at Peña Capón reflect a sequence of recurrent and virtually uninterrupted human occupations spanning the central part of the Upper Palaeolithic which, to date, lacks a direct counterpart in the whole Iberian hinterland (*78, 82*). Most of the known sequence was developed during Greenland Stadial 3 (GS 3), a stadial phase within the LGM [sensu (*58*)]. More significantly, a relevant part of it (Levels 6 to 2b), covering the Terminal Gravettian, the Proto-Solutrean and most of the Middle Solutrean, coincided with the rapid cooling of Heinrich Stadial 2 (HS 2) (Fig. S11). Harsh environmental conditions are confirmed for Levels 6, 5, 4 and 1 by palaeoenvironmental data, including pollen, micromammal and anthracological analyses. These data indicate the predominance of pine forests at higher altitudes and evergreen oak and juniper woodlands at lower ones, as well as of shrub and herb communities dominated by cryoxerophytic and heliophilous/cryophilous elements, together with the presence of cold-adapted microfauna, such as *Microtus agrestis* and a general loss of biodiversity compared to levels 3, 2b and 2a (*59*). The latter levels, corresponding to the bulk of the Solutrean occupations between 25.3 and 24.0 ka cal BP (levels 2a, 2b and 3) were developed during a warmer period, as especially revealed by the presence of deciduous oak groves enriched with numerous mesophilous trees, including beech, and the existence of animals well-adapted to wooded environments, such as roe deer, wildcat and badger (*59*).

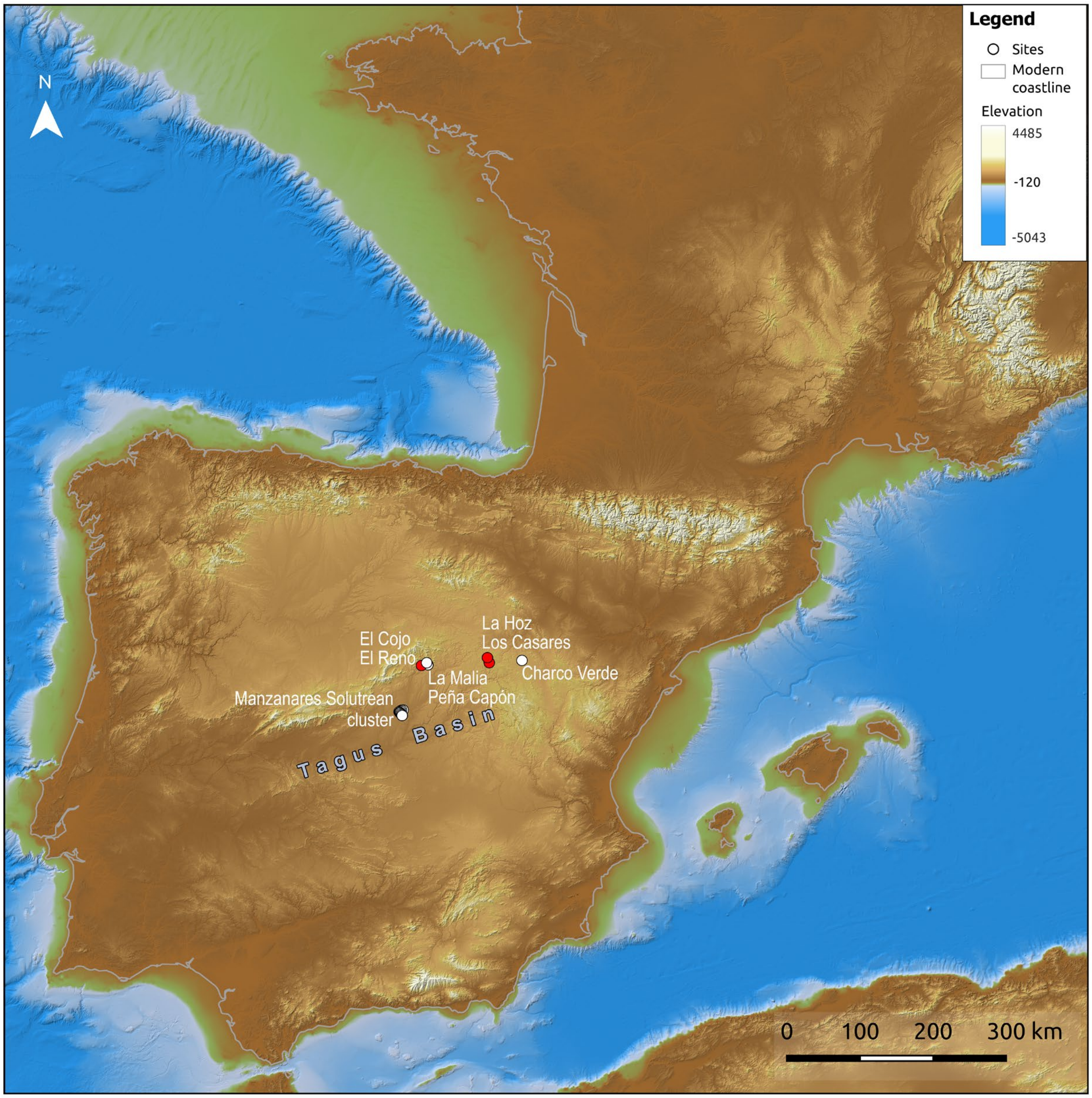

Figure S24. Archaeological sites bearing evidence of human occupations during the LGM in the Middle and Upper Tagus basin (*Charco Verde belongs to the Ebro basin, very close to the Tagus basin limit). Red circles: sites with pre-Magdalenian rock art motifs [see (*59*) for discussion].

This alternation of warmer and harsher environmental conditions developed during a stadial phase bears relevant implications for understanding human-climate-environment interactions during the settlement of the Iberian interior by modern humans. The Peña Capón data contradicts traditional views and recent models positing that harsh climatic and environmental conditions hampered the human occupation of inland Iberia during most of the Upper Palaeolithic, as they demonstrate that this area of the Iberian hinterland was recurrently occupied regardless of climatic and environmental variability (*59, 78*). The nearby La Malia rock

shelter (*82*), and the more distant but still interior site of Charco Verde II (*85*), have recently shown results demonstrating the existence of human occupations in high altitude inland environments during cold and arid periods of the LGM. Together with other regional sites showing archaeological evidence during this period in the Middle and Upper Tagus basin, including El Reno and Los Casares rock art motifs and the Solutrean cluster of the Manzanares River, in Madrid (*78, 83, 120, 133-135*) (Fig. S24), these evidences make increasingly clear that there was an organized and complex settlement throughout this basin, covering not only the whole LGM time frame, but beginning during Aurignacian times, as shown by the evidence recorded at La Malia (*82*).

Text S2. Supplementary micropalaeontological and textural results on chert sourcing

2.1. Archaeological assemblages

1,041 lithic artefacts from Peña Capón, corresponding to most of the retouched artefacts throughout the sequence and a selection of non-retouched products, were subject to micropalaeontological and textural analysis (Table S6). This allowed the identification of five chert varieties from three sedimentary environments, besides an indeterminate group composed of 22 samples that were too altered to be studied. The main macroscopic characteristics of each defined chert type (Figure S25) are the following:

		Lithotype 1		Lithotype 2		Lithotype 3		Lithotype 4		Lithotype 5		Undefined	
**Level**	**Samples**	**n**	**%**	**n**	**%**	**n**	**%**	**n**	**%**	**n**	**%**	**n**	**%**
Surface	8	2	25	0	-	0	-	3	37.5	3	37.5	0	-
0 (Upper Solutrean)	3	2	66.7	0	-	0	-	1	33.3	0	-	0	-
1 (Middle Solutrean)	327	298	91.1	5	1.5	5	1.5	14	4.3	3	0.9	2	0.6
2a (Middle Solutrean)	156	129	82.7	8	5.1	5	3.2	6	3.9	4	2.6	4	2.6
2b (Middle Solutrean)	320	240	75	13	4.1	0	-	33	10.3	21	6.6	13	4.1
3 (Middle Solutrean)	155	139	89.7	5	3.2	4	2.6	4	2.6	1	0.65	2	1.3
4 (Proto-Solutrean)	27	20	74.1	1	3.70	0	-	5	18.5	0	-	1	3.70
5 (Terminal Gravettian)	27	24	88.9	0	-	0	-	3	11.1	0	-	0	-
6 (Terminal Gravettian)	18	16	88.9	1	5.6	0		1	5.6	0	-	0	-
**Total**	**1041**	**870**	**83.6**	**33**	**3.17**	**14**	**1.3**	**70**	**6.7**	**32**	**3.1**	**22**	**2.1**

Table S6. Distribution of lithic samples subject to macroscopic analysis throughout the stratigraphic sequence of Peña Capón and their assignment to the five identified lithological groups.

- 870 samples were classified as **lithotype 1**. This chert type shows whitish colorations with a quite smooth original relicts of a mudstone texture. The metal oxides and the gypsum lenticular pseudomorph constitute the main inclusions content. This chert does not possess bioclastic content, as its origin is found in a hypersaline continental sedimentary environment.
- 33 samples were classified as **lithotype 2**. This chert possesses reddish to brownish colorations in an originally mudstone to wackestone texture. The main inclusion content is constituted by metal oxides and micritic residues, being abundant depending on the sample. The bioclastic content is absent, as these cherts originated in a hypersaline sedimentary environment.
- 14 samples were classified as **lithotype 3**. This third evaporitic variety is composed of light brownish to whitish cherts with an originally azoic mudstone texture, being the inclusions and the micropalaeontological content virtually absent. The only observed inclusion are some blackish spots that might correspond to amorphous organic matter.
- 70 samples were classified as **lithotype 4**. They are composed of brownish colored cherts with a heterogeneous macroscopic variety of original textures ranging from wackestone to packstone type. The inclusions content includes metal oxides, micritic residues and detrital grains of quartz. The *charophyte algae* and the gastropods sections constitute the main micropalaeontological content. These cherts originated in a lacustrine sedimentary environment.
- 32 samples were classified as **lithotype 5**. This orange to reddish siliceous variety with extremely smooth surfaces was defined as a siliceous jasper variety regarding the macroscopic

texture. This type exhibits an original mudstone to wackestone texture with inclusions of metal oxides. Some blackish spots, probably corresponding to metal oxides or amorphous organic matter, were present in some samples (Fig. 5 in main text).

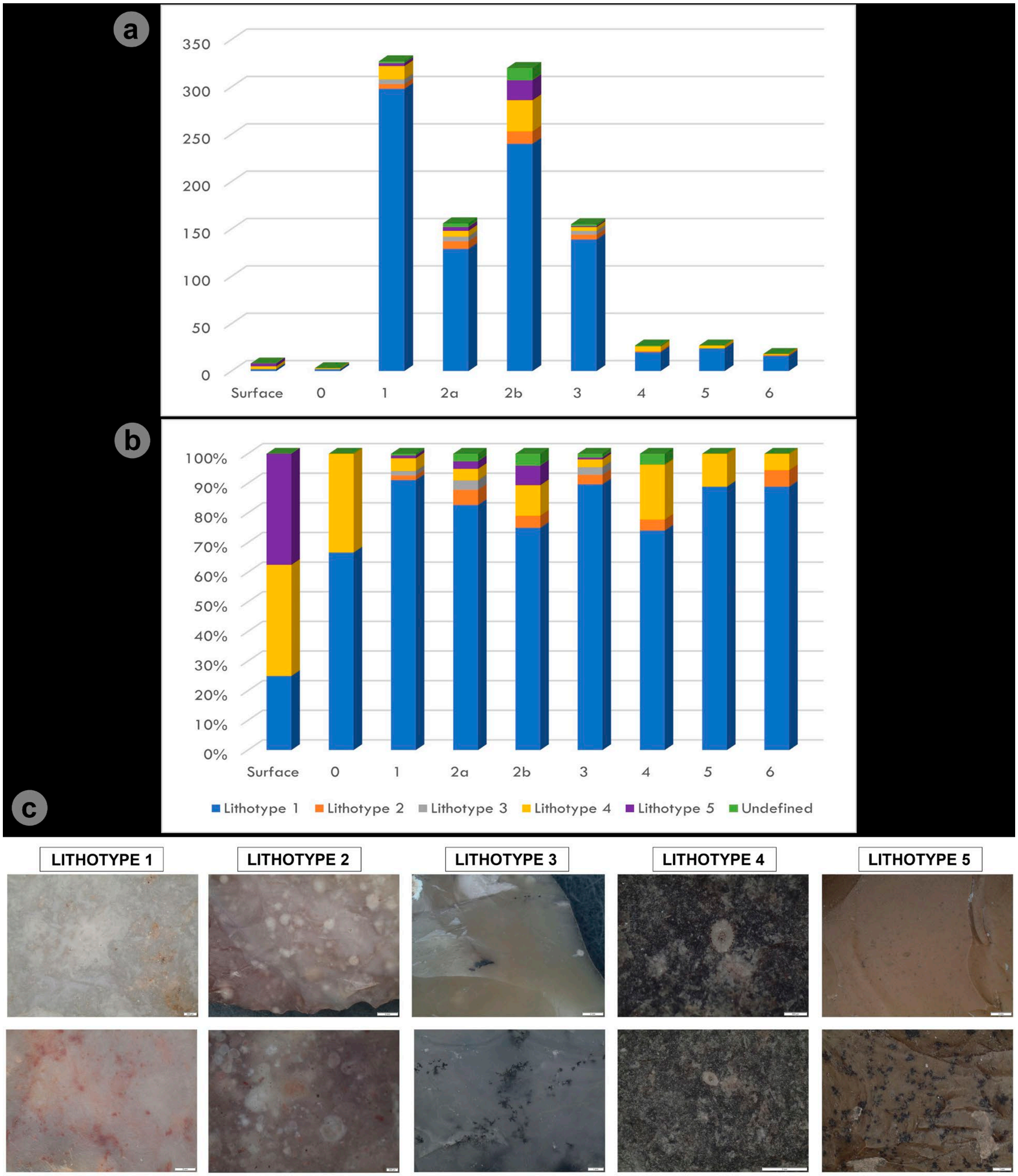

Fig. S25. Number (a) and percentage (b) of lithic artefacts assigned to each lithological group by stratigraphic level, and captions of the texture from the five lithotypes at the stereoscopic microscope (c).

Evaporitic cherts are the dominant rocks in all archaeological levels. Lithotype 1 is always the most represented variety throughout the sequence, with averages usually above 70%. Lithotype 2 appears in most levels except 0 and 5, with averages between 1 and 5%, and Lithotype 3 was only minimally identified in the Middle Solutrean occupations, except for Level 2b, where is absent. The lacustrine chert variety defined as lithotype 4 is found in all levels, ranging from 2.6 to 18.5% (besides Level 0, where the sample is too low to be considered statistically significant). Finally, the jasper variety defined as lithotype 5 was only detected in limited quantities in the Middle Solutrean occupations (Levels 1 to 3) (Figs. 2 & 3 in main text).

2.2. Geological samples

For the evaporitic cherts defined as lithotypes 1 to 3 in the archaeological assemblages, we found five different geological units showing similar macroscopic characteristics, mostly outcropping in the Tagus Tertiary basin, including the Madrid basin (*71*) (Figs. 2 & 3 in main text and Dataset S1):

1. Unit 53 (IGME Geode 50, Z1700) (*136*): chert appears embedded within limestones and marls containing fauna from the Lower Oligocene in the Paleogene sediments forming the sedimentary refiling of the Tagus Tertiary basin. An outcrop was identified at Huérmeces del Cerro (Guadalajara province), showing white nodular cherts with metal oxides and gypsum pseudomorphs without bioclastic remains and originated in a hypersaline continental environment.
2. Unit 167 (IGME Geode 50, Z1700) (*136-138*): nodular chert outcrop within the Intermediate Unit in the Madrid Miocene basin. Eight chert outcrops were identified within the dolomites and limestones from this unit, largely outcropping in the Upper Tagus basin, with outcrops identified near the villages of Gajanejos, El Sotillo and Romancos (Guadalajara province) and near Perales de Tajuña and Rivas-Vaciamadrid towns (Madrid province). Chert appears as nodules that can measure up to 40 cm long, with a main macroscopic originally mudstone type texture including metal oxides and gypsum pseudomorph inclusions. They mostly originated in a hypersaline continental environment. However, some outcrops revealed the presence of charophyte algae and gastropods sections, indicating that in specific places the silicifications from this formation were produced in a lacustrine sedimentary environment.
3. Unit 168 (IGME Geode 50, Z2400) (*139*): chert appears embedded in the carbonates from the Intermediate Unit in the Madrid Miocene basin. Several chert outcrops were documented close to the Manzanares and Jarama rivers, within the Madrid districts of Berrocales, Cañaveral (Vicálvaro) and Vallecas and the town of Coslada (Madrid province). These cherts present originally mudstone to wackestone macroscopic textures with only metal oxides and carbonate remains as inclusions.
4. Unit 171 (IGME Geode 50, Z2300) (*140*): chert appears embedded in the limestones from the Lower Páramo Unit, outcropping near the town of Alconada de Maderuelo (Segovia province), in the Duero basin. They originated in a hypersaline sedimentary environment, revealing an originally mudstone texture with metal oxides and lenticular gypsum pseudomorphs as inclusions.
5. Unit 184 (IGME Geode 50, Z2400) (*139*): chert appears embedded in limestones from the Miocene Intermediate Unit outcropping in the Tagus basin, near the town of Hita (Guadalajara province). These cherts originated in a hypersaline environment and show original relicts of a mudstone azoic texture, without evidence of mineral or bioclastic inclusions.

For the second main macroscopic variety, defined as lithotype 4 and composed of cherts originated in a lacustrine sedimentary environment, their inclusions and micropaleontological content allowed the differentiation of five geological units with similar macroscopic features. One is found near the Middle Duero River basin, whereas the others are located in the Midde Ebro (Figures 2 & 3 in main text and Dataset S1) (*71, 72, 141*):

1. Unit 195 (IGME Geode 50, Z2300) (*139*): in the limestones from the Arévalo formation, stratified in thin layers close to the Duero basin, nodular cherts were identified near the towns of Arévalo, Palacio de Goda (Ávila province) and Mucientes (Valladolid province). They originated in a lacustrine sedimentary environment, being the charophyte algae and the gastropods sections the main micropalaeontological content in an originally wackestone texture with metal oxides and carbonate remains as inclusions (*142*).
2. Bujaraloz – Sariñena Unit (Aagenian – Aragonian, Miocene): cherts appear embedded in carbonate levels of lacustrine origin outcropping in the south-eastern sector of the Ebro basin (*143*), near Candasnos and Peralba (Zaragoza province).
3. Torrente de Cinca – Alcolea de Cinca Unit (Chattian – Agenian, Miocene – Oligocene): chert appears embedded within carbonate deposits formed in shallow lacustrine conditions, largely outcropping in the northern and southern margins of the Ebro basin, as in the Valcuerna ravine, near the town of Peñalba (Huesca province).
4. Sierra de Lanaja – Montes de Castejón Unit (Upper Aragonian, Miocene): abundant nodular and stratified cherts appear embedded within the margo-carbonate lacustrine sediments of this unit. They possess extremely fine grains, being one of the best-quality lacustrine cherts outcropping in the Monegros region (*143*), frequently used for the production of gunflints until the 19th century (*144, 145*). Outcrops were identified in the San Caprasio mount (La Torraza), the village of Muel and La Muela (Zaragoza province).
5. Sierra de Pallaruelo – Monte de la Sora Unit (Lower Aragonian, Miocene): chert appears embedded in the margo-carbonate levels from the top of the unit, largely outcropping in Zaragoza province. We selected the outcrops of Santa Quiteria hermitage and San Borombón mount for analysis.

For the jasper silicifications of lithotype 5, most archaeological samples were similar to one single geological unit outcropping in the northeast of the Guadalajara province (Unit 155) (Figs. 2 & 3 in main text). However, the foliate preform from Level 3 attributed to this lithotype discussed above, showed a clear different fabric. This was characterized by relatively abundant dendritic blackish spots and some allogenic quartz crystals inclusions, narrow macroquartz veins with a previous white fibrous silica rim cement, and a generally well-preserved peloidal texture. As already mentioned, these features were macroscopically surprisingly similar to those of the jasperoids silicifications of carbonate sedimentary rocks from the Hettangian and Sinemurian ages (Lower Jurassic) outcropping west of the Central Massif area, in France. The main characteristics of the two units (Figs. 2 & 3 in main text and Dataset S1) are as follows:

1. Unit 155 (IGME Geode 50, Z1700) (*137*): a jasperoid silicification with nodular morphologies outcrops embedded within the limestones from El Pedregal formation (Aalenian – Bajocian, Jurassic), in the Carravilla mount near the village of Concha (Guadalajara province).
2. 'Infralias' jasperoids: different jasperoids silicifications from the Hettangian – Sinemurian (Lower Jurassic) discontinuously outcrop around the western border of the *Massif Central*

(France), in the Corrèze, Dordogne, Lot, Charente, Vienne, Indre and Cher regions (*66-68*). We selected samples recovered at the counties of Corrèze (Curemonte, Lostanges and Puy-d'Arnac outcrops), Dordogne (Clermont-d'Excideuil, Saint-Martin-de-Fressengeas and Saint-Sulpice-d'Excideuil outcrops), Charente (La Pautissie, Épenède) and Vienne (Mauprévoir), as some of them were macroscopically very similar to the archaeological samples classified as lithotype 5 and showing dendritic blackish spots. Besides these spots, the geological samples showed allogenic quartz crystals, narrow macroquartz veins with a previous white fibrous silica rim cement and a general peloidal texture. These peloids were usually not very well preserved, and deformation by contact between grains was common (Fig. 4 in main text).

Text S3. Supplementary geochemical results on chert sourcing

103 chert artefacts from Peña Capón (Table S7) were analysed by laser-ablation inductively coupled plasma mass spectrometry (LA-ICP-MS). We focused on the Solutrean and Proto-Solutrean occupations, as cherts from the Terminal Gravettian levels (5 and 6) were few and less diverse: 3 samples were analysed from Level 0, 18 from Level 1, 23 from Level 2a, 40 from Level 2b, 6 from Level 3 and 6 from Level 4. Furthermore, 7 surface samples were selected due to their macroscopic features (Dataset S3).

ACRONYM	OUTCROP / SITE	FORMATION / UNIT	AGE	TYPE	LA-ICP-MS (n)
HUE	Huérmeces del Cerro	Unit 53	Lower Oligocene	Evaporitic	15
HI	Hita	Unit 184	Vallesian – Aragonian	Evaporitic	15
GAJ	Gajanejos	Unit 167	Vallesian - Aragonian	Evaporitic	15
RO2	Romancos 2	Unit 167	Vallesian – Aragonian	Evaporitic	15
SOT	El Sotillo	Unit 167	Vallesian – Aragonian	Evaporitic	15
PERTA	Perales de Tajuña	Unit 167	Vallesian – Aragonian	Evaporitic	15
LAMA	La Marañosa	Unit 167	Vallesian – Aragonian	Evaporitic	15
RIVAS	Rivas – Vaciamadrid	Unit 167	Vallesian - Aragonian	Evaporitic	15
CA3	Cañaveral – Área 3	Unit 168	Vallesian – Aragonian	Evaporitic	15
CAHU	Cañaveral – El Humedal	Unit 168	Vallesian – Aragonian	Evaporitic	15
CA32	Cañaveral – Parcela 32	Unit 168	Vallesian – Aragonian	Evaporitic	15
CVBE	Cantera Vieja – Berrocales	Unit 168	Vallesian – Aragonian	Evaporitic	15
BEN	Berrocales Norte	Unit 168	Vallesian – Aragonian	Evaporitic	15
COSF	Coslada – S. Fernando D	Unit 168	Vallesian – Aragonian	Evaporitic	15
COSFI	Coslada – S. Fernando I	Unit 168	Vallesian – Aragonian	Evaporitic	15
VALLE	Vallecas	Unit 168	Vallesian – Aragonian	Evaporitic	15
CDP 30	CDP 30	Unit 168	Vallesian – Aragonian	Evaporitic	15
VAVI	Vallecas – Víctor	Unit 168	Vallesian – Aragonian	Evaporitic	15
ALC	Alconada de Maderuelo	Unit 171	Aragonian	Evaporitic	15
ARE	Arévalo	Unit 195	Vallesian	Lacustrine	15
PALGO	Palacio de Goda	Unit 195	Vallesian	Lacustrine	10
MU	Mucientes	Unit 195	Vallesian	Lacustrine	15
VA	Valcuerna	Cinca Unit	Chattian - Aagenian	Lacustrine	20
PC	Puente Candasnos	Bujaraloz-Sariñena Unit	Aagenian – Aragonian	Lacustrine	20
SQ	Santa Quiteria	Pallaruelo-Sora Unit	Lower Aragonian	Lacustrine	12
LT	La Torraza	Lanaja-Castejón Unit	Aragonian	Lacustrine	13
LM1	La Muela 1	Lanaja-Castejón Unit	Upper Aragonian	Lacustrine	10
LM2	La Muela 2	Lanaja-Castejón Unit	Upper Aragonian	Lacustrine	20
HOR	Campo de las Horgas	Lanaja-Castejón Unit	Upper Aragonian	Lacustrine	20
SB1	San Borombón 1	Pallaruelo-Sora Unit	Lower Aragonian	Lacustrine	11
SB2	San Borombón 2	Pallaruelo-Sora Unit	Lower Aragonian	Lacustrine	20
CAR	Loma Carravilla	Unit 155	Aalenian – Bajocian	Jasper	15
CON	Concha	Unit 155	Aalenian - Bajocian	Jasper	15
CUR	Curemonte		Hettangian	Jasper	5
PUY	Puy d’Arnac		Hettangian	Jasper	10
LOST	Lostanges		Hettangian	Jasper	10
SULP	St Sulpice d’Excideuil		Hettangian	Jasper	15
CLER	Clermont d’Excideuil		Hettangian	Jasper	15
STMAR	St Martin de Fressengeas		Hettangian	Jasper	5
LAPAU	La Pautissie		Hettangian	Jasper	15
EPE	Épenède		Hettangian	Jasper	15
MAU	Mauprévoir		Hettangian	Jasper	15
PCAP	Peña Capón	-	-	Evaporitic	47
PCAP	Peña Capón	-	-	Lacustrine	38
PCAP	Peña Capón	-	-	Jasper	18

LAU	Laugerie Haute	-	-	Jasper	4
FOU	Fourneau du Diable	-	-	Jasper	1
COM	Combe Saunière	-	-	Jasper	1

Table S7. Geological and archaeological samples analysed by LA-ICP-MS.

3.1. Evaporitic cherts

47 evaporitic cherts recovered at Peña Capón were compared with 300 geological samples from 5 geological units by means of laser-ablation inductively coupled plasma mass spectrometry (LA-ICP-MS) (Table S7). These units were located mostly in the Tagus River basin, including the Madrid Miocene basin (Unit 53, Unit 167, Unit 168 and Unit 184), but also in the Duero basin (Unit 171). After eliminating values for Be, Ca and Cr, data were automatically normalized prior to further statistical analysis by Pearson correlation. We first run a Principal Component Analysis (PCA) with all the dosed elements after eliminating those with measures below the detection limits, but no differences were observed between geological units. We therefore applied a descriptive statistical approach aimed at detecting which elements presented higher standard deviations, taking also into account the establishment of differences between geological units. After that, a Linear Discriminant Analysis (LDA) was run to observe whether it was possible to distinguish between the geological sourcing areas. As their inclusions contents were scarce, the evaporitic cherts were extremely silica pure, and hence they were very similar both at the stereoscopic microscope and the geochemical level. Therefore, the establishment of differences between sources was not easy, not even at the chemical scale. Based on the results of Mg and Ti values, we made a LDA that allowed a first discrimination between specific geological sources (Figure S26a). The resulting plot of the variability with F1 (68.58%) and F2 (31.42%) allows discrimination between several sources. There was one archaeological artefact (PC23 24) that was placed far from the main general dispersion of archaeological and geological samples and hence was discarded as an outlier. When considering the Mg and Ti amounts, the archaeological samples were mostly connected to cherts from units 53, 184 and 168, which were overlapping. The geological units 167 and 171 were far from any archaeological sample, thus suggesting that none of the studied archaeological cherts came from these two formations. This absence could be due to the large amount of opaline amorphous textures in these cherts (*71*), which could have compromised the knapping process.

Based on these results, we plotted all the geological samples from units 53, 168 and 184, together with the evaporitic archaeological samples, on a scatterplot showing Ln B/Mg vs Ln Ti/Mg, (Figure S26b). Again, artefact PC23 24 was revealed as an outlier, as its Ti/Mg ratio was significantly higher than those of the whole archaeological and geological sets. Despite the three geological units were slightly overlapped, it was possible to foresee differences between them and thus directly connect many of the archaeological evaporitic samples with a specific formation. Thus, our results show that a significant number of artefacts discarded at Peña Capón throughout the sequence of human occupations came from the regional outcrops of Huérmeces del Cerro (Unit 53) and Hita (Unit 184), as well as from different outcrops within Unit 168, mostly outcropping in the Madrid Miocene basin (Figs. 2 and 6 in main text, Dataset S3). Due to the small differences observed at the geochemical level between outcrops from Unit 168, it was not possible to present a specific scatterplot distinguishing outcrops of this unit, as all of them show an extremely similar chemical signal that does not allow a clear discrimination.

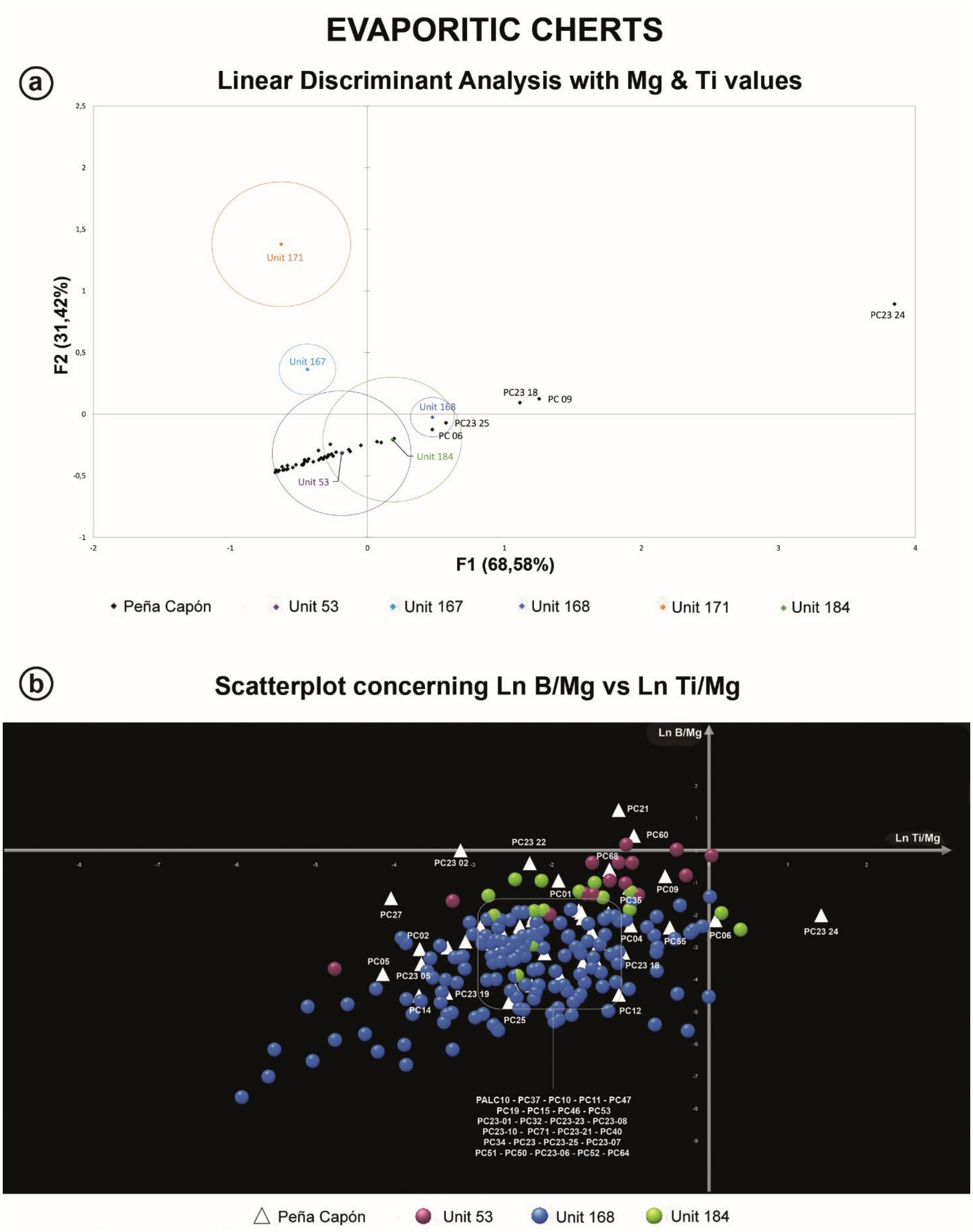

Fig. S26. **a:** Linear Discriminant Analysis (LDA) with the evaporitic cherts considering Mg and Ti values separated by units (for the geological units / outcrops only the centroids are represented, with 95% ellipse for confidence interval). **b:** Scatterplot concerning Ln B/Mg vs Ln Ti/Mg, including all the evaporitic geological and archaeological samples.

3.2. Lacustrine cherts

38 archaeological lacustrine cherts macroscopically attributed to lithotype 4 were compared with 146 geological samples from 5 geological units located in the Duero basin (Unit 195) and the Middle Ebro basin (Bujaraloz-Sariñena Unit, Cinca Unit, Lanaja-Castejón Unit and Pallaruelo-Sora Unit) through the chemical signature obtained by LA-ICP-MS. A first LDA was performed to observe if differences between geological units existed. Thus, B and U values were selected, as these elements allowed a clear discrimination between sources. In the resulting plot, with 100% of the variability represented (F1: 66.33% and F2: 33.67%), the lacustrine cherts from the Duero basin (Unit 195) were clearly separated from the other sources, and most of the archaeological samples were placed near the dispersion area of these cherts. However, up to 10 of the archaeological cherts were placed closer to the dispersion area of the Middle Ebro Basin clusters, mostly fitting within the Lanaja-Castejón and Pallaruelo-Sora units (Figure S27). Two artefacts must be considered as outliers (PC38 and PALC02), whereas the geological cherts from the Bujaraloz-Sariñena Unit and the Cinca Unit were placed far from the archaeological artefacts and were discarded as potential sources.

Scatterplots with specific trace elements were calculated for cross-checking the results of the LDA analysis (Figure S28a, b). We selected B, Mg, As and U values, as these elements allowed the best discrimination between sources. In the first scatterplot (Figure S28a), which included all the archaeological artefacts and the suitable geological samples as shown by the LDA, Unit 195 was clearly discriminated, whereas there was a small overlapping between some of the Pallaruelo-Sora and Lanaja-Castejón dispersion areas. The archaeological samples were mostly placed in the dispersion area of the Lanaja-Castejón and Pallaruelo-Sora units on the one hand, and the Unit 195 on the other. However, there was a group of artefacts that did not fit with any of the geological units. The proximity of these samples to Unit 195 probably shows that these artefacts were sourced from an outcrop from this unit not yet found and analysed.

Finally, the scatterplot was re-coloured by outcrops with the aim of connecting the archaeological samples to specific outcrops (Figure S28b). This showed a robust connection between a group of archaeological samples and the Mucientes outcrop from Unit 195, while the Palacio de Goda and Arévalo outcrops from the same unit do not show any clear relation to any sample. Similarly, for the Lanaja-Castejón Unit, while the La Torraza outcrop can be discriminated and shows no relation to any archaeological artefact, the remaining three outcrops in this unit, La Muela 1, La Muela 2 and Campo de las Horgas, are not only quite similar to each other (and hence geochemically indistinguishable), but also to several archaeological samples, as shown by their dispersion areas. Likewise, samples from the Pallaruelo-Sora Unit are quite homogeneous, and it is not possible to discriminate between the San Borombón 1 and San Borombón 2 outcrops, both matching the dispersion areas of several archaeological artefacts. However, Santa Quiteria outcrop is discarded as a potential source, as it does not fit with any of the archaeological artefacts. Therefore, our results show that the sourcing areas for the lacustrine cherts (lithotype 4) discarded at Peña Capón are found at the Mucientes outcrop (Unit 195) in the Duero Basin –and probably in other outcrops from this unit not yet identified– and at the Lanaja-Castejón Unit (La Muela 1, La Muela 2 and Campo de las Horgas outcrops) and Pallaruelo-Sora Unit (San Borombón 1 and San Borombón 2 outcrops), in the Middle Ebro Basin. To date, for the Upper Palaeolithic the Mucientes chert had only been recorded in the Côa Valley (*17, 18, 142*).

LACUSTRINE CHERTS

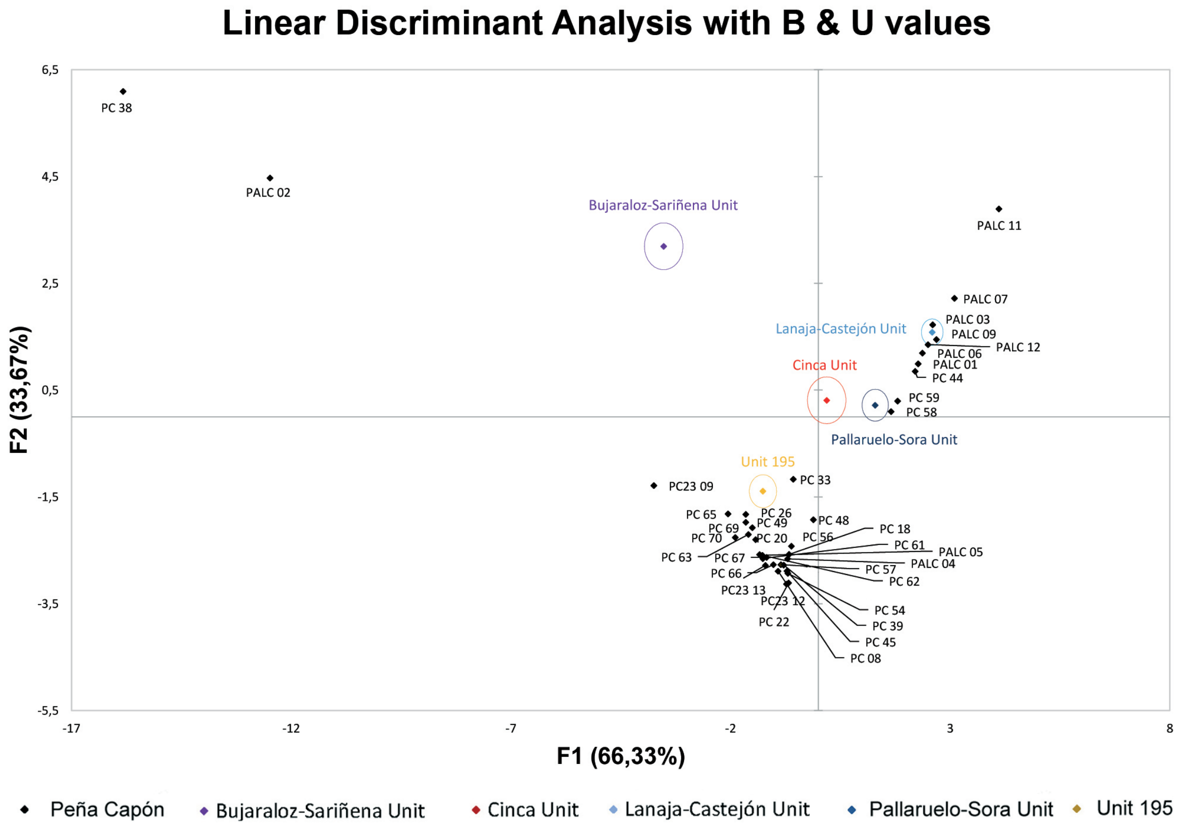

Fig. S27. LDA with the lacustrine cherts concerning B and U values separated by geological units).

The geochemical results on jasperoid cherts are included at length in the main text.

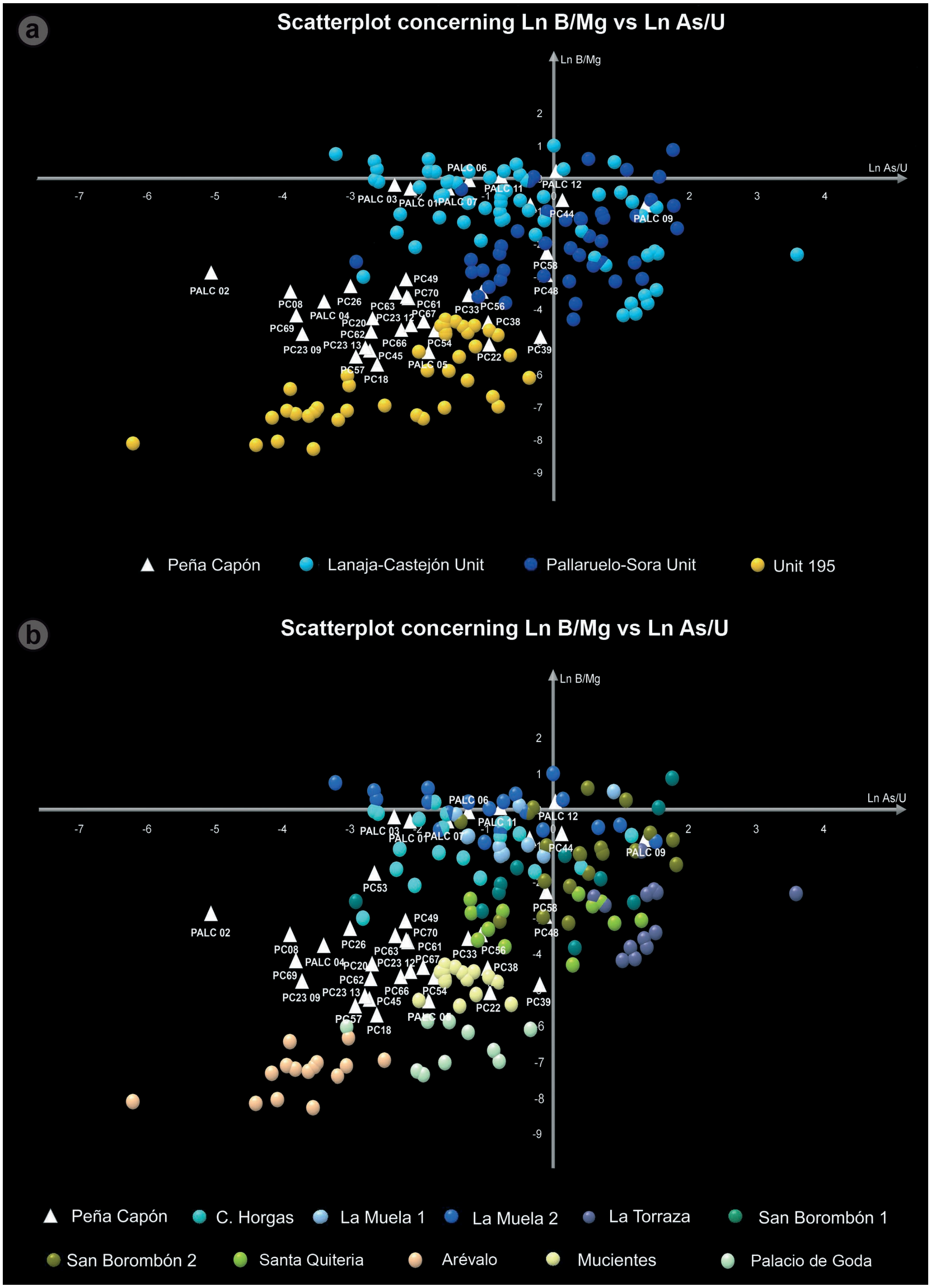

Fig. S28. **a**: Scatterplot with the lacustrine cherts concerning Ln B/Mg vs Ln As/U including all the geological units. **b**: Same as **a**, separated by outcrops. For the geological units/outcrops only the centroids are represented, with 95% ellipse for confidence interval.

Text S4. Supplementary methods and results of Least cost analysis and interpretation of lithic procurement

Based on the hypothesis that the raw materials of the artefacts discarded at a hunter-gatherer site were sourced by their makers in their exploitation area, most probably as part of their embedded procurement strategies (*5, 50, 52*), Least Cost Analysis (LCA) offers an increasingly established method of connecting discard locations of artefacts to their potential sources (*17, 18, 100, 103, 146, 147*). Usually aimed at inferring mobility and territoriality strategies of human groups, LCA is based on GIS algorithms and accounts for the uneven character of the landscape, including obstacles such as hills, rivers and other topographic features. Thus, despite known limitations (*103, 104, 148, 149*), LCA remains useful for exploring the dynamics of population movements in past hunter–gatherer landscapes and assessing the time and physical effort that the access to resources might have entailed.

Though we do not assume here that all the outcrops and geological units indicated by the geochemical analysis as sources for the cherts discarded at Peña Capón were exploited directly by their users as a result of direct catchment, we used LCA as a tool for establishing a quantitative spatial reference between the site and the sources. Likewise, it is unrealistic to suppose that groups would travel many hundreds of kilometres along strict LCPs, especially when points of attraction, like food sources, water, human settlements or other raw materials, are located at intermediate points (*149, 150*). Hence, our LCA is intended to provide a quantitative estimation of the time and physical effort separating the outcrops from the discard location (Peña Capón) without assuming a specific relationship or a single route connecting them.

We calculated Least Cost Paths (LCPs) and anisotropic (accounting for uphill or downhill direction) travel times from Peña Capón to all the confirmed outcrops and geological units, 7 in Spain, and two (Saint-Sulpice-d'Excideuil and Mauprévoir) in France, according to the workflow described in the methods section. In Table S8 we show the individualized results for all of them (see also Dataset S4 and Fig. 6 in main text).

OUTCROP	FORMATION	AGE	TYPE	Travel time from Peña Capón (seconds)	LCP method	Distance (m)
Hita	Unit 184	Vallesian - Aragonian	Evaporitic	15952.06575	anisotropic naismith	20632.4
					llobera_sluckin	20075.39

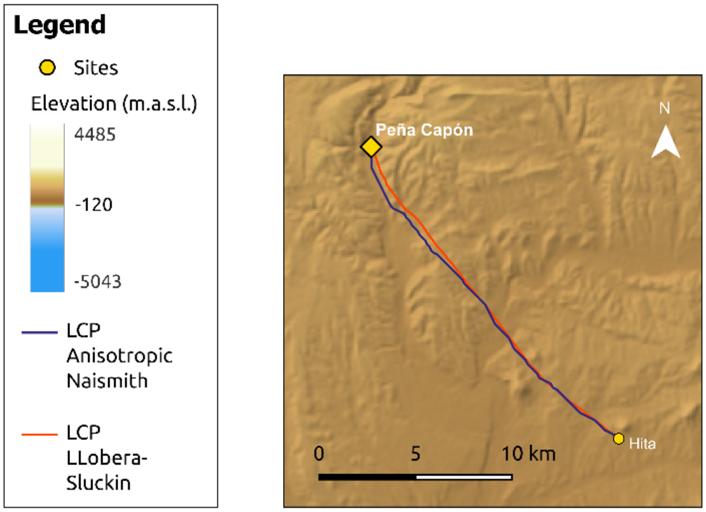

OUTCROP	FORMATION	AGE	TYPE	Travel time from Peña Capón (seconds)	LCP method	Distance (m)
Huérmeces del Cerro	Unit 53	Lower Oligocene	Evaporitic	30813.89083	anisotropic naismith	38455.11
					llobera_sluckin	37605.37

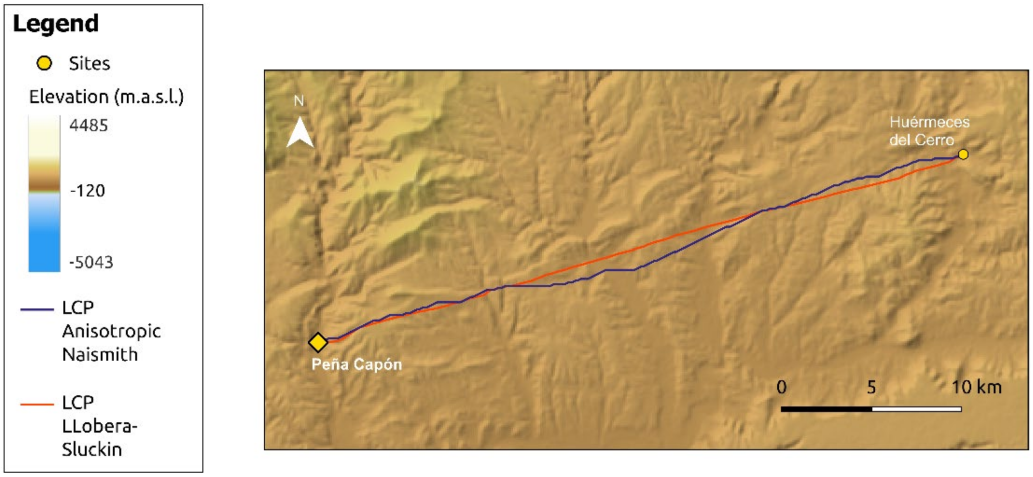

Table S8. Least Cost Paths from Peña Capón to all the outcrops and geological units confirmed by the LA-ICP-MS to be sourced for the lithics discarded at the site.

OUTCROP	FORMATION	AGE	TYPE	Travel time from Peña Capón (seconds)	LCP method	Distance (m)
Unit 168 mid	Unit 168	Vallesian - Aragonian	Evaporitic	53013.06316	anisotropic naismith	71853.8
					llobera_sluckin	70955.33

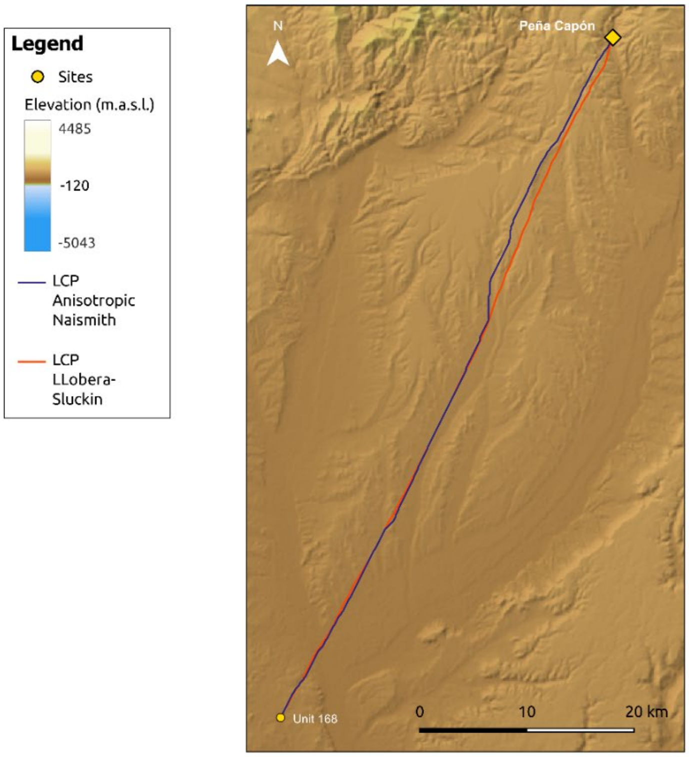

OUTCROP	FORMATION	AGE	TYPE	Travel time from Peña Capón (seconds)	LCP method	Distance (m)
Unit 155 mid	Unit 155	Aalenian - Bajocian	Jasper	84869.44278	anisotropic naismith	106610.5
					llobera_sluckin	105124.6

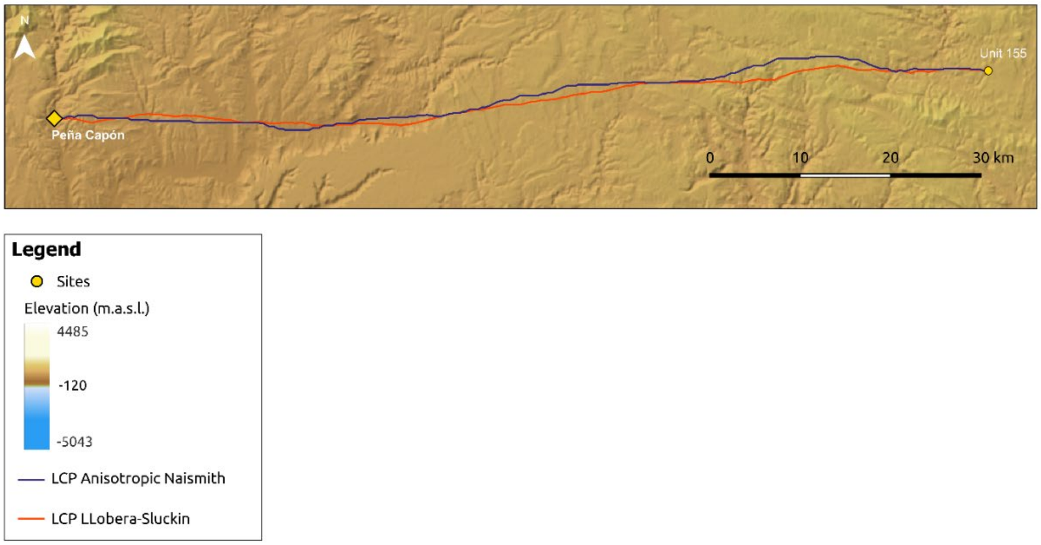

Table S8. (continued).

OUTCROP	FORMATION	AGE	TYPE	Travel time from Peña Capón (seconds)	LCP method	Distance (m)
Mucientes	Unit 195	Vallesian	Lacustrine	125440.1736	anisotropic naismith	161744
					llobera_sluckin	159018.1

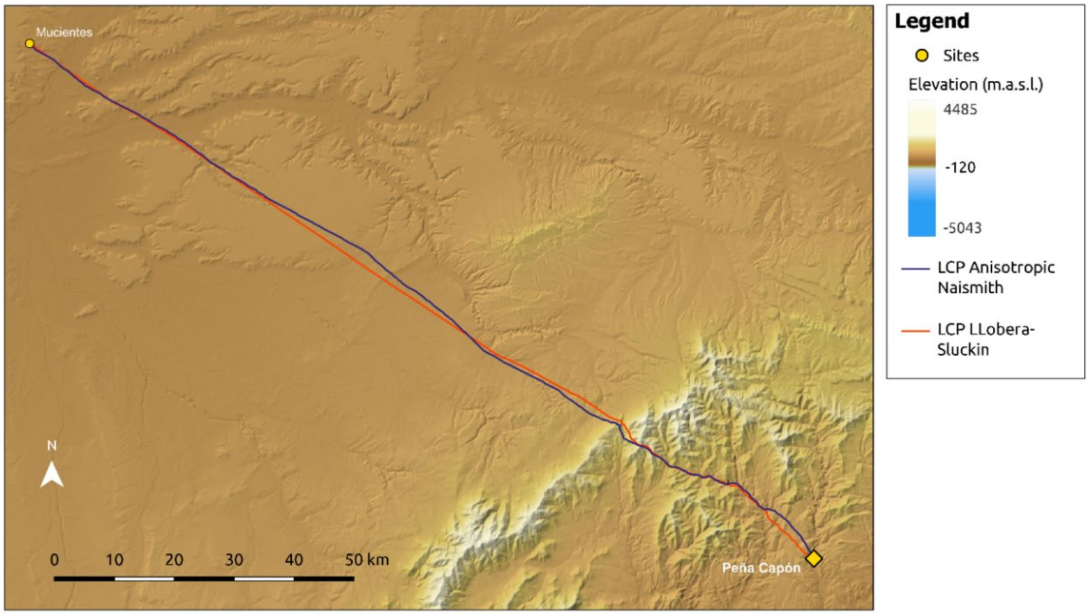

OUTCROP	FORMATION	AGE	TYPE	Travel time from Peña Capón (seconds)	LCP method	Distance (m)
Lanaja-Castejón mid	Lanaja-Castejón Unit	Upper Aragonian	Lacustrine	149784.131	anisotropic naismith	194618.9
					llobera_sluckin	189804.2

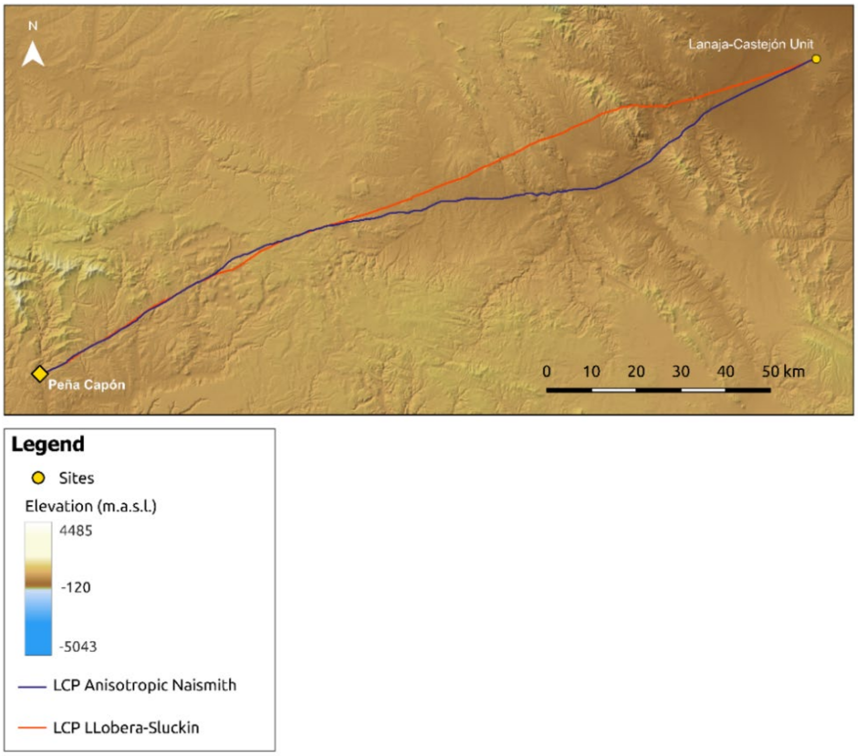

Table S8. (continued).

OUTCROP	FORMATION	AGE	TYPE	Travel time from Peña Capón (seconds)	LCP method	Distance (m)
Pallaruelo-Sora mid	Pallaruelo-Sora Unit	Lower Aragonian	Lacustrine	152196.7636	anisotropic naismith	197107.2
					llobera_sluckin	192091.8

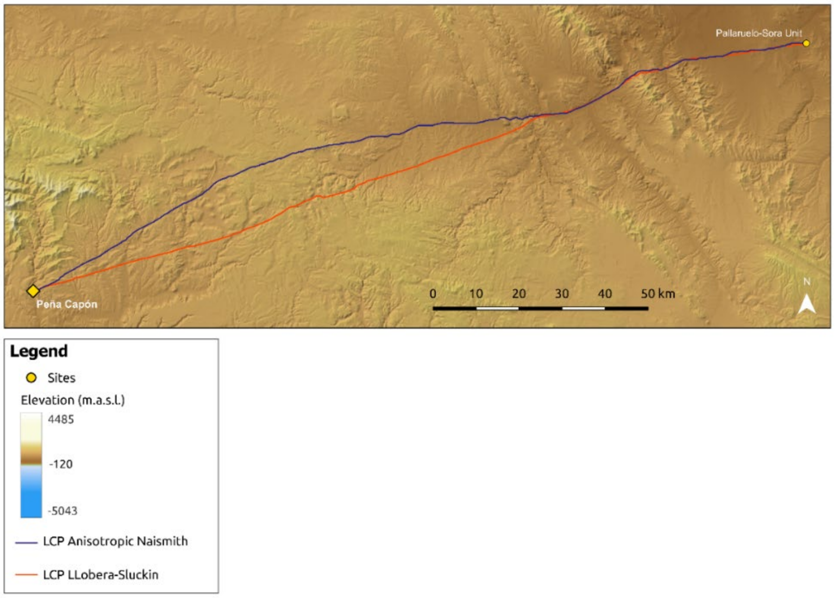

OUTCROP	FORMATION	AGE	TYPE	Travel time from Peña Capón (seconds)	LCP method	Distance (m)
St Sulpice d'Excideuil	Infralias jasperoids	Hettangian - Sinemurian	Jasper	474942.8121	anisotropic naismith	620209.1

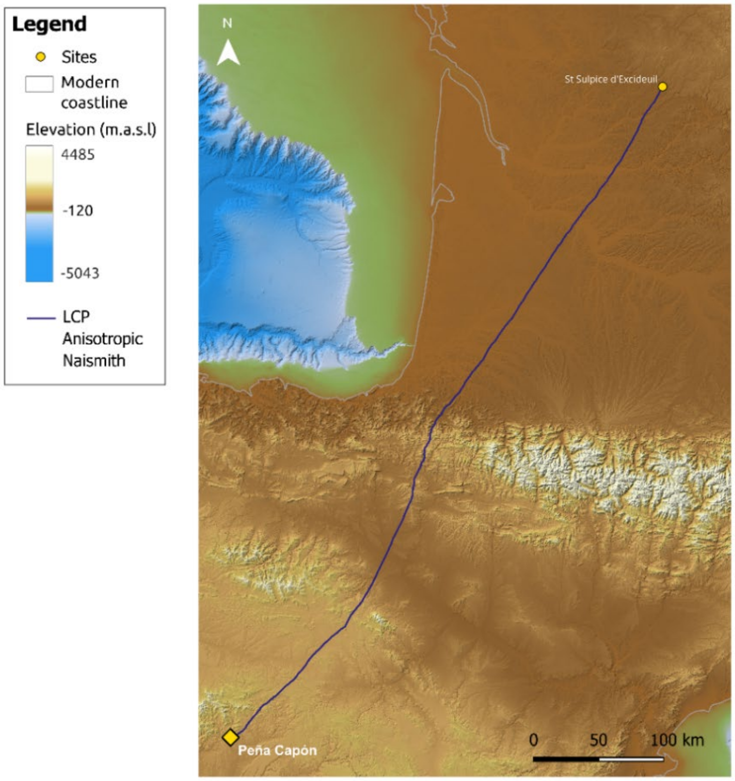

Table S8. (continued).

OUTCROP	FORMATION	AGE	TYPE	Travel time from Peña Capón (seconds)	LCP method	Distance (m)
Mauprévoir	Infralias jasperoids	Hettangian - Sinemurian	Jasper	512728.3251	anisotropic naismith	671670.3

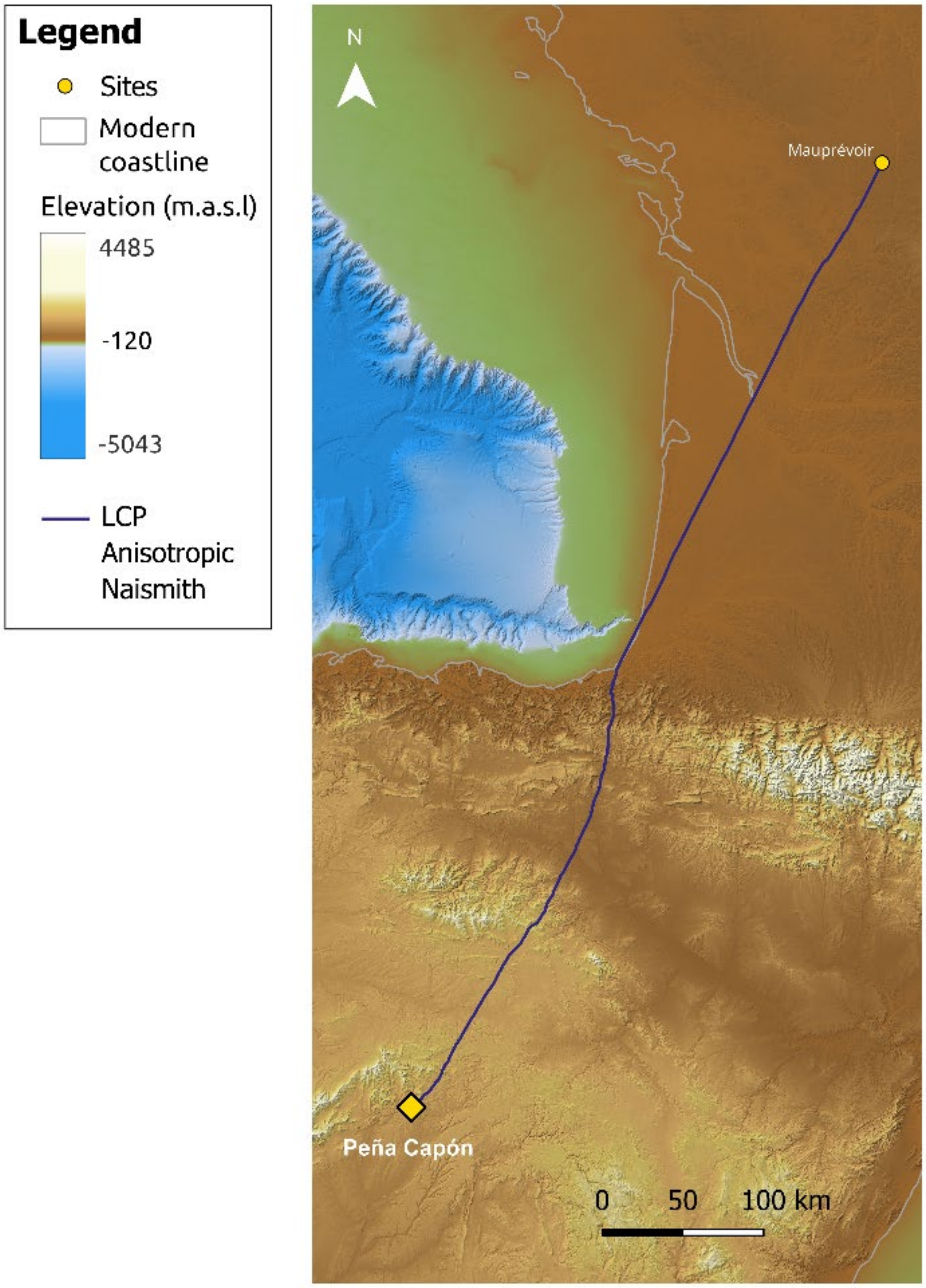

Table S8. (continued).

As for the classification of the outcrops/units in terms of their spatial relation to the discard location of the lithics, there is no consensus in the literature as to what are 'local', 'regional', or 'exogenous' rocks. Several researchers have proposed classifications based on parameters such as the Euclidean distance between the source and the discard location, the potential home range of the human groups, the quantity of the lithic artefacts and their classification within the *chaîne opèratoire* sequence, the knapping quality of the raw materials, risk mitigation, and the time and effort needed to walk between the two points (*5, 16, 45, 47, 54, 151-153*). Here we first focus on this last aspect, determined using Least Cost Analysis. In so doing, we consider the ethnographic evidence showing that hunter-gatherers rarely forage for more than eight hours a day (and usually less), being the other 16 hours for sleeping, resting, socializing and leisure (*154*) [see Table 1-1 in (*5*) for a compilation of data]. Thus, rocks that can be acquired in less than eight hours, including a round-trip from the site to the source, are considered local –or immediate if they can be accessed in less than 2 hours. For those rocks needing more than one day to be procured, we use the categories of 'regional', 'exogenous', 'exotic' and 'ultra-exotic' depending on the time necessary for their potential direct procurement (Table S9; Fig. 6 in Main text). Naturally, besides travel and resting time, an additional time should be considered for surveying the outcrops and extracting the rocks, including on-site quality testing and probably developing the initial knapping. However, given the high variability of factors affecting the time needed for these actions, depending on the needs of the human groups, their previous knowledge, and the nature and accessibility of the outcrops, we did not attempt to quantify these variables. In any case, the time limits between the 'exogenous', 'exotic' and especially 'ultra-exotic' categories cannot be considered in absolute terms, as all of them, and especially the latter two, are conceived in the framework of social exchanges and not direct procurement (see below). Therefore, in these cases the time needed for directly accessing the outcrops traveling from the site is not so relevant in behavioural terms.

Round-trip time from site to source, in hours	Including resting time	Classification
Less than 2 hours	Less than 2 hours	Immediate
Between 2 and 8 hours	Between 2 hours and 1 day	Local
Between 8 and 24 hours	Between 1 and 3 days	Regional
Between 24 and 56 hours	Between 3 and 7 days	Exogenous
Between 56 and 168 hours	Between 1 and 3 weeks	Exotic
More than 168 hours	More than three weeks	Ultra-exotic

Table S9. Classification of rocks according to the time needed for their potential direct procurement from their discard location and going back.

Determining whether the rocks coming from a given outcrop were [1] directly procured by the groups responsible for their discard, either as part of embedded procurement or as a result of special-purpose trips, or [2] were the result of exchange or down-the-line trade, is a difficult task (*5, 14, 16, 17, 51, 54, 73, 87*). Our working hypothesis is that the presence at a site of exogenous and exotic rocks is best explained as the result of exchange or trade, and not of direct or embedded

procurement. The main argument for this is that two- or even three-days foraging trips are rare but still relatively frequent in the ethnographic record, while longer, special-purpose trips are very rare and do not account for the risk-minimizing strategy characterizing the hunter-gatherer lifeways (*5, 51, 52, 154*), including lithic procurement by highly mobile groups, and especially when other good quality raw materials are found closer (*37, 54*). Likewise, when considering annual range movements, the longest documented straight-line distances covered by foraging trips very rarely exceed 150 km, with few examples reaching up to 250 km, and only one extreme case reaching 400 km, as shown by the caribou hunting trips of the Chipewyan from west of Hudson's Bay (North America) (*37, 51*). Moreover, most of such trips (intended for acquiring food rather than lithic resources) use means of transportation, such as dog sleds and watercrafts, and occur only in sub-arctic environments.

Text S5. Supplementary results on social networks and lithic raw material circulation

In all archaeological levels from Peña Capón subject to macroscopic, geochemical and Least Cost analyses, we have documented, together with a large number of regional cherts, the presence of exogenous, exotic and ultra-exotic rocks (Text S2.1. and Dataset S3). This points to the existence of exchanges of lithic raw materials during Proto-Solutrean, Middle Solutrean and Upper Solutrean times, connecting Central Iberia with different regions of the Tagus, Ebro and Duero basins and, in the case of the Middle Solutrean, also with Southwest-Central France (Fig. 6 in main text). This pattern strongly suggests the existence of long-distance contacts, and hence of geographically broad social networks, maintained during at least 3,000 years. In the case of the Middle Solutrean an especially large network was active during ~1,400 years, as it involved the largest distance between a lithic source and its discard location documented in Western Europe. The existence of these broad social networks is demonstrated by [1] the least-cost distance between the discard location and the source of the exotic and ultra-exotic rocks, [2] the time potentially needed to directly access the outcrops and return to the site, [3] the quantity and techno-typological classification of the exotic and ultra-exotic rocks within the operational chain of lithic production, and [4] the size of the area defined by the sources of all rocks discarded on each occupation recorded at Peña Capón.

Points [1] and [2] have been already discussed in Text S4 (see also Dataset S4). With regards to point [3], the exotic and ultra-exotic rocks discarded at the site, and especially the Hettangian jasperoid cherts, account for a very limited quantity of the whole assemblage (Text S2.1., Dataset S3 and Main text) and are composed of either retouched tools or small flakes, including the preform of a Solutrean laurel leaf point from Level 3 (Fig. 1 in main Text), thus supporting exchange as the most likely process explaining their presence at the rock shelter (*16, 19*). Furthermore, although the exotic lacustrine cherts from the Middle Ebro valley and, to a lesser extent, Mucientes, are amongst those with the highest knapping aptitudes of all the studied assemblages, the jasperoid cherts from Southwest France present lower knapping quality than other exploited rocks whose sources are closer to Peña Capón, such as the jaspers from Unit 155 or the mentioned lacustrine rocks. Hence, it is very unlikely that the human groups exchanged these rocks due to their knapping quality, and it is even less likely that they travelled almost 700 km for their direct procurement. To estimate the overall knapping aptitude of a rock we considered its general texture, grain size and homogeneity, as well as the absence or presence of microfractures and fissures and the size of the nodules (*71*).

Another alternative reason that could explain the presence of ultra-exotic rocks at Peña Capón would be a colonization process, as some of the largest raw material displacements have been reported during such processes (*37, 155*). However, besides the fact that Hettangian jasperoids are found throughout three archaeological levels at Peña Capón, spanning ~1,400 years, there is no evidence supporting any colonization process or population migration during the early or middle phases of the Solutrean from France to Iberia. The prevailing consensus sees the Solutrean as rooted in western European Late Gravettian and Proto-Solutrean technologies (*31, 64, 116, 130, 156, 157*) and the search for a single Solutrean origin is not a scientific debate anymore (*64*). In fact, although the Solutrean most probably emerged in a very rapid fashion with no significant chronological differences across the whole extension of the technocomplex, current chronometric data suggest that the Iberian occurrences are slightly older (*59, 127, 157-159*), being Peña Capón level 3 among the oldest examples of Middle Solutrean technologies (*59*) (Text S1).

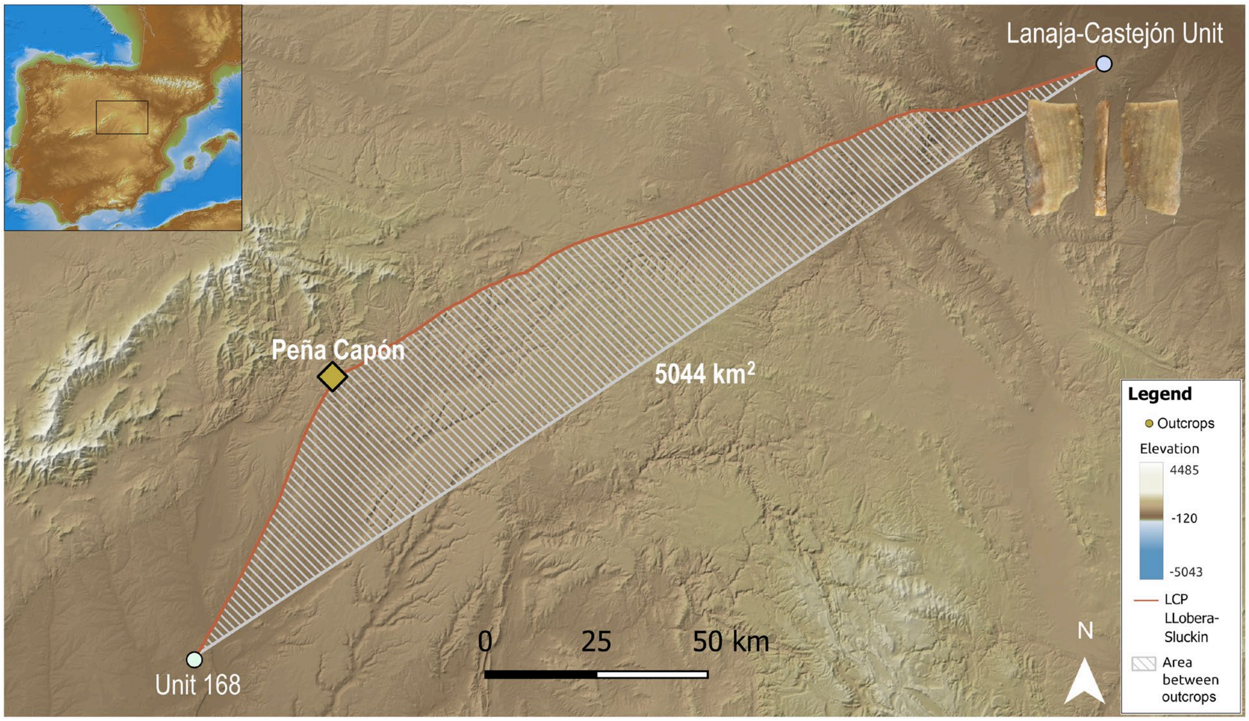

Figure S29. Maximum area defined by the sources of lithic raw materials (as indicated by LA-ICP-MS) discarded at Peña Capón Level 0. A representative lithic artefact is shown for each source.

With respect to the size of the areas defined by the sources of rocks discarded on each Peña Capón level (point 4 above), if we compare such areas (Figs. S29-S34) with the annual land-use ranges of ethnographic hunter-gatherers, the outcome is clear. There is not a single hunter-gatherer group in the ethnographic record (*5, 51*) whose annual home range even approaches the sizes for the areas defined in levels 2a, 2b and 3 of Peña Capón, where Hettangian jasperoids are present. Not even highly mobile subarctic foragers, such as the Baffinland Inuit, who might cover an annual territory of around 25,000 km^2 [or even less, according to Ellis (37): note 1], are close to the 57,405 km^2 indicated by data of Peña Capón Level 3, the 80,739 km^2 of Level 2a, or the 88,995 km^2 of Level 2b (Fig. S35). Areas for Level 1 and Level 4, 24,555 km^2 and 21,940 km^2 respectively, are in the range of the mentioned subarctic hunter-gatherers, but still above the highly mobile Nunamiut, which may cover a range of up to 20,500 km^2 in some cases (*160*). The smallest area is recorded in the Upper Solutrean of Level 0, with 5,044 km^2, which is, however, well above most of the ranges covered by many ethnographic hunter-gatherers, including sub-artic, tropical forests, boreal forests and temperate desert groups. In Figure S35 we show a selection of the annual land-use areas covered by these groups, based on the compilation of data provided by Kelly [(*5*): table 4-1]. This figure clearly illustrates that the areas defined by the location of the raw material sources discarded in Levels 2a, 2b, and 3 of Peña Capón (and probably also those derived from Levels 1 and 4) are too large to be considered part of the annual home range of the human groups settled at the rock shelter during the Middle Solutrean [see (*17*) for a similar reasoning concerning raw materials exploited during the Upper Palaeolithic in the Côa Valley, Portugal].

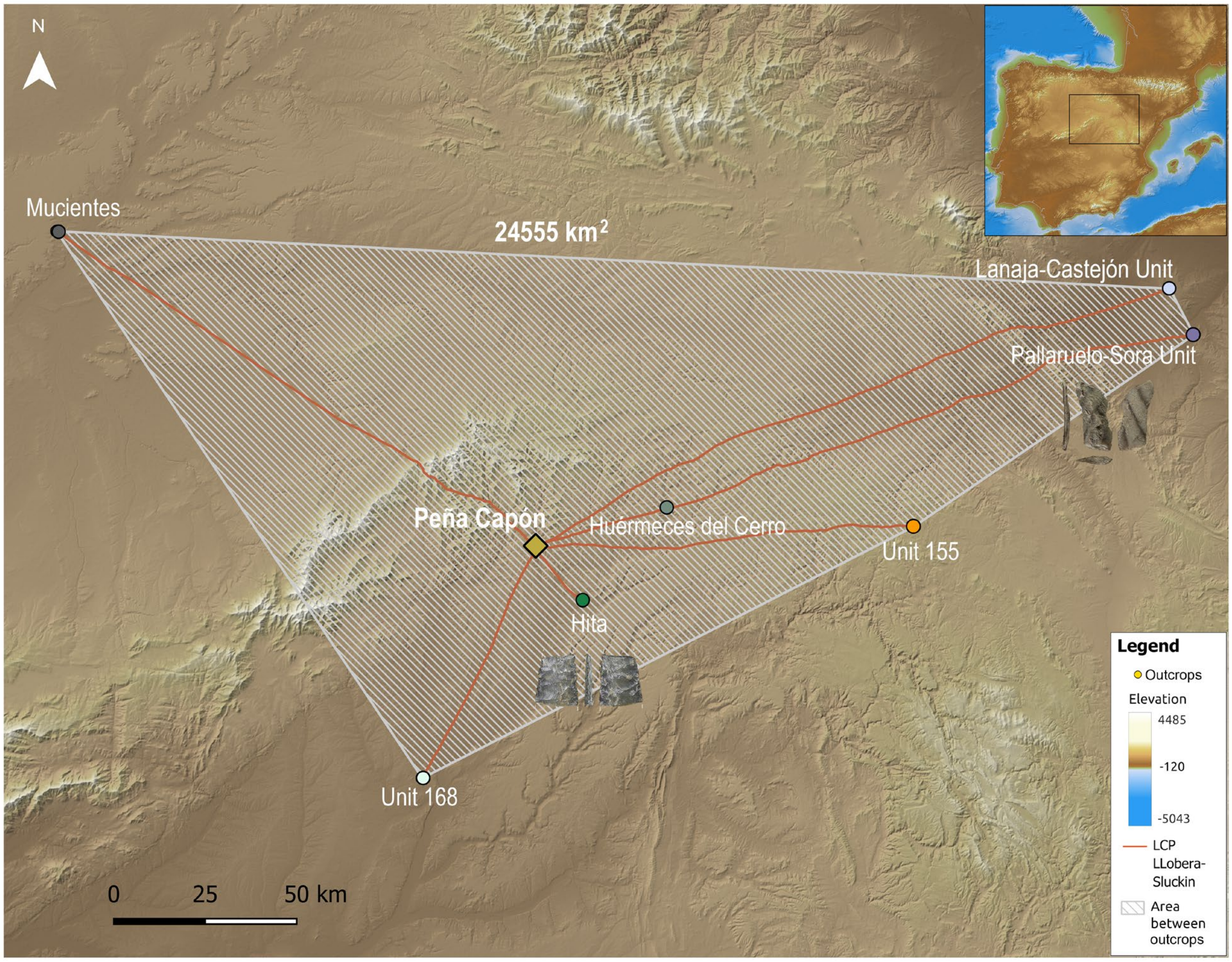

Figure S30. Maximum area defined by the sources of lithic raw materials (as indicated by LA-ICP-MS) discarded at Peña Capón Level 1. A representative lithic artefact is shown for each source.

In any case, we should bear in mind that these areas are conservative calculations, as they only consider lithic objects that have been analysed by LA-ICP-MS. While the analysed samples were large enough to be representative (Text S3 and Dataset S3), they did not include all the assemblages subject to macroscopic study (not to mention all the excavated assemblages), and hence it is possible that some exotic or ultra-exotic sources are yet to be confirmed by further studies. Likewise, although in some levels (0, 2a, 3 and 4) the regional evaporitic cherts of Hita and Huérmeces del Cerro have not been documented by the geochemical analyses (as they were not specifically targeted), in all of them these cherts account for most of the exploited raw materials according to the micropalaeontological and textural study (Text S2.1.). Yet, for the calculation of areas and the representation of the raw material sources (Figs. S29-S34), we considered only the results confirmed by LA-ICP-MS, as only the most robust data were retained.

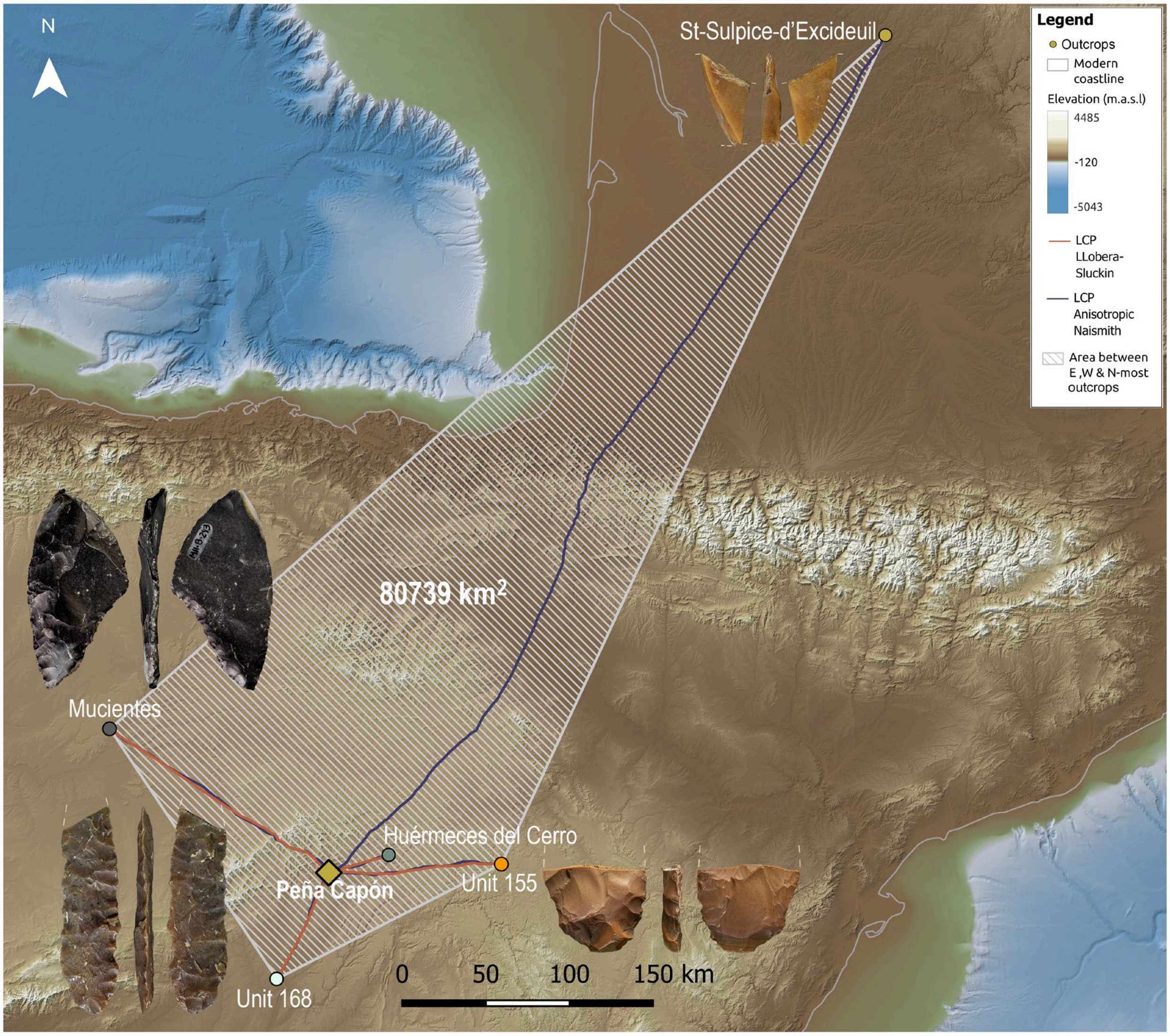

Figure S31. Maximum area defined by the sources of lithic raw materials (as indicated by LA-ICP-MS) discarded at Peña Capón Level 2a. A representative lithic artefact is shown for each source.

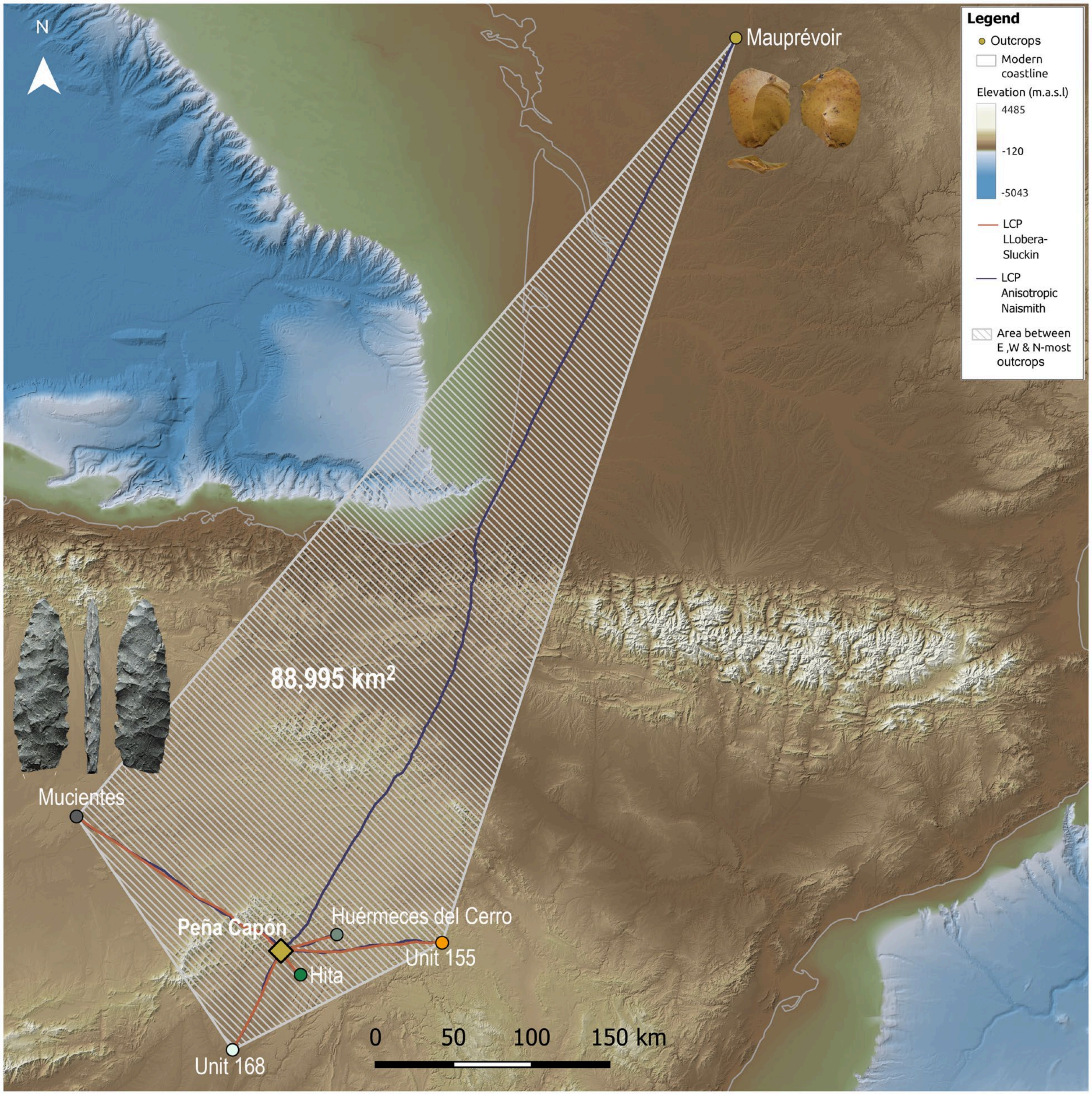

Figure S32. Maximum area defined by the sources of lithic raw materials (as indicated by LA-ICP-MS) discarded at Peña Capón Level 2b. A representative lithic artefact is shown for each source.

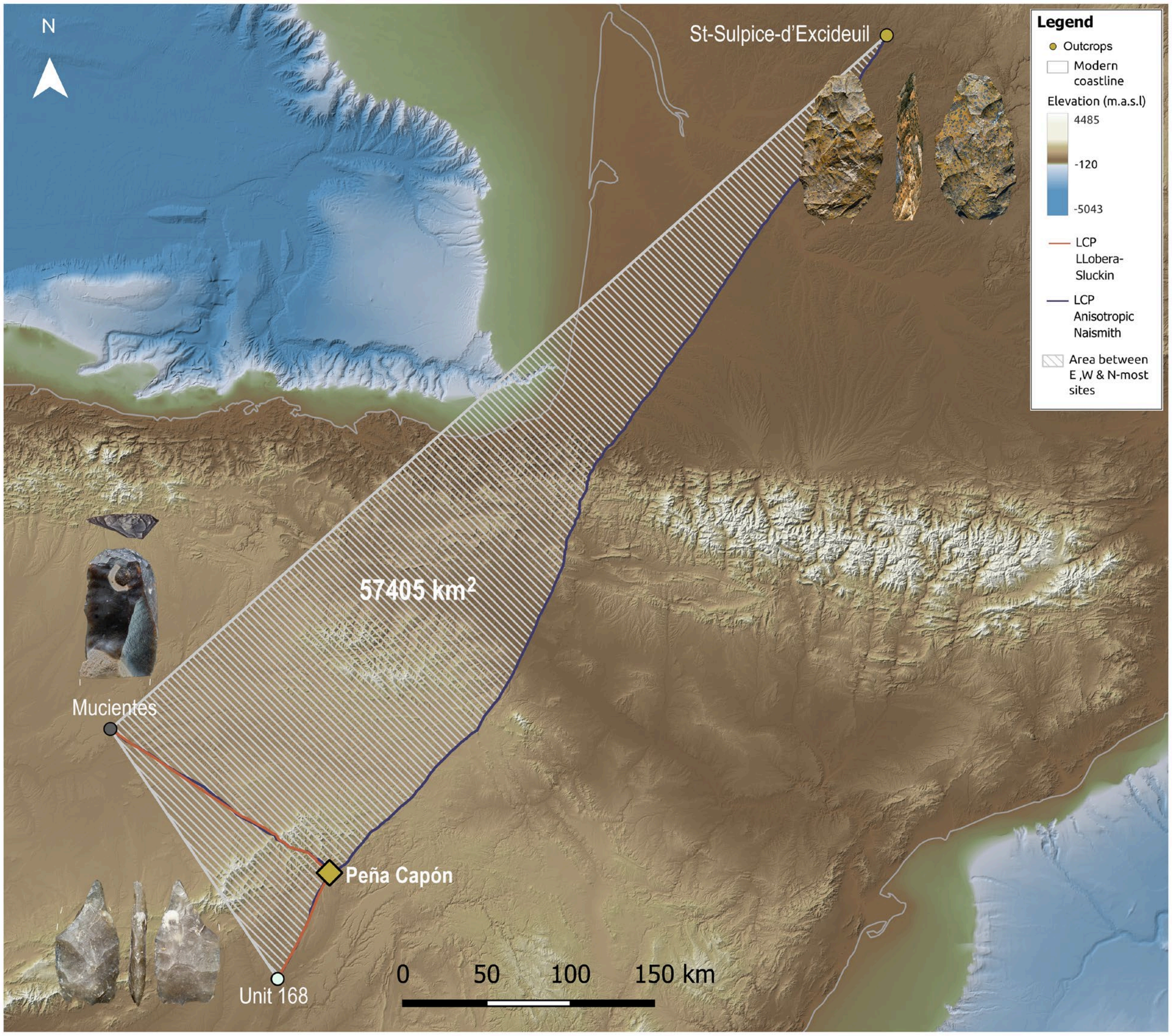

Figure S33. Maximum area defined by the sources of lithic raw materials (as indicated by LA-ICP-MS) discarded at Peña Capón Level 3. A representative lithic artefact is shown for each source.

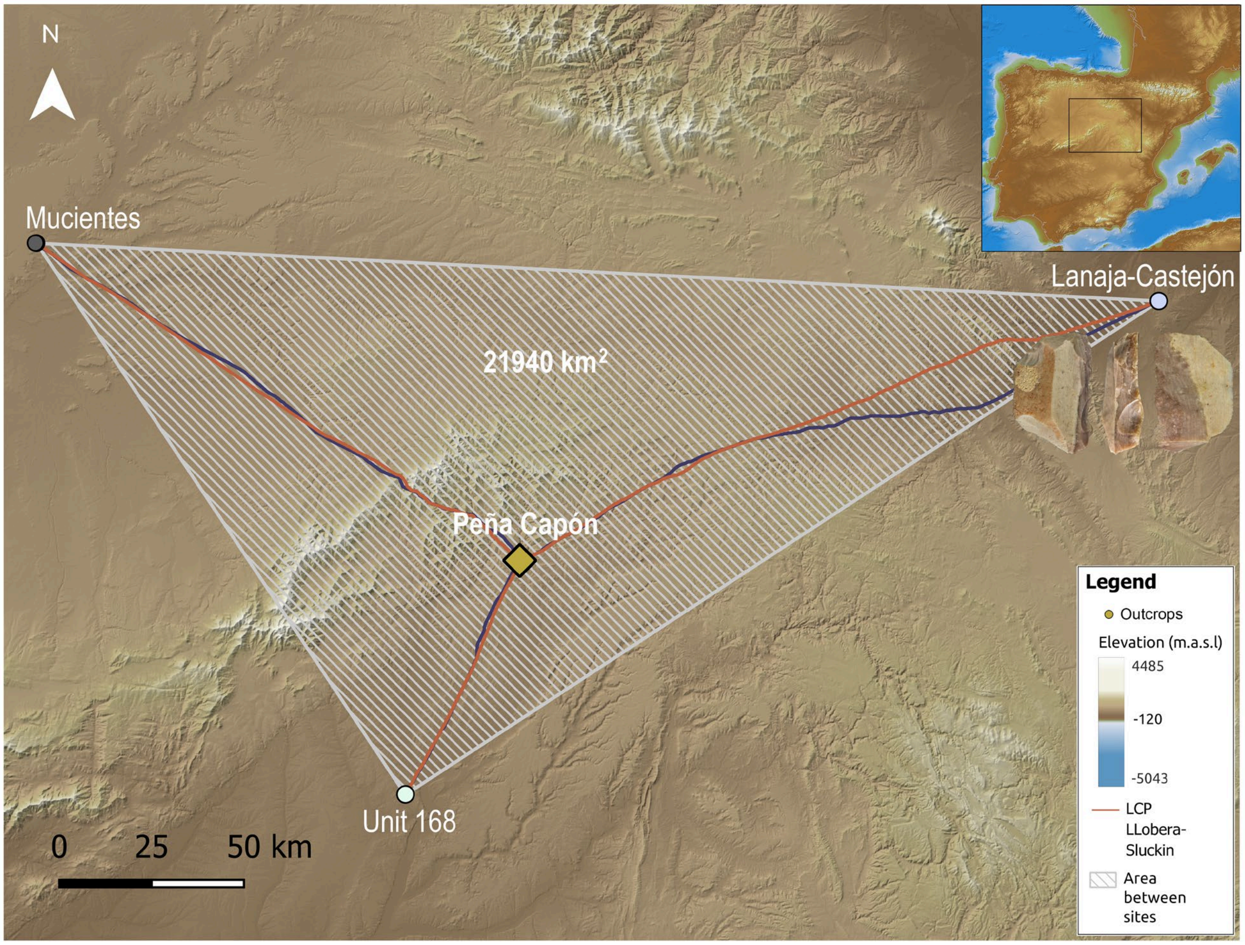

Figure S34. Maximum area defined by the sources of lithic raw materials (as indicated by LA-ICP-MS) discarded at Peña Capón Level 4. A representative lithic artefact is shown for each source.

A secondary outcome of our study is that it provisionally confirms the classic model of a shift in territoriality patterns between the Middle and Upper Solutrean. This model sees the appearance of geographically distinctive armatures during the Upper Solutrean as a reflection of the reduction of the broad social networks operating during the Middle Solutrean, partly due to climate amelioration (*15, 64, 88*). Although this model is not shared by all scholars (*31, 63, 127*), the results obtained in our chert sourcing analyses provide further support for it. As no ultra-exotic materials have been documented in Peña Capón Level 0, it could be argued that the broad large network connecting Central Iberia to Southwest/central France during the Middle Solutrean broke-up during the Upper Solutrean (Fig. S29). However, the analysed samples from Level 0 were scarce compared to the Middle Solutrean levels (see Text S2), and hence this interpretation needs further refinement and empirical testing by future research.

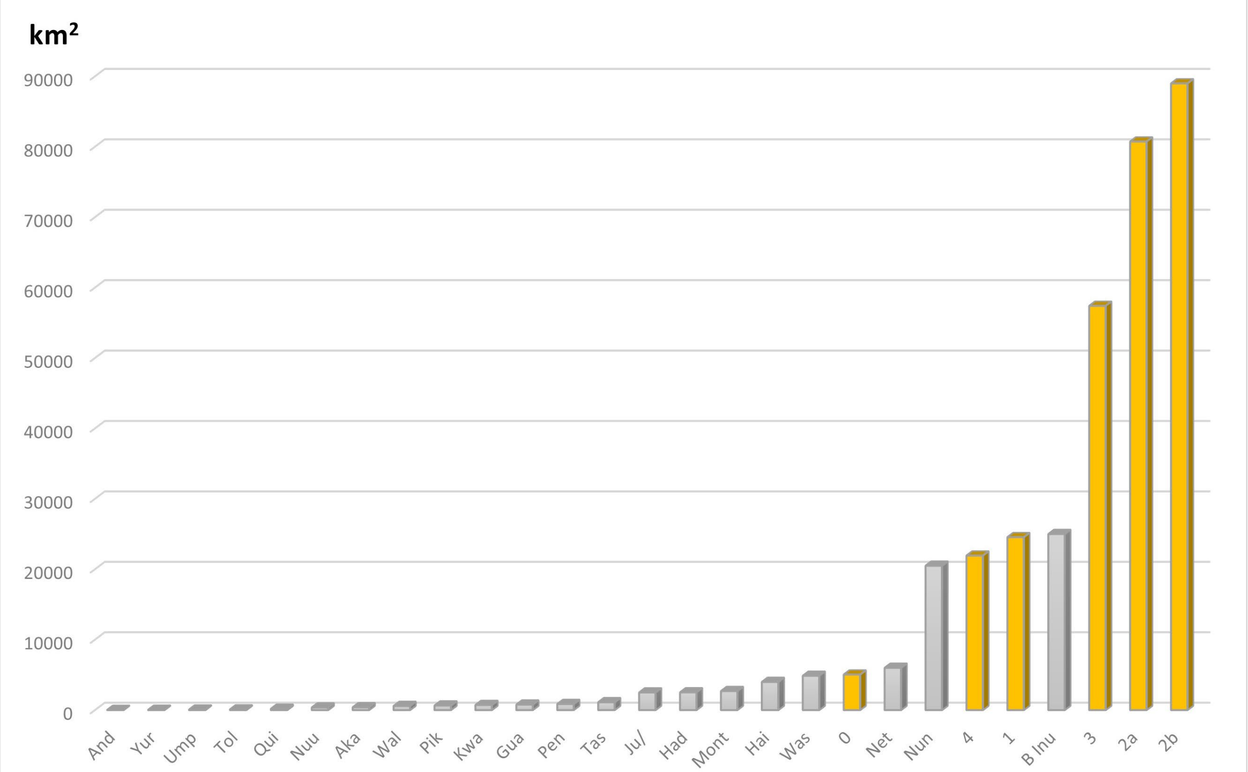

Figure S35. Comparison between ethnographic hunter-gatherers' annual home ranges and the areas defined by lithic raw material sources discarded at Peña Capón. Ethnographic data is based on different sources compiled by Kelly (*5*: Table 4-1). Peña Capón levels are highlighted in yellow. We use a representative selection of groups living in sub-artic environments, tropical forests, boreal forests and temperate deserts, including the most mobile groups. Equestrian and maritime cases are not considered. Baffinland Inuit (**B. Inu**); Nunamiut (**Nun**); Netsilingmiut (**Net**); Waswanipi Cree (**Was**); Haisla (**Hai**); Montagnais (**Mont**); Guayaki (**Gua**); Hadza (**Had**); Ju/'hoansi (Dobe) (**Ju/**); Tasmanians, Big River (**Tas**); Penan (**Pen**); Kwakwak'awakw (Ft. Rupert) (**Kwa**); Pikangikum (Ojibwa) (**Pik**); Walapai (**Wal**); Nuuchahnulth (Nootka) (**Nuu**); Aka (**Aka**); Quileute (**Qui**); Tolowa (**Tol**); Umpila (Nesbitt R., Cape York) (**Ump**); Yurok (**Yur**); Andamanese (coastal) (**And**).

A further factor in the discussion of the implications of the lithic raw material data from Peña Capón relates to the number of hunter-gatherer bands involved in the process of raw material circulation and social networking, and their distribution across the landscape. When considering the ideal sizes of hunter-gatherer groups and their exploitation areas, both archaeological and ethnographic (*5, 12, 14, 51*), the very large areas defined by the lithic sources in the Middle Solutrean levels of Peña Capón cannot be explained only by the participation of a limited number of interconnected bands. Given the geographic distances involved, the circulation network must have included multiple exchange processes between different human groups connected and organized within different social and spatial levels, participating in a down-the-line trade process (*73*) which fosters cumulative movement through multiple groups (*87, 161*). Although it is not possible to know the exact number of exchange events and the distances actually travelled by the different human groups involved, the data point to the existence of a complex network allowing the circulation of goods and information throughout a very large geographic area (*3-9, 14*). We

illustrate this with a heuristic model based on Whallon's estimates (*14*) for the spatial organization of Upper Palaeolithic hunter-gatherer bands and their territories. According to this model [see also (*162*)], forager groups are ideally organized in a first level of 'minimal' or local bands, composing 25-30 people that guarantee the presence of mates and are thus organized as a breeding unit. Based on data from the Late Upper Palaeolithic of Central and Western Europe, Whallon proposes a foraging area for this minimum band of 2,500 km^2, and he defines an ideal hexagonal spatial unit with a radius of 28 km for representing it on the landscape. Yet, it is known that forager local bands are not self-sufficient in the long term and thus cannot survive in isolation [e.g. (*74*)]. Thus, Whallon defines the second level of spatial organization as the 'maximal' or regional band, corresponding to the minimum size of a stable, long-term viable population of human foragers. Based on ethnographic data, this maximal band integrates between 475 and 570 people and is spatially modelled as a series of adjacent minimal band territories arranged in two tiers, comprising 47,500 km^2, with a radius of 123 km (Fig. S36). These numbers and sizes are in agreement with those compiled and discussed by Kelly (*5*).

Within maximal bands, human groups are integrated by the cultural mechanisms of a given cultural system, and hence individuals from different bands, but from the same regional group, are expected to interact and share information to a greater degree than individuals from different regional groups. Thus, these groups maintain strong ties and social networks across wide geographic areas, where cultural similarities are to be found. Furthermore, Whallon proposes that these maximal bands are not self-contained isolated entities either, and hence it is expected that contacts among adjacent maximal bands exist (Fig. S36, top), although these would be less frequent and mostly limited to "informational" or "non-utilitarian" mobility. However, the circulation of utilitarian goods, such as lithic raw materials, would be limited to distances within a maximal band (usually below a straight-line distance of 200 km). He also proposes that these contacts surpassing the limits of the maximal band are to be expected especially in unstable environments with major resource fluctuations, as also stressed by other authors (*21, 74*). Overall, it is proposed that multi-scalar social networks among hunter-gatherer societies operated as 'safety nets' to mitigate risk during periods of environmental stress and resource uncertainty (*14, 74*), hence enabling the transmission of social knowledge by means of 'non-utilitarian' mobility, and eventually driving and shaping cultural evolution (*21*) and cumulative culture (*75, 76*).

Naturally, Whallon's heuristic model is "constructed under the idealized assumptions of hexagonal packing of spatial units, or territories, over a perfect, uniform plane", and he observes that the "exact spatial dimensions of this model will be specific to particular cases" and hence the hexagonal territories should be "fitted over specific geographical features or topography" (*14*). Yet, he also stresses that "the proportional relationships within the overall spatial organization should remain essentially constant", and hence the proposal may be applied to other contexts as a heuristic model (*163-164*). Here we apply Whallon's heuristic model to the territory defined by the sources of the raw materials identified at Peña Capón, as indicated by the area between the farthest identified outcrops and the Least Cost Paths connecting them to the rock shelter. In so doing, we set the Peña Capón rock shelter as the central starting point of the main maximal band, and then follow the area defined by the identified lithic sources, plotting as many local (hexagons) and maximal bands (groups of 19 hexagons) as needed, always within the area defined by the outcrops (Fig. S37). Only the maximal band defined by Peña Capón is fully represented, while for the rest of the area only local bands are represented to avoid over assumptions on the distribution of foragers throughout the territory. The heuristic capacity of this method is first justified by the reasonable concordance between the local band size (single hexagon with a radius of 28 km) and

the area defined for classifying rocks as ‘local’, corresponding to a maximum of 8-hours round trip from the site, in turn conceived as a measure for the limits of the foraging area or exploitation range of the human groups.

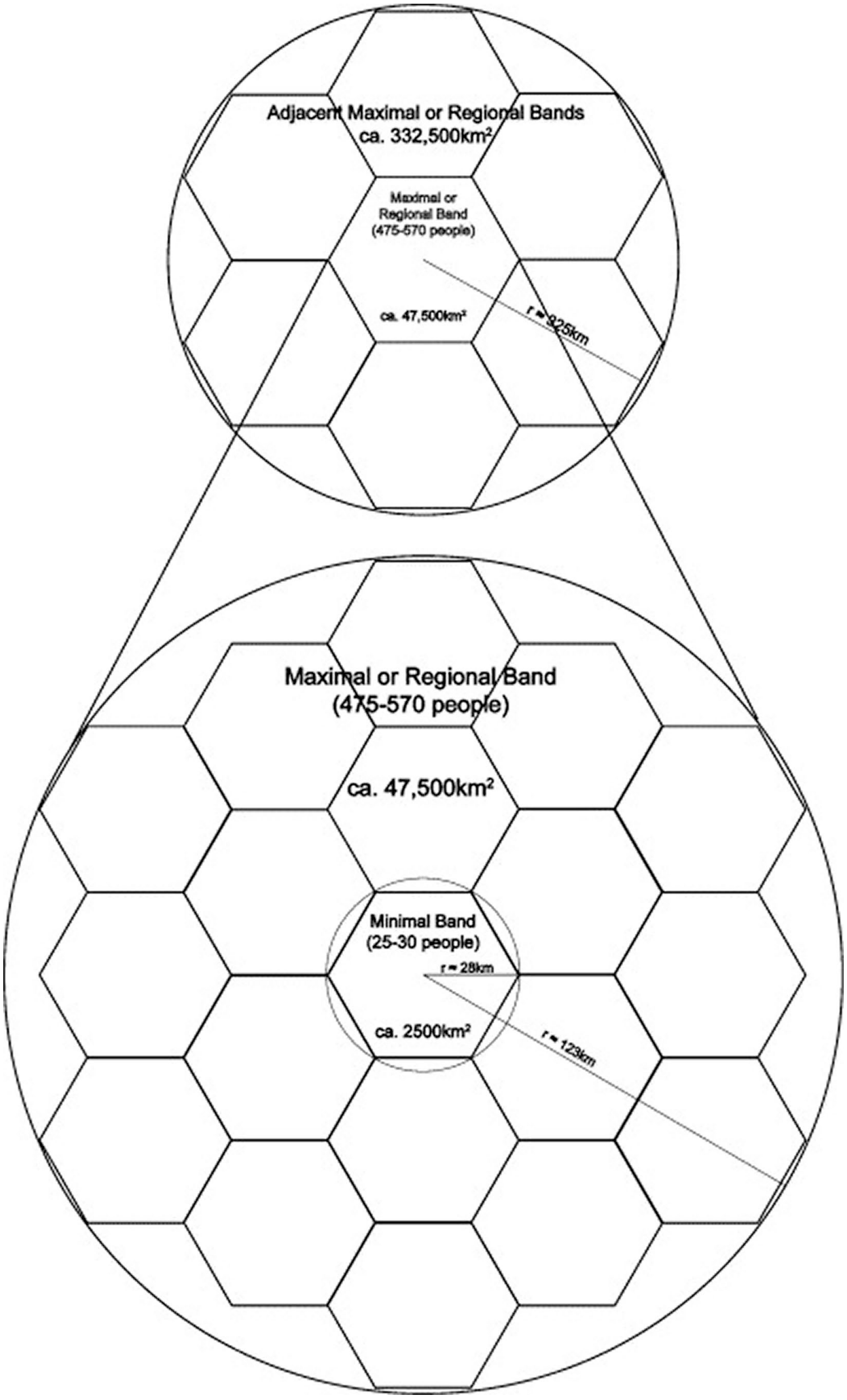

Figure S36. Heuristic model of spatial organization of hunter-gatherer bands and their territories proposed by Whallon (*14*).

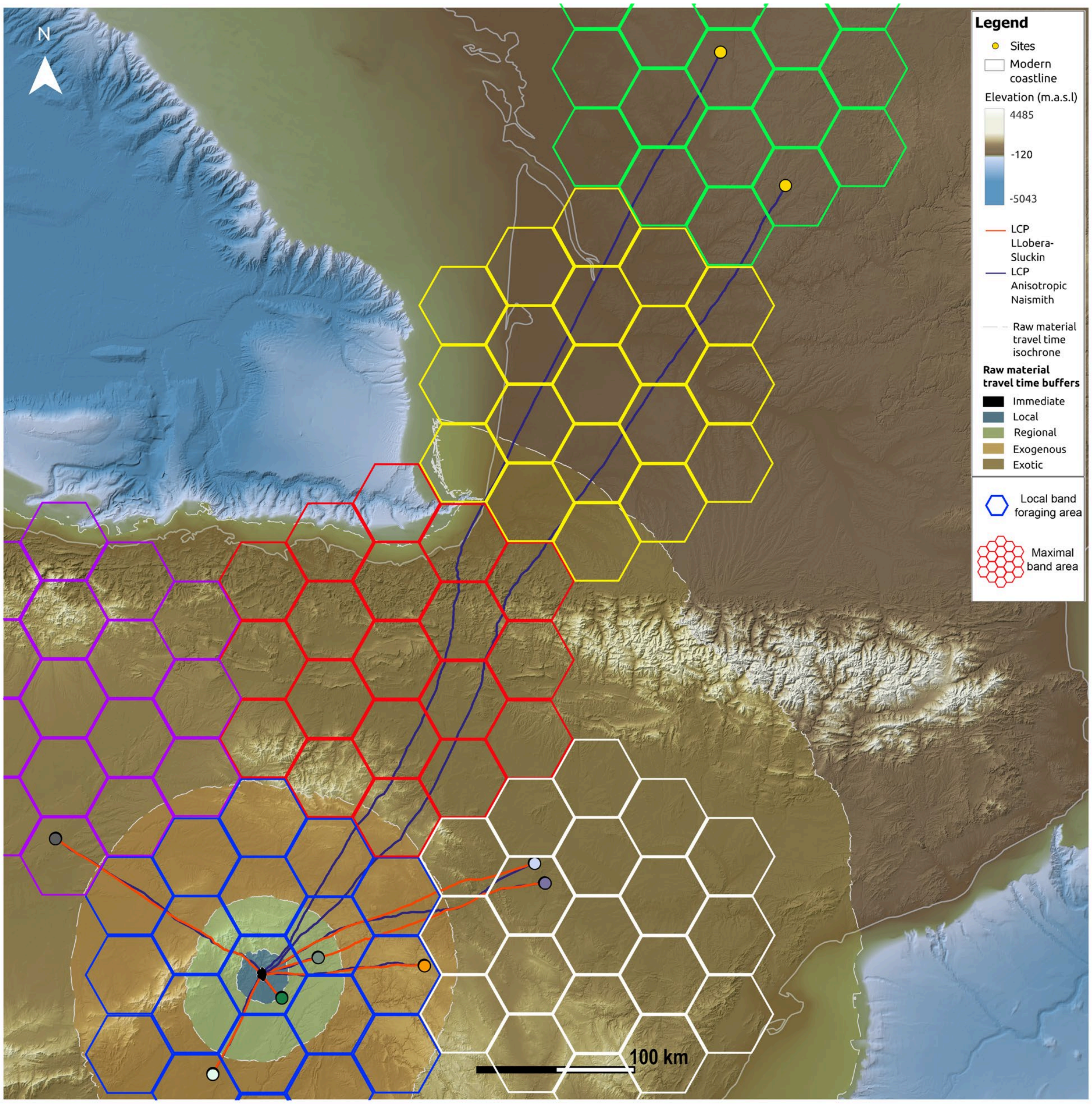

Figure S37. Application of the heuristic model proposed by Whallon (*14*) to the geographic area defined by the sources of the raw material identified at Peña Capón and comparison with the raw-material time-cost classification used in this study.

In Figure S37 we show an ideal subdivision of the territory according to this model, where we represented all the maximal bands (each with a different colour) necessary to maintain the social networks responsible for the raw material circulation documented at Peña Capón. In subsequent figures S38 to S43 we include the minimum number of local and regional bands that would have been involved in the networking process according to the data recorded in each sedimentary level. The outcomes show that in all levels, a minimum of two maximal bands participated in the network. This is the case of Level 0, where the presence at Peña Capón of the exotic lacustrine cherts of the Middle Ebro valley is best explained as a result of an exchange

network involving at least four minimal bands, being some of them integrated within two different maximal bands (Fig. S38). In the Middle Solutrean of Level 1 (Fig. S39) and the Proto-Solutrean of Level 4 (Fig. S43), the network enlarges to include another maximal band, which was most probably responsible for the circulation and exchange of the lacustrine cherts of Mucientes (Middle Duero valley). Naturally, the largest number of maximal bands is found in the Middle Solutrean levels where the Hettangian jasperoids from Southwest France are found. Thus, the wide social networks inferred from the data of Levels 2a, 2b and 3, imply the participation of at least five (Level 3) or six (Levels 2a and 2b) different adjacent maximal bands involving multiple exchange events at different points along the landscape. As already mentioned, it is not possible to know the exact number of exchange events, the actual number of minimal and maximal bands participating in the network, or the distances travelled by the different human groups involved. Therefore, the networking processes represented in Figs. S38 to S43 must be considered as minimum or conservative estimates with respect to the network's complexity in the context of a heuristic model. Likewise, while in the case of exotic and ultra-exotic rocks it is sufficiently demonstrated that their presence at Peña Capón is due to exchange networks, for regional rocks such as the evaporitic cherts of Unit 168 or the jaspers of Unit 155, both direct catchment and exchange remain as hypotheses to be verified or falsified.

In Figs S38 to S43 we also plotted all the currently known archaeological sites that could be part of the social networks documented in each Peña Capón level, including those within the limits of the defined network, as well as others which are located not farther than two hexagons (i.e. 112 km) away in areas where no sites are known [Dataset S5; see also (*31, 59*)]. Thus, for Level 0 we plotted Upper Solutrean sites, which were limited to the single location of *Arenero de Vidal*, in the Manzanares valley (Madrid), where barbed-and-tanged points were reported (*165*). For levels 1, 2a, 2b and 3 we plotted Middle Solutrean sites or sites dated to 25.3-23.9 ka cal BP, as this is the timeframe for the three levels containing Hettangian materials (see Text S1.4.). Finally, for Level 4 no sites were plotted, as there is not a single Proto-Solutrean location recorded in the area defined by the maximal bands involved in the network. Likewise, for the very large areas defined for levels 2a, 2b and 3 (Figs. S40-S42), the plotted sites do not show the density of human settlements expected for maintaining such a broad social system in the long term, as also highlighted by other scholars studying land-use patterns and social networks in Iberia during the LGM (*84*). Even though the existence of empty landscapes with few people is consistent with a scenario involving long-distance trips (*37, 56*), and areas such as the Landes region were known to be a periglacial desert during the Last Glacial (*166*), there are wide empty spaces where human settlements should be present if such broad network had been sustainable through generations (*84*). This is especially the case for the regions separating Peña Capón from the western Pyrenees, where no Middle Solutrean sites are known throughout the Northern *Meseta* and the Ebro valley (see also Fig. 10 in main text). Although rock art depictions of arguably Solutrean age based on stylistic grounds have been reported at Los Casares cave (Iberian System range, Guadalajara province), the open-air site of Domingo García and La Griega cave (northern foothills of the Central System range, Segovia province), their chronological assignment is not enough accurate and hence they cannot be retained as secure evidence of human settlement during the Middle Solutrean (*59, 83, 134, 135*). As discussed in the main text, our results on lithic raw material circulation strengthen the hypothesis that the scarcity of sites in the mentioned regions is mostly due to the absence of systematic research in these areas compared to the Iberian coastal regions, combined with geomorphological factors limiting the location of open-air sites (*17, 18, 59, 78, 82-84*). Among the latter factors, the presence of large dune fields and cover sands along the Duero and Tagus

basins, such as *Tierra de Pinares* sand and the *Manchega* Plain, may explain the absence of Late Pleistocene sites across large areas, as those aeolian sands, deposited during the LGM, the Late Glacial and the Holocene, may have buried older archaeological sites (*84*).

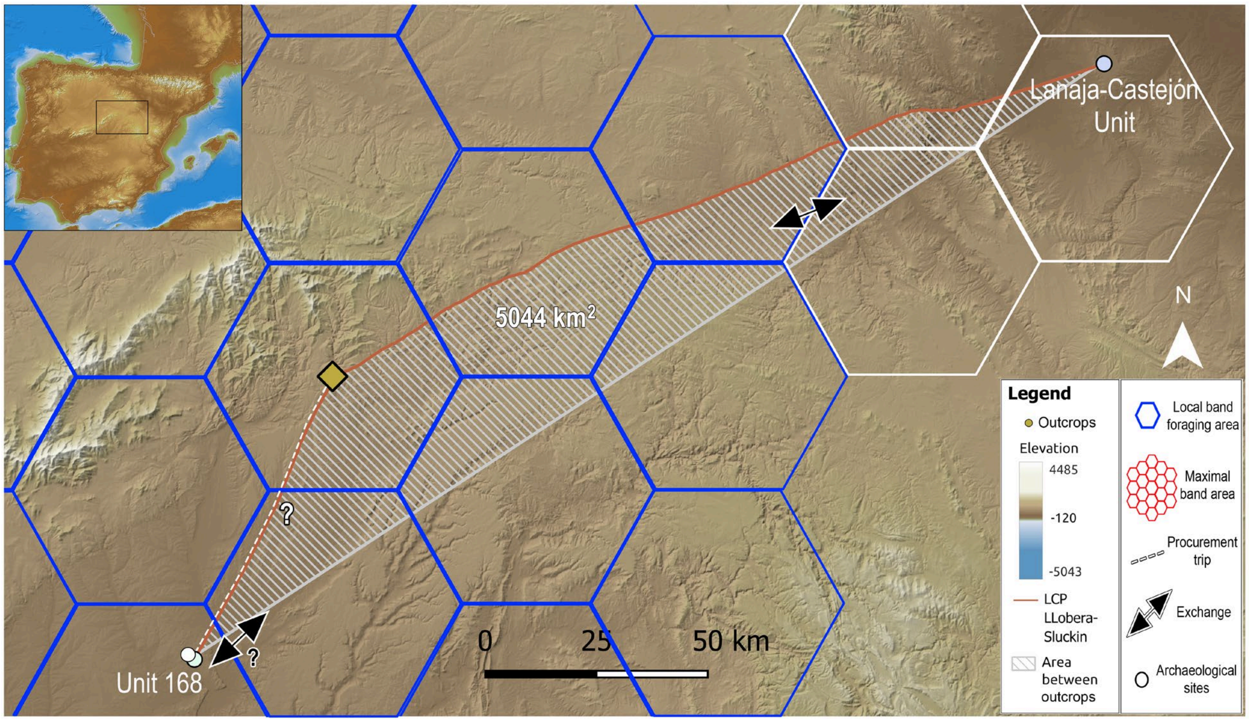

Figure S38. Heuristic model showing the potential minimal and maximal bands involved in the raw material circulation network documented in Peña Capón Level 0.

Furthermore, besides the connection between Peña Capón and the Middle Ebro valley recorded for levels 0 and 1 (Figs. S38-39), there is evidence connecting these and other regions within the extensive network proposed for levels 2a, 2b and 3 at different times during the Upper Palaeolithic. Raw material circulation and shared typological traits have been recorded during the Gravettian, the Upper Solutrean and the Magdalenian across the Ebro valley, which is conceived as an important crossroads connecting the southern plateau, the Mediterranean region, the western Cantabrian area, and ultimately across the Western Pyrenees through the French Aquitaine (*79, 167-168*). Thus, it is to be expected that these routes were also in use during the Middle Solutrean despite sites accurately dated within the time frame of Peña Capón levels 2a, 2b and 3 had not been reported yet.

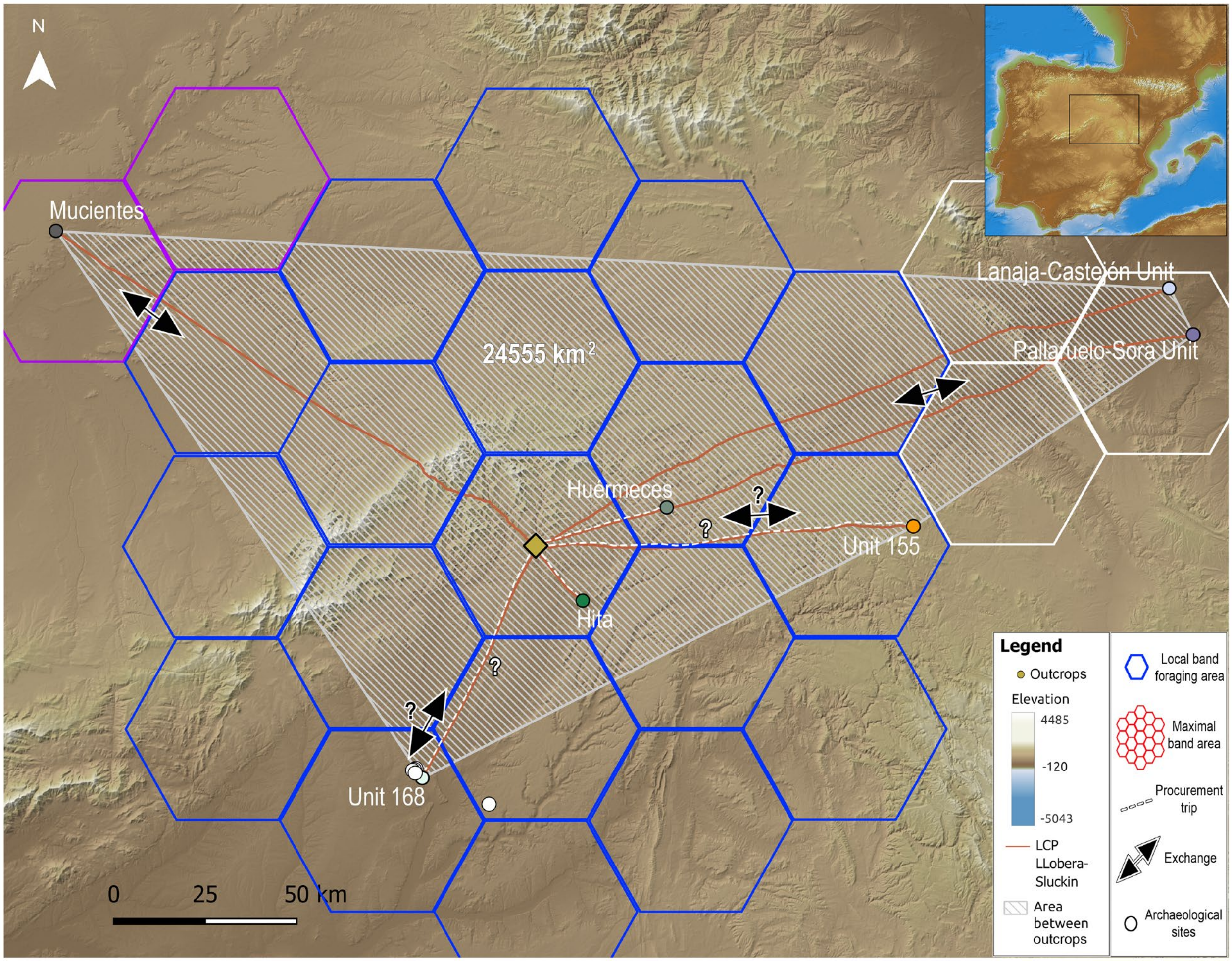

Figure S39. Heuristic model showing the potential minimal and maximal bands involved in the raw material circulation network documented in Peña Capón Level 1.

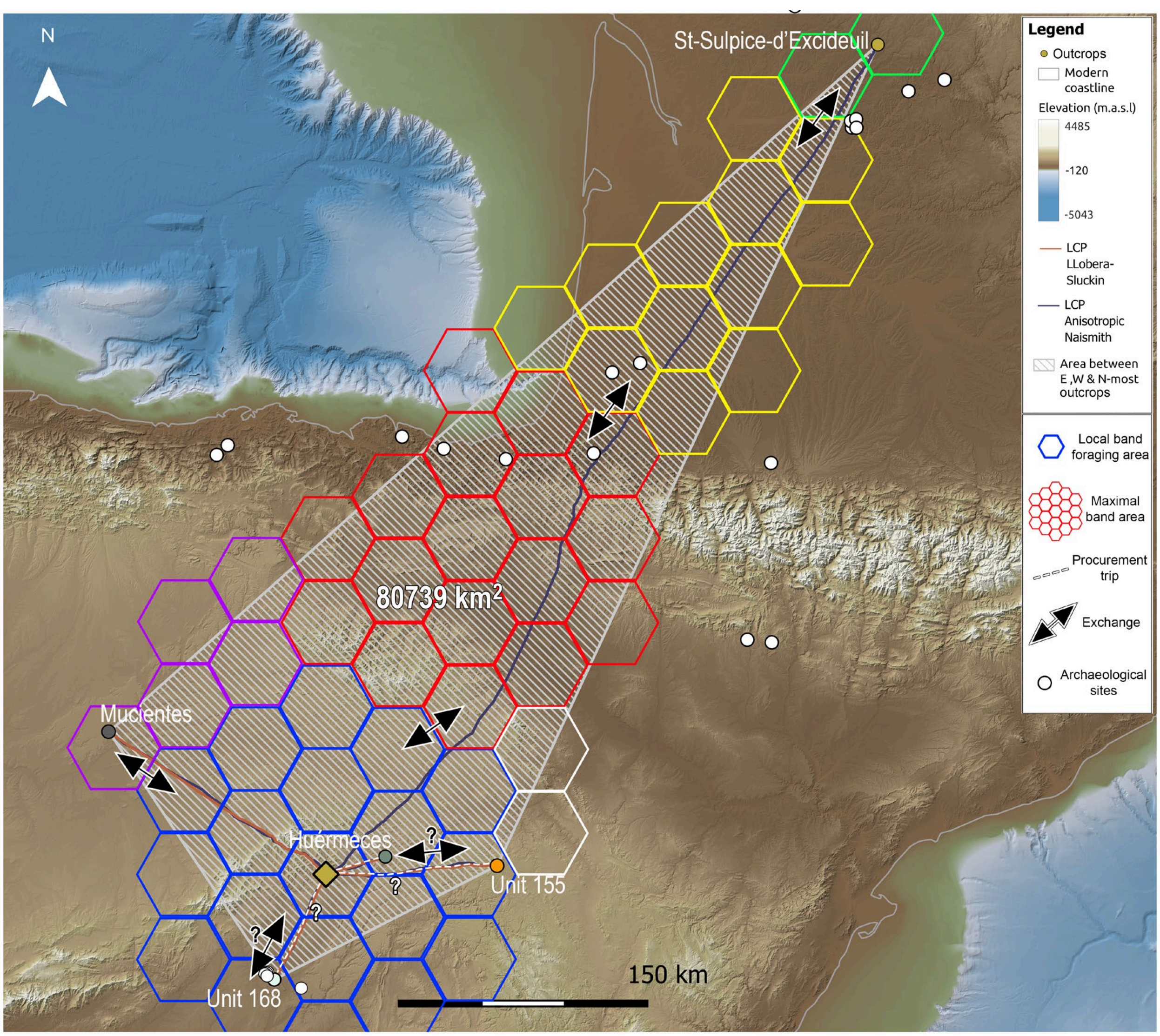

Figure S40. Heuristic model showing the potential minimal and maximal bands involved in the raw material circulation network documented in Peña Capón Level 2a.

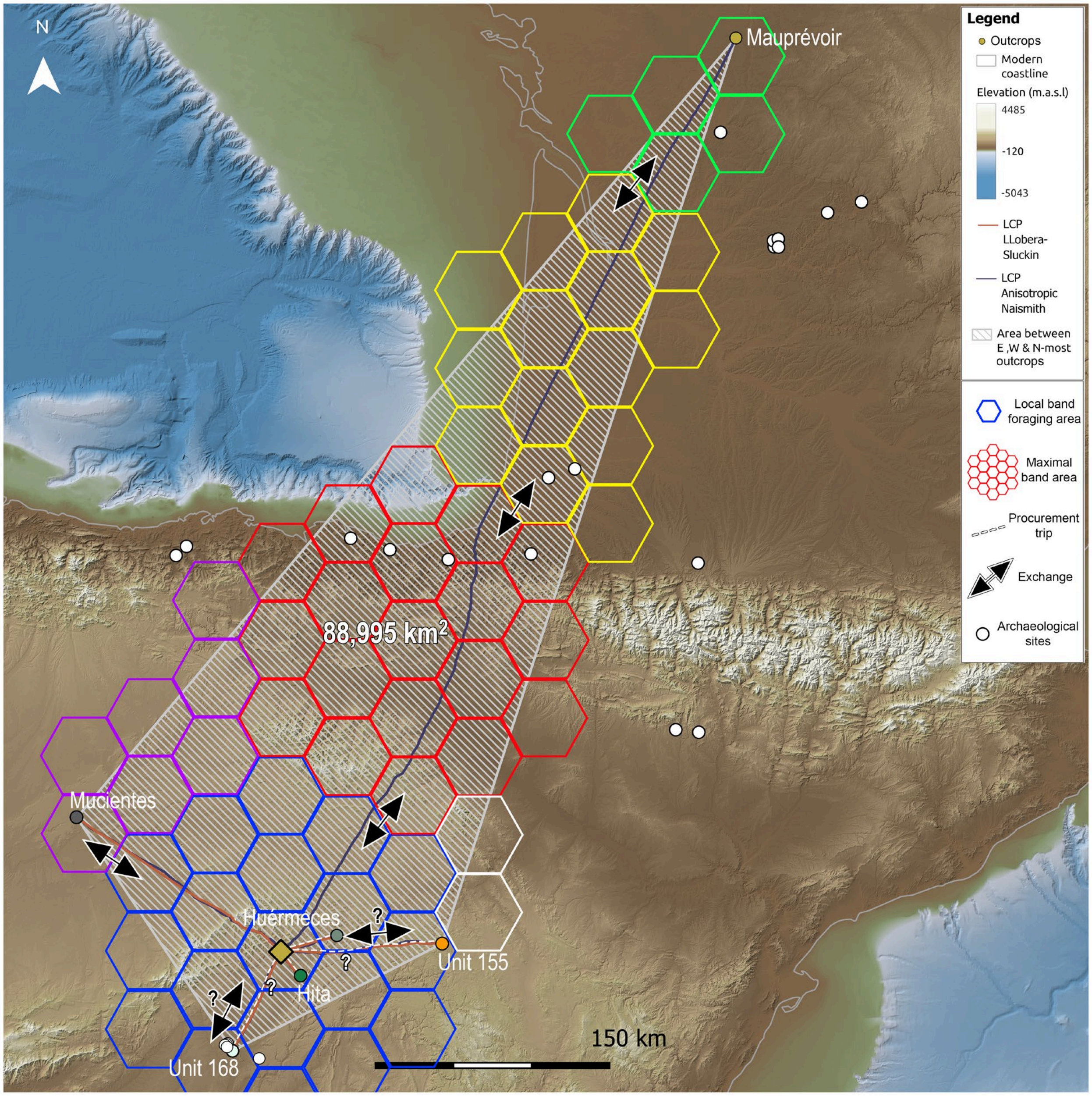

Figure S41. Heuristic model showing the potential minimal and maximal bands involved in the raw material circulation network documented in Peña Capón Level 2b.

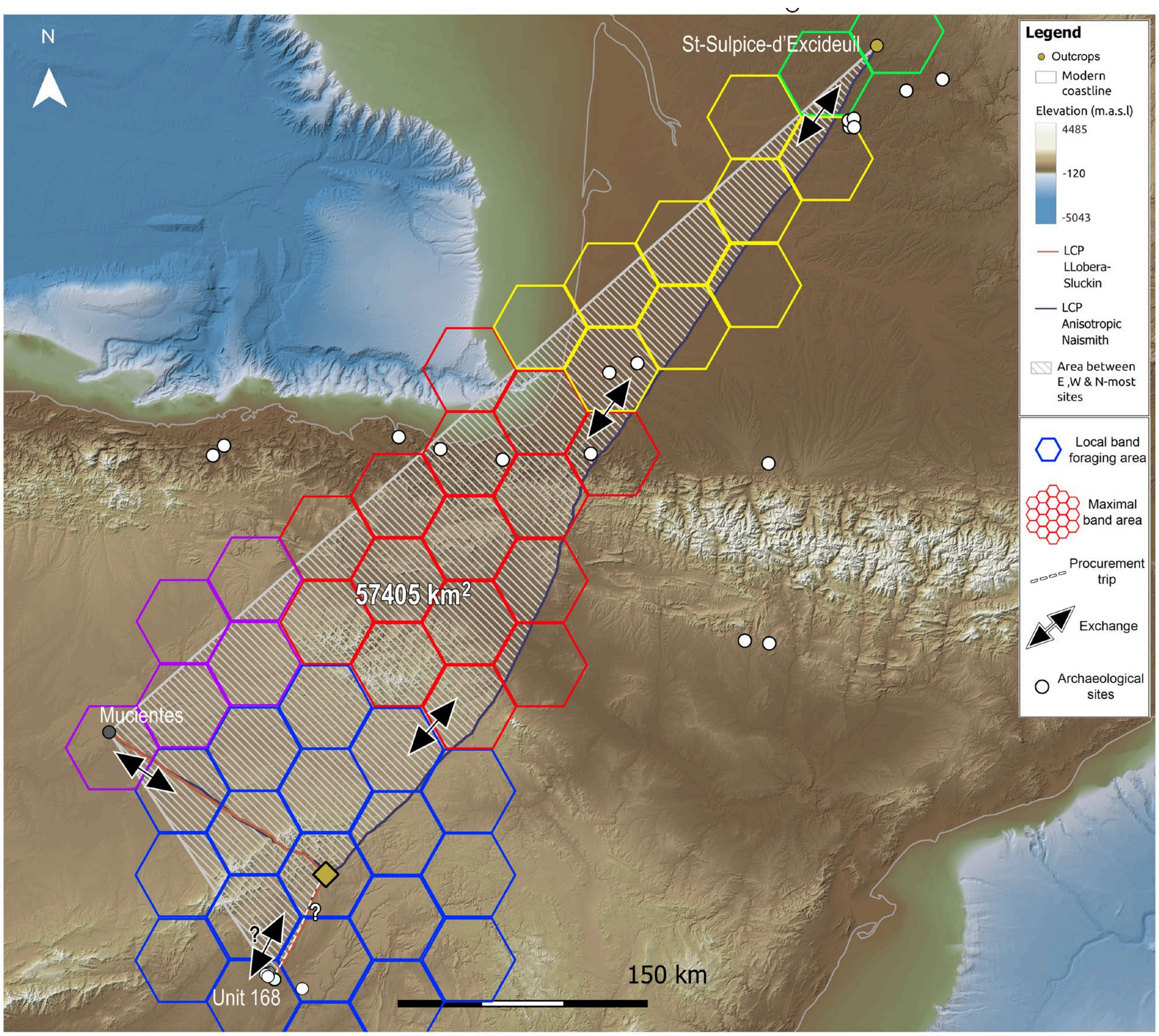

Figure S42. Heuristic model showing the potential minimal and maximal bands involved in the raw material circulation network documented in Peña Capón Level 3.

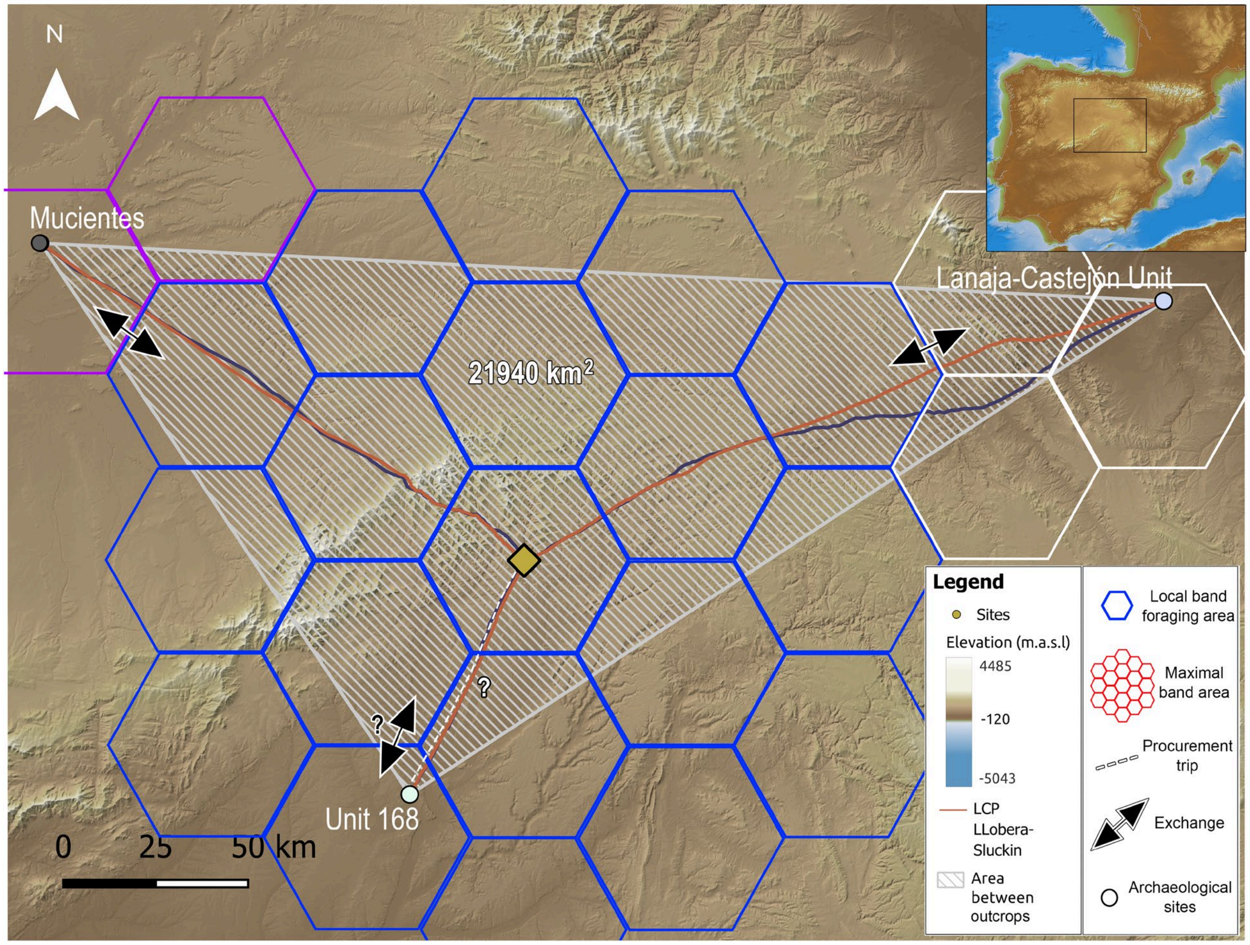

Figure S43. Heuristic model showing the potential minimal and maximal bands involved in the raw material circulation network documented in Peña Capón Level 4.

Text S6. Regional Upper Palaeolithic rock art and stylistic commonalities with SW France

As already discussed in Text S1.6., Peña Capón was not an isolated settlement within the landscape during the Upper Palaeolithic. There are other related sites within its regional setting in the Upper Tagus basin, and their number has been increased in the last years (Fig. S24). Among these locations, El Reno Cave (Valdesotos, Guadalajara province) is found just 9 km from Peña Capón (Fig. S1), in the Upper Jarama River valley, and it hosts a cluster of cave art motifs showing pre-Magdalenian stylistic features compatible with a Solutrean chronology (*83, 169, 170*). The more modest cavity of El Cojo, just 120 m away from El Reno, also bears similar images (*171*). In the absence of direct chronometric dates, the estimated age of these Palaeolithic representations is based on their stylistic nature, as they found strong parallels in other sites of the Iberian interior, such as Los Casares (Guadalajara) and La Griega (Segovia) caves (Fig. S44), and the open-air sites of the Côa Valley, in inland Portugal (*170, 172*). At these sites, and especially in Los Casares and El Reno caves, there are superimpositions with figures showing strong Magdalenian traits found above others suggesting old conventions (*135, 169*). More significantly, the pre-Magdalenian stylistic motifs at the site of Fariseu (Côa Valley) have been archaeologically dated to Solutrean or Gravettian times, as they were covered by archaeological levels dated by radiocarbon and luminescence dating (*17, 173*). All representations from these sites assigned to the pre-Magdalenian phase share a series of thematic, associative and formal characteristics. The most important among them are the existence of a series of little-varying figurative motifs, dominated by horses, deer and ibex (and aurochs especially in the Côa valley), and complemented by the occasional appearance of rectangular signs, and the construction of zoomorphs expressed only as outlines, in absolute profile or using the straight bi-angular perspective, and with very simple stylistic conventions (*170*).

Of particular interest to our study is the existence, among some of these pre-Magdalenian graphic assemblages of the Iberian interior, of some relatively specific features proposing stylistic commonalities with very distant regions, and namely with the Dordogne area in Southwest France. In fact, the most significant parallel connecting these two regions is precisely found in El Reno cave, and hence in close spatial relationship to Peña Capón. Here, the peculiar way of representing the hooves of some of the pre-Magdalenian-style horses holds a special significance. This convention has been documented in two associated figures and consists of a globular depiction of the horses' hooves, in one case by means of a circular linear outline painted in sienna colour, and in the other by using red paint applied in extension to create an oval-shaped hoof (Fig. S45: A & B). This graphical convention constitutes an exception within the chrono-cultural context of the pre-Magdalenian rock art of inland Iberia, where the zoomorphic representations as a rule lack body details, with the ends of the limbs being omitted in virtually all cases. Its presence is also quite exceptional in the cave art inventories of the rest of the Iberian Peninsula, with only one potential case at the *Techo de los Polícromos* of Altamira (Cantabria), where two red horses, also of pre-Magdalenian chronology, show similar features (*174, 175*).

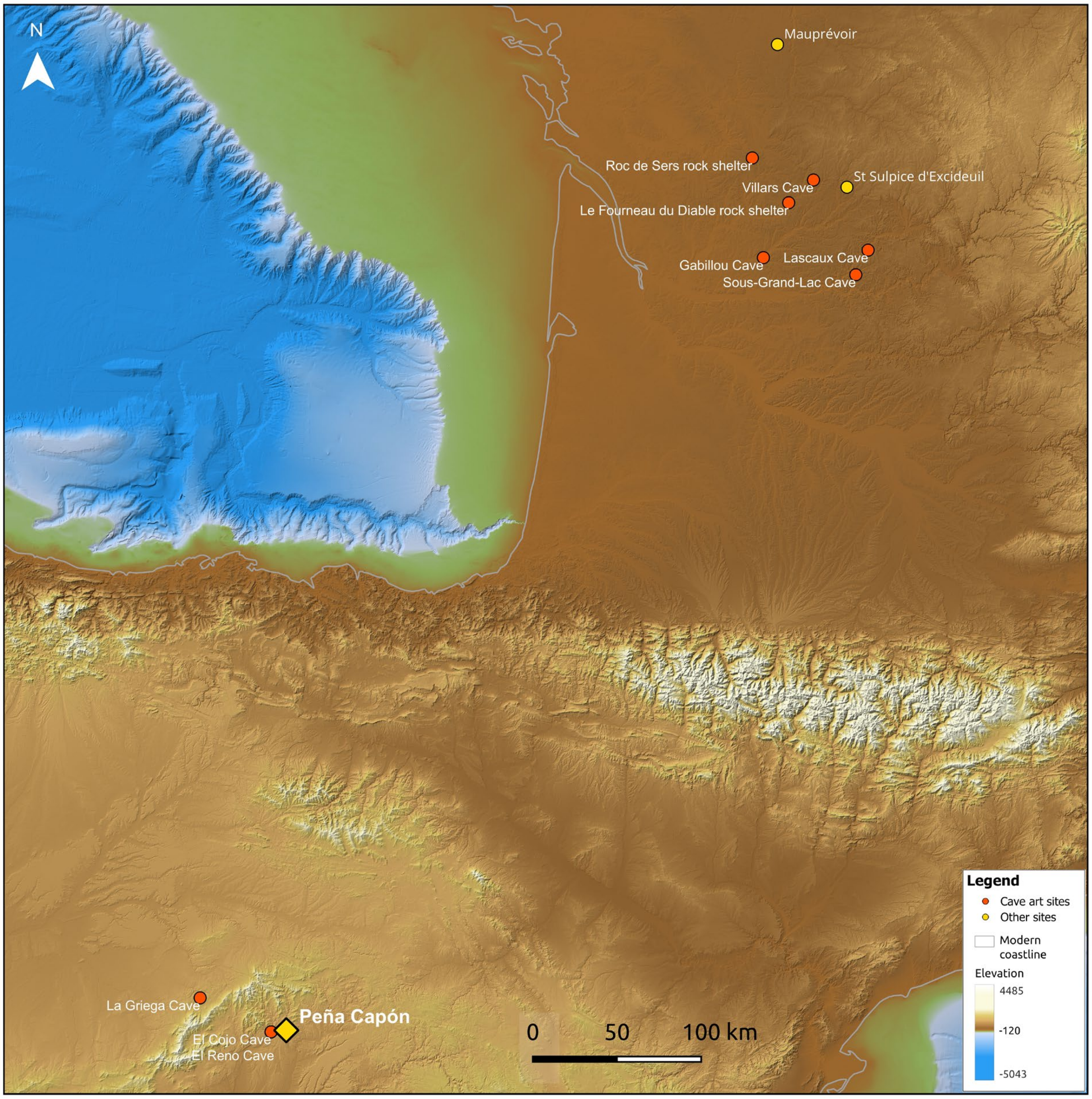

Figure S44. Location of Upper Palaeolithic cave art sites in Central Iberia and Southwest France showing stylistic commonalities based on the depiction of ungulate hooves. The outcrops of Saint-Sulpice-d'Excideuil and Mauprévoir, sources for the jasperoid cherts discarded at Peña Capón, are also plotted.

However, this peculiar way of depicting ungulates' limbs with a circular or oval shape representing the hoof is very common in Southwest France, and more specifically in the regions of Dordogne and Charente (Fig. S46). In fact, the relatively high frequency of this convention led A. Leroi-Gourhan to propose it as one of the specific attributes of his regional 'style III', with well-known examples in the caves of Lascaux (Montignac) (*176*) (Fig. S45: C, Fig. S46: A & B, Fig. S47: 1-3), Gabillou (Sourzac) (*177, 178*) (Fig. S46: C) or Villars (Villars) (*179*) (Fig. S46: D). This convention is found indistinctly on all ungulate species represented in these caves, with special emphasis on horses (Fig. S47: 1) and bovids (Fig. S46: B and Fig. S47: 3), although its

appearance on red deer figures is not uncommon, especially in Lascaux (*176*) (Fig. S47: 2). Its widespread application in this region, together with its scarcity in other areas of Southwest Europe during the Upper Palaeolithic, suggest that this convention holds a chronological, cultural and territorial value, rather than being a "natural" way of representing ungulates' hooves.

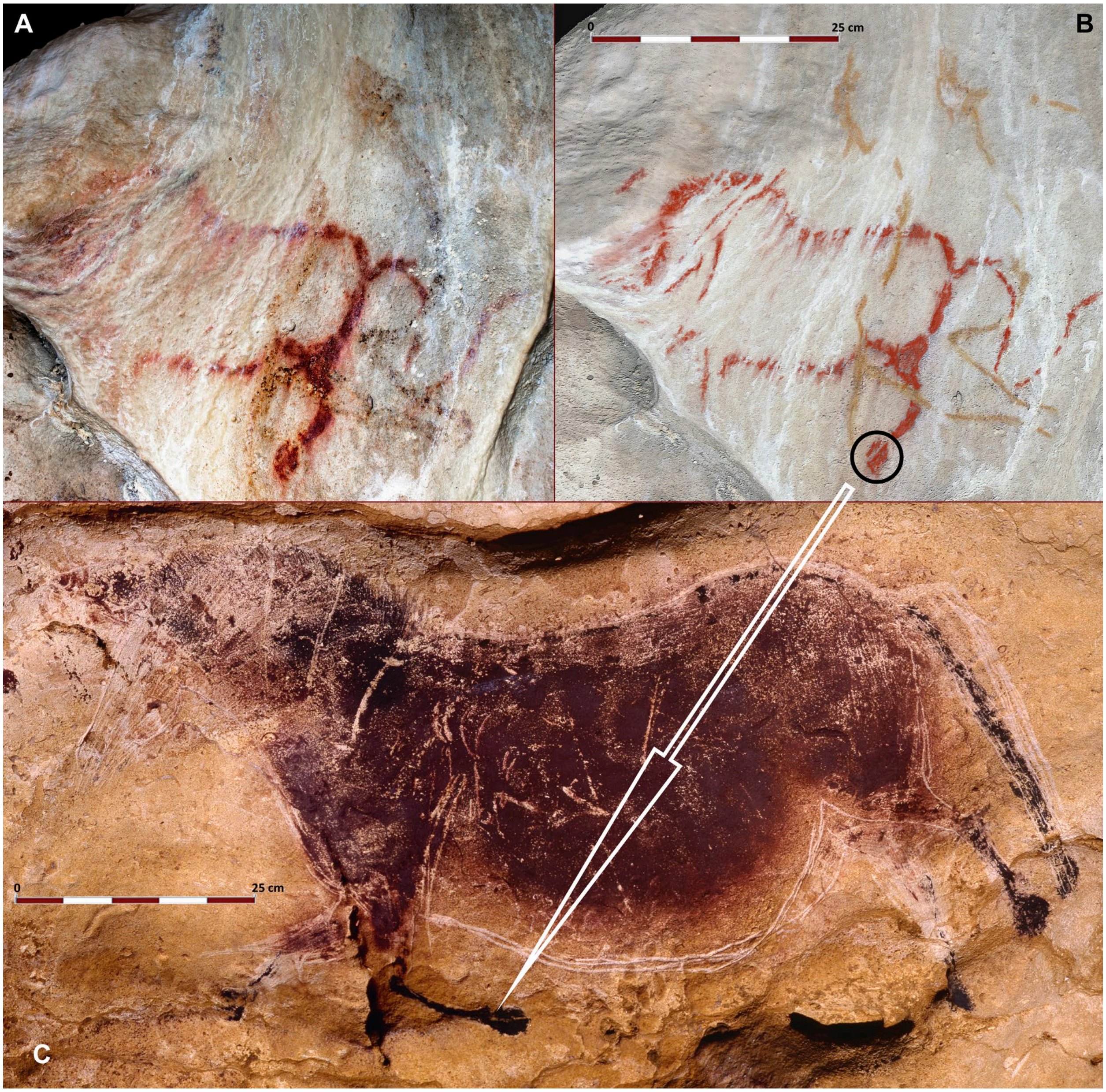

Figure S45. Photo (A) and tracing (B) of two horses painted in red and sienna found at El Reno Cave, showing stylistic commonalities with a painted and engraved equid form La Nef de Lascaux [n. 24 in Leroi-Gourhan (*216*)]. The black circle and the white arrow point to the strong similarities of the ways in which the horses' hooves are represented. Photo credits: A: Javier Alcolea-González, C: N. Aujoulat (Centre national de Préhistoire / Ministère de la Culture).

There is a consensus in considering the cave art of these sites as part of a graphic tradition prior to the 'classicism' of the Middle Magdalenian. Radiocarbon dating of the archaeological

context of Lascaux (*176, 180, 181*) and Gabillou (*180*), and those of the Villars graphic representations (*179*), propose a direct or indirect relationship of these graphic motifs with either the Solutrean-Badegoulian transition (*182*), the Badegoulian (*183*), or the Badedogulian-Magdalenian transition (*181*). In the case of Lascaux, this interpretation implies a very unitary view of its rich and large graphic inventory, which would have been created in its entirety in a few centuries. The multidisciplinary study of the archaeological context of the cave (*176, 184*) would guarantee this homogeneity. Although some have criticized this unitary vision (*185*), it has recently received additional support by means of new radiocarbon determinations of the archaeological context (*181*)–although the direct dating of the paintings, and hence their actual chronology, remains to be addressed.

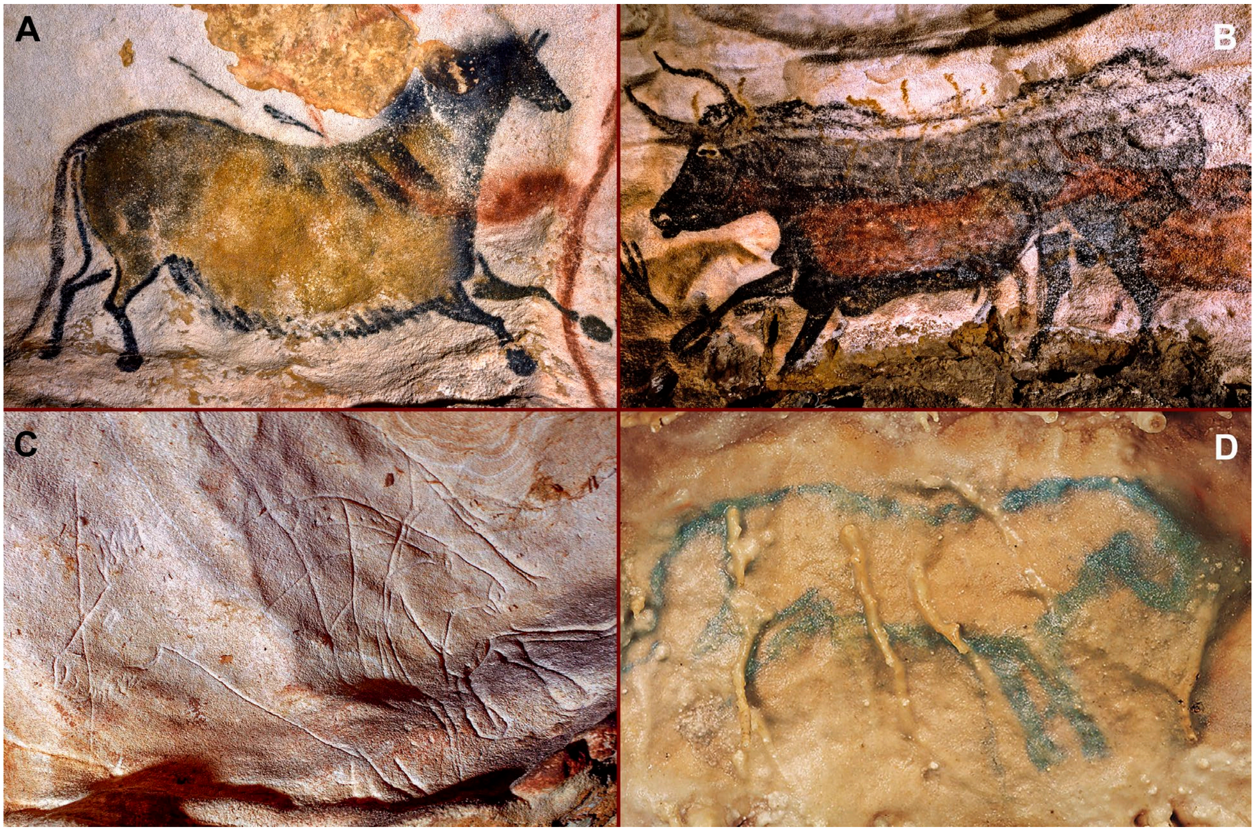

Figure S46. A: Third "Chinese" horse from the Axial Gallery of Lascaux. B. Great black auroch from the Axial Gallery of Lascaux. C. Horse and horse's hind quarters with globular hoves from Gabillou cave. D: Blue horse from Villars cave. Photo credits: A & B: N. Aujoulat (Centre national de Préhistoire / Ministère de la Culture). C: Modified from Heinrich Wendel (© The Wendel Collection, Neanderthal Museum). D: Modified from https://grotte-villars.com, with permission from Catherine Birckel-Versaveaud.

In other sites, the chronological setting of these motifs is more precisely related to the Solutrean, as in the bovids of the sculptural frieze of Fourneau du Diable (*186*) or some of the sculpted horses of Roc de Sers (*187*). In both cases the globular depiction of the ungulates' hooves is sound (Fig. S48). All these cases, together with others of uncertain chronology due to the absence of archaeological context of chronometric dating, such as the horses from Sous-Grand-Lac (*188*) (Fig. S47: 4), show the existence of a regional graphical tradition starting at least during

Solutrean times and lasting probably until the Middle Magdalenian. Hence, the existence in central Iberia of similar stylistic traits during the same period suggests a connection between the two regions, or at least a shared cultural and symbolic background.

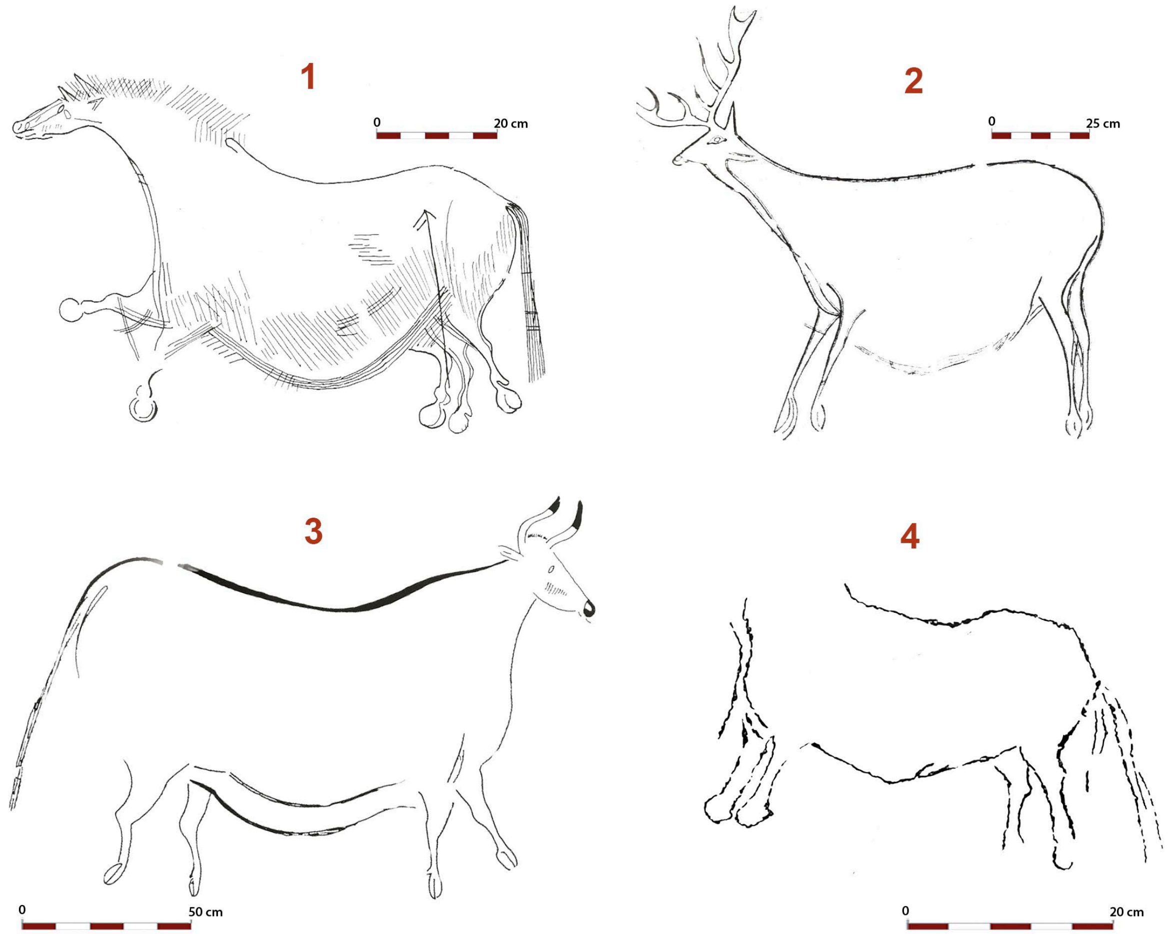

Figure S47. 1: Tracing of the engraved and painted horse from the 'Panel de l'Empreinte' of the Nave of Lascaux [modified from (*216*): Pl. XXIV]. 2: Tracing of an engraved red deer from panel XIV of the Apse of Lascaux [modified from (*217*): Pl. XIXa]. 3: Tracing of the engraved and painted great auroch from the Black cow panel of the Nave of Lascaux [modified from (*216*): Pl. XXV]. 4: Tracing of an engraved horse from Sous Grand Lac cave [modified from (*188*): fig. 5].

In sum, the geochemical evidence from the Peña Capón lithic artefacts, coupled with the rock art similarities between central Iberia and Southwest France (and in turn the techno-typological homogeneity of the ancient and middle phases of the Solutrean), has provided the confirmation of the hypothesis that Solutrean hunter-gatherers were interconnected throughout the whole (or most of the) extension of the technocomplex (*15, 17, 18, 31, 59, 64, 65, 189*). The existence at Peña Capón of lithic artefacts produced on rocks sourced from the western border of the French Central Massif not only confirms this interconnection, but also provides robust evidence in support of recurrent physical contacts among foragers along vast extensions of the territory, and that these contacts were persistent at least throughout the whole extension of the Middle Solutrean, lasting

~1400 years. Furthermore, the nature of the Peña Capón region as an aggregation area during this period, as discussed in the Main text, is supported by both the presence at the site of exotic and ultra-exotic rocks, and the existence of others arguably contemporary sites in the surroundings, including the rock art depictions of El Reno cave. As has been stressed by several scholars (*80, 190*), the symbolic and social nature of Palaeolithic rock art depictions could be related, at least in some contexts, to exchange processes and the materialization of aggregation sites. The combination of explicit symbolic evidence and stone tools when studying Paleolithic social networks is thus revealed as an effective and comprehensive approach (*17, 23*).

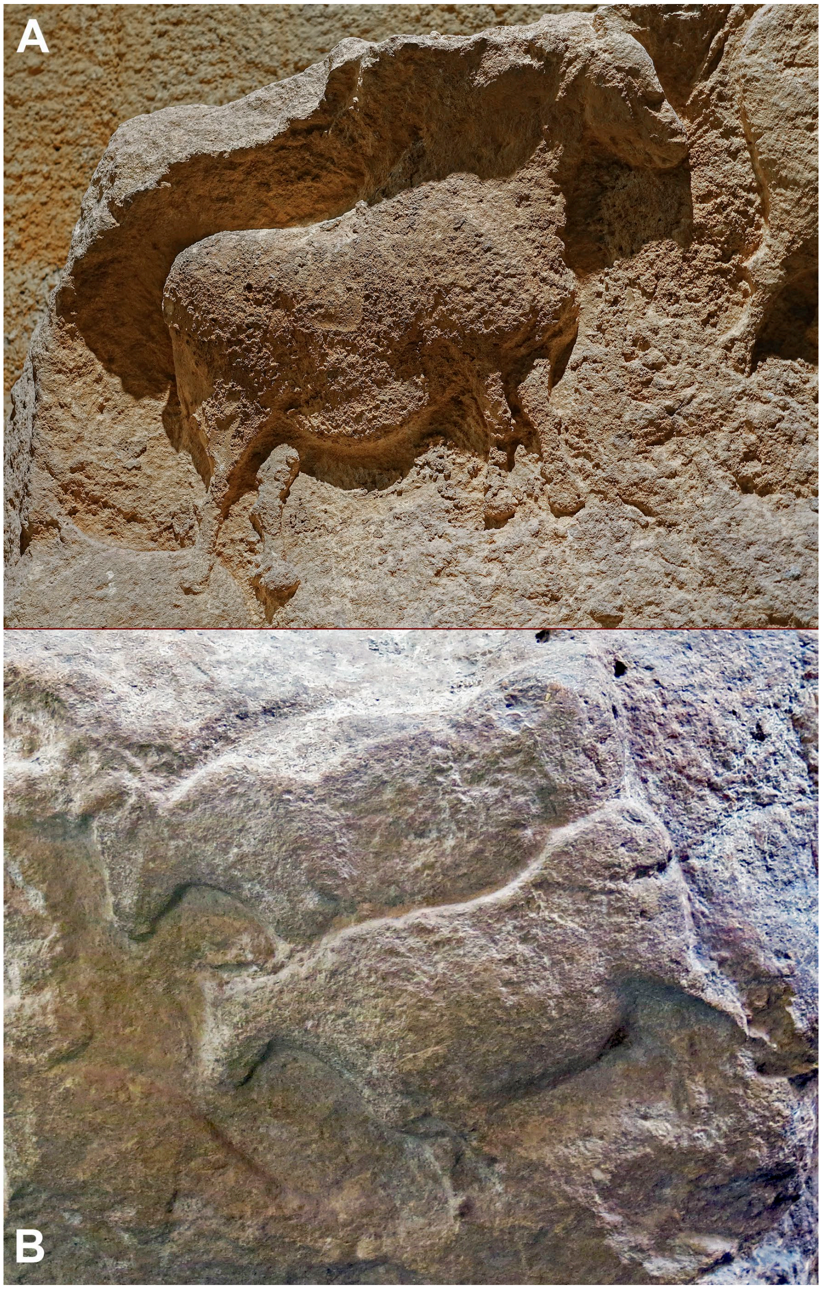

Figure S48. A: Bas-relief horse from Roc-de-Sers. B: Bas-relief aurochs from Fourneau-du-Diable. Photo credits: A: Don Hitchcock, https://www.donsmaps.com/rocdesers.html. B. Don Hitchcock, https://www.donsmaps.com/diable.html.

Text S7. Use-wear analysis and diacritical reading of the foliate preform from Level 3 (PC23-11)

Among the three archaeological artifacts sourced in the Hettangian outcrops from the western border of the French Central Massif and discarded at Peña Capón during the Middle Solutrean (two more artifacts correspond to surface findings; see Main Text and Dataset S3), only one of them is a retouched tool (Fig. 1 in Main Text). Labelled as PC23-11 for the LA-ICP-MS analysis, this piece is not, however, a finished tool, but a bifacially reduced blank, most probably a preform [e.g. (*191*)] aimed at the eventual production of a projectile point of the laurel leaf type, typical of the Middle and Upper Solutrean (*122*). Considering the relevance of this piece (Fig. 1.1 in Main Text) we have conducted (1) a use-wear analysis aimed at shedding light on the potential uses and alterations of the object, and (2) a diacritical reading aimed at reconstructing the knapping sequence involved in its production. The combination of both methodologies allows robust data to be obtained about the 'life history' of this artefact, considered in the context of its transport from Southwest France to Central Iberia.

Few functional analyses have been carried out on Solutrean stone artifacts, and most of them have been focused on small-sized tools or weapons, such as shouldered points (or *points à cran*), barbed-and-tanged points, bladelets and flakes (*192-195*). Yet, to our knowledge, no use-wear analyses have been conducted on middle or large-sized laurel leaves. The systemic observation of PC23-11, focused both on the artefact's edges and its two flat surfaces, was undertaken at low and high magnification (40 x, 100x, 200x) with a reflected light microscope (Nikon Eclipse LV100N POL). We identified both surfaces of the object as A and B (see Fig. S49) and conceived left and right edges taking as reference the orientation of surface A.

The main traces identified were short striations and especially rounded ridges, which were mostly recorded on the artefact's flat surfaces, whilst most of the edge perimeter was revealed as fresh, with only some very localized nondiagnostic shallow polish. No significant post-depositional surface modifications were identified. The rounded ridges (shaded in yellow in Fig. S50) appear mostly on the highest areas (maximal thickness) of both surfaces, and when observed with high magnification (100x and 200x) some of them show short multi-directional striations (Fig. S49: A.6 and B.8). Only a short stretch of the right edge shows similar rounded ridges, located in the most convex part of such edge, which is also the widest part of the preform. Most of the artefact's perimeter exhibits micro-scars that were probably related to the faceting of striking platforms. Also, a spot on the right edge near the proximal end shows a set of short parallel striations placed transversally to the edge (Fig. S49: B.3) that could have been produced by the action of knapping, as it has been recognized in other archaeological and experimental contexts (*196-199*). Only two small zones on the right edge show polished areas, which are nondiagnostic due to their weakness and shallowness (open pattern and irregular topography).

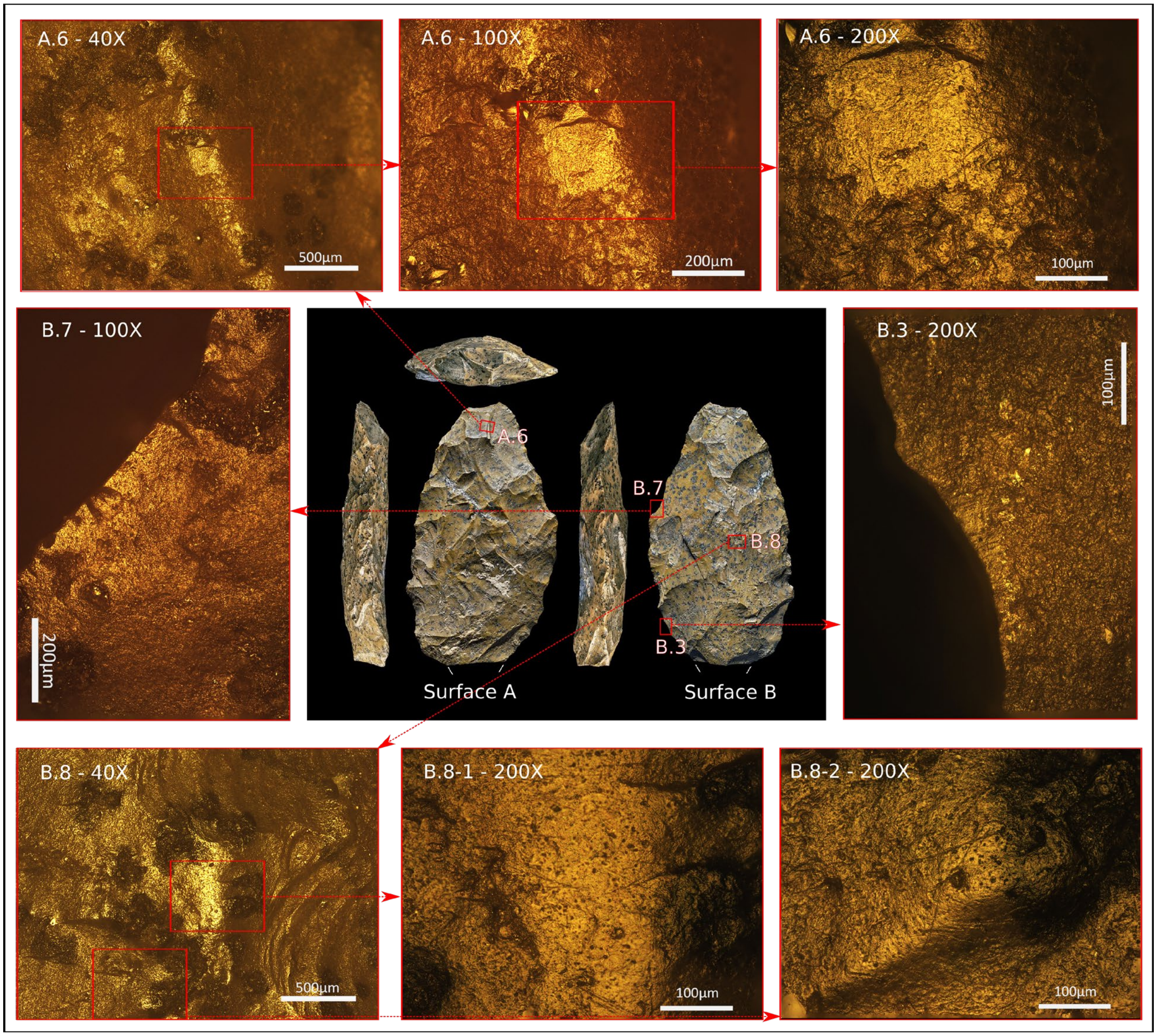

Figure S49. Use-wear analysis on PC23-11 (laurel leaf preform made on Hettangian jasperoid chert discarded at Peña Capón Level 3). Both surfaces of the bifacial piece show rounded ridges (see Fig. S50) with multi-directional short striations (A.6, B.8). Some limited sections of the edges show weak and non-diagnostic polishes probably related to a one-off use (B.7) and technical traces related to the production stage (B.3). Photo credits: Felipe Cuartero and Manuel Alcaraz-Castaño.

Rounding in stone tools has usually been associated with the working of dry hide, soft stone or even soil (*196, 197, 200*) and it is considered a good indicator showing the contact between the tool and the worked material (*198*). It is also analyzed for understanding some properties of the worked material, such as its abrasiveness (*201*). Rounding is also a characteristic feature of post-depositional surface modifications due to geological processes (such as rolling into sediment with different clasts), but in such cases it appears equally distributed on the edges and the surfaces (*202*). As for the multi-directional striations, these have been observed in experiments tanning hide with ochre as an additive (*198*). More significantly, both striations and rounding have been identified as wear traces related to the transportation of lithic objects in quivers and leather bags.

By means of experimental programs, transportation wear has been replicated by a number of scholars, not only in chert materials (*203-206*), but also in bone and ivory artifacts (*207*). In all cases, major wear traces in the form of random striation and edge and ridge rounding were reported arising from the transportation of lithics, both as a consequence of their contact with other lithic artefacts within the bag, or with the leather bag itself. Although we have not conducted detailed experiments with the Hettangian jasperoids located at Peña Capón, the similarity of the wear traces found at PC23-11 with those reported by the mentioned scholars, coupled with the geochemical results demonstrating that at least the raw material of the object was indeed transported, strongly suggest that such transportation left wear traces on the object. Furthermore, the diacritical analysis of this object shows that such traces were produced after the piece was knapped, and hence it was probably transported in its final form from the location where it was produced. Whether such location was somewhere close to the raw material's source, in Saint-Sulpice-d'Excideuil and Clermont-d'Excideuil outcrops, or anywhere else within the social network connecting such area with Peña Capón (Figs. S33 & S42), is difficult to ascertain, as it has been demonstrated that transport wear can be produced during journeys of less than 60 km (*206*).

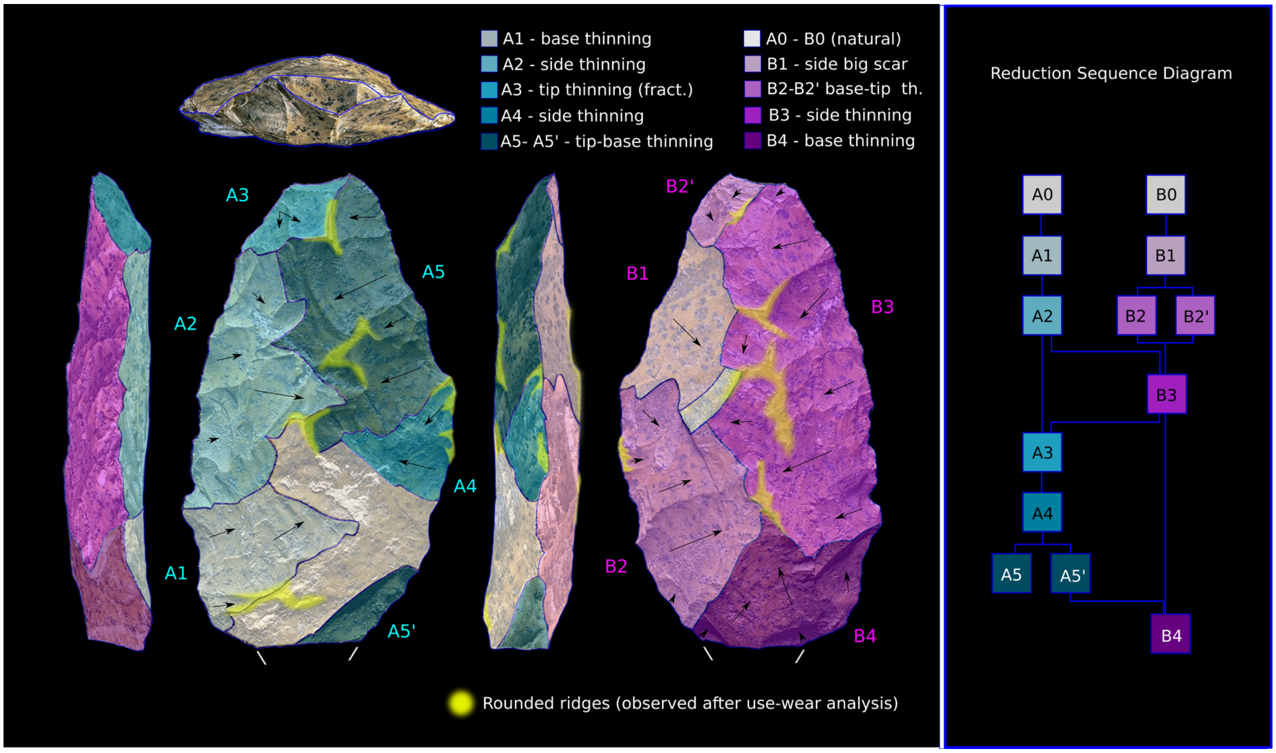

Figure S50. Diacritical analysis and reduction sequence diagram of PC23-11 (laurel leaf preform made on Hettangian jasperoid chert discarded at Peña Capón Level 3). The reduction process was produced in nine main phases on both faces. The location of the rounded ridges observed in the wear analysis and their relation to the diacritical analysis show that this rounding was produced after the artefact was shaped.

The diacritical analysis of PC23-11 was aimed at reconstructing the knapping sequence of this foliate preform by chronologically ordering its removal negatives into different phases or series (*208, 209*). We mainly followed the parameters proposed by Baena & Cuartero (*209*), according to which we identified nine main stages of reduction, which were subsequently

organized into a so-called reduction sequence diagram (*210*) (Fig. S50). The blank used for shaping this preform was probably a large flake or slab limited by natural parallel surfaces (surfaces A0 and B0 in Fig. S50). We identified a first series of scars on surface A from the base to the tip on the left edge (A1 and A2), followed by a second set of removals on surface B and the right edge, from the tip to the base (B1 and B2-B2'). A second series of removals on surface B from the left edge (B3) was followed by a single blow on the left edge on surface A, next to the tip, that broke the distal end (A3). The last series on surface A, on the right edge (A4, A2) and on the base of surface B (B4) completed the configuration of the final rough ovate perimeter of the artefact. As discussed above, by superposing the rounded ridges identified by the wear analysis (yellow shading in Fig. S50) onto the diacritical reading, we can clearly perceive that there is no relation between this rounding and the different phases of production, as it affected equally to all of the topographically highest (thickest) reliefs of this preform. Therefore, the rounding, and hence the transportation of the object, was produced after its final configuration by knapping.

Provenance

The studied archaeological artifacts were excavated at the Peña Capón site during the 2015, 2019, 2021 and 2022 fall seasons. Geological samples were collected from natural outcrops during several field campaigns in 2021, 2023, 2024 and 2025. The artifacts were validated for authenticity by the authors, as they come from secure stratigraphic and geomorphological contexts dating to the Late Pleistocene, and have been precisely dated by the radiocarbon assessment of organic materials from the same stratigraphic levels. All archaeological assemblages from Peña Capón are curated at the University of Alcalá and the *Museo de Guadalajara* (Spain) and are available for any researcher upon request.

Dataset S1. (separate file)
Data of all sampled outcrops & geological units.

Dataset S2 (separate file)
Data of the outcrops & geological units confirmed by LA-ICP-MS as sources.

Dataset S3 (Excel file)
Geochemical raw data for all archaeological and geological samples analyzed by LA-ICP-MS.

Dataset S4 (separate file)
Data on timecosts and distances from Peña Capón to outrcrops/units confirmed as sources.

Dataset S5 (separate file)
Data of sites dated to 25.3 - 23.9 ka cal BP or classified as Middle Solutrean within the social network of levels 2a, 2b & 3.

Dataset S6 (separate file)
Data and technological study of lithic assemblages from Levels 0 to 6.

REFERENCES

1. P. Wiessner, "Risk, reciprocity and social influences on Kung San economics," in *Politics and History in Band Societies*, E. Leacock, R. Lee, Eds. (Cambridge Univ. Press, 1982), pp. 61–84.

2. K. R. Hill, R. S. Walker, M. Bozicević, J. Eder, T. Headland, B. Hewlett, A. M. Hurtado, F. Marlowe, P. Wiessner, B. Wood, Co-residence patterns in hunter-gatherer societies show unique human social structure. *Science* **331**, 1286–1289 (2011).

3. R. Whallon, W. A. Lovis, R. K. Hitchcock, Eds., *Information and its Role in Hunter-Gatherer Bands* (UCLA/Cotsen Institute of Archaeology Press, 2011).

4. C. Apicella, F. Marlowe, J. Fowler, N. A. Christakis, Social networks and cooperation in hunter-gatherers. *Nature* **481**, 497–501 (2012).

5. R. L. Kelly, *The Lifeways of Hunter-Gatherers: The Foraging Spectrum* (Cambridge Univ. Press, 2013).

6. A. B. Migliano, A. E. Page, J. Gómez-Gardeñes, G. D. Salali, S. Viguier, M. Dyble, J. Thompson, N. Chaudhary, D. Smith, J. Strods, R. Mace, M. G. Thomas, V. Latora, L. Vinicius, Characterization of hunter-gatherer networks and implications for cumulative culture. *Nat. Hum. Behav.* **1**, 0043 (2017).

7. F. Marlowe, *The Hadza: Hunter-Gatherers of Tanzania* (University of California Press, 2019).

8. J. Fernández-López de Pablo, V. Romano, M. Derex, E. Gjesfjeld, C. Gravel-Miguel, M. J. Hamilton, A. B. Migliano, F. Riede, S. Lozano, Understanding hunter-gatherer cultural evolution needs network thinking. *Trends Ecol. Evol.* **37**, 632–636 (2022).

9. B. Buchanan, M. J. Hamilton, "Networks and cultural transmission in hunter-gatherer societies," in *The Oxford Handbook of Archaeological Network Research*, (Oxford Univ. Press, 2023), pp. 459–474 .

10. C. Gamble, "Culture and society in the Upper Paleolithic of Europe," in *Hunter-Gatherer Economy in Prehistory*, G. N. Bailey, Ed. (Cambridge Univ. Press, 1983), pp. 201–211.

11. M. A. Jochim, *A Hunter-Gatherer Landscape: Southwest Germany in the Late Paleolithic and Mesolithic* (Plenum Press, 1998).

12. C. Gamble, *The Paleolithic Societies of Europe* (Cambridge Univ. Press, 1999).

13. M. Vanhaeren, F. d'Errico, Aurignacian ethno-linguistic geography of Europe revealed by personal ornaments. *J. Archaeol. Sci.* **33**, 1105–1128 (2006).

14. R. Whallon, Social networks and information: Non-"utilitarian" mobility among hunter-gatherers. *J. Anthropol. Archaeol.* **25**, 259–270 (2006).

15. W. E. Banks, J. Zilhão, F. d'Errico, M. Kageyama, A. Sima, A. Ronchitelli, Investigating links between ecology and bifacial tool types in Western Europe during the Last Glacial Maximum. *J. Archaeol. Sci.* **36**, 2853–2867 (2009).

16. J. Féblot-Augustins, "Revisiting European Upper Paleolithic raw material transfers: The demise of the cultural ecological paradigm?," in *Lithic Materials and Paleolithic Societies*, B. Adams, B. S. Blades, Eds. (Wiley-Blackwell, 2009), pp. 25–46.

17. T. Aubry, L. Luís, J. Mangado Llach, H. Matias, We will be known by the tracks we leave behind: Exotic lithic raw materials, mobility and social networking among the Côa Valley foragers (Portugal). *J. Anthropol. Archaeol.* **31**, 528–550 (2012).

18. T. Aubry, L. Luís, J. Mangado, H. Matias, Adaptation to resources and environments during the Last Glacial Maximum by hunter-gatherer societies in Atlantic Europe. *J. Anthropol. Res.* **71**, 523–544 (2015).

19. A. Tomasso, G. Porraz, Hunter-gatherer mobility and embedded raw-material procurement strategies in the Mediterranean Upper Paleolithic. *Evol. Anthropol.* **25**, 164–174 (2016).

20. O. Fuentes, C. Lucas, E. Robert, An approach to Palaeolithic networks: The question of symbolic territories and their interpretation through Magdalenian art. *Quat. Int.* **503**, 233–247 (2019).

21. V. Romano, S. Lozano, J. Fernández-López de Pablo, Reconstructing social networks of Late Glacial and Holocene hunter-gatherers to understand cultural evolution. *Philos. Trans. R. Soc. B* **377**, 20200318 (2022).

22. L. V. Golovanova, V. B. Doronichev, E. V. Doronicheva, V. F. Sapega, M. S. Shackley, Long-distance contacts and social networks of the Upper Palaeolithic humans in the North-Western Caucasus (Mezmaiskaya Cave, Russia). *J. Archaeol. Sci. Rep.* **39**, 103118 (2021).

23. C. Gravel-Miguel, F. Coward, "Paleolithic social networks and behavioral modernity," in *The Oxford Handbook of Archaeological Network Research* (Oxford Univ. Press, 2023), pp. 443–458.

24. M. Sánchez de la Torre, E. Rafart, C. González-Olivares, B. Gratuze, X. Mangado, Long-distance movements during the last glacial maximum in the Pyrenean mountain range: Fresh insights for Montlleó archaeological site. *J. Archaeol. Sci. Rep.* **61**, 104905 (2025).

25. S. L. Kuhn, Moving on from here: Suggestions for the future of "mobility thinking" in studies of paleolithic technologies. *J. Paleolit. Archaeol.* **3**, 664–681 (2020).

26. Q. Fu, C. Posth, M. Hajdinjak, M. Petr, S. Mallick, D. Fernandes, A. Furtwängler, W. Haak, M. Meyer, A. Mittnik, B. Nickel, A. Peltzer, N. Rohland, V. Slon, S. Talamo, I. Lazaridis, M. Lipson, I. Mathieson, S. Schiffels, P. Skoglund, A. P. Derevianko, N. Drozdov, V. Slavinsky, A. Tsybankov, R. G. Cremonesi, F. Mallegni, B. Gély, E. Vacca, M. R. G. Morales, L. G. Straus, C. Neugebauer-Maresch, M. Teschler-Nicola, S. Constantin, O. T. Moldovan, S. Benazzi, M. Peresani, D. Coppola, M. Lari, S. Ricci, A. Ronchitelli, F. Valentin, C. Thevenet, K. Wehrberger, D. Grigorescu, H. Rougier, I. Crevecoeur, D. Flas, P. Semal, M. A. Mannino, C. Cupillard, H. Bocherens, N. J. Conard, K. Harvati, V. Moiseyev, D. G. Drucker, J. Svoboda, M. P. Richards, D. Caramelli, R. Pinhasi, J. Kelso, N. Patterson, J. Krause, S. Pääbo, D. Reich, The genetic history of Ice Age Europe. *Nature* **534**, 200–205 (2016).

27. C. Posth, H. Yu, A. Ghalichi, H. Rougier, I. Crevecoeur, Y. Huang, H. Ringbauer, A. B. Rohrlach, K. Nägele, V. Villalba-Mouco, R. Radzeviciute, T. Ferraz, A. Stoessel, R. Tukhbatova, D. G. Drucker, M. Lari, A. Modi, S. Vai, T. Saupe, C. L. Scheib, G. Catalano, L. Pagani, S. Talamo, H. Fewlass, L. Klaric, A. Morala, M. Rué, S. Madelaine, L. Crépin, J.-B.

Caverne, E. Bocaege, S. Ricci, F. Boschin, P. Bayle, B. Maureille, F. Le Brun-Ricalens, J.-G. Bordes, G. Oxilia, E. Bortolini, O. Bignon-Lau, G. Debout, M. Orliac, A. Zazzo, V. Sparacello, E. Starnini, L. Sineo, J. van der Plicht, L. Pecqueur, G. Merceron, G. Garcia, J.-M. Leuvrey, C. B. Garcia, A. Gómez-Olivencia, M. Połtowicz-Bobak, D. Bobak, M. Le Luyer, P. Storm, C. Hoffmann, J. Kabaciński, T. Filimonova, S. Shnaider, N. Berezina, B. González-Rabanal, M. R. González Morales, A. B. Marín-Arroyo, B. López, C. Alonso-Llamazares, A. Ronchitelli, C. Polet, I. Jadin, N. Cauwe, J. Soler, N. Coromina, I. Rufí, R. Cottiaux, G. Clark, L. G. Straus, M.-A. Julien, S. Renhart, D. Talaa, S. Benazzi, M. Romandini, L. Amkreutz, H. Bocherens, C. Wißing, S. Villotte, J. F.-L. de Pablo, M. Gómez-Puche, M. A. Esquembre-Bebia, P. Bodu, L. Smits, B. Souffi, R. Jankauskas, J. Kozakaitė, C. Cupillard, H. Benthien, K. Wehrberger, R. W. Schmitz, S. C. Feine, T. Schüler, C. Thevenet, D. Grigorescu, F. Lüth, A. Kotula, H. Piezonka, F. Schopper, J. Svoboda, S. Sázelová, A. Chizhevsky, A. Khokhlov, N. J. Conard, F. Valentin, K. Harvati, P. Semal, B. Jungklaus, A. Suvorov, R. Schulting, V. Moiseyev, K. Mannermaa, A. Buzhilova, T. Terberger, D. Caramelli, E. Altena, W. Haak, J. Krause, Palaeogenomics of Upper Palaeolithic to Neolithic European hunter-gatherers. *Nature* **615**, 117–126 (2023).

28. H. Rathmann, M. T. Vizzari, J. Beier, S. E. Bailey, S. Ghirotto, K. Harvati, Human population dynamics in Upper Paleolithic Europe inferred from fossil dental phenotypes. *Sci. Adv.* **10**, eadn8129 (2024).

29. M. Sikora, A. Seguin-Orlando, V. C. Sousa, A. Albrechtsen, T. Korneliussen, A. Ko, S. Rasmussen, I. Dupanloup, P. R. Nigst, M. D. Bosch, G. Renaud, M. E. Allentoft, A. Margaryan, S. V. Vasilyev, E. V. Veselovskaya, S. B. Borutskaya, T. Deviese, D. Comeskey, T. Higham, A. Manica, R. Foley, D. J. Meltzer, R. Nielsen, L. Excoffier, M. Mirazon Lahr, L. Orlando, E. Willerslev, Ancient genomes show social and reproductive behavior of early Upper Paleolithic foragers. *Science* **358**, 659–662 (2017).

30. J. Zilhão, "The upper Palaeolithic of Europe," in *The Cambridge World Prehistory*, C. Renfrew, P. Bahn, Eds. (Cambridge Univ. Press, 2014), pp. 1753–1785.

31. L. G. Straus, The human occupation of southwestern Europe during the Last Glacial Maximum. *J. Anthropol. Res.* **71**, 465–492 (2015).

32. V. Villalba-Mouco, M. S. van de Loosdrecht, A. B. Rohrlach, H. Fewlass, S. Talamo, H. Yu, F. Aron, C. Lalueza-Fox, L. Cabello, P. Cantalejo Duarte, J. Ramos-Muñoz, C. Posth, J. Krause, G.-C. Weniger, W. Haak, A 23,000-year-old southern Iberian individual links human groups that lived in Western Europe before and after the Last Glacial Maximum. *Nat. Ecol. Evol.* **7**, 597–609 (2023).

33. I. Schmidt, A. Zimmermann, Population dynamics and socio-spatial organization of the Aurignacian: Scalable quantitative demographic data for western and central Europe. *PLOS ONE* **14**, e0211562 (2019).

34. E.-C. Nițu, M. Cârciumaru, A. Nicolae, O. Cîrstina, F. I. Lupu, M. Leu, Mobility and social identity in the Mid Upper Paleolithic: New personal ornaments from Poiana Cireșului (Piatra Neamț, Romania). *PLOS ONE* **14**, e0214932 (2019).

35. D. Garate, O. Rivero, J. Rios-Garaizar, M. Arriolabengoa, I. Intxaurbe, S. Salazar, Redefining shared symbolic networks during the Gravettian in Western Europe: New data from the rock art findings in Aitzbitarte caves (Northern Spain). *PLOS ONE* **15**, e0240481 (2020).

36. G. W. Weber, A. Lukeneder, M. Harzhauser, P. Mitteroecker, L. Wurm, L.-M. Hollaus, S. Kainz, F. Haack, W. Antl-Weiser, A. Kern, The microstructure and the origin of the Venus from Willendorf. *Sci. Rep.* **12**, 2926 (2022).

37. C. Ellis, Measuring Paleoindian range mobility and land-use in the Great Lakes/Northeast. *J. Anthropol. Archaeol.* **30**, 385–401 (2011).

38. R. Schild, Flint mining and trade in Polish prehistory as seen from the perspective of the chocolate flint of Central Europe. *Acta Archaeol. Carpathica* **16**, 147–177 (1976).

39. D. Stefański, J. Wilczyński, Extralocal raw materials in the Swiderian culture: Case study of Kraków-Bieżanów sites. *Anthropologie* **50**, 427–442 (2012).

40. A. Picin, The Beginning of the Early Upper Paleolithic in Poland. *J. Paleolit. Archaeol.* **6**, 12 (2023).

41. M. Sudoł-Procyk, M. Brandl, M. T. Krajcarz, M. Malak, M. Skrzatek, D. Stefański, E. Trela-Kieferling, D. H. Werra, Chocolate flint: New perspectives on its deposits, mining, use and distribution by prehistoric communities in Central Europe. *Antiquity* **95**, 1–7 (2021).

42. S. X. Yang, J. F. Zhang, J. P. Yue, S. Fu, X. Liu, Z. Jia, Y. Hou, D. Cui, H. Miao, M. Rezek, J. Gao, F. Li, W. Wang, D. Wu, Initial Upper Palaeolithic material culture by 45,000 years ago at Shiyu in northern China. *Nat. Ecol. Evol.* **8**, 552–563 (2024).

43. M. Langlais, D. Sacchi, Note sur les matières premières siliceuses exploitées par les magdaléniens de la grotte Gazel (Aude, France) [Note on the siliceous raw materials exploited by the Magdalenian groups of the Gazel cave (Aude, France)], in *Notions de territoire et de mobilité. Exemples de l'Europe et des premières nations en Amérique du Nord avant le contact européen* [*Concepts of territory and mobility: Examples from Europe and the First Nations in North America before European contact*], C. Bressy, A. Burke, P. Chalard, H. Martin, Eds. (ERAUL 116, Liège, 2006), pp. 71–75.

44. L. Slimak, Circulations de matériaux très exotiques au Paléolithique moyen, une notion de détail. *Bull. Soc. préhist. fr.* **105**, 267–281 (2008).

45. A. Tarriño, I. Elorrieta, M. García-Rojas, Flint as raw material in prehistoric times: Cantabrian Mountain and Western Pyrenees data. *Quat. Int.* **364**, 94–108 (2015).

46. M. S. Corchón, P. Ortega, Los niveles solutrenses de la Sala I de la cueva de Las Caldas (25000–21000 cal BP) [The Solutrean levels of Chamber I from Las Caldas cave (25000–21000 cal BP)], in *La Cueva de Las Caldas (Priorio, Oviedo): ocupaciones solutrenses, análisis espaciales y arte parietal* [*Las Caldas cave (Priorio, Oviedo): Solutrean occupations, spatial analyses and parietal art*], M. S. Corchón, Ed. (Ediciones Univ. de Salamanca, 2017), pp. 35–189.

47. E. Vaissié, S. Caux, J.-P. Faivre, Supply distances and territories in southwest France through the Middle and Upper Palaeolithic: A petro-techno-economic approach. *Bull. Soc. Préhist. Fr.* **118**, 7–32 (2021).

48. S. Martín Jarque, R. Montes, M. Álvarez-Alonso, M. Yravedra, M. Marín-Arroyo, Raw materials and lithic production during the early Magdalenian in Cantabrian Spain: Cova Rosa (Ribadesella, Asturias). *J. Paleolit. Archaeol.* **7**, 20 (2024).

49. D. Herrero-Alonso, J.-M. Maíllo-Fernández, N. Abellán-Beltrán, M. Moral, I. González-Molina, I. Solano-Megías, S. Luzón-Ruiz, J. Marín, A. Álvarez-Vena, D. Martín-Perea, A. Neira, F. Bernaldo de Quirós, A. Tarriño, Neanderthal mobility over very long distances: The case of El Castillo cave (northern Spain) and the "Vasconian" Mousterian. *J. Hum. Evol.* **205**, 103715 (2025).

50. L. R. Binford, Organization and formation processes: Looking at curated technologies. *J. Anthropol. Res.* **35**, 255–273 (1979).

51. L. R. Binford, *Constructing Frames of Reference: An Analytical Method for Archaeological Theory Building Using Ethnographic and Environmental Data Sets* (University of California Press, 2001).

52. B. Winterhalder, F. Lu, B. Tucker, Risk-sensitive adaptive tactics: Models and evidence from subsistence studies in biology and anthropology. *J. Archaeol. Res.* **7**, 301–348 (1999).

53. A. Burke, M. Kageyama, G. Latombe, M. Fasel, M. Vrac, G. Ramstein, P. M. A. James, Risky business: The impact of climate and climate variability on human population dynamics in Western Europe during the Last Glacial Maximum. *Quat. Sci. Rev.* **164**, 217–229 (2017).

54. R. A. Gould, S. Saggers, Lithic procurement in Central Australia: A closer look at Binford's idea of embeddedness in archaeology. *Am. Antiq.* **50**, 117–136 (1985).

55. D. B. Bamforth, The Windy Ridge quartzite quarry: Hunter-gatherer mining and hunter-gatherer land use on the North American Continental Divide. *World Archaeol.* **38**, 511–527 (2006).

56. J. D. Speth, Paleoindian bison hunting on the North American great plains—Two critical nutritional constraints. *PaleoAnthropology* **2020**, 74–97 (2020).

57. A. Agam, M. Finkel, Re-thinking the concept of embedded procurement: Insights from the Lower Paleolithic of the Levant. *J. Archaeol. Sci. Rep.* **60**, 104824 (2024).

58. P. U. Clark, A. S. Dyke, J. D. Shakun, A. E. Carlson, J. Clark, B. Wohlfarth, J. X. Mitrovica, S. W. Hostetler, A. M. McCabe, The Last Glacial Maximum. *Science* **325**, 710–714 (2009).

59. M. Alcaraz-Castaño, J. J. Alcolea-González, M. de Andrés-Herrero, S. Castillo-Jiménez, F. Cuartero, G. Cuenca-Bescós, M. Kehl, J. A. López-Sáez, L. Luque, S. Pérez-Díaz, R. Piqué, M. Ruiz-Alonso, G.-C. Weniger, J. Yravedra, First modern human settlement recorded in the Iberian hinterland occurred during Heinrich Stadial 2 within harsh environmental conditions. *Sci. Rep.* **11**, 15161 (2021).

60. T. Aubry, Approches spatiales du solutréen: le dessous des cartes et des plans [Spatial approaches to the Solutrean: Beyond maps and plans], in *Le Solutréen 40 ans après Smith'66. Actes du colloque, Tours, 2007* [The Solutrean 40 Years after Smith '66. Proceedings of the Conference, Tours, 2007] (Suppl. *Revue archéologique du centre de la France* 47, 2013), pp. 199–211.

61. T. J. Koch, P. Schmidt, The unique laurel-leaf points of Volgu document long-distance transport of raw materials in the Solutrean. *Archaeol. Anthropol. Sci.* **14**, 101 (2022).

62. S. Mandera, M. Sudoł-Procyk, M. Malak, M. Skrzatek, M. T. Krajcarz, New deposit of chocolate flint in Załęże gull y (Kraków-Częstochowa Upland, Poland)–Raw material characterization and its availability for prehistoric communities. *J. Archaeol. Sci. Rep.* **53**, 104328 (2024).

63. L. G. Straus, "The original arms race: Iberian perspectives on the Solutrean phenomenon," in *Feuilles de pierre*, J. K. Kozłowski, Ed. (ERAUL 42, 1990), pp. 425–447.

64. J. Zilhão, "Seeing the leaves and not missing the forest: A Portuguese perspective of the Solutrean," in *Pleistocene Foragers on the Iberian Peninsula: Their Culture and Environment*, A. Pastoors, B. Auffermann, Eds. (Neanderthal Museum, 2013), pp. 201–216.

65. J. Cascalheira, N. Bicho, Testing the impact of environmental change on hunter-gatherer settlement organization during the Upper Paleolithic in western Iberia. *J. Quat. Sci.* **33**, 323–334 (2018).

66. M. Séronie-Vivien, M.-R. Séronie-Vivien, *Les Silex du Mésozoïque nord-aquitain. Approche géologique de l'étude des silex pour servir à la recherche préhistorique* [*The Flint from the North-Aquitaine Mesozoic: A Geological Approach to the Study of Flint for Prehistoric Research*] (Supplément au *Bull. Soc. Linn.*, Tome XV, Société Linnéenne, Bordeaux, 1987).

67. A. Turq, Les ressources en matières premières lithiques. *Paléo* **2**, 98–141 (2000).

68. L. Marguet, "*La pétroarchéologie et son application aux jaspéroides de l'Infralias* [*Petroarchaeology and its application to the Infralias jasperoids*]," thesis, University of Bordeaux (2022).

69. P.-Y. Demars, Circulation des silex dans le nord de l'Aquitaine au Paléolithique supérieur: l'occupation de l'espace par les derniers chasseurs-cueilleurs. *Gall. Préhistoire* **40**, 1–28 (1998).

70. J.-G. Bordes, F. Bon, F. Le Brun-Ricalens, Le transport des matières premières lithiques à l'Aurignacien entre le Nord et le Sud de l'Aquitaine [The transport of lithic raw materials during the Aurignacian between the Northern and Southern Aquitaine], in *Territoires, déplacements, mobilité, échanges durant la Préhistoire* [*Territories, Movements, Mobility, and Exchanges during Prehistory*], J. Jaubert, M. Barbaza, Eds. (CTHS, 2005), pp. 185–198.

71. M. Sánchez de la Torre, X. Mangado Llach, S. Castillo-Jiménez, L. Luque, J. J. Alcolea-González, M. Alcaraz-Castaño, New data on chert catchment analysis in inland Iberia during the Late Pleistocene. *Geoarchaeology* **38**, 615–630 (2023).

72. L. M. García-Simón, R. Domingo, The Monegros-type chert: Petrographic characterization and prehistoric use. *J. Lithic Stud.* **3**, 357–374 (2016).

73. C. Renfrew, “Trade as action at a distance: Questions of integration and communication,” in *Ancient Civilization and Trade*, J. A. Sabloff, C. C. Lamberg-Karlovsky, Eds. (University of New Mexico Press, 1975), pp. 3–60.

74. B. Fitzhugh, P. S. Colby, E. Gjesfjeld, “Modeling hunter-gatherer information networks: An archaeological case study from the Kuril Islands,” in *Information and its Role in Hunter-Gatherer Bands*, R. Whallon, W. A. Lovis, R. K. Hitchcock, Eds. (Cotsen Institute of Archaeology Press, 2011), pp. 85–115.

75. A. B. Migliano, L. Vinicius, The origins of human cumulative culture: From the foraging niche to collective intelligence. *Philos. Trans. R. Soc. B* **377**, 20200317 (2022).

76. K. Garg, C. Padilla-Iglesias, N. Restrepo Ochoa, V. B. Knight, Hunter–gatherer foraging networks promote information transmission. *R. Soc. Open Sci.* **8**, 211324 (2021).

77. M. Singh, L. Glowacki, Human social organization during the Late Pleistocene: Beyond the nomadic-egalitarian model. *Evol. Hum. Behav.* **43**, 418–431 (2022).

78. M. Alcaraz-Castaño, MULTIPALEOIBERIA: Hacia un nuevo escenario sobre las dinámicas de poblamiento y adaptaciones culturales de los últimos neandertales y primeros humanos modernos en el interior de la península ibérica [MULTIPALEOIBERIA: Towards a new scenario on population dynamics and cultural adaptations of the last Neandertals and first modern humans in inland Iberia]. *Trab. Prehist.* **80**, e03 (2023).

79. M. García-Rojas, E. Domínguez-Ballesteros, A. Prieto, A. Calvo, A. Sánchez, A. Tarriño, A. Arrizabalaga, A great step forward. Lithic raw material procurement and management among Palaeolithic Hunter-gatherers in the Basque Crossroads. *J. Lithic Stud.* **7**, 19 (2020).

80. M. W. Conkey, A. Beltrán, G. A. Clark, J. G. Echegaray, M. G. Guenther, J. Hahn, B. Hayden, K. Paddayya, L. G. Straus, K. Valoch, The identification of prehistoric hunter-gatherer aggregation sites: The case of Altamira [and comments and reply]. *Curr. Anthropol.* **21**, 609–630 (1980).

81. M. Shott, “Hunter-gatherer aggregation in theory and evidence: The North American paleoindian case,” in *Hunter-Gatherers in Theory and Archaeology*, G. Crothers, Ed. (Southern Illinois Univ., 2004), pp. 68–102.

82. N. Sala, M. Alcaraz-Castaño, M. Arriolabengoa, V. Martínez-Pillado, A. Pantoja-Pérez, A. Rodríguez-Hidalgo, E. Téllez, M. Cubas, S. Castillo, L. J. Arnold, M. Demuro, M. Duval, A. Arteaga-Brieba, J. Llamazares, J. Ochando, G. Cuenca-Bescós, A. B. Marín-Arroyo, M. M. Seijo, L. Luque, C. Alonso-Llamazares, M. Arlegi, M. Rodríguez-Almagro, C. Calvo-Simal, B. Izquierdo, F. Cuartero, L. Torres-Iglesias, L. Agudo-Pérez, A. Arribas, J. S. Carrión, D. Magri, J.-X. Zhao, A. Pablos, Nobody’s land? The oldest evidence of early Upper Paleolithic settlements in inland Iberia. *Sci. Adv.* **10**, eado3807 (2024).

83. J. Cascalheira, M. Alcaraz-Castaño, J. Alcolea-González, M. de Andrés-Herrero, A. Arrizabalaga, J. E. Aura Tortosa, N. Garcia-Ibaibarriaga, M.-J. Iriarte-Chiapusso, Paleoenvironments and human adaptations during the Last Glacial Maximum in the Iberian Peninsula: A review. *Quat. Int.* **581-582**, 28–51 (2021).

84. G.-C. Weniger, M. de Andrés-Herrero, V. Bolin, M. Kehl, T. Otto, A. Potì, Y. Tafelmaier, Late Glacial rapid climate change and human response in the Westernmost Mediterranean (Iberia and Morocco). *PLOS ONE* **14**, e0225049 (2019).

85. J. Aragoncillo-Del Río, J.-J. Alcolea-González, L. Luque, S. Castillo-Jiménez, G. Jiménez-Gisbert, J.-A. López-Sáez, J.-M. Maíllo-Fernández, M. Ruiz-Alonso, I. Triguero, J. Yravedra, M. Alcaraz-Castaño, Human occupations of upland and cold environments in inland Spain during the Last Glacial Maximum and Heinrich Stadial 1: The new Magdalenian sequence of Charco Verde II. *PLOS ONE* **18**, e0291516 (2023).

86. P. Utrilla, L. Montes, C. Mazo, A. Alday, J. M. Rodanés, M. F. Blasco, R. Domingo, M. Bea, El Paleolítico superior en la cuenca del Ebro a principios del siglo XXI [The Upper Palaeolithic in the Ebro basin at the beginnings of the 21st century], in *El Paleolítico superior peninsular. Novedades del siglo XXI. Homenaje al profesor Javier Fortea* [*The Iberian Upper Paleolithic: 21st-Century Developments. A Tribute to Professor Javier Fortea*], X. Mangado, Ed., (Univ. de Barcelona, 2010), pp. 23–61.

87. M. Shott, Technological organization and settlement mobility: An ethnographic examination. *J. Anthropol. Res.* **42**, 15–51 (1986).

88. I. Schmidt, Beyond Solutrean point types. *J. Anthropol. Res.* **71**, 493–508 (2015).

89. B. Gratuze, Obsidian characterization by laser ablation ICP-MS and its application to prehistoric trade in the Mediterranean and the Near East: Sources and distribution of obsidian within the Aegean and Anatolia. *J. Archaeol. Sci.* **26**, 869–881 (1999).

90. B. Gratuze, Application de la spectrométrie de masse à plasma avec prélèvement par ablation laser (LA-ICP-MS) à l'étude des recettes de fabrication et de la circulation des verres anciens [Application of laser ablation inductively coupled plasma mass spectrometry (LA-ICP-MS) to the study of manufacturing recipes and the circulation of ancient glass], in *Circulation des matériaux et des objets dans les sociétés anciennes* [*Circulation of Materials and Objects in Ancient Societies*], P. Dillmann, L. Bellot-Gurlet, Eds. (Éditions Archives Contemporaines, 2014), pp. 165–216.

91. Addinsoft, XLSTAT statistical and data analysis solution (New York, USA, 2022); https://xlstat.com.

92. C. A. Speer, LA-ICP-MS analysis of Clovis period projectile points from the Gault Site. *J. Archaeol. Sci.* **52**, 1–11 (2014).

93. M. Brandl, M. M. Martinez, C. Hauzenberger, P. Filzmoser, P. Nymoen, N. Mehler, A multi-technique analytical approach to sourcing Scandinavian flint: Provenance of ballast flint from the shipwreck "Leirvigen 1", Norway. *PLOS ONE* **13**, e0200647 (2018).

94. M. A. Bustillo, Petrografía y medios sedimentarios de la caliza del páramo (provincia de Madrid) [Petrography and Sedimentary Environments of the Páramo Limestone (Province of Madrid)]. *Bol. Geol. Miner.* **91**, 503–514 (1980).

95. M. E. Arribas, Estudio litoestratigráfico de una unidad de edad paleógena. Sector N de la cuenca terciaria del Tajo (provincia de Guadalajara). *Estud. Geol.* **42**, 103–116 (1986).

96. J. Parcerisas, A. Tarrino, Los sílex de los Páramos del Tajo (sector norte): Avance de una definición formal de aplicación arqueológica [The flint of the Tagus Moorlands (Northern Sector): Toward a formal definition for archaeological application], in *Actas del Segundo Simposio de Arqueología de Guadalajara* [*Proceedings of the Second Symposium on the Archaeology of Guadalajara*], E. García-Soto, M. A. García Valero, J. P. Martínez Naranjo, Eds. (Centro de Profesores de Sigüenza, 2008), pp. 73–88.

97. S. Bárez del Cueto, J. B. Preysler, A. Pérez-González, C. Torres, I. R. Pérez, J. Vega de Miguel, Acheulian flint quarries in the Madrid Tertiary basin, central Iberian Peninsula: First data obtained from geoarchaeological studies. *Quat. Int.* **411**, 329–348 (2016).

98. M. A. Bustillo, J. L. Pérez, Variabilidad litológica y geoquímica de los niveles silíceos del yacimiento arqueológico de Casa Montero y su comparación con otros de la cuenca de Madrid [Lithological and geochemical variability of the siliceous levels of the Casa Montero archaeological site and their comparison with others from the Madrid Basin], in *Geoarqueología del sílex en la Península Ibérica* [*Geoarchaeology of flint in the Iberian Peninsula*], A. Tarrino, A. Morgado, X. Terradas, Eds. (Universidad de Granada, 2016), pp. 11–25.

99. A. Abrunhosa, M. Á. Bustillo, T. Pereira, B. Márquez, A. Pérez-González, J. L. Arsuaga, E. Baquedano, Petrographic and SEM-EDX characterization of Mousterian white/beige chert tools from the Navalmaíllo rock shelter (Madrid, Spain). *Geoarchaeology* **35**, 883–896 (2020).

100. T. Aubry, C. Gameiro, J. Mangado, L. Luis, H. Matis, T. Pereiro, Upper Palaeolithic lithic raw material sourcing in Central and Northern Portugal as an aid to reconstructing hunter-gatherer societies. *J. Lithic Stud.* **3**, 7–28 (2016).

101. M. Mukul, V. Srivastava, S. Jade, M. Mukul, Uncertainties in the shuttle radar topography mission (SRTM) heights: Insights from the Indian Himalaya and peninsula. *Sci. Rep.* **7**, 41672 (2017).

102. W. R. Peltier, R. G. Fairbanks, Global glacial ice volume and Last Glacial Maximum duration from an extended Barbados sea level record. *Quat. Sci. Rev.* **25**, 3322–3337 (2006).

103. D. A. White, The basics of least cost analysis for archaeological applications. *Adv. Archaeol. Pract.* **3**, 407–414 (2015).

104. J. Lewis, Probabilistic modelling for incorporating uncertainty in least cost path results: A postdictive Roman road case study. *J. Archaeol. Method Theory* **28**, 911–924 (2021).

105. M. Llobera, T. J. Sluckin, Zigzagging: Theoretical insights on climbing strategies. *J. Theor. Biol.* **249**, 206–217 (2007).

106. I. I. Ullah, S. M. Bergin, "Modeling the consequences of village site location: Least cost path modeling in a coupled GIS and agent-based model of village agropastoralism in eastern Spain," in *Least Cost Analysis of Social Landscapes: Archaeological Case Studies* (University of Utah Press, 2012), pp. 155–173.

107. S. Fontanari, "*Stiluppo di metodologie GIS per la determinazione dell'accessibilità territoriale come supporto alle decisioni nella gestione ambientale* [*Development of GIS Methodologies for the Determination of Territorial Accessibility as Decision Support in Environmental Management*]," thesis, Università degli Studi di Trento, Facoltà di Ingegneria (2002); https://hdl.handle.net/11582/794.

108. E. Langmuir, *Mountaincraft and Leadership: A Handbook for Mountaineers and Hillwalking Leaders in the British Isles* (Scottish Sports Council, 1995).

109. E. W. Dijkstra, A note on two problems in connexion with graphs. *Numer. Math.* **1**, 269–271 (1959).

110. D. Becker, M. De Andrés-Herrero, C. Willmes, G.-C. Weniger, G. Bareth, Investigating the influence of different DEMs on GIS-based cost distance modeling for site catchment analysis of prehistoric sites in Andalusia. *ISPRS Int. J. Geoinf.* **6**, 36 (2017).

111. GRASS GIS documentation, r.walk manual (2024); https://grass.osgeo.org/grass83/manuals/r.walk.html.

112. J. M. Portero García, J. M. Aznar Aguilera, A. Pérez González, *Mapa Geológico de España 1:50.000, MAGNA n. 486, Jadraque* [*Geological Map of Spain 1:50,000, MAGNA No. 486, Jadraque*] (Instituto Geológico y Minero de España, 1994).

113. J. M. Portero García, J. M. Aznar Aguilera, A. Pérez González, *Mapa Geológico de España 1:50.000, MAGNA n. 485, Valdepeñas de la Sierra* [*Geological Map of Spain 1:50,000, MAGNA No. 485, Valdepeñas de la Sierra*] (Instituto Geológico y Minero de España, 1995).

114. A. Pérez-González, Depresión del Tajo [Tagus depression], in *Geomorfología de España* [*Geomorphology of Spain*], M. Gutiérrez Elorza, Ed. (Rueda, 1994), pp. 389–436.

115. P. G. Silva, E. Roquero, M. López-Recio, P. Huerta, A. M. Martínez-Graña, Chronology of fluvial terrace sequences for large Atlantic rivers in the Iberian Peninsula (Upper Tagus and Duero drainage basins, Central Spain). *Quat. Sci. Rev.* **166**, 188–203 (2017).

116. M. Alcaraz-Castaño, J. Alcolea González, R. De Balbín Behrmann, M. Á. García Valero, J. Y. S. de los Terreros, J. B. Preysler, Los orígenes del Solutrense y la ocupación pleniglaciar del interior de la Península Ibérica: Implicaciones del nivel 3 de Peña Capón (valle del Sorbe, Guadalajara) [The origins of the Solutrean and the pleniglacial occupation of inner Iberia: Implications of the Peña Capón level 3 (Sorbe Valley, Guadalajara, Spain)]. *Trab. Prehist.* **70**, 28–53 (2013).

117. M. Alcaraz-Castaño, J. J. Alcolea-González, R. de Balbín Behrmann, M. Kehl, G. C. Weniger, "Recurrent human occupations in Central Iberia around the Last Glacial Maximum. The Solutrean Sequence of Peña Capón Updated," in *Human Adaptations to the Last Glacial Maximum: The Solutrean and Its Neighbors*, I. Schmidt, J. Cascalheira, Eds. (Cambridge Scholars Publishing, 2019), pp. 148–170.

118. A. Alcaina, M. Alcaraz-Castaño, J. J. Alcolea-González, Espeleología al servicio de la arqueología. Descubrimiento e investigación de los yacimientos de Peña Cabra y Peña Capón (Muriel, Guadalajara) [Speleology in the Service of Archaeology: Discovery and Investigation of the Peña Cabra and Peña Capón Sites (Muriel, Guadalajara)]. *ESPELEOMADRID* 8 (Época III) (2018), pp. 94–98.

119. J. J. Alcolea-González, R. de Balbín, M. A. García Valero, P. J. Jiménez, A. Aldecoa, A. B. Casado, N. Suárez Rueda, Avance al estudio del poblamiento paleolítico del Alto Valle del Sorbe (Muriel, Guadalajara) [Advances in the study of paleolithic settlement in the Upper Sorbe Valley (Muriel, Guadalajara)], in *II Congreso de Arqueología Peninsular I, Paleolítico y Epipaleolítico* [*II Congress on the Archaeology of the Iberian Peninsula I, Paleolithic and Epipaleolithic*], R. de Balbín, P. Bueno, Eds. (Fundación Rei Afonso Henriques, 1997), pp. 201–218.

120. M. Alcaraz-Castaño, Central Iberia around the Last Glacial Maximum. *J. Anthropol. Res.* **71**, 565–578 (2015).

121. J. Yravedra, M.-A. Julien, M. Alcaraz-Castaño, V. Estaca-Gómez, J. Alcolea-González, R. de Balbín-Behrmann, C. Lécuyer, C. H. Marcel, A. Burke, Not so deserted… paleoecology and human subsistence in Central Iberia (Guadalajara, Spain) around the Last Glacial Maximum. *Quat. Sci. Rev.* **140**, 21–38 (2016).

122. P. Smith, *Le Solutréen en France* [The Solutrean in France] (Delmas, 1966).

123. J. M. Fullola, El Solutreo-Gravetiense o Parpallense, industria mediterránea [The Solutreo-Gravettian or Parpallean, Mediterranean industry]. *Zephyrus* **28-29**, 125–133 (1978).

124. L. G. Straus, *El solutrense vasco-cantábrico. Una nueva perspectiva* [The Vasco-Cantabrian Solutrean. A new perspective] (Monografías/Centro Nacional de Investigación y Museo de Altamira 10, 1983).

125. M. Rasilla, El Solutrense en el contexto del Paleolítico Superior Occidental [The Solutrean in the context of the Western Upper Palaeolithic]. *Férvedes* **1**, 9–19 (1994).

126. J. Zilhão, *O Paleolítico Superior da Estremadura portuguesa* [*The Upper Palaeolithic of the Portuguese Estremadura*], vol. I–II (Edições Colibri, 1997).

127. J. Cascalheira, N. Bicho, On the chronological structure of the Solutrean in Southern Iberia. *PLOS ONE* **10**, e0137308 (2015).

128. N. Bicho, J. Cascalheira, J. Marreiros, T. Pereira, Rapid climatic events and long term cultural change: The case of the Portuguese Upper Paleolithic. *Quat. Int.* **428**, 3–16 (2017).

129. F. Almeida, "The terminal Gravettian of Portuguese Estremadura: Technological Variability of the Lithic Industries," thesis, Southern Methodist University, Dallas, TX (2000).

130. J. Belmiro, N. Bicho, J. Haws, J. Cascalheira, The Gravettian–Solutrean transition in westernmost Iberia: New data from the sites of Vale Boi and Lapa do Picareiro. *Quat. Int.* **587-588**, 19–40 (2021).

131. L. R. Binford, Willow smoke and dogs' tails: Hunter-gatherer settlement systems and archaeological site formation. *Am. Antiq.* **45**, 4–20 (1980).

132. C. Perreault, P. J. Brantingham, Mobility-driven cultural transmission along the forager–collector continuum. *J. Anthropol. Archaeol.* **30**, 62–68 (2011).

133. M. Alcaraz-Castaño, M. López-Recio, F. Tapias, F. Cuartero, J. Baena, B. Ruiz-Zapata, J. Morín, A. Pérez-González, M. Santonja, The human settlement of Central Iberia during MIS 2: New technological, chronological and environmental data from the Solutrean workshop of Las Delicias (Manzanares River valley, Spain). *Quat. Int.* **431**, 104–124 (2017).

134. J. J. Alcolea-González, M. Alcaraz-Castaño, Nuevas investigaciones en la cueva de Los Casares (Riba de Saelices, Guadalajara). Un proyecto de estudio integral para un yacimiento clásico del Paleolítico ibérico [New Research in Los Casares Cave (Riba de Saelices, Guadalajara): An Integrated Study Project for a Classic Site of the Iberian Palaeolithic], in *Actualidad de la investigación arqueológica en España II (2019–2020). Conferencias impartidas en el Museo Arqueológico Nacional* [*Current Archaeological Research in Spain II (2019–2020): Lectures Delivered at the National Archaeological Museum*], A. Carretero, C. Papi, Eds. (Ministerio de Cultura y Deporte, 2020), pp. 169–184.

135. J. J. Alcolea-González, M. Alcaraz-Castaño, I. Triguero, L. Luque, La cueva de Los Casares (Riba de Saelices, Guadalajara): Primeros resultados de la revisión de su Arte Parietal Paleolítico [Los Casares Cave (Riba de Saelices, Guadalajara): First results of the review of its paleolithic parietal art], in *Imágenes de una sociedad. Estudios sobre el Paleolítico*

superior en homenaje al profesor César González Sainz [*Images of a Society: Studies on the Upper Paleolithic in Honor of Professor César González Sainz*], D. Garate Maidagan, J. E. González Urquijo, Eds. (Editorial Tantín, 2025), pp. 21–39.

136. F. L. López Olmedo, J. P. Palacio Suárez, M. D. T. Dávila Ruiz, F. Luís López, E. García Rojo, M. Martínez Cano, P. Monzón Lara, *Mapa Geológico Digital continuo E. 1:50.000, Zona Ibérica (Zona-1700)* [*Continuous Digital Geological Map 1:50,000, Iberian Zone (Zone-1700)*] (GEODE, Instituto Geológico y Minero de España, Online, 2025); https://info.igme.es/cartografiadigital/geologica/geode.aspx.

137. J. M. Portero García, J. M. Aznar Aguilera, A. Pérez González, *Mapa Geológico de España, E. 1:50.000, Jadraque (486)* [*Geological Map of Spain 1:50,000, Jadraque (486)*] (Instituto Geológico y Minero de España, 1983).

138. J. M. Aznar Aguilera, J. M. Portero García, A. Pérez González, *Mapa Geológico de España, E. 1:50.000, Brihuega (511)* [*Geological Map of Spain 1:50,000, Brihuega (511)*] (Instituto Geológico y Minero de España, 1984).

139. M. Montes, A. Salazar, J. I. Ramírez, F. Nozal, F. L. López Olmedo, *Mapa Geológico Digital continuo E. 1:50.000, Zona Tajo-Mancha (Zona-2400)* [*Continuous Digital Geological Map 1:50,000, Tagus-Mancha Zone (Zone-2400)*] (GEODE, Instituto Geológico y Minero de España, Online, 2025); https://info.igme.es/cartografiadigital/geologica/geode.aspx.

140. A. Pineda, *Mapa Geológico Digital continuo E. 1:50.000, Zona Cuenca del Duero-Almazán (Zona-2300)* [*Continuous Digital Geological Map 1:50,000, Duero-Almazán Basin Zone (Zone-2300)*] (GEODE, Instituto Geológico y Minero de España, Online, 2025); https://info.igme.es/cartografiadigital/geologica/geode.aspx.

141. M. Sánchez de la Torre, L. M. García-Simón, F.-X. Le Bourdonnec, R. Domingo, Geochemical fingerprinting of Monegros cherts: Redefining the origin of a prehistoric tracer. *Archaeometry* **61**, 1233–1245 (2019).

142. M. N. Fuertes-Prieto, A. Neira-Campos, E. Fernández-Martínez, F. Gómez-Fernández, E. Alonso-Herrero, “Mucientes chert” in the northern Iberian plateau (Spain). *J. Lithic Stud.* **1**, 117–135 (2014).

143. J. Quirantes, *Estudio sedimentológico y estratigráfico del Terciario continental de Los Monegros* [*Sedimentological and stratigraphic study of the Continental Tertiary of Los Monegros*] (Institución Fernando el Católico, 1978).

144. I. Barandiarán, Un taller de piedras de fusil en el Ebro Medio [A Gunflint Workshop in the Middle Ebro]. *Cuad. Etnol. Etnogr. Nav.* **6**, 189–228 (1974).

145. A. Tarriño, M. Bea, L. M. García-Simón, F. Pérez-Lambán, R. Domingo, Centros de explotación de sílex en la zona centro del Valle del Ebro. La Muela (Zaragoza) [Chert exploitation centers in the Central Zone of the Ebro Valley: La Muela (Zaragoza)], in *Geoarqueología del sílex en la Península Ibérica* [*Geoarchaeology of chert in the Iberian Peninsula*], A. Tarriño, A. Morgado, X. Terradas, Eds. (Universidad de Granada, 2016), pp. 229–243.

146. J. C. Lothrop, A. L. Burke, S. Winchell-Sweeney, G. Gauthier, Coupling lithic sourcing with least cost path analysis to model Paleoindian pathways in northeastern North America. *Am. Antiq.* **83**, 462–484 (2018).

147. L. M. Jiménez, X. Mangado, C. B. González, F.-X. Le Bourdonnec, B. Gratuze, J. M. Fullola, M. Sánchez de la Torre, Patterns of lithic procurement strategies in the Pre-Pyrenean Middle Magdalenian sequence of Cova del Parco (Alòs de Balaguer, Spain). *Geoarchaeology* **39**, 453–469 (2024).

148. C. Gravel-Miguel, C. D. Wren, Agent-based least-cost path analysis and the diffusion of Cantabrian Lower Magdalenian engraved scapulae. *J. Archaeol. Sci.* **99**, 1–9 (2018).

149. R. J. Hewitt, M. Alcaraz-Castaño, V. C. Hernandez, M. W. Morley, Modelling mobility of Hunter-gatherer populations: A dynamic simulation approach based on cellular automata. *J. Archaeol. Method Theory* **32**, 57 (2025).

150. D. A. Raichlen, B. M. Wood, A. D. Gordon, A. Z. P. Mabulla, F. W. Marlowe, H. Pontzer, Evidence of Lévy walk foraging patterns in human hunter–gatherers. *Proc. Natl. Acad. Sci. U.S.A.* **111**, 728–733 (2014).

151. L. R. Binford, The archaeology of place. *J. Anthropol. Archaeol.* **1**, 5–31 (1982).

152. A. Prieto, M. García-Rojas, A. Sánchez, A. Calvo, E. Dominguez-Ballesteros, J. Ordoño, M. I. García-Collado, Stones in Motion: Cost units to understand flint procurement strategies during the Upper Palaeolithic in the south-western Pyrenees using GIS. *J. Lithic Stud.* **3**, 133–160 (2015).

153. V. Delvigne, A. Lafarge, P. Fernandes, D. Pesesse, R. Angevin, P. Bindon, M. Langlais, M. Piboule, A. Queffelec, C. Tuffery, J. P. Raynal, Quels territoires en préhistoire ? Une analyse par réseaux de lieux pour penser l'espace au Paléolithique supérieur [Which Territories in Prehistory? A Place-Network Analysis for Understanding Space in the Upper Paleolithic]. *Bull. Soc. Préhist. Fr.* **118**, 27–69 (2021).

154. B. Winterhalder, Diet choice, risk, and food sharing in a stochastic environment. *J. Anthropol. Archaeol.* **5**, 369–392 (1986).

155. E. Hertell, M. Tallavaara, "High mobility or gift exchange: Early Mesolithic exotic chipped lithics in Southern Finland," in *Mesolithic Interfaces: Variability in Lithic Technologies in Eastern Fennoscandia*, T. Rankama, Ed. (Monographs of the Archaeological Society of Finland 1, 2011), pp. 10–41.

156. J. Zilhão, T. Aubry, La pointe de Vale Comprido et les origines du Solutréen [The Vale Comprido point and the origins of the Solutrean]. *Anthropologie* **99**, 125–142 (1995).

157. C. Renard, Continuity or discontinuity in the Late Glacial Maximum of south-western Europe: The formation of the Solutrean in France. *World Archaeol.* **43**, 726–743 (2011).

158. L. G. Straus, El Paleolítico Superior de la península ibérica. *Trab. Prehist.* **75**, 9–51 (2018).

159. W. E. Banks, P. Bertran, S. Ducasse, L. Klaric, P. Lanos, C. Renard, M. Mesa, An application of hierarchical Bayesian modeling to better constrain the chronologies of Upper

Paleolithic archaeological cultures in France between ca. 32,000–21,000 calibrated years before present. *Quat. Sci. Rev.* **220**, 188–214 (2019).

160. L. R. Binford, *Nunamiut Ethnoarchaeology* (Academic Press, 1978).

161. R. Torrence, *Production and Exchange of Stone Tools: Prehistoric Obsidian in the Aegean* (Cambridge Univ. Press, 2009).

162. H. M. Wobst, Boundary conditions for Paleolithic social systems: A simulation approach. *Am. Antiq.* **39**, 147–178 (1974).

163. D. Boric, E. Cristiani, "Social networks and connectivity among the Palaeolithic and Mesolithic foragers of the Balkans and Italy," in *Southeast Europe Before Neolithisation*, R. Krauss, H. Floss, Eds. (RessourcenKulturen 1, Univ. Tübingen, 2014), pp. 73–112.

164. C. Seong, J. Kim, Moving in and moving out: Explaining final Pleistocene–Early Holocene hunter-gatherer population dynamics on the Korean Peninsula. *J. Anthropol. Archaeol.* **66**, 101407 (2022).

165. L. Pericot, J. Fullola, El Solutrense ibérico [The Iberian Solutrean], in *Préhistoire africaine. Mélanges offerts au doyen Lionel Balout* [*African Prehistory: Essays Presented to Dean Lionel Balout*] (Editions D.P., 1981), pp. 41–44.

166. P. Bertran, L. Sitzia, W. E. Banks, M. D. Bateman, P. Y. Demars, M. Hernandez, M. Lenoir, N. Mercier, F. Prodeo, The Landes de Gascogne (southwest France): Periglacial desert and cultural frontier during the Palaeolithic. *J. Archaeol. Sci.* **40**, 2274–2285 (2013).

167. P. Utrilla, R. Domingo, L. Montes, C. Mazo, J. M. Rodanés, F. Blasco, A. Alday, The Ebro Basin in NE Spain: A crossroads during the Magdalenian. *Quat. Int.* **272-273**, 88–104 (2012).

168. P. Utrilla, C. Mazo, R. Domingo, Fifty thousand years of prehistory at the cave of Abauntz (Arraitz, Navarre): A nexus point between the Ebro Valley, Aquitaine and the Cantabrian Corridor. *Quat. Int.* **364**, 294–305 (2015).

169. J. J. Alcolea-González, R. de Balbín, M. A. García, P. J. Jiménez, Nouvelles decouvertes d'Art Pariétal Paléolithique á la Meseta: La grotte del Reno (Valdesotos, Guadalajara) [New discoveries of Palaeolithic parietal art in the Plateau: El Reno cave (Valdesotos, Guadalajara)]. *Anthropologie* **101**, 144–163 (1997).

170. J. J. Alcolea-González, R. de Balbín-Behrmann, El Arte rupestre Paleolítico del interior peninsular [Palaeolithic rock art in interior Iberia], in *Arte sin artistas, una mirada al Paleolítico* [*Art Without Artists: A View on the Paleolithic*] (Museo Arqueológico Regional, Comunidad de Madrid, 2012), pp. 187–207.

171. R. de Balbín-Behrmann, Estado actual de la investigación del Arte Paleolítico en Guadalajara [Current state of research on paleolithic art in Guadalajara], in *Actas del Primer Simposio de Arqueología de Guadalajara* [*Proceedings of the First Symposium on the Archaeology of Guadalajara*] (Ayuntamiento de Sigüenza, 2002), vol. 1, pp. 187–228.

172. R. Balbín-Behrmann, J. J. Alcolea-González, La grotte de Los Casares et l'art paléolithique de la Meseta espagnole [Los Casares Cave and the Paleolithic Art of the Spanish Meseta]. *Anthropologie* **6**, 397–451 (1992).

173. T. Aubry, J. D. Sampaio, Fariseu: Cronologia e interpretaçâo funcional do sitio [Fariseu: Chronology and Functional Interpretation of the Site], in *Actas do III Congresso de Arqueologia de Tras-os-Montes, Alto Douro e Beira Interior* [*Proceedings of the III Congress of Archaeology of Trás-os-Montes, Alto Douro, and Beira Interior*] (Associação Cultural Desportiva e Recreativa de Freixo de Numão, 2008), pp. 7–30.

174. H. Breuil, H. Obermaier, *La Cueva de Altamira en Santillana del Mar* [*Altamira cave in Santillana del Mar*] (Ediciones El Viso, 1984).

175. C. de las Heras, J. A. Lasheras, La cueva de Altamira [Altamira cave], in *Los cazadores-recolectores del Pleistoceno y el Holoceno en Iberia y el Estrecho de Gibraltar* [*Pleistocene and Holocene Hunter-Gatherers in Iberia and the Strait of Gibraltar*], R. Sala, Ed. (Universidad de Burgos, 2014), pp. 615–627.

176. A. Leroi-Gourhan, J. Allain, Eds., *Lascaux inconnu* [*Unknown Lascaux*]. *Gallia préhistoire. Supplément* 12 (Centre National de la Recherche Scientifique, 1979).

177. J. Gaussen, *La Grotte ornée de Gabillou [The decorated cave of Gabillou]. Publication de l'Institut de Préhistoire de la Université de Bordeaux*, no. 3 (Université de Bordeaux, 1964).

178. J. Gaussen, Grotte de Gabillou [Gabillou Cave], in *L'art des cavernes. Atlas des grottes ornées paléolithiques françaises* [*The Art of the Caves: Atlas of French Paleolithic Decorated Caves*] (Ministère de la Culture, 1984), pp. 225–231.

179. B. Delluc, G. Delluc, G. Delorme, D. Genty, H. Valladas, M. Patou-Mathis, S. A. de Beaune, La grotte ornée de Villars (Dordogne). Révision de la décoration et apports nouveaux [The Painted Cave of Villars (Dordogne): Review of the Decoration and New Contributions]. *Préhistoire du Sud-Ouest* **24**, 97–157 (2016).

180. N. Aujoulat, J.-J. Cleyet-Merle, J. Gaussen, N. Tisnerat, H. Valladas, Approche chronologique de quelques sites ornés paléolithiques du Périgord par datation carbone 14, en spectrométrie de masse par accélérateur, de leur mobilier archéologique/chronological approach to a few decorated Paleolithic Sites in the Perigord based on C14 dating of associated archaeological objects. *Paléo* **10**, 319–323 (1998).

181. S. Ducasse, M. Langlais, Twenty years on, a new date with Lascaux. Reassessing the chronology of the cave's Paleolithic occupations through new 14C AMS dating. *Paléo* **30–1**, 130–147 (2019).

182. N. Aujoulat, *Lascaux. Gesture, space and time* [*Lascaux: Gesture, Space, and Time*] (Seuil, 2004).

183. F. Djindjian, "Art during the Last Glacial Maximum in Western Europe," in *The Grotte du Placard at 150. New considerations on an exceptional prehistoric site*, F. Delage, Ed. (Archaeopress Archaeology, 2018), pp. 170–185.

184. A. Leroi-Gourhan, Grotte de Lascaux [Lascaux Cave], in *L'art des cavernes. Atlas des grottes ornées paléolithiques françaises* [*The Art of the Caves: Atlas of French Paleolithic Decorated Caves*] (Ministère de la Culture, 1984), pp. 180–200.

185. P. G. Bahn, Lascaux: Composition or accumulation? *Zephyrus XLVII* **3–13**, (1994).

186. N. Aujoulat, Le Fourneau du Diable, in *L'art des cavernes. Atlas des grottes ornées paléolithiques françaises* [*The Art of the Caves: Atlas of French Paleolithic Decorated Caves*] (Ministère de la Culture, 1984), pp. 89–91.

187. S. Tymula, *L'art solutréen du Roc de Sers (Charente)* [*The Solutrean art of Roc de Sers (Charente)*] (Éditions de la Maison des sciences de l'homme, 2002).

188. B. Delluc, G. Delluc, La Grotte ornée de Sous-Grand-Lac (Dordogne). *Gallia Préhistoire* **14**, 245–252 (1971).

189. J. Zilhão, Vers une chronologie plus fine du cycle ancien de l'art paléolithique de la Côa: quelques hypothèses de travail [Toward a finer chronology of the early cycle of paleolithic art in the Côa: Some working hypotheses], in *El Arte Prehistórico desde los inicios del siglo XXI. Primer Symposium Internacional de Arte Prehistórico de Ribadesella* [*Prehistoric Art from the Beginnings of the 21st Century: First International Symposium on Prehistoric Art of Ribadesella*], R. de Balbín, P. Bueno, Eds. (Asociación Cultural de Amigos de Ribadesella, 2001), pp. 75–90.

190. A. Laming-Emperaire, Art rupestre et organisation sociale [Rock art and social organization], in *Santander Symposium*, M. Almagro Basch, M. A. García Guinea, Eds. (Patronato de las Cuevas Prehistóricas de la Provincia de Santander, Santander, 1972), pp. 65–79.

191. E. Callahan, *The Basics of Biface Knapping in the Eastern Fluted Point Tradition: A Manual for Flintknappers and Lithic Analysts* (Piltdown Productions, ed. 4, 2000).

192. J.-M. Geneste, H. Plisson, Le Solutréen de la grotte de Combe Saunière 1 (Dordogne). Première approche palethnologique. *Gall. Préhistoire* **29**, 9–27 (1986).

193. H. Plisson, J.-M. Geneste, Analyse technologique des pointes à cran solutréennes du Placard (Charente), du Fourneau du Diable, du Pech de la Boissiere et de Combe-Saunière (Dordogne) [Technological analysis of Solutrean shouldered points from Placard (Charente), Fourneau du Diable, Pech de la Boissiere and Combe-Saunière (Dordogne)]. *Paléo* **1**, 65–106 (1989).

194. W. E. Banks, "Artifacts as landscapes: A use-wear case study of Upper Paleolithic assemblages at the Solutré Kill Site, France," in *Confronting Scale in Archaeology*, G. Lock, B. L. Molyneaux, Eds. (Springer, 2006), pp. 89–111.

195. J. F. Gibaja, N. Bicho, Provenience, technology, morphology and the use of proto-solutrean and solutrean points from Vale Boi (Algarve, southern Portugal): Preliminary results. *Supplément à la Revue archéologique du centre de la France* **47**, 185–196 (2013).

196. L. H. Keeley, *Experimental Determination of Stone Tool Uses: A Microwear Analysis* (University of Chicago Press, 1980).

197. C. Gutiérrez Sáez, Introducción a las huellas de uso: Los resultados de la experimentación [Introduction to use-wear: Results from experimentation]. *Espac. Tiempo Forma Ser. Prehist. Arqueol.* **3**, 15–54 (1990).

198. J. González Urquijo, J. J. Ibáñez Estévez, Metodología de análisis funcional de instrumentos tallados en sílex [Methodology of functional analysis of flint knapped artefacts]. *Cuadernos de arqueología de Deusto* 14 (Universidad de Deusto, Servicio de publicaciones, 1994).

199. N. Taipale, V. Rots, Every hunter needs a knife: Hafted butchering knives from Maisières-Canal and their effect on lithic assemblage characteristics. *J. Archaeol. Sci. Rep.* **36**, 102874 (2021).

200. A. F. Pawlik, Microscopic use-wear analysis: A basic introduction on how to reconstruct the functions of stone tools. *Hukay* **3**, 1–25 (2001).

201. N. Kononenko, Experimental and archaeological studies of use-wear and residues on obsidian artefacts from Papua New Guinea. *Tech. Rep. Aust. Mus. Online* **21**, 1–244 (2011).

202. G. Bustos-Pérez, S. Díaz, J. Baena, An experimental approach to degrees of rounding among lithic artifacts. *J. Archaeol. Method Theory* **26**, 1243–1275 (2019).

203. V. Rots, *Prehension and Hafting Wear on Flint Tools. A Methodology* (Leuven University Press, 2010).

204. N. Mazzucco, I. Clemente Conte, “Lithic tools transportation: New experimental data,” in *Experimentación en Arqueología. Estudio y difusión del pasado. Sèrie Monogràfica del MAC*, 25, A. Palomo, R. Piqué, X. Terradas, Eds. (Museu d’Arqueologia de Catalunya, 2013), pp. 237–245.

205. L. Kaňáková, Lithic arrowheads of the Nitra culture–The use of actual and experimental use-wear analyses to identify the differential effects of quiver transportation. *Lithic Technol.* **45**, 283–294 (2020).

206. D. Visentin, M. Cecchetti, F. Fontana, I. Clemente Conte, Transport wear and its role in understanding prehistoric lithic raw materials management strategies. *J. Archaeol. Sci. Rep.* **61**, 104953 (2025).

207. F. d’Errico, “Identification des traces de manipulation, suspension, polissage sur l’art mobilier en os, bois de cervidés, ivoire,” in *Traces et fonction: les gestes retrouvés, Colloque international de Liège 1993. ERAUL* 50, S. Beyres, M. Otte, H. Plisson, Eds. (Université de Liège, 1993), pp. 177–187.

208. M. Dauvois, *Précis de dessin dynamique et structural des industries lithiques préhistoriques* [*Handbook of Dynamic and Structural Drawing of Prehistoric Lithic Industries*], P. Fanlac, Ed. (CNRS, 1976).

209. J. Baena, F. Cuartero, Más allá de la tipología lítica: lectura diacrítica y experimentación como claves para la reconstrucción del proceso tecnológico [Beyond lithic typology: Diachronic analysis and experimentation as keys for reconstructing the technological process], in *Miscelánea en homenaje a Victoria Cabrera* [*Miscellany in Honor of Victoria Cabrera*]. *Vol. I, Zona Arqueológica* 7, J. M. Maíllo, E. Baquedano, Eds. (Museo Arqueológico Regional, 2006), pp. 144–161.

210. N. Castañeda, Diagrama de secuencias de reducción (DSR): aproximación metodológica para el análisis de núcleos líticos y remontajes [Reduction sequence diagram (RSD): Methodological approach for the analysis of lithic cores and reassemblies]," in *Seis décadas de tipología analítica: actas en homenaje a Georges Laplace* [*Six Decades of Analytical Typology: Proceedings in Honor of Georges Laplace*], A. Calvo, A. Sánchez, M. García-Rojas, M. Alonso, Eds. (Universidad del País Vasco, 2015), pp. 92–104.

211. C. Bronk Ramsey, Bayesian analysis of radiocarbon dates. *Radiocarbon* **51**, 337–360 (2009).

212. P. Reimer, W. E. N. Austin, E. Bard, A. Bayliss, P. G. Blackwell, C. Bronk Ramsey, M. Butzin, H. Cheng, R. L. Edwards, M. Friedrich, P. M. Grootes, T. P. Guilderson, I. Hajdas, T. J. Heaton, A. G. Hogg, K. A. Hughen, B. Kromer, S. W. Manning, R. Muscheler, J. G. Palmer, C. Pearson, J. van der Plicht, R. W. Reimer, D. A. Richards, E. M. Scott, J. R. Southon, C. S. M. Turney, L. Wacker, F. Adolphi, U. Büntgen, M. Capano, S. M. Fahrni, A. Fogtmann-Schulz, R. Friedrich, P. Köhler, S. Kudsk, F. Miyake, J. Olsen, F. Reinig, M. Sakamoto, A. Sookdeo, S. Talamo, The IntCal20 Northern Hemisphere radiocarbon age calibration curve (0–55 cal kBP). *Radiocarbon* **62**, 725–757 (2020).

213. C. Bronk Ramsey, Dealing with outliers and offsets in radiocarbon dating. *Radiocarbon* **51**, 1023–1045 (2009).

214. S. O. Rasmussen, M. Bigler, S. P. Blockley, T. Blunier, S. L. Buchardt, H. B. Clausen, I. Cvijanovic, D. Dahl-Jensen, S. J. Johnsen, H. Fischer, V. Gkinis, M. Guillevic, W. Z. Hoek, J. J. Lowe, J. B. Pedro, T. Popp, I. K. Seierstad, J. P. Steffensen, A. M. Svensson, P. Vallelonga, B. M. Vinther, M. J. C. Walker, J. J. Wheatley, M. Winstrup, A stratigraphic framework for abrupt climatic changes during the Last Glacial period based on three synchronized Greenland ice-core records: Refining and extending the INTIMATE event stratigraphy. *Quat. Sci. Rev.* **106**, 14–28 (2014).

215. M. F. Sánchez Goñi, S. P. Harrison, Millennial-scale climate variability and vegetation changes during the Last Glacial: Concepts and terminology. *Quat. Sci. Rev.* **29**, 2823–2827 (2010).

216. A. Leroi-Gourhan, La Nef et le Diverticule des Félins [The nave and the niche of the felines], in *Lascaux inconnu* [*Unknown Lascaux*]. *Gallia préhistoire. Supplément 12*, A. Leroi-Gourhan, J. Allain, Eds. (Centre National de la Recherche Scientifique, 1979), pp. 301–342.

217. D. Vialou, Le Passage et l'Abside [The passage and the apse], in *Lascaux inconnu* [*Unknown Lascaux*]. *Gallia préhistoire. Supplément 12*, A. Leroi-Gourhan, J. Allain, Eds. (Centre National de la Recherche Scientifique, 1979), pp. 191–300.
